# Brønsted Acid-Catalyzed Synthesis of 4-Functionalized Tetrahydrocarbazol-1-ones from 1,4-Dicarbonylindole Derivatives

**DOI:** 10.1021/acs.joc.3c02248

**Published:** 2023-12-12

**Authors:** Sara Gómez-Gil, Marta Solas, Samuel Suárez-Pantiga, Roberto Sanz

**Affiliations:** Área de Química Orgánica, Departamento de Química, Facultad de Ciencias, Universidad de Burgos, Pza. Misael Bañuelos s/n, 09001-Burgos, Spain

## Abstract

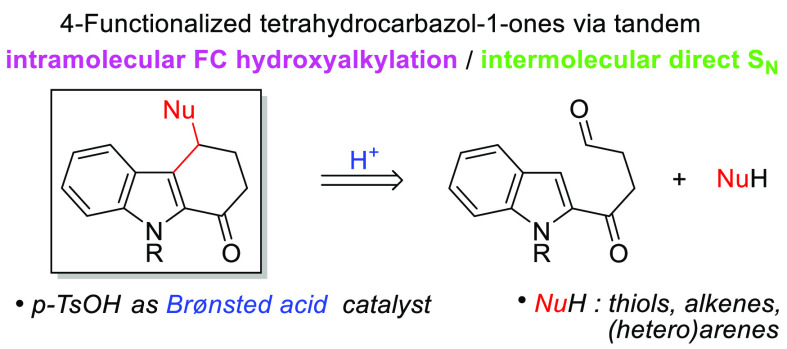

A *p*-toluenesulfonic acid-catalyzed cascade reaction is reported for the synthesis of 4-functionalized tetrahydrocarbazolones via the reaction of 4-(indol-2-yl)-4-oxobutanal derivatives with a variety of nucleophiles in acetonitrile or hexafluoroisopropanol. After the initial intramolecular Friedel–Crafts hydroxyalkylation, the 3-indolylmethanol intermediate is subsequently activated and reacted with the external nucleophile. The reaction conditions are crucial to avoid alternative reaction pathways, allowing direct substitution reaction with thiols, (hetero)arenes, alkenes, or sulfinates. The procedure features high overall yields to access a diverse family of compounds bearing the tetrahydrocarbazole core.

## Introduction

**Scheme 1:**
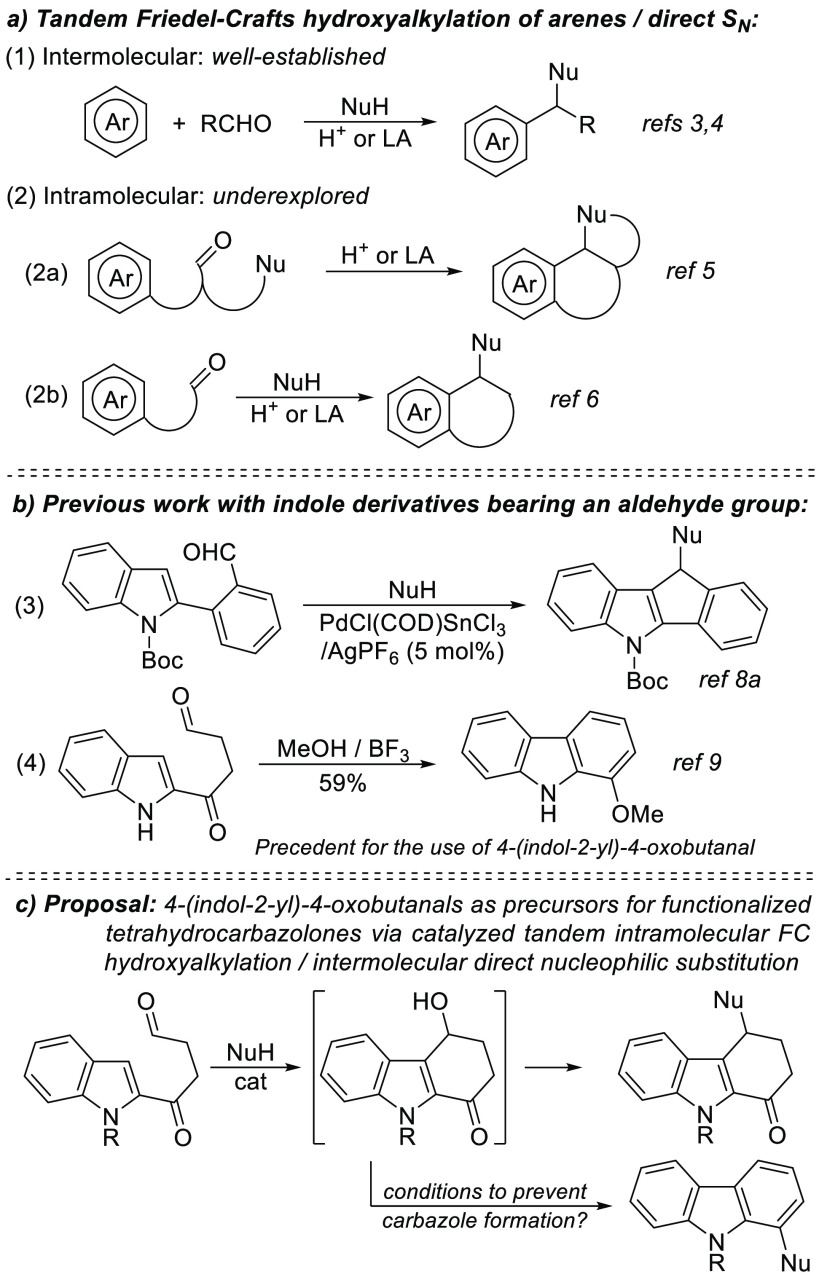
Background, Previous Work and Proposed Tetrahydrocarbazolone Synthesis from 4-(Indol-2-yl)-4-oxobutanals

Friedel–Crafts (FC) alkylation represents a key tool for the functionalization of (hetero)arenes and the preparation of relevant aromatic compounds in organic synthesis.^1^ Recent advances in the field are mainly related to the development of suitable mild catalysts and the use of alternative and more environmentally friendly electrophilic partners such as alcohols, aldehydes and ketones.^2^ The intermolecular Brønsted or Lewis acid-catalyzed reaction between aldehydes and arenes (Scheme 1a, eq 1) represents a useful tool for the construction of relevant triarylmethanes or 1,1,-diarylalkanes (when NuH = Ar)^3^ or benzyl-functionalized substrates (for other NuH).^4^ This type of reaction is a tandem process consisting of a FC hydroxyalkylation followed by direct nucleophilic substitution of the resulting alcohol, which can also be considered as a FC alkylation. However, the version in which one of the steps proceeded intramolecularly remains underexplored, even though this strategy provides efficient access to the construction of unsymmetrical cyclized products. In this case, the other step could also take place intramolecularly (Scheme 1a, eq 2a),^5^ or with an external nucleophile (Scheme 1a, eq 2b).^6^

On the other hand, the indole core is ubiquitous in numerous biologically active compounds, and therefore the development of methodologies for direct indole functionalization is an open challenge in organic synthesis, in which its FC reaction through the nucleophilic C3 position is one of the most useful approaches.^7^

In this field, previous work involving the methodology shown in eq 2b, with indole derivatives bearing an aldehyde group, is limited to a few examples where the aldehyde is aromatic, leading to five-membered rings and requiring the use of metallic catalysts (Scheme 1b, eq 3).^8^ A related substrate, such as 4-(indol-2-yl)-4-oxobutanal, has been prepared by Moody et al., but it cyclizes to 1-methoxycarbazol on treatment with BF_3_/MeOH, where the external nucleophile attacks the ketone instead of the double reaction with the aldehyde (Scheme 1b, eq 4).^9^

In this context, and following our interest in the development of Brønsted acid-catalyzed functionalization of indole derivatives via direct nucleophilic substitution reactions,^10^ we proposed to switch the reactivity described for 4-(indol-2-yl)-4-oxobutanals, preventing their carbazole formation, and thus allowing the synthesis of functionalized tetrahydrocarbazolones via the strategy established in eq 2b, i.e. a tandem intramolecular FC hydroxyalkylation/intermolecular direct S_N_ (Scheme 1c).

The tetrahydrocarbazole scaffold,^11^ including tetrahydrocarbazolone derivatives, is an important structural motif that appears in various molecules with biological activity,^12^ and has been shown to be a useful intermediate platform for the synthesis of several carbazole derivatives.^13^ Thus, the development of new approaches to functionalized tetrahydrocarbazolones, in addition to the classical Fischer indole synthesis with arylhydrazones derived from 1,2-cyclohexanediones, α-aminocylcohexanones, or generated via the Japp–Klingemann,^14^ intramolecular FC of indolecarboxylic acids,^15^ and the oxidation of tetrahydrocarbazoles,^16^ remains an interesting goal in the field.^17^ In fact, only particular examples of tetrahydrocarbazol-1-ones further functionalized at C-4 have recently been reported in the literature,^18^ and to the best of our knowledge, there is no specific synthetic route for their preparation.

Herein, we present our results on the development of an efficient strategy for the synthesis of 4-functionalized tetrahydrocarbazol-1-ones from readily available 4-(indol-2-yl)-4-oxobutanals.

## Results and Discussion

**Scheme 2:**
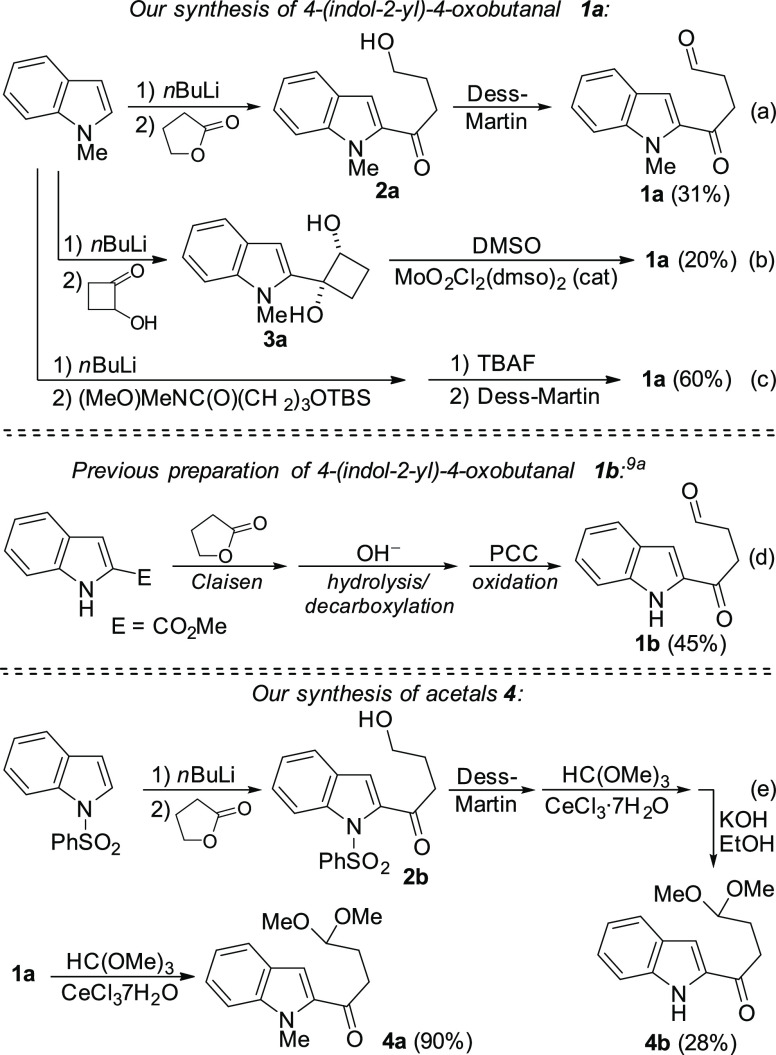
Preparation of 4-(Indol-2-yl)-4-oxobutanal **1a** and Dimethylacetals **4a**,**b**

For the preparation of the required 4-(indol-2-yl)-4-oxobutanal **1a** we envisaged a two-step synthetic route consisting of the reaction of 2-lithio-1-methylindole with γ-butyrolactone,^19^ leading to the ketoalcohol **2a**, and further oxidation of the primary hydroxyl group (Scheme 2a). However, the overall yield was moderate due to the competitive double addition of the organolithium to the lactone. An alternative approach involved the use of 2-hydroxycyclobutanone as an electrophilic reagent for 2-lithio-1-methylindole, giving rise to cyclobutane-1,2-diol derivative **3a**. Subsequent oxidative cleavage with DMSO under dioxomolybdenum catalysis^20^ afforded **1a** but, again, with moderate overall yield (Scheme 2b). In search of a better result, we tested the reaction of the lithiated indole with the Weinreb amide,^21^ which gave access to silyl-protected **2a**, which was further desilylated and oxidized with the Dess-Martin reagent, providing **1a** in a significant overall yield of 60% (Scheme 2c). On the other hand, for the preparation of the previously described **1b**, we initially followed the reported procedure. However, in our hands, the final oxidation delivered a very poor yield of the desired **1b**, which was not improved by using other oxidants, such as Dess-Martin periodinane or Swern reaction, instead of PCC (Scheme 2d). We therefore developed an alternative route involving lithiation of the *N*-sulfonyl protected indole and trapping with γ-butyrolactone to give **2b**. Its oxidation, acetalization,^22^ and further basic hydrolysis yielded the dimethylacetal **4b** with a useful overall yield (Scheme 2e). We also carried out the acetalization of **1a** to give **4a** in high yield.

**Scheme 3:**
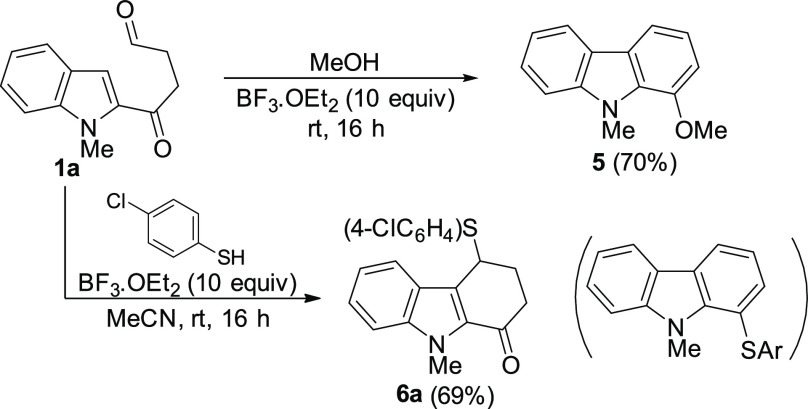
Preliminary Results: Reactions of **1a** with MeOH and 4-ClC_6_H_4_SH

First, **1a** was treated with MeOH under the conditions reported by Moody et al.^9a^ and, as in their results, 1-methoxycarbazole **5** was isolated in high yield (Scheme 3). This product formally arises from the annulation of the indole with the aldehyde and the reaction of the ketone with the external nucleophile. At this point, we thought that softer nucleophiles, rather than *O*-centered ones, might lead to the desired concurrent addition of the indole and the external nucleophile to the same carbonyl group. Gratifyingly, when 4-chlorothiophenol was employed under the same reaction conditions, tetrahydrocarbazolone **6a** was obtained in good yield, instead of the thio-functionalized carbazole, analog to **5** (Scheme 3).

Considering the potential usefulness of this process for the synthesis of functionalized tetrahydrocarbazolone derivatives, we next further evaluated the reaction of indolyl-functionalized γ-ketoaldehyde **1a** with 4-chlorothiophenol, looking for optimal and softer conditions than those employed in Scheme 3 (Table 1). First, BF_3_ could be lowered to catalytic amounts with an even better yield, but with trace amounts of dithioacetal **7a**, derived from a competitive reaction of the thiol with the aldehyde (entries 1 and 2). Other Lewis acids, such as Cu(OTf)_2_, also promoted the reaction, but with more competitive dithioacetal formation (entry 3). Gratifyingly, a simple Brønsted acid, such as *p*-toluenesulfonic acid monohydrate (*p*-TsOH), provided similar results to BF_3_·OEt_2_ (entry 4). However, diphenyl phosphate was less effective in this transformation (entry 5). Due to its easy availability and handling, we selected *p*-TsOH as the catalyst for the subsequent studies. The use of 1 equiv of *p*-TsOH resulted in a higher amount of the competitive dithioacetal **7a** (entry 6). The same tendency was observed when increasing the amount of the thiol (entry 7). We checked that using 10 mol % of *p*-TsOH the reaction was completed in 2 h with an even better yield and with only trace amounts of **7a** (entry 8). Other solvents such as hexafluoroisopropanol (HFIP), MeNO_2_ or toluene provided **6a** with similar efficiency (entries 9–11). Interestingly, the reaction is also completed in HFIP without the acid catalyst, although it requires heating at 60 °C for a longer time (entry 12). In the absence of the thiol, only decomposition was observed in both MeCN and HFIP (entries 13 and 14).

**Table 1:**
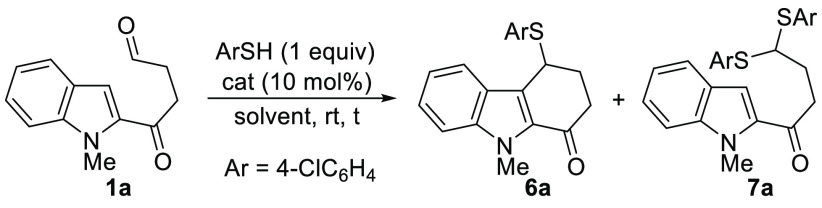
Optimization of the Reaction Conditions for the Synthesis of Tetrahydrocarbazolone **6a**^a^

entry	cat.	solvent	*t* (h)	yield **6a** (%)^b^	yield **7a** (%)^b^
1^c^	BF_3_·OEt_2_	MeCN	16	75	3
2	BF_3_·OEt_2_	MeCN	16	80	4
3	Cu(OTf)_2_	MeCN	16	58	20
4	*p*-TsOH	MeCN	16	79	5
5	(PhO)_2_P(O)OH	MeCN	16	55	8
6^d^	*p*-TsOH	MeCN	16	64	16
7^e^	*p*-TsOH	MeCN	16	66	29
8	*p*-TsOH	MeCN	2	83	5
9	*p*-TsOH	HFIP	2	81	4
10	*p*-TsOH	MeNO_2_	3	78	9
11	*p*-TsOH	toluene	6	76	8
12^f^	–	HFIP	16	80	6
13^g^	*p*-TsOH	MeCN	16	–	–
14^g^	*p*-TsOH	HFIP	16	–	–

a Reaction conditions: **1a** (0.15 mmol), 4-ClC_6_H_4_SH (0.15 mmol, unless otherwise established), catalyst (10 mol %, unless otherwise established), solvent (1 mL), rt, under N_2_ atmosphere.

b Determined by ^1^H NMR using 1,3,5-trimethoxybenzene as internal standard.

c 10 equiv of BF_3_.OEt_2_ were used.

d 1 equiv of *p*-TsOH was used.

e 1.5 equiv of the thiol were employed.

f Carried out at 60 °C. At rt 80% of conversion.

g No thiol was added. Only decomposition products were observed.

After optimizing the reaction conditions (entry 8, Table 1), we evaluated the scope of the reaction for the synthesis of 4-thiotetrahydrocarbazol-1-ones **6** (Table 2). Thiophenols bearing electron-withdrawing substituents led to tetrahydrocarbazolones **6a**–**e** in good yields (entries 1 and 3–6). We also checked that the dimethylacetal **4a**, derived from **1a**, behaves similarly in its reaction with a thiol, although a slightly lower yield was obtained (entry 2). Other arylthiols are useful counterparts (entries 7 and 8), but when a more electron-rich thiophenol such as 3-methoxybenzenethiol was used, the corresponding carbazolone **6h** was obtained in only moderate yield due to the competitive formation of the corresponding dithioacetal **7h** (entry 9). This effect was even more pronounced when an aliphatic thiol was used, leading in this case to a lower yield of the 4-alkylthio carbazolone **6i** (entry 10), indicating that competitive thioacetalization is favored with more nucleophilic thiols. Fortunately, ethyl mercaptoacetate could be engaged in the process leading to the ester-functionalized carbazolone **6j** (entry 11). Finally, to check if *NH* substrates could be employed, the acetal **4b** was reacted with the model thiol to deliver the *NH* carbazolone **6k** in moderate yield (entry 12). Interestingly, as expected from the result shown in Table 1 (entry 9), the reactions proceed with similar efficiency in HFIP.^23^

**Table 2:**
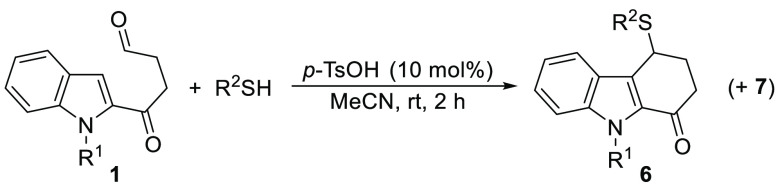
Synthesis of 4-Thiotetrahydrocarbazol-1-ones **6**^a^

entry	starting material	R^1^	R^2^	product	yield (%)^b^
1^c^	**1a**	Me	4-ClC_6_H_4_	**6a**	73
2	**4a**	Me	4-ClC_6_H_4_	**6a**	64
3	**1a**	Me	2-BrC_6_H_4_	**6b**	62
4	**1a**	Me	2-FC_6_H_4_	**6c**	71
5	**1a**	Me	4-CF_3_C_6_H_4_	**6d**	67
6	**1a**	Me	4-NO_2_C_6_H_4_	**6e**	74
7	**1a**	Me	4-MeC_6_H_4_	**6f**	70
8	**1a**	Me	2-Naphthyl	**6g**	64
9	**1a**	Me	3-MeOC_6_H_4_	**6h**	46^c^
10	**1a**	Me	*n*-C_12_H_25_	**6i**	33^d^
11^e^	**1a**	Me	CH_2_CO_2_Et	**6j**	71
12^e^	**4b**	H	4-ClC_6_H_4_	**6k**	34

a Reaction conditions: **1** or **4** (0.4 mmol), R^2^SH (0.4 mmol), *p*-TsOH (0.04 mmol, 10 mol %), solvent (4 mL), rt for 2 h, under N_2_ atmosphere.

b Isolated yield after column chromatography. Only trace amounts of the corresponding dithioacetals **7** were observed in the crude reaction mixture, unless otherwise established.

c Isolated along with 29% of the corresponding dithioacetal **7h**.

d An additional 50% of the dithioacetal **7i** was isolated independently.

e Carried out in HFIP as solvent.

**Scheme 4:**
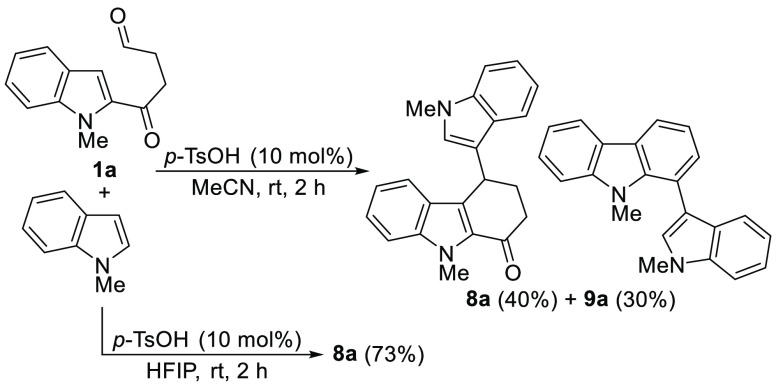
Brønsted Acid Catalyzed Reaction of **1a** with *N*-Methylindole

At this point, we decided to extend the scope of the process by testing other π-nucleophiles. We focused our attention on indoles and started with *N*-methylindole under the optimal conditions described for thiols (Scheme 4). Surprisingly, an almost equimolar mixture of tetrahydrocarbazolone **8a** and carbazole **9a** was obtained, which could be isolated independently. After some experimentation trying to control the selectivity of the reaction,^24^ and considering that HFIP has relevant properties (high polarity, relatively acidic OH) that make it a suitable solvent for the direct nucleophilic substitution of alcohols,^25^ we found that a simple change of solvent from MeCN to HFIP led exclusively to **8a** (Scheme 4). In addition, HFIP is known to increase the acidity of *p*-TsOH through hydrogen bonding interactions.^3f,6d^

**Scheme 5:**
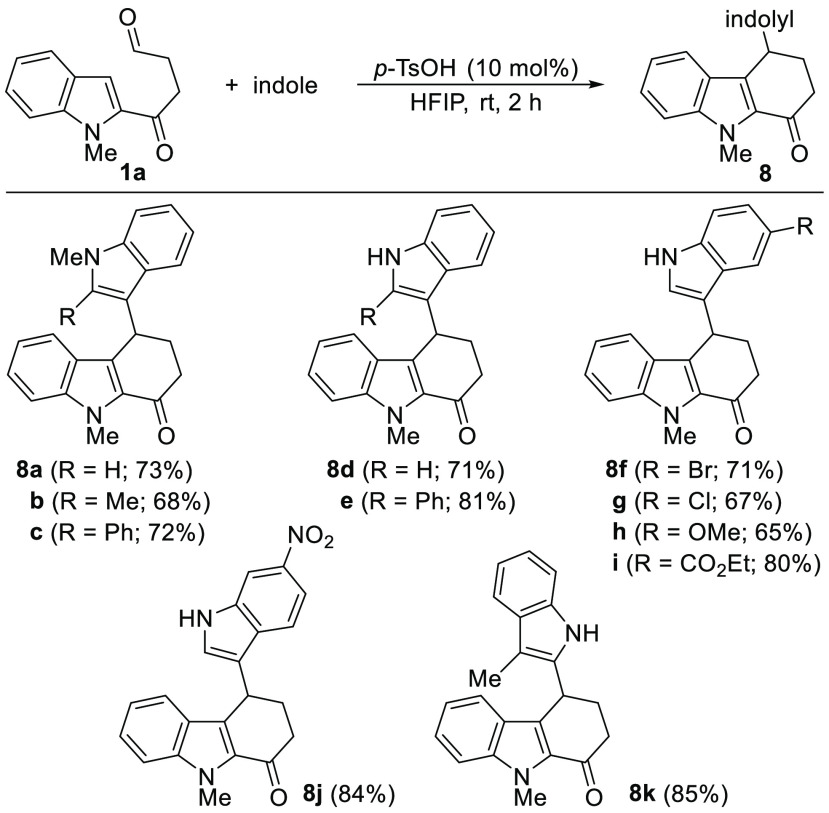
Synthesis of 4-Indolyltetrahydrocarbazol-1-ones **8**

Once the reaction conditions were reoptimized, the scope of the 4-indolyltetrahydrocarbazol-1-ones **8** was evaluated using different indoles as nucleophiles (Scheme 5). *N*-Methylindoles with different substitution at C-2 led to tetrahydrocarbazolones **8a**–**c** in high yields. The structure of **8a** was further confirmed by X-ray analysis.^26^ The use of *NH*-indoles was equally effective and allowed the synthesis of **8d**,**e** which were also obtained in high yields. 5-Substituted indoles with both electron-withdrawing and electron-donating groups could also be employed to give the corresponding indolyl carbazolones **8f**–**i**. Similarly, 6-nitroindole delivered **8j** in high yield. Finally, when skatole was reacted with **1a**, the attack through C-2 led to the carbazolone derivative **8k** also in very high yield (Scheme 5). Surprisingly, when the reaction of the indoles with **1a** was carried out in MeCN, the corresponding 1-indolylcarbazoles **9** were only produced in trace amounts, with the exception of *N*-methylindole and *NH*-indole, which allowed the isolation of **9a** and **9d** in 30% and 10% yield, respectively.^27^ In any case, the yields for the synthesis of **8** were consistently higher in HFIP than in MeCN.

**Scheme 6:**
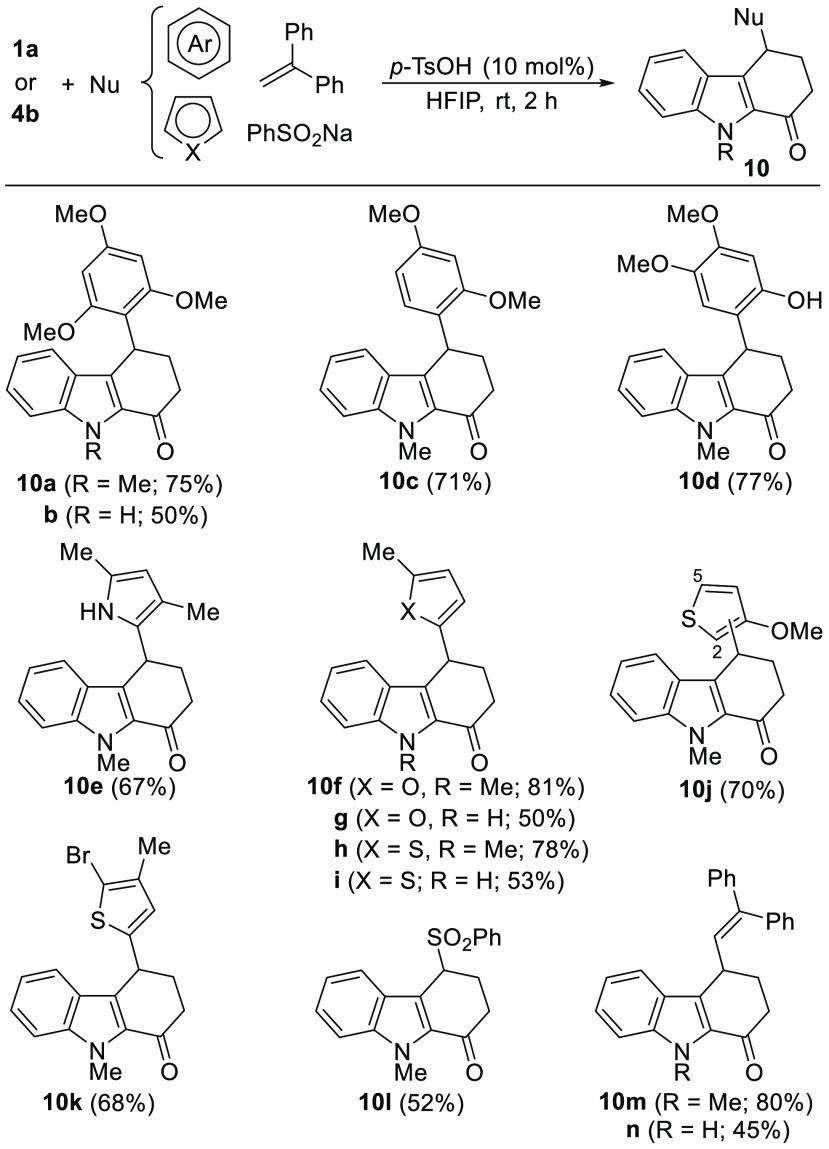
Brønsted Acid-Catalyzed Reaction of **1a** and **4b** with (Hetero)aromatics, 1,1-Diphenylethylene and Sodium Benzenesulfinate

With suitable catalytic conditions for the preparation of 4-indolyltetrahydrocarbazol-1-ones **8**, we turned our attention to evaluating the applicability of this strategy to the synthesis of various 4-(hetero)aryltetrahydrocarbazolones **10** by employing other suitable electron-rich (hetero)aromatics as nucleophiles (Scheme 6). A selection of these functionalized carbazolones **10** were readily prepared by varying the nucleophilic partner with oxoaldehyde **1a** and dimethylacetal **4b**. Methoxy-functionalized benzenes, including 1,3,5-trimethoxybenzene, 1,3-dimethoxybenzene, or 3,4-dimethoxyphenol, reacted regioselectively with **1a** to give 4-aryltetrahydrocarbazolones **10a**–**d**, which were isolated in high yields. Interestingly, **10a** could be prepared on the 2 mmol-scale, allowing the isolation of 534 mg (73% yield) of this substrate. However, other arenes attempted, such as 1,2,3-trimethoxybenzene and 2,6-dimethoxyphenol, only led to decomposition, showing that a delicate balance between the nucleophilicity of the external and the internal nucleophiles is essential for the success of the reaction. Interestingly, electron-rich heteroaromatics, such as selected furans, thiophenes and pyrroles, were able to participate in this process, yielding the 4-heteroaryltetrahydrocarbazolones **10e**–**k** in good yields and complete regioselectivity except in the case of employing 3-methoxythiophene, which afforded **10j** as an approximately 5/1 mixture of regioisomers. Other heteroarenes like *N*-methylpyrrole, benzofuran or benzothiophene were unsuccessful partners.^28^ In addition, sodium benzenesulfinate could also be used as an external nucleophile, giving rise to 4-sulfonyltetrahydrocarbazolone **10l** in moderate yield, as complete conversion could not be achieved. Gratifyingly, 1,1-diphenylethylene efficiently participated in the reaction to give 4-alkenyltetrahydrocarbazolones **10m**,**n** in high yields (Scheme 6). In general, slightly lower yields were obtained for the *NH*-carbazolones derived from **4b**.

**Scheme 7:**
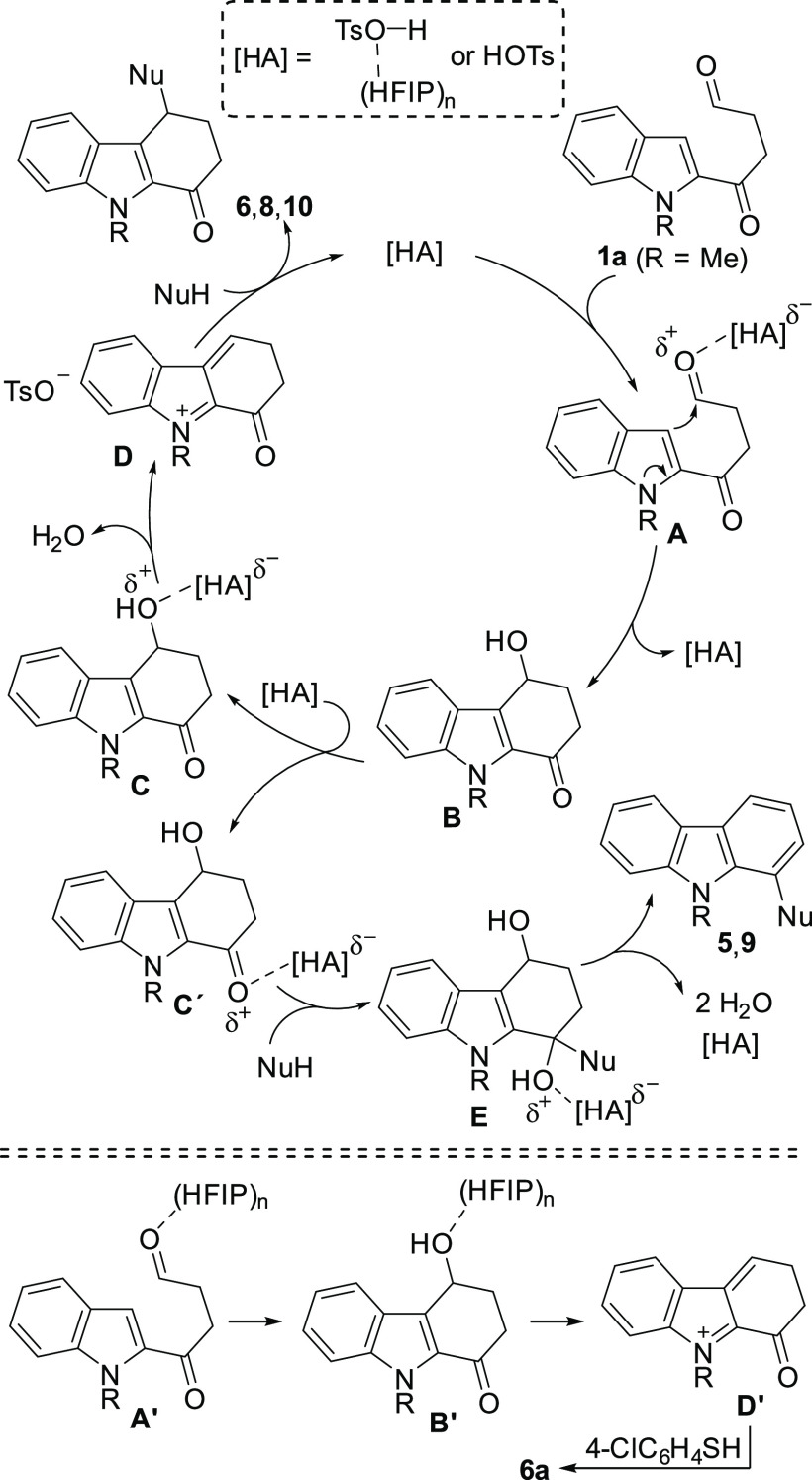
Mechanistic Proposal

Our proposal for the formation of the tetrahydrocarbazolone derivatives **6**, **8** and **10** from 1,4-ketoaldehyde **1a** is outlined in Scheme 7. Although PSTA in MeCN efficiently catalyzes the process, the reaction is enhanced when employing HFIP as the solvent. In this sense, the complexation of the PTSA with HFIP molecules increases its acidity, facilitating the interaction with the solvated ketoaldehyde **1a**. So, initially, the activation of **1a** by the acid catalyst ([HA]) could generate the intermediate **A**. Then, the acid-catalyzed intramolecular attack of the indole to the activated aldehyde would release a solvated 4-hydroxy-2,3,4,9-tetrahydro-1*H*-carbazol-1-one intermediate **B**. Subsequent activation of **B** by the acid catalyst could afford a new intermediate **C**, which would evolve into the cationic indoleneiminium derivative **D** via elimination of water. A final attack by the external nucleophile would yield the functionalized tetrahydrocarbazolones **6**, **8** or **10**, releasing the acid catalyst. A similar pathway could be proposed for the use of acetals **4**. Alternatively, if intermediate **B** undergoes carbonyl protonation leading to **C′**, the subsequent attack of the external nucleophile would produce an intermediate such as **E**, which would eliminate two molecules of water to give the carbazole derivatives **5** and **9** and regenerate the acid catalyst (Scheme 7).^29^ It is worth pointing out that HFIP is not only a stronger acid (p*K*_*a*_ = 9.3) than related alcohol *i*PrOH and an excellent hydrogen bond donor, but also its lower nucleophilicity and strong ionization power make HFIP an ideal medium for generating cations facilitating a variety of synthetic transformations.^30^ In this sense, as mentioned in the optimization (Table 1, entry 12), the HFIP molecules could also activate the 1,4-ketoaldehyde **1a** to obtain **A′**, facilitating an intramolecular attack of the indole to generate **B′**, although with lower efficiency than when PTSA is used. Then, solvated intermediated **B′** could be directly transformed into cationic indoleneiminium **D′** that, after the attack of the thiol nucleophile, released the functionalized tetrahydrocarbazolone **6a**.

**Scheme 8:**
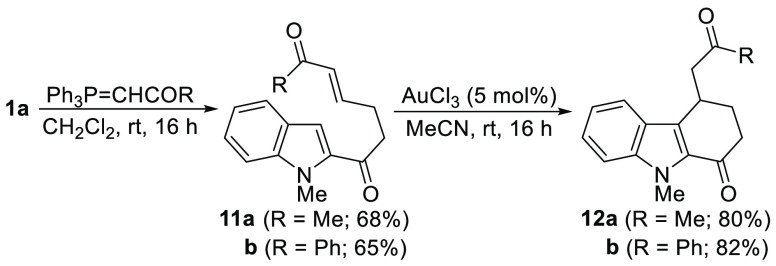
Synthesis of 4-Acylmethyltetrahydrocarbazol-1-ones **12** from **1a**

In addition, taking advantage of γ-ketoaldehyde **1a**, we envisaged that tetrahydrocarbazolones **12** functionalized with an acylmethyl group at C-4 could be accessed in a two-step process (Scheme 8). First, a selective Wittig reaction with selected stabilized ylides provided the corresponding diketones **11**. Then, the intramolecular Michael addition of the indole could be efficiently catalyzed by AuCl_3_^31^ leading to the expected 4-acylmethyltetrahydrocarbazolones **12** in high yields (Scheme 8).

**Scheme 9:**
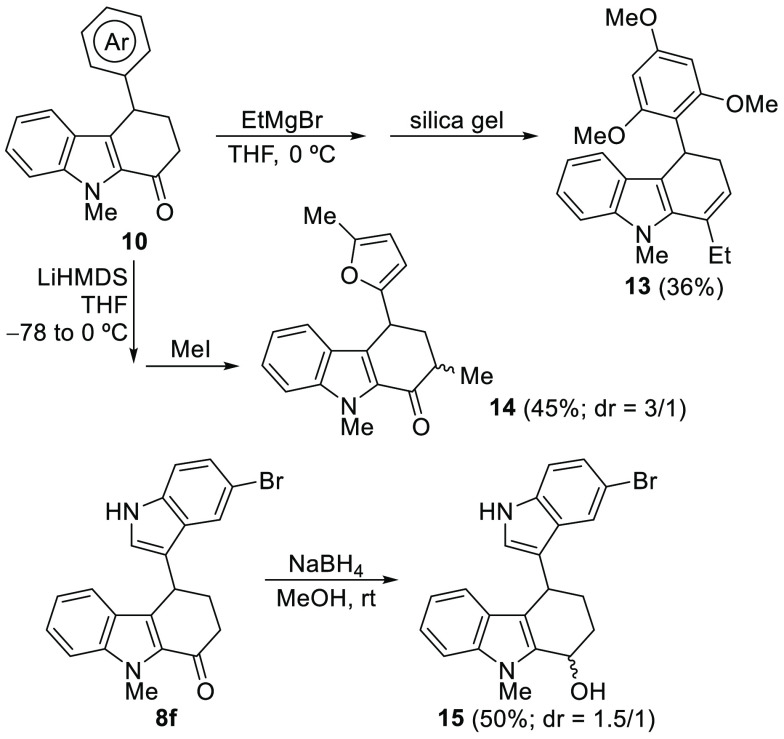
Derivatization of Selected Functionalized Tetrahydrocarbazolones **8** and **10**

At this point, we tried to increase the synthetic value of our protocol for the synthesis of tetrahydrocarbazolones by their further transformation. For example, treatment of **10a** with EtMgBr gave rise to the expected alcohol, which was purified by silica gel chromatography to afford dihydrocarbazole **13** in moderate yield (Scheme 9). Selected carbazolone **10f** was α-alkylated by base-mediated-enolization and subsequent reaction with methyl iodide, giving carbazolone **14** with low diastereoselectivity (Scheme 9). Finally, the reduction of indole-functionalized carbazolone **8f** led to the expected alcohol **15** as a mixture of diastereoisomers (Scheme 9).

## Conclusions

In conclusion, an efficient cascade reaction has been described for the synthesis of 4-functionalized tetahydrocarbazolones involving a tandem intermolecular FC hydroxyalkylation/intermolecular direct nucleophilic substitution of readily accessible 1,4-dicarbonylindole compounds using *p*-TsOH as a cheap, readily available, and easy to handle Brønsted acid catalyst. After the initial attack of the indole to the aldehyde, which enables the formation of the tetrahydrocarbazolone core, the key intermediate bearing the structure of 3-indolylmethanol could be reactivated by the action of the acid catalyst, allowing the subsequent reaction with a wide variety of external nucleophiles such as thiols, (hetero)arenes, alkenes or sulfinates. By fine-tuning the reaction conditions, the competitive dehydration of this crucial intermediate, which leads to the alternative and previously described carbazole formation process, is prevented. Moreover, the tetrahydrocarbazolones obtained are suitable for further derivatization reactions, providing access to a variety of compounds containing the valuable tetrahydrocarbazole core.

## Experimental Section

### General Methods

All reactions involving air-sensitive compounds were carried out under an N_2_ atmosphere in oven-dried glassware. All common reagents and solvents were purchased from commercial suppliers and used without any further purification. TLC was performed on alumina-backed plates coated with silica gel 60 with F_254_ indicator, using UV light or Ce/Mo solution and heat as a visualization agent. Flash silica gel chromatography was performed using Merk silica gel 60, 230–240 mesh. NMR spectra were recorded on a Varian Mercury Plus or Bruker Advanced III HD (300 MHz ^1^H; 75.4 MHz ^13^C, 282 MHz ^19^F) or Bruker Advanced NEO 4500 (500 MHz ^1^H, 126 MHz ^13^C) instrument at room temperature. Chemical shifts (δ) are reported in ppm, using residual solvent peak as the internal reference (CDCl_3_: δ_H_ = 7.26 and δ_C_ = 77.16; (CD_3_)_2_CO: δ_H_ = 2.05 and δ_C_ = 29.84 and 206.26; DMSO-*d*_6_: δ_H_ = 2.50 and δ_C_ = 39.50). Coupling constants (*J*) are given in hertz (Hz). Data are reported as follows: chemical shift, multiplicity (s: singlet, bs: broad single, bm: broad multiplet, d: doublet, dd: doublet of doublets, ddd: doublet of doublets of doublets, dddd: doublet of doublets of doublets of doublets, dq: doublet of quartets, dt: doublet of triplets, ddt: doublet of doublets of triplets, dtd: doublet of triplets of doublets, td: triplet of doublets, t: triplet, tt: triplet of triplets, q: quartet, m: multiplet), coupling constants and integration. Carbon multiplicities have been assigned by DEPT experiments. Low-resolution electron impact mass spectra (EI-LRMS) were obtained at 70 eV, and only the molecular ion and/or base peaks and significant MS peaks are given. High-resolution mass spectra (HRMS) were recorded on an instrument equipped with a QTOF analyzer using ESI (+) or APCI (+). Melting points were measured on a Gallenkamp apparatus using open capillary tubes and were uncorrected. For simplicity, the *p*-toluenesulfonic acid monohydrate is represented as *p*-TsOH.

### Synthesis of 4-Indol-2-yl-4-oxobutanal **1a**: Procedure I-a

To a stirred solution of *N*-methylindole (5.24 g, 40 mmol) in anhydrous Et_2_O (40 mL) was added *n*-BuLi (16 mL, 40 mmol, 2.5 M solution in hexane) at 0 °C, and the resulting mixture was heated at 40 °C for 2 h. Next, γ-butyrolactone (5.16 g, 60 mmol) was added at 0 °C and the resulting mixture was stirred for 2 h at 0 °C. Then, the mixture was quenched with aq. NH_4_Cl (5 mL). THF was removed under reduced pressure, and the aqueous layer was extracted with EtOAc (3 × 15 mL). The combined organic layers were dried over anhydrous Na_2_SO_4_, filtered and concentrated in vacuo. The residue was filtered through a pad of silica to remove the excess of *N*-methylindole using a mixture of hexane/EtOAc (2/1) to afford the alcohol **2a** (3.47 g), which was not isolated in pure form. To a solution of the obtained alcohol **2a** (3.47 g) in DCM (40 mL) was added DMP (22 g, 52 mmol) and the resulting mixture was stirred at rt for 1 h. Then, volatiles were removed under reduced pressure and the residue was purified by flash column chromatography using a 5/1 mixture of hexane/EtOAc as eluent to afford ketoaldehyde **1a** as a brown solid (2.67 g, 31% referred to *N*-methylindole).

#### 4-(1-Methyl-1H-indol-2-yl)-4-oxobutanal (**1a**)

Brown solid: mp 76–78 °C. *R*_*f*_ = 0.34 (hexane/EtOAc, 5/1). ^1^H NMR (300 MHz, CDCl_3_): δ 9.91 (t, *J* = 0.8 Hz, 1H), 7.70 (dt, *J* = 8.1, 1.0 Hz, 1H), 7.43–7.33 (m, 3H), 7.20–7.11 (m, 1H), 4.05 (s, 3H), 3.36 (dd, *J* = 6.8, 6.1 Hz, 2H), 2.91 (td, *J* = 6.5, 0.8 Hz, 2H). ^13^C{^1^H} NMR (75.4 MHz, CDCl_3_): δ 200.8 (CH), 191.7 (C), 140.2 (C), 134.3 (C), 126.2 (CH), 125.9 (C), 123.1 (CH), 120.9 (CH), 111.6 (CH), 110.5 (CH), 37.9 (CH_2_), 32.3 (CH_3_), 32.2 (CH_2_). HRMS (ESI+) *m*/*z*, calcd for C_13_H_14_NO_2_^+^ [M + H]^+^ 216.1019, found 216.1020.

### Synthesis of 4-Indol-2-yl-4-oxobutanal **1a**: Procedure I-b^20^

To a stirred solution of 2-hydroxycyclobutan-1-one (430 mg, 5 mmol) in anhydrous THF (5 mL) was added (1-methyl-1*H*-indol-2-yl)lithium (15 mmol, prepared by treatment of 1-methylindole (1.97 g, 15 mmol) with *n-*BuLi (6 mL, 15 mmol, 2.5 M in hexane) in 10 mL of THF) at −78 °C, and the resulted solution was stirred at rt for 2 h (monitored by TLC). Then, the mixture was quenched with aq. NH_4_Cl (5 mL). THF was removed under reduced pressure and the aqueous layer was extracted with EtOAc (3 × 15 mL). The combined organic layers were dried over anhydrous Na_2_SO_4_, filtered and concentrated in vacuo. Next, in a 35 mL microwave tube, the residue was dissolved in DMSO (8 mL) and MoO_2_Cl_2_(DMSO)_2_ (35 mg, 2 mol %) was added, and the vessel was sealed with a septum. The reaction mixture was stirred at 90 °C for 10 min under microwave irradiation (80 W). After completion of the reaction, the resulting mixture was purified by flash column chromatography using a 5/1 mixture of hexane/EtOAc as eluent to afford ketoaldehyde **1a** as a brown solid (215 mg, 20% referred to 2-hydroxycyclobutan-1-one).

### Synthesis of 4-Indol-2-yl-4-oxobutanal **1a**: Procedure I-c

Synthesis of **S1**:^21^ To a solution of *N*,*O*-dimethylhydroxylamine hydrochloride (5.4 g, 55 mmol) in anhydrous DCM (100 mL) was added dropwise dimethylaluminum chloride (55 mL, 55 mmol, 1 M in hexane) at 0 °C. The resulting mixture was stirred at this temperature for 1 h. γ-Butyrolactone (4.31 g, 50 mmol) was added slowly and the mixture was stirred for 30 h at rt. Then, the reaction was quenched by slow addition of water (50 mL). The aqueous layer was extracted with DCM (3 × 40 mL) and the combined organic layers were dried over anhydrous Na_2_SO_4_, filtered and concentrated in vacuo yielded 4-hydroxy-*N*-methoxy-*N*-methylbutanamide as a yellowish oil which was used in the next step without further purification. Next, to a solution of 4-hydroxy-*N*-methoxy-*N*-methylbutanamide (7.36 g, 50 mmol) and imidazole (10.21 g, 150 mmol) in DMF (50 mL) TBSCl (11.3 g, 75 mmol) was added at 0 °C. The mixture was stirred at rt for 3 h. Then, the reaction was quenched with water and extracted with EtOAc (3 × 30 mL). The combined organic layers were washed with brine (2 × 30 mL), dried over anhydrous Na_2_SO_4_, filtered and concentrated in vacuo. The residue was purified by flash column chromatography using a 5/1 mixture of hexane/EtOAc as eluent affording **S1**.

#### 4-((*tert*-Butyldimethylsilyl)oxy)-*N*-methoxy-*N*-methylbutanamide (**S1**)

Colorless oil (5.49 g, 42% referred to γ-butyrolactone): *R*_*f*_ = 0.3 (hexane/EtOAc, 5/1). ^1^H NMR (300 MHz, CDCl_3_): δ 3.84–3.56 (m, 5H), 3.14 (s, 3H), 2.48 (t, *J* = 7.5 Hz, 2H), 1.97–1.75 (m, 2H), 1.13–0.55 (m, 9H), 0.21–0.34 (m, 6H). ^13^C{^1^H} NMR (75.4 MHz, CDCl_3_): δ 62.4 (CH_2_), 61.2 (CH_3_), 32.3 (CH_3_), 28.3 (CH_2_), 27.7 (CH_2_), 26.0 (3 × CH_3_), 18.4 (C), –5.26 (2 × CH_3_), one quaternary carbon is missing.

##### Synthesis of **1a**

To a solution of *N*-methylindole (1.705 g, 13 mmol) in anhydrous THF (20 mL) was added *n*-BuLi (5.2 mL, 13 mmol, 2.5 M solution in hexane) at 0 °C, and the resulting mixture was stirred at rt for 3 h. Next, a solution of the Weinreb amide **S1** (2.61 g, 10 mmol) in anhydrous THF (4 mL) was added at 0 °C, and the mixture was stirred at rt for 3 h (monitored by GC-MS). Then, the mixture was quenched with aq. NH_4_Cl (5 mL). THF was removed under reduced pressure, and the aqueous layer was extracted with EtOAc (3 × 15 mL). The combined organic layers were dried over anhydrous Na_2_SO_4_, filtered, and concentrated in vacuo. The residue was used in the next step without further purification. To a solution of the obtained crude 4-((*tert*-butyldimethylsilyl)oxy)-1-(1-methyl-1*H*-indol-2-yl)butan-1-one (**S2**) in anhydrous THF (10 mL) was added TBAF (40 mL, 40 mmol, 1 M solution in THF), and the resulting mixture was stirred at rt for 4 h. Then, the reaction was quenched with water, THF was removed under reduced pressure, and the aqueous layer was extracted with EtOAc (3 × 15 mL). The combined organic layers were dried over anhydrous Na_2_SO_4_, filtered and concentrated in vacuo. The residue was dissolved in DCM (10 mL) and DMP (5.51 g, 13 mmol) was added, and the resulting mixture was stirred for 1 h at rt (monitored by TLC). Then volatiles were removed under reduced pressure, and the residue was purified by flash column chromatography using a 5/1 mixture of hexane/EtOAc as eluent affording ketoaldehyde **1a** as a brown solid (1.29 g, 60% referred to the Weinreb amide **S1**).

### Synthesis of 4,4-Dimethoxy-1-(1-methyl-1*H*-indol-2-yl)butan-1-one (**4a**)

To a solution of 4-(1-methyl-1*H*-indol-2-yl)-4-oxobutanal (**1a**) (215 mg, 1 mmol) in MeOH (2 mL) were added CeCl_3_·7H_2_O (323 mg, 1 mmol) and H(COMe)_3_ (848 mg, 8 mmol).^22^ The resulting mixture was stirred at rt for 16 h (monitored by GC-MS). After completion, the reaction was quenched with aq. NaHCO_3_ (2 mL) and extracted with Et_2_O (3 × 5 mL). The combined organic layers were washed with water (15 mL), dried over Na_2_SO_4_, filtered and concentrated in vacuo. The residue was purified by flash column chromatography using a 5/1 mixture of hexane/EtOAc as eluent affording **4a** (235 mg, 90%).

#### 4,4-Dimethoxy-1-(1-methyl-1H-indol-2-yl)butan-1-one (**4a**)

Orange oil: *R*_*f*_ = 0.31 (hexane/EtOAc, 5/1). ^1^H NMR (300 MHz, CDCl_3_): δ 7.69 (d, *J* = 8.0 Hz, 1H), 7.45–7.35 (m, 2H), 7.33 (s, 1H), 7.16 (dd, *J* = 8.0, 3.9 Hz, 1H), 4.49 (t, *J* = 5.6 Hz, 1H), 4.07 (s, 3H), 3.37 (s, 6H), 3.07 (t, *J* = 7.4 Hz, 2H), 2.08 (td, *J* = 7.4, 5.6 Hz, 2H). ^13^C{^1^H} NMR (75.4 MHz, CDCl_3_): δ 193.6 (C), 140.1 (C), 134.8 (C), 125.9 (CH), 123.0 (CH), 120.8 (CH), 111.4 (CH), 110.4 (CH), 104.1 (CH), 53.3 (2 × CH_3_), 34.7 (CH_2_), 32.3 (CH_3_), 27.6 (CH_2_), one quaternary carbon is missing due to overlapping of signals. LRMS (EI): *m*/*z* (%) 261 (M^+^, 60), 158 (100), 89 (77). HRMS (ESI+) *m*/*z*, calcd for C_15_H_19_NNaO_3_^+^ [M + Na]^+^ 284.1257, found 284.1255.

### Synthesis of 1-(1*H*-indol-2-yl)-4,4-dimethoxybutan-1-one **4b**

To a stirred solution of 1-(phenylsulfonyl)-1*H*-indole (5.146 g, 20 mmol) in anhydrous THF (40 mL) was added *n*-BuLi (8.8 mL, 22 mmol, 2.5 M solution in hexane) at −78 °C, and the resulting mixture was stirred at that temperature for 75 min. Next, γ-butyrolactone (3.443 g, 40 mmol) was added at −78 °C and the resulting mixture was stirred for 2 h at 0 °C. Then, the mixture was quenched with aq. NH_4_Cl (5 mL). THF was removed under reduced pressure, and the aqueous layer was extracted with EtOAc (3 × 15 mL). The combined organic layers were dried over anhydrous Na_2_SO_4_, filtered and concentrated in vacuo. The residue was filtered through a pad of silica to remove the excess of 1-(phenylsulfonyl)-1*H*-indole using a 2/1 mixture of hexane/EtOAc to afford alcohol **2b** (2.82 g), which was not isolated in pure form. To a solution of the obtained crude alcohol **2b** in DCM (20 mL) was added DMP (11 g, 26 mmol), and the resulting mixture was stirred at rt for 1 h. Then, volatiles were removed under reduced pressure and the residue was diluted with EtOAc (20 mL) and washed with brine (3 × 20 mL). The organic layer was dried over anhydrous Na_2_SO_4_, filtered and concentrated in vacuo. The residue was dissolved in methanol (20 mL), and CeCl_3_·7H_2_O (6.46 g, 20 mmol) and H(COMe)_3_ (2.86 g, 27 mmol) were added. The solution was stirred at rt for 16 h. Then, the reaction was quenched with aq. NaHCO_3_ (20 mL) and extracted with Et_2_O (3 × 15 mL). The combined organic layers were washed with water (15 mL), dried over Na_2_SO_4_, filtered and concentrated in vacuo. Next, the residue was diluted in EtOH (20 mL) and the solvent was degassed under a current of N_2_ for 10 min. Then, powered KOH (5.61 g, 100 mmol) was added and the solution was heated to reflux (78 °C) for 3 h. After cooling to rt, EtOH was removed under reduced pressure. The concentrated was dissolved in EtOAc (20 mL) and washed with brine (3 × 15 mL). The combined organic layers were dried over anhydrous Na_2_SO_4_, filtered and concentrated in vacuo. The residue was purified by flash column chromatography using a 5/1 mixture of hexane/EtOAc as eluent affording **4b** (1.38 g, 28% referred to *N*-phenylsulfonyl indole).

#### 1-(1H-Indol-2-yl)-4,4-dimethoxybutan-1-one (**4b**)

Purple solid: mp 95–97 °C. *R*_*f*_ = 0.35 (hexane/EtOAc, 5/1). ^1^H NMR (300 MHz, CDCl_3_): δ 9.10 (bs, 1H), 7.71 (d, *J* = 8.1 Hz, 1H), 7.46–7.39 (m, 1H), 7.35 (ddd, *J* = 8.2, 6.9, 1.1 Hz, 1H), 7.24 (dd, *J* = 2.1, 0.9 Hz, 1H), 7.15 (ddd, *J* = 8.2, 6.9, 1.1 Hz, 1H), 4.48 (t, *J* = 5.5 Hz, 1H), 3.36 (s, 6H), 3.05 (t, *J* = 7.5 Hz, 2H), 2.10 (td, *J* = 7.5, 5.5 Hz, 2H). ^13^C{^1^H} NMR (75.4 MHz, CDCl_3_): δ 192.7 (C), 137.4 (C), 135.2 (C), 127.7 (C), 126.4 (CH), 123.2 (CH), 121.1 (CH), 112.3 (CH), 109.4 (CH), 104.0 (CH), 53.4 (2 × CH_3_), 33.2 (CH_2_), 27.6 (CH_2_). LRMS (EI): *m*/*z* (%) 247 (M^+^, 5), 144 (100), 89 (53). HRMS (ESI+) *m*/*z*, calcd for C_14_H_17_NNaO_3_^+^ [M + Na]^+^ 270.1101, found 270.1101.

### Synthesis of Carbazole **5**

To a stirred solution of 4-(1-methyl-1*H*-indol-2-yl)-4-oxobutanal (**1a**) (86 mg, 0.4 mmol) in MeOH (4 mL) was added BF_3_·Et_2_O (1 mL, 4 mmol, 48% solution in Et_2_O), and the resulting mixture was stirred at rt for 16 h. Then, the reaction was quenched with water (2 mL) and extracted with Et_2_O (3 × 5 mL). The combined organic layers were dried over anhydrous Na_2_SO_4_, filtered and concentrated in vacuo. The residue was purified by flash column chromatography using silica gel and a 5/1 mixture of hexane/EtOAc as eluent to afford carbazole **5** (59 mg, 70%).

#### 1-Methoxy-9-methyl-9H-carbazole (**5**)^32^

Brown oil: *R*_*f*_ = 0.34 (hexane/EtOAc, 5/1). ^1^H NMR (300 MHz, CDCl_3_): δ 8.12–8.02 (m, 1H), 7.73 (dd, *J* = 7.8, 0.9 Hz, 1H), 7.49 (ddd, *J* = 8.2, 7.0, 1.2 Hz, 1H), 7.41 (dd, *J* = 8.2, 0.9 Hz, 1H), 7.24 (ddd, *J* = 8.0, 4.3, 1.2 Hz, 1H), 7.14 (t, *J* = 7.8 Hz, 1H), 7.00–6.87 (m, 1H), 4.18 (s, 3H), 4.01 (s, 3H). ^13^C{^1^H} NMR (75.4 MHz, CDCl_3_): δ 147.2 (C), 125.7 (CH), 124.8 (C), 123.0 (C), 120.3 (CH), 119.3 (CH), 118.9 (CH), 113.2 (CH), 108.8 (CH), 107.3 (CH), 55.9 (CH_3_), 32.3 (CH_3_), two quaternary carbons are missing due to overlapping of signals.

### General Procedure II for the Synthesis of Tetrahydrocarbazol-1-ones **6** from **1a**

To a stirred solution of 4-(1-methyl-1*H*-indol-2-yl)-4-oxobutanal (**1a**) (86 mg, 0.4 mmol) in anhydrous MeCN (4 mL) were added the corresponding thiol (0.4 mmol) and *p*-TsOH (7.6 mg), and the resulted solution was stirred at rt for 2 h (monitored by TLC). Then, the resulting mixture was quenched with water (2 mL) and extracted with Et_2_O (3 × 5 mL). The combined organic layers were dried over anhydrous Na_2_SO_4_, filtered and concentrated in vacuo. The residue was purified by flash column chromatography using silica gel and mixtures of hexane/EtOAc as eluent to afford the corresponding tetrahydrocarbazolones **6a**–**j**.

#### 4-((4-Chlorophenyl)thio)-9-methyl-2,3,4,9-tetrahydro-1H-carbazol-1-one (**6a**)

General procedure II was followed using 4-chlorobenzenethiol (58 mg, 0.4 mmol) obtaining **6a**, which was isolated by flash column chromatography (hexane/EtOAc, 5/1) as an orange solid (100 mg, 73%): mp 143–145 °C. *R*_*f*_ = 0.34 (hexane/EtOAc, 5/1). ^1^H NMR (300 MHz, CDCl_3_): δ 7.76 (dt, *J* = 8.0, 0.9 Hz, 1H), 7.46 (d, *J* = 8.4 Hz, 2H), 7.42 (d, *J* = 7.2 Hz, 1H), 7.37 (d, *J* = 8.5 Hz, 1H), 7.31 (d, *J* = 8.4 Hz, 2H), 7.19 (ddd, *J* = 8.0, 6.6, 1.3 Hz, 1H), 4.94 (t, *J* = 3.4 Hz, 1H), 4.09 (s, 3H), 3.21 (ddd, *J* = 17.7, 13.0, 4.7 Hz, 1H), 2.57–2.40 (m, 2H), 2.34–2.24 (m, 1H). ^13^C{^1^H} NMR (75.4 MHz, CDCl_3_): δ 191.4 (C), 139.7 (C), 134.4 (2 × CH), 134.1 (C), 133.8 (C), 130.4 (C), 129.4 (2 × CH), 127.0 (CH), 125.7 (C), 124.0 (C), 121.6 (CH), 121.0 (CH), 110.6 (CH), 42.5 (CH), 35.6 (CH_2_), 31.8 (CH_3_), 29.9 (CH_2_). LRMS (EI): *m*/*z* (%) 341 (M^+^,5), 198 (100), 170 (43). HRMS (ESI+) *m*/*z*, calcd for C_19_H_17_ClNOS^+^ [M + H]^+^ 342.0714, found 342.0706.

#### 4-((2-Bromophenyl)thio)-9-methyl-2,3,4,9-tetrahydro-1H-carbazol-1-one (**6b**)

General procedure II was followed using 2-bromobenzenethiol (99 mg, 0.4 mmol) obtaining **6b**, which was isolated by flash column chromatography (hexane/EtOAc, 5/1) as an orange solid (87 mg, 62%): mp 123–125 °C. *R*_*f*_ = 0.32 (hexane/EtOAc, 5/1). ^1^H NMR (300 MHz, CDCl_3_): δ 7.86 (dt, *J* = 8.1, 1.0 Hz, 1H), 7.64 (ddd, *J* = 13.8, 7.7, 1.5 Hz, 2H), 7.47–7.34 (m, 2H), 7.31 (td, *J* = 7.7, 1.5 Hz, 1H), 7.23–7.14 (m, 2H), 5.16 (dd, *J* = 4.0, 2.5 Hz, 1H), 4.09 (s, 3H), 3.33 (m, 1H), 2.72–2.36 (m, 2H), 2.36–2.16 (m, 1H). ^13^C{^1^H} NMR (75.4 MHz, CDCl_3_): δ 191.5 (C), 139.7 (C), 136.2 (C), 134.3 (CH), 133.6 (CH), 130.6 (C), 129.1 (CH), 128.14 (C), 128.09 (CH), 126.9 (CH), 125.3 (C), 124.2 (C), 121.8 (CH), 121.0 (CH), 110.5 (CH), 41.0 (CH), 35.7 (CH_2_), 31.8 (CH_3_), 29.3 (CH_2_). LRMS (EI): *m*/*z* (%) 386 (M^+^, 60), 213 (100), 181 (33). HRMS (ESI+) *m*/*z*, calcd for C_19_H_17_BrNOS^+^ [M + H]^+^ 386.0209, found 386.0208.

#### 4-((2-Fluorophenyl)thio)-9-methyl-2,3,4,9-tetrahydro-1H-carbazol-1-one (**6c**)

General procedure II was followed using 2-fluorobenzenethiol (51 mg, 0.4 mmol) obtaining **6c**, which was isolated by flash column chromatography (hexane/EtOAc, 5/1) as an orange solid (92 mg, 71%): mp 96–98 °C. *R*_*f*_ = 0.27 (hexane/EtOAc, 5/1). ^1^H NMR (300 MHz, CDCl_3_): δ 7.86 (dt, *J* = 8.2, 1.1 Hz, 1H), 7.56 (td, *J* = 7.5, 1.7 Hz, 1H), 7.46–7.30 (m, 3H), 7.28–7.08 (m, 3H), 5.20–5.00 (m, 1H), 4.09 (s, 3H), 3.53–3.21 (m, 1H), 2.61–2.39 (m, 2H), 2.24–2.16 (m, 1H). ^13^C{^1^H} NMR (75.4 MHz, CDCl_3_): δ 191.5 (C), 163.1 (d, ^1^*J*_C–F_ = 246.3 Hz, C), 139.7 (C), 136.2 (CH), 130.5 (d, ^3^*J*_C–F_ = 8.0 Hz, CH), 130.3 (C), 126.9 (CH), 125.8 (C), 124.7 (d, ^3^*J*_C–F_ = 3.8 Hz, CH), 124.2 (C), 121.9 (d, ^2^*J*_C–F_ = 19.0 Hz, C), 121.8 (CH), 121.0 (CH), 116.2 (d, ^2^*J*_C–F_ = 23.2 Hz, CH), 110.5 (CH), 41.4 (d, ^4^*J*_C–F_ = 2.9 Hz, CH), 35.4 (CH_2_), 31.7 (CH_3_), 30.0 (CH_2_). ^19^F NMR (282 MHz, CDCl_3_): δ (ppm) = −107.02 to −107.10 (m, 1F). LRMS (EI): *m*/*z* (%) could not be recorded. HRMS (ESI+) *m*/*z*, calcd for C_19_H_17_FNOS^+^ [M + H]^+^ 327.1041, found 327.1045.

#### 9-Methyl-4-((4-(trifluoromethyl)phenyl)thio)-2,3,4,9-tetrahydro-1H-carbazol-1-one (**6d**)

General procedure II was followed using 4-(trifluoromethyl)benzenethiol (71 mg, 0.4 mmol) obtaining **6d**, which was isolated by flash column chromatography (hexane/EtOAc, 5/1) as a brown solid (101 mg, 67%): mp 155–157 °C. *R*_*f*_ = 0.31 (hexane/EtOAc, 5/1). ^1^H NMR (300 MHz, CDCl_3_): δ 7.76 (d, *J* = 8.1 Hz, 1H), 7.59 (s, 4H), 7.50–7.35 (m, 2H), 7.29–7.14 (m, 1H), 5.13 (t, *J* = 3.5 Hz, 1H), 4.10 (s, 3H), 3.33–3.14 (m, 1H), 2.64–2.50 (m, 2H), 2.41–2.30 (m, 1H). ^13^C{^1^H} NMR (126 MHz, CDCl_3_): δ 191.2 (C), 141.0 (C), 139.7 (C), 130.9 (2 × CH), 130.6 (C), 129.1 (q, ^2^*J*_C–F_ = 32.8 Hz, C), 127.1 (CH), 126.0 (q, ^3^*J*_C–F_ = 3.8 Hz, 2 × CH), 124.9 (C), 124.1 (q, ^1^*J*_C–F_ = 272.0 Hz, C), 123.9 (C), 121.5 (CH), 121.1 (CH), 110.7 (CH), 41.1 (CH), 35.6 (CH_2_), 31.8 (CH_3_), 29.8 (CH_2_). ^19^F NMR (282 MHz, CDCl_3_): δ (ppm) = −62.48. LRMS (EI): *m*/*z* (%) could not be recorded. HRMS (ESI+) *m*/*z*, calcd for C_20_H_17_F_3_NOS^+^ [M + H]^+^ 377.1009, found 377.1012.

#### 9-Methyl-4-((4-nitrophenyl)thio)-2,3,4,9-tetrahydro-1H-carbazol-1-one (**6e**)

General procedure II was followed using 4-nitrobenzenethiol (62 mg, 0.4 mmol) obtaining **6e**, which was isolated by flash column chromatography (hexane/EtOAc, 5/1) as a yellow solid (104 mg, 74%): mp 178–180 °C. *R*_*f*_ = 0.29 (hexane/EtOAc, 5/1). ^1^H NMR (300 MHz, CDCl_3_): δ 8.24–8.12 (m, 2H), 7.75 (dt, *J* = 8.1, 1.0 Hz, 1H), 7.63–7.52 (m, 2H), 7.48–7.35 (m, 2H), 7.20 (ddd, *J* = 8.1, 6.4, 1.5 Hz, 1H), 5.26 (t, *J* = 3.3 Hz, 1H), 4.10 (s, 3H), 3.30–3.06 (m, 1H), 2.80–2.50 (m, 2H), 2.48–2.34 (m, 1H). ^13^C{^1^H} NMR (75.4 MHz, CDCl_3_): δ 191.0 (C), 146.4 (C), 139.7 (C), 130.7 (C), 128.8 (2 × CH), 127.2 (CH), 124.3 (2 × CH), 123.9 (C), 123.8 (CH), 121.3 (CH), 110.8 (CH), 40.3 (CH), 35.6 (CH_3_), 31.8 (CH_2_), 29.7 (CH_2_), two quaternary carbons are missing due to overlapping of signals. LRMS (EI): *m*/*z* (%) could not be recorded. HRMS (ESI+) *m*/*z*, calcd for C_19_H_17_N_2_O_3_S^+^ [M + H]^+^ 353.0954, found 353.0956.

#### 9-Methyl-4-(p-tolylthio)-2,3,4,9-tetrahydro-1H-carbazol-1-one (**6f**)

General procedure II was followed using 4-methylbenzenethiol (50 mg, 0.4 mmol) obtaining **6f**, which was isolated by flash column chromatography (hexane/EtOAc, 5/1) as a brown solid (90 mg, 70%): mp 108–110 °C. *R*_*f*_ = 0.33 (hexane/EtOAc, 5/1). ^1^H NMR (300 MHz, CDCl_3_): δ 7.81 (dt, *J* = 8.1, 1.0 Hz, 1H), 7.46 (d, *J* = 8.1 Hz, 2H), 7.43–7.34 (m, 2H), 7.24–7.12 (m, 3H), 4.89 (t, *J* = 3.3 Hz, 1H), 4.09 (s, 3H), 3.36–3.17 (m, 1H), 2.58–2.40 (m, 2H), 2.37 (s, 3H), 2.35–2.24 (m, 1H). ^13^C{^1^H} NMR (75.4 MHz, CDCl_3_): δ 191.7 (C), 139.7 (C), 138.2 (C), 133.7 (2 × CH), 131.6 (C), 130.3 (C), 130.0 (2 × CH), 126.9 (C), 126.4 (C), 124.1 (CH), 121.8 (CH), 120.8 (CH), 110.5 (CH), 42.6 (CH), 35.6 (CH_3_), 31.7 (CH_2_), 30.0 (CH_2_), 21.3 (CH_3_). LRMS (EI): *m*/*z* (%) 320 (M^+^, 2), 198 (100), 170 (28). HRMS (ESI+) *m*/*z*, calcd for C_20_H_20_NOS^+^ [M + H]^+^ 322.1260, found 322.1260.

#### 9-Methyl-4-(naphthalen-2-ylthio)-2,3,4,9-tetrahydro-1H-carbazol-1-one (**6g**)

General procedure II was followed using naphthalene-2-thiol (64 mg, 0.4 mmol) obtaining **6g**, which was isolated by flash column chromatography (hexane/EtOAc, 5/1) as a brown solid (91.5 mg, 64%): mp 141–143 °C. *R*_*f*_ = 0.28 (hexane/EtOAc, 5/1). ^1^H NMR (300 MHz, CDCl_3_): δ 8.74 (dt, *J* = 8.4, 1.0 Hz, 1H), 8.01–7.76 (m, 4H), 7.66 (ddd, *J* = 8.4, 6.8, 1.4 Hz, 1H), 7.57 (ddd, *J* = 8.2, 6.8, 1.4 Hz, 1H), 7.50–7.35 (m, 3H), 7.29–7.08 (m, 1H), 5.01 (dd, *J* = 4.0, 2.7 Hz, 1H), 4.11 (s, 3H), 3.37 (ddd, *J* = 17.0, 13.0, 4.0 Hz, 1H), 2.53 (dt, *J* = 17.0, 3.6 Hz, 1H), 2.43–2.32 (m, 1H), 2.28–2.17 (m, 1H). ^13^C{^1^H} NMR (75.4 MHz, CDCl_3_): δ 191.7 (C), 139.7 (C), 134.7 (C), 134.3 (C), 133.5 (CH), 132.4 (C), 130.4 (C), 129.3 (CH), 128.9 (CH), 127.0 (CH), 126.9 (CH), 126.5 (CH), 126.4 (C), 125.8 (CH), 125.7 (CH), 124.2 (C), 121.7 (CH), 120.9 (CH), 110.6 (CH), 42.2 (CH), 35.7 (CH_2_), 31.8 (CH_3_), 29.9 (CH_2_). LRMS (EI): *m*/*z* (%) could not be recorded. HRMS (ESI+) *m*/*z*, calcd for C_23_H_20_NOS^+^ [M + H]^+^ 359.1292, found 359.1293.

#### 4-((3-Methoxyphenyl)thio)-9-methyl-2,3,4,9-tetrahydro-1H-carbazol-1-one (**6h**)

General procedure II was followed using 3-methoxybenzenethiol (56 mg, 0.4 mmol) obtaining a 1.2/1 mixture of the corresponding product **6h** and the thioacetal **7h**. Isolated as a c.a. 2.5/1 mixture of **6h**/**7h** (117 mg) (46% **6h**; 29% **7h**): Brown oil. *R*_*f*_ = 0.32 (hexane/EtOAc, 5/1). ^1^H NMR (300 MHz, CDCl_3_): δ 7.80 (dt, *J* = 8.2, 1.0 Hz, 1H, **6h**), 7.69 (dt, *J* = 8.1, 1.1 Hz, 2H, **7h**), 7.50–7.04 (m, 6H, **6h** + 12H, **7h**), 6.88–6.79 (m, 1H, **6h** + 2H, **7h**), 5.01 (dd, *J* = 4.1, 2.8 Hz, 1H, **6h**), 4.80–4.60 (m, 1H, **7h**), 4.09 (s, 3H, **6h**), 4.05 (s, 3H, **7h**), 3.80 (s, 3H, **6h**), 3.77 (s, 3H, **7h**), 3.41–3.13 (m, 1H, **6h**+**7h**), 2.66–2.28 (m, 3H, **6h**+**7h**). ^13^C{^1^H} NMR (75.4 MHz, CDCl_3_): δ 193.0 (C), 191.6 (C), 160.0 (2 × C), 159.8 (C), 140.2 (C), 139.7 (C), 136.6 (C), 135.3 (2 × C), 130.4 (C), 130.0 (CH), 129.8 (CH), 126.9 (CH), 126.0 (CH), 124.7 (CH), 124.5 (4 × CH), 124.1 (CH), 123.0 (4 × CH), 121.7 (CH), 120.9 (CH), 120.8 (CH), 117.9 (CH), 117.5 (CH), 113.7 (CH), 113.3 (CH), 111.6 (CH), 110.5 (CH), 110.4 (CH), 57.3 (CH), 55.4 (CH_3_), 55.3 (2 × CH_3_), 41.8 (CH), 37.0 (CH_2_), 35.6 (CH_2_), 32.2 (CH_3_), 31.7 (CH_3_), 31.0 (CH_2_), 30.0 (CH_2_). LRMS (EI): *m*/*z* (%) could not be recorded. HRMS for **6h** (APCI+) *m*/*z*, calcd for C_20_H_20_NO_2_S^+^ [M + H]^+^ 338.1209, found 338.1216.

#### 4-(Dodecylthio)-9-methyl-2,3,4,9-tetrahydro-1H-carbazol-1-one (**6i**)

General procedure II was followed using dodecanethiol (81 mg, 0.4 mmol) obtaining **6i**, which was isolated by flash column chromatography (hexane/EtOAc, 7/1) as a brown solid (53 mg, 33%): mp 72–74 °C. *R*_*f*_ = 0.22 (hexane/EtOAc, 7/1). ^1^H NMR (300 MHz, CDCl_3_): δ 7.84 (dt, *J* = 8.1, 1.0 Hz, 1H), 7.49–7.30 (m, 2H), 7.19 (ddd, *J* = 8.0, 6.6, 1.3 Hz, 1H), 4.59 (t, *J* = 3.7 Hz, 1H), 4.07 (s, 3H), 3.19 (ddd, *J* = 17.5, 13.2, 4.5 Hz, 1H), 2.81–2.46 (m, 4H), 2.46–2.22 (m, 1H), 1.79–1.57 (m, 2H), 1.42 (bs, 2H), 1.27 (s, 16H), 0.91 (s, 3H). ^13^C{^1^H} NMR (75.4 MHz, CDCl_3_): δ 191.8 (C), 139.8 (C), 130.2 (C), 127.6 (C), 126.9 (CH), 124.2 (C), 121.8 (CH), 120.7 (CH), 110.5 (CH), 37.8 (CH), 35.9 (CH_2_), 32.3 (CH_2_), 32.1 (CH_2_), 31.7 (CH_3_), 30.5 (CH_2_), 30.0 (CH_2_), 29.79 (CH_2_), 29.78 (CH_2_), 29.75 (CH_2_), 29.68 (CH_2_), 29.5 (CH_2_), 29.4 (CH_2_), 29.2 (CH_2_), 22.8 (CH_2_), 14.3 (CH_3_). LRMS (EI): *m*/*z* (%) 399 (M^+^, 2), 198 (100), 55 (75). HRMS (ESI+) *m*/*z*, calcd for C_25_H_38_NOS^+^ [M + H]^+^ 401.2701, found 401.2703.

#### 4,4-Bis(dodecylthio)-1-(1-methyl-1H-indol-2-yl)butan-1-one (**7i**)

General procedure II was followed using dodecanethiol (81 mg, 0.4 mmol) obtaining **7i**, which was isolated by flash column chromatography (hexane/EtOAc, 7/1) as a colorless oil (120 mg, 50%): *R*_*f*_ = 0.42 (hexane/EtOAc, 7/1). ^1^H NMR (300 MHz, CDCl_3_): δ 7.69 (dt, *J* = 8.1, 0.9 Hz, 1H), 7.37 (dd, *J* = 4.7, 1.3 Hz, 3H), 7.16 (dd, *J* = 8.0, 4.0 Hz, 1H), 4.07 (s, 3H), 3.88 (t, *J* = 6.9 Hz, 1H), 3.26 (t, *J* = 7.2 Hz, 2H), 2.73–2.57 (m, 4H), 2.57–2.45 (m, 4H), 2.36–2.06 (m, 2H), 1.70–1.51 (m, 7H), 1.35–1.26 (m, 23H), 1.02–0.80 (m, 12H). ^13^C{^1^H} NMR (75.4 MHz, CDCl_3_): δ 193.0 (C), 140.2 (C), 134.8 (C), 126.0 (CH), 125.9 (C), 123.0 (CH), 111.5 (CH), 110.4 (CH), 51.4 (CH), 37.3 (CH_2_), 32.3 (CH_3_), 32.0 (CH_2_), 30.9 (CH_2_), 30.5 (CH_2_), 29.8 (CH_2_), 29.75 (CH_2_), 29.72 (CH_2_), 29.67 (CH_2_), 29.65 (CH_2_), 29.55 (CH_2_), 29.5 (CH_2_), 29.4 (CH_2_), 29.2 (CH_2_), 29.18 (CH_2_), 28.5 (CH_2_), 24.8 (CH_2_), 22.8 (CH_2_), 14.2 (2 × CH_3_), seven CH_2_ are missing due to overlapping of signals. LRMS (EI): *m*/*z* (%) could not be recorded. HRMS (ESI+) *m*/*z*, calcd for C_37_H_63_NNaOS_2_^+^ [M + Na]^+^ 624.4275, found 624.4277.

#### Ethyl 2-((9-methyl-1-oxo-2,3,4,9-tetrahydro-1H-carbazol-4-yl)thio)acetate (**6j**)

General procedure II was followed using ethyl 2-mercaptoacetate (48 mg, 0.4 mmol) obtaining **6j**, which was isolated by flash column chromatography (hexane/EtOAc, 5/1) as a white solid (90 mg, 71%): mp 105–107 °C. *R*_*f*_ = 0.33 (hexane/EtOAc, 5/1). ^1^H NMR (300 MHz, CDCl_3_): δ 7.89 (dt, *J* = 8.2, 1.1 Hz, 1H), 7.46–7.35 (m, 2H), 7.22 (ddd, *J* = 8.0, 6.6, 1.1 Hz, 1H), 4.87 (t, *J* = 3.5 Hz, 1H), 4.28 (q, *J* = 7.1 Hz, 2H), 4.08 (s, 3H), 3.45–3.29 (m, 2H), 3.26–3.14 (m, 1H), 2.68–2.52 (m, 2H), 2.50–2.34 (m, 1H), 1.36 (t, *J* = 7.1 Hz, 3H). ^13^C{^1^H} NMR (75.4 MHz, CDCl_3_): δ 191.4 (C), 170.7 (C), 139.6 (C), 130.2 (C), 126.9 (CH), 126.1 (C), 124.0 (C), 121.7 (CH), 120.9 (CH), 110.4 (CH), 61.6 (CH_2_), 38.3 (CH), 35.6 (CH_2_), 33.7 (CH_2_), 31.6 (CH_3_), 29.7 (CH_2_), 14.3 (CH_3_). LRMS (EI): *m*/*z* (%) 317 (M^+^, 5), 198 (100), 170 (34). HRMS (ESI+) *m*/*z*, calcd for C_17_H_20_NO_3_S^+^ [M + H]^+^ 318.1158, found 318.1161.

### Synthesis of Tetrahydrocarbazol-1-one **6a** from Dimethylacetal **4a**

To a stirred solution of 4,4-dimethoxy-1-(1-methyl-1*H*-indol-2-yl)butan-1-one (**4a**) (104 mg, 0.4 mmol) in anhydrous HFIP (4 mL) were added 4-chlorobenzenethiol (58 mg, 0.4 mmol) and *p*-TsOH (7.6 mg), and the resulted solution was stirred at rt for 2 h (monitored by TLC). Then, the resulting mixture was quenched with water (2 mL) and extracted with Et_2_O (3 × 5 mL). The combined organic layers were dried over anhydrous Na_2_SO_4_, filtered and concentrated in vacuo. The residue was purified by flash column chromatography using silica gel and a 5/1 mixture of hexane/EtOAc as eluent to afford **6a** as an orange solid (87.5 mg, 64%). Their characterization data have been reported above.

### Synthesis of Tetrahydrocarbazol-1-ones **6k** from **4b**

To a stirred solution of 1-(1*H*-indol-2-yl)-4,4-dimethoxybutan-1-one (**4b**) (99 mg, 0.3 mmol) in anhydrous HFIP (3 mL) were added 4-chlorobenzenthiol (99 mg, 0.3 mmol) and *p*-TsOH (5.7 mg), and the resulted solution was stirred at rt for 2 h (monitored by TLC). Then, the resulting mixture was quenched with water (2 mL) and extracted with ether (3 × 5 mL). The combined organic layers were dried over anhydrous Na_2_SO_4_, filtered and concentrated in vacuo. The residue was purified by flash column chromatography using silica gel and a 3/1 mixture of hexane/EtOAc as eluent to afford **6k** as a brown solid (33 mg, 34%).

#### 4-((4-Chlorophenyl)thio)-2,3,4,9-tetrahydro-1H-carbazol-1-one (**6k**)

Mp 183–185 °C. *R*_*f*_ = 0.28 (hexane/EtOAc, 3/1). ^1^H NMR (300 MHz, CDCl_3_): δ 9.39 (bs, 1H), 7.82–7.64 (m, 1H), 7.50–7.36 (m, 4H), 7.33–7.25 (m, 2H), 7.22–7.16 (m, 1H), 4.91 (t, *J* = 3.7 Hz, 1H), 3.23–3.10 (m, 1H), 2.74–2.44 (m, 2H), 2.44–2.30 (m, 1H). ^13^C{^1^H} NMR (75.4 MHz, CDCl_3_): δ 190.6 (C), 137.9 (C), 134.6 (2 × CH), 134.2 (C), 133.5 (C), 131.4 (C), 129.5 (2 × CH), 127.4 (CH), 126.3 (C), 125.2 (C), 121.8 (CH), 121.3 (CH), 112.9 (CH), 42.1 (CH), 34.2 (CH_2_), 30.5 (CH_2_). LRMS (EI): *m*/*z* (%) could not be recorded. HRMS (ESI+) *m*/*z*, calcd for C_18_H_15_ClNOS^+^ [M + H]^+^ 328.0557, found 328.0560.

### General Procedure III for the Synthesis of Tetrahydrocarbazol-1-ones **8** from **1a**

To a stirred solution of 4-(1-methyl-1*H*-indol-2-yl)-4-oxobutanal (**1a**) (86 mg, 0.4 mmol) in anhydrous HFIP (4 mL) were added the corresponding indole (0.4 mmol) and *p*-TsOH (7.6 mg, 10 mol %), and the resulted solution was stirred at rt for 2 h (monitored by TLC). Then, the resulting mixture was quenched with water (2 mL) and extracted with Et_2_O (3 × 5 mL). The combined organic layers were dried over anhydrous Na_2_SO_4_, filtered and concentrated in vacuo. The residue was purified by flash column chromatography using silica gel and mixtures of hexane/EtOAc as eluent to afford the corresponding tetrahydrocarbazolones **8a**–**k**. When the reaction of **1a** with *N*-methylindole was carried out in MeCN, carbazole **9a** was also obtained (30% yield). When the same reaction was carried out with 1.5 equiv of *N*-methylindole, **S3** was also isolated (31% yield).

#### 9-Methyl-4-(1-methyl-1H-indol-3-yl)-2,3,4,9-tetrahydro-1H-carbazol-1-one (**8a**)

General procedure III was followed using *N*-methylindole (53 mg, 0.4 mmol) obtaining **8a**, which was isolated by flash column chromatography (hexane/EtOAc, 5/1) as a brown solid (97 mg, 73%): mp 183–185 °C. *R*_*f*_ = 0.27 (hexane/EtOAc, 5/1). ^1^H NMR (300 MHz, CDCl_3_): δ 7.73 (d, *J* = 7.9 Hz, 1H), 7.45–7.23 (m, 5H), 7.24–7.14 (m, 1H), 7.01 (ddd, *J* = 8.1, 5.5, 2.4 Hz, 1H), 6.48 (s, 1H), 4.97–4.91 (m, 1H), 4.20 (s, 3H), 3.66 (s, 3H), 2.85–2.71 (m, 1H), 2.67–2.47 (m, 3H). ^13^C{^1^H} NMR (75.4 MHz, CDCl_3_): δ 192.9 (C), 140.0 (C), 137.5 (C), 130.9 (C), 130.5 (C), 127.7 (CH), 127.2 (C), 126.6 (CH), 124.6 (C), 122.4 (CH), 121.8 (CH), 120.1 (CH), 119.1 (CH), 119.0 (CH), 115.7 (C), 110.3 (CH), 109.5 (CH), 37.0 (CH_2_), 32.7 (CH), 32.0 (CH_2_), 31.8 (CH_3_), 30.5 (CH_3_). LRMS (EI): *m*/*z* (%) 328 (M^+^, 100), 299 (65), 271 (48). HRMS (ESI+) *m*/*z*, calcd for C_22_H_21_N_2_O [M + H]^+^ 329.1648, found 329.1650.

#### 4-(1,2-Dimethyl-1H-indol-3-yl)-9-methyl-2,3,4,9-tetrahydro-1H-carbazol-1-one (**8b**)

General procedure III was followed using 1,2-dimethylindole (58 mg, 0.4 mmol) obtaining **8b**, which was isolated by flash column chromatography (hexane/EtOAc, 5/1) as a pink solid (93 mg, 68%): mp 167–169 °C. *R*_*f*_ = 0.27 (hexane/EtOAc, 5/1). ^1^H NMR (300 MHz, CDCl_3_): δ 7.45–6.57 (bm, 8H), 4.78 (bs, 1H), 4.17 (s, 3H), 3.70 (bs, 3H), 3.04–2.21 (bm, 7H), some signals broaden due to restricted bond rotation. ^13^C{^1^H} NMR (126 MHz, CDCl_3_): δ 192.6 (C), 140.0 (C), 136.7 (C), 133.1 (C), 131.6 (C), 130.5 (C), 126.4 (CH), 125.0 (C), 122.5 (CH), 120.6 (CH), 119.9 (CH), 118.9 (CH), 112.4 (CH), 110.1 (CH), 108.7 (CH), 40.0 (CH), 33.4 (CH_2_), 31.8 (2 × CH_3_), 29.6 (CH_2_), 10.7 (CH_3_), two quaternary carbons are missing due to overlapping of signals, some signals broaden due to restricted bond rotation. LRMS (EI): *m*/*z* (%) 342 (M^+^, 100), 327 (44), 299 (81). HRMS (ESI+) *m*/*z*, calcd for C_23_H_23_N_2_O^+^ [M + H]^+^ 343.1805, found 343.1806.

#### 9-Methyl-4-(1-methyl-2-phenyl-1H-indol-3-yl)-2,3,4,9-tetrahydro-1H-carbazol-1-one (**8c**)

General procedure III was followed using 1-methyl-2-phenylindole (83 mg, 0.4 mmol) obtaining **8c**, which was isolated by flash column chromatography (hexane/EtOAc, 4/1) as a brown solid (116 mg, 72%): mp 175–177 °C. *R*_*f*_ = 0.27 (hexane/EtOAc, 4/1). ^1^H NMR (300 MHz, DMSO-*d*_6_): δ (ppm) 7.65–7.37 (bm, 8H), 7.21 (ddd, *J* = 8.3, 6.5, 1.5 Hz, 1H), 7.07 (t, *J* = 7.6 Hz, 1H), 6.90–6.69 (m, 3H), 4.51 (bs, 1H), 4.02 (s, 3H), 3.61 (s, 3H), 2.80–2.51 (m, 2H), 2.36–2.19 (m, 1H), some signals broaden due to restricted bond rotation. ^13^C{^1^H} NMR (75.4 MHz, DMSO-*d*_6_): δ 191.3 (C), 139.3 (C), 137.7 (C), 137.0 (C), 131.2 (C), 130.4 (2 × CH), 130.2 (2 × CH), 129.7 (C), 128.6 (C), 128.4 (C), 126.0 (CH), 124.3 (CH), 121.4 (CH), 121.3 (CH), 119.8 (CH), 119.3 (C), 118.9 (CH), 113.7 (C), 110.7 (CH), 110.1 (CH), 32.7 (CH_2_), 31.3 (CH_3_), 30.7 (CH), one CH_2_ is missing due to overlapping with the solvent signal, some signals broaden due to restricted bond rotation. LRMS (EI): could not be recorded. HRMS (ESI+) *m*/*z*, calcd for C_28_H_25_N_2_O^+^ [M + H]^+^ 406.1994, found 406.1994.

#### 4-(1H-Indol-3-yl)-9-methyl-2,3,4,9-tetrahydro-1H-carbazol-1-one (**8d**)

General procedure III was followed using indole (47 mg, 0.4 mmol) obtaining **8d**, which was isolated by flash column chromatography (hexane/EtOAc, 5/1) as a yellowish solid (89 mg, 71%): mp 182–184 °C. *R*_*f*_ = 0.3 (hexane/EtOAc, 5/1). ^1^H NMR (300 MHz, CDCl_3_): δ 8.08 (bs, 1H), 7.73 (dd, *J* = 7.7, 1.2 Hz, 1H), 7.46–7.36 (m, 3H), 7.38–7.14 (m, 3H), 6.99 (ddd, *J* = 8.0, 5.3, 2.5 Hz, 1H), 6.61 (dd, *J* = 2.5, 0.9 Hz, 1H), 4.94 (t, *J* = 4.7 Hz, 1H), 4.19 (s, 3H), 2.83–2.69 (m, 1H), 2.70–2.52 (m, 3H). ^13^C{^1^H} NMR (75.4 MHz, CDCl_3_): δ 192.9 (C), 140.0 (C), 136.8 (C), 130.8 (C), 130.6 (C), 126.8 (C), 126.7 (C), 124.6 (CH), 123.0 (CH), 122.3 (CH), 122.2 (CH), 120.2 (CH), 119.6 (CH), 119.1 (CH), 117.3 (C), 111.5 (CH), 110.3 (CH), 37.2 (CH), 31.9 (CH_2_), 31.8 (CH_3_), 30.7 (CH_2_). LRMS (EI): *m*/*z* (%) 314 (M^+^, 100), 285 (50), 257 (18). HRMS (ESI+) *m*/*z*, calcd for C_21_H_19_N_2_O [M + H]^+^ 315.1492, found 315.1498.

#### 9-Methyl-4-(2-phenyl-1H-indol-3-yl)-2,3,4,9-tetrahydro-1H-carbazol-1-one (**8e**)

General procedure III was followed using 2-phenylindole (77 mg, 0.4 mmol) obtaining **8e**, which was isolated by flash column chromatography (hexane/EtOAc, 3/1) as a brown solid (126 mg, 81%): mp 268–270 °C. *R*_*f*_ = 0.29 (hexane/EtOAc, 3/1). ^1^H NMR (300 MHz, DMSO-*d*_6_): δ 11.35 (s, 1H), 7.69 (bs, 2H), 7.60–7.33 (m, 5H), 7.19 (ddd, *J* = 8.4, 5.9, 2.2 Hz, 1H), 7.01–6.95 (m, 2H), 6.77–6.59 (m, 3H), 4.99–4.76 (m, 1H), 4.07 (s, 3H), 2.90–2.57 (m, 3H), 2.40–2.24 (m, 1H). ^13^C{^1^H} NMR (75.4 MHz, DMSO-*d*_6_): δ 191.4 (C), 139.3 (C), 136.3 (C), 135.0 (C), 132.9 (C), 130.6 (C), 129.7 (C), 128.8 (2 × CH), 128.6 (2 × CH), 127.7 (C), 127.0 (C), 125.9 (CH), 124.3 (CH), 121.4 (CH), 121.2 (CH), 119.7 (CH), 119.6 (C), 118.5 (CH), 113.0 (CH), 111.4 (CH), 110.7 (CH), 32.5 (CH_2_), 32.3 (CH), 31.4 (CH_3_). One CH_2_ is missing due to overlapping with the solvent signals. LRMS (EI): *m*/*z* (%) 390 (M^+^, 100), 361 (38), 347 (27). HRMS (ESI+) *m*/*z*, calcd for C_27_H_23_N_2_O [M + H]^+^ 391.1805, found 391.1800.

#### 4-(5-Bromo-1H-indol-3-yl)-9-methyl-2,3,4,9-tetrahydro-1H-carbazol-1-one (**8f**)

General procedure III was followed using 5-bromoindole (78 mg, 0.4 mmol) obtaining **8f**, which was isolated by flash column chromatography (hexane/EtOAc, 2/1) as a yellowish solid (112 mg, 71%): mp 204–206 °C. *R*_*f*_ = 0.28 (hexane/EtOAc, 2/1). ^1^H NMR (300 MHz, CDCl_3_): δ 8.26 (bs, 1H), 7.84 (d, *J* = 2.0 Hz, 1H), 7.53–7.35 (m, 2H), 7.34–7.21 (m, 3H), 6.97 (ddd, *J* = 8.0, 4.7, 3.0 Hz, 1H), 6.59 (d, *J* = 2.0 Hz, 1H), 4.83 (t, *J* = 4.7 Hz, 1H), 4.14 (s, 3H), 2.77–2.63 (m, 1H), 2.61–2.43 (m, 3H). ^13^C{^1^H} NMR (75.4 MHz, CDCl_3_): δ 192.7 (C), 140.0 (C), 135.4 (C), 130.5 (C), 130.3 (C), 128.5 (C), 126.7 (CH), 125.0 (CH), 124.4 (C), 124.2 (CH), 122.1 (CH), 121.6(CH), 120.2 (CH), 117.0 (C), 113.0 (C), 112.8 (CH), 110.4 (CH), 37.1 (CH_2_), 31.78 (CH_2_), 31.77 (CH), 30.5 (CH_3_). LRMS (EI): *m*/*z* (%) 378 (M^+^,100), 349 (17), 335 (14). HRMS (ESI+) *m*/*z*, calcd for C_21_H_18_BrN_2_O^+^ [M + H]^+^ 395.0579, found 395.0579.

#### 4-(5-Chloro-1H-indol-3-yl)-9-methyl-2,3,4,9-tetrahydro-1H-carbazol-1-one (**8g**)

General procedure III was followed using 5-chloroindole (61 mg, 0.4 mmol) obtaining **8g**, which was isolated by flash column chromatography (hexane/EtOAc, 5/1) as a brown solid (93 mg, 67%): mp 203–205 °C. *R*_*f*_ = 0.31 (hexane/EtOAc, 5/1). ^1^H NMR (300 MHz, CDCl_3_): δ 8.13 (bs, 1H), 7.68–7.62 (m, 1H), 7.40–7.35 (m, 2H), 7.32–7.24 (m, 2H), 7.18 (ddd, *J* = 8.6, 2.0, 0.3 Hz, 1H), 6.97 (ddd, *J* = 8.0, 5.3, 2.6 Hz, 1H), 6.68–6.57 (m, 1H), 4.83 (t, *J* = 4.8 Hz, 1H), 4.15 (s, 3H), 2.78–2.39 (m, 4H). ^13^C{^1^H} NMR (75.4 MHz, CDCl_3_): δ 192.7 (C), 140.0 (C), 135.2 (C), 130.5 (C), 130.3 (C), 127.8 (C), 126.7 (CH), 125.4 (C), 124.5 (C), 124.4 (CH), 122.6 (CH), 122.2 (CH), 120.3 (CH), 118.7 (C), 117.3 (CH), 112.6 (CH), 110.4 (CH), 37.1 (CH_2_), 31.82 (CH_3_), 31.79 (CH_2_), 30.6 (CH). LRMS (EI): *m*/*z* (%) 348 (M^+^, 100), 321 (30), 319 (72). HRMS (ESI+) *m*/*z*, calcd for C_21_H_18_ClN_2_O^+^ [M + H]^+^ 349.1102, found 349.1104.

#### 4-(5-Methoxy-1H-indol-3-yl)-9-methyl-2,3,4,9-tetrahydro-1H-carbazol-1-one (**8h**)

General procedure III was followed using 5-methoxyindole (59 mg, 0.4 mmol) obtaining **8h**, which was isolated by flash column chromatography (hexane/EtOAc, 3/1) as a green solid (89.5 mg, 65%): mp 117–119 °C. *R*_*f*_ = 0.31 (hexane/EtOAc, 3/1). ^1^H NMR (300 MHz, CDCl_3_): δ 7.92 (bs, 1H), 7.41–7.29 (m, 4H), 7.13 (d, *J* = 2.5 Hz, 1H), 6.97 (ddd, *J* = 8.0, 5.3, 2.5 Hz, 1H), 6.90 (ddd, *J* = 8.8, 2.5, 0.4 Hz, 1H), 6.61–6.46 (m, 1H), 4.86 (t, *J* = 4.6 Hz, 1H), 4.15 (s, 3H), 3.88 (s, 3H), 2.77–2.68 (m, 1H), 2.64–2.48 (m, 3H). ^13^C{^1^H} NMR (75.4 MHz, CDCl_3_): δ 192.9 (C), 154.1 (C), 140.0 (C), 131.9 (C), 130.8 (C), 130.6 (C), 127.2 (C), 126.7 (CH), 124.6 (C), 123.8 (CH), 122.4 (CH), 120.2 (CH), 117.0 (C), 112.24 (CH), 112.20 (CH) 110.3 (CH), 101.1 (CH), 56.1 (CH_3_), 37.2 (CH_2_), 31.8 (CH_3_), 31.7 (CH_2_), 30.7 (CH). LRMS (EI): *m*/*z* (%) 344 (M^+^, 100), 315 (50), 128 (22). HRMS (ESI+) *m*/*z*, calcd for C_22_H_21_N_2_O_2_^+^ [M + H]^+^ 345.1598, found 345.1600.

#### Methyl 3-(9-methyl-1-oxo-2,3,4,9-tetrahydro-1H-carbazol-4-yl)-1H-indole-5-carboxylate (**8i**)

General procedure III was followed using methyl 5-indolecarboxylate (64 mg, 0.4 mmol) obtaining **8i**, which was isolated by flash column chromatography (hexane/EtOAc, 5/1) as a brown solid (119 mg, 80%): mp 210–212 °C. *R*_*f*_ = 0.27 (hexane/EtOAc, 5/1). ^1^H NMR (300 MHz, CDCl_3_): δ 8.60–8.35 (m, 2H), 7.94 (dd, *J* = 8.6, 1.6 Hz, 1H), 7.43–7.33 (m, 3H), 7.31–7.20 (m, 1H), 6.96 (ddd, *J* = 8.0, 5.4, 2.4 Hz, 1H), 6.63 (d, *J* = 2.4 Hz, 1H), 4.94 (t, *J* = 4.1 Hz, 1H), 4.14 (s, 3H), 3.95 (s, 3H), 2.80–2.43 (m, 4H). ^13^C{^1^H} NMR (75.4 MHz, CDCl_3_): δ 192.7 (C), 168.3 (C), 140.0 (C), 139.4 (C), 130.6 (C), 130.3 (C), 126.7 (CH), 126.5 (C), 124.42 (C), 124.38 (CH), 123.7 (CH), 122.2 (CH), 122.0 (CH), 121.7 (CH), 120.3 (CH), 118.9 (C), 111.4 (CH), 110.4 (CH), 52.1 (CH_3_), 37.0 (CH_2_), 31.9 (CH_3_), 31.8 (CH_2_), 30.4 (CH). LRMS (EI): *m*/*z* (%) 372 (M^+^, 100), 343 (55), 128 (18). HRMS (ESI+) *m*/*z*, calcd for C_23_H_21_N_2_O_3_^+^ [M + H]^+^ 373.1547, found 373.1546.

#### 9-Methyl-4-(6-nitro-1H-indol-3-yl)-2,3,4,9-tetrahydro-1H-carbazol-1-one (**8j**)

General procedure III was followed using 6-nitroindole (65 mg, 0.4 mmol) obtaining **8j**, which was isolated by flash column chromatography (hexane/EtOAc, 3/1) as a yellow solid (121 mg, 84%): mp 177–179 °C. *R*_*f*_ = 0.30 (hexane/EtOAc, 3/1). ^1^H NMR (300 MHz, CDCl_3_): δ 8.77 (bs, 1H), 8.37 (dd, *J* = 2.1, 0.5 Hz, 1H), 8.01 (dd, *J* = 8.8, 2.1 Hz, 1H), 7.66 (d, *J* = 8.8 Hz, 1H), 7.43–7.31 (m, 2H), 7.17 (dt, *J* = 8.2, 1.0 Hz, 1H), 7.00 (dd, *J* = 2.6, 0.7 Hz, 1H), 6.95 (ddd, *J* = 8.2, 6.0, 1.8 Hz, 1H), 4.90 (t, *J* = 5.1 Hz, 1H), 4.16 (s, 3H), 2.86–2.38 (m, 4H). ^13^C{^1^H} NMR (75.4 MHz, CDCl_3_): δ 192.4 (C), 143.5 (C), 140.1 (C), 135.3 (C), 131.3 (C), 130.5 (C), 129.7 (C), 128.8 (CH), 126.9 (CH), 124.3 (C), 122.0 (CH), 120.5 (CH), 119.1 (CH), 118.8 (C), 115.2 (CH), 110.6 (CH), 108.6 (CH), 37.4 (CH_2_), 32.2 (CH_2_), 31.9 (CH_3_), 30.8 (CH). LRMS (EI): could not be recorded. HRMS (ESI+) *m*/*z*, calcd for C_21_H_18_N_3_O_3_^+^ [M + H]^+^ 360.1343, found 360.1344.

#### 9-Methyl-4-(3-methyl-1H-indol-2-y)-2,3,4,9-tetrahydro-1H-carbazol-1-one (**8k**)

General procedure III was followed using 3-methylindole (53 mg, 0.4 mmol) obtaining **8k**, which was isolated by flash column chromatography (hexane/EtOAc, 3/1) as a gray solid (111 mg, 85%): mp 235–239 °C. *R*_*f*_ = 0.33 (hexane/EtOAc, 3/1). ^1^H NMR (300 MHz, CDCl_3_): δ 7.68 (bs, 1H), 7.66–7.57 (m, 1H), 7.46–7.32 (m, 2H), 7.20–7.08 (m, 3H), 7.02–6.84 (m, 2H), 4.83 (dd, *J* = 8.8, 4.9 Hz, 1H), 4.12 (s, 3H), 2.90–2.64 (m, 2H), 2.60–2.46 (m, 1H), 2.46 (s, 3H), 2.46–2.29 (m, 1H). ^13^C{^1^H} NMR (75.4 MHz, CDCl_3_): δ 192.0 (C), 139.9 (C), 135.4 (C), 135.2 (C), 130.8 (C), 129.4 (C), 128.0 (C), 127.0 (CH), 124.4 (C), 122.1 (CH), 121.6 (CH), 120.9 (CH), 119.3 (CH), 118.5 (CH), 110.8 (CH), 110.4 (CH), 107.8 (C), 38.9 (CH_2_), 32.8 (CH_2_), 32.2 (CH_3_), 31.8 (CH), 8.8 (CH_3_). LRMS (EI): *m*/*z* (%) 328 (M+, 100), 313 (29), 285 (21). HRMS (ESI+) *m*/*z*, calcd for C_22_H_21_N_2_O^+^ [M + H]^+^ 329.1648, found 329.1650.

#### 9-Methyl-1-(1-methyl-1H-indol-3-yl)-9H-cabazole (**9a**)

Isolated by flash column chromatography (hexane/EtOAc, 5/1) as a pink oil (13 mg, 30%): *R*_*f*_ = 0.41 (hexane/EtOAc, 5/1). ^1^H NMR (300 MHz, CDCl_3_): δ 8.30–8.12 (m, 2H), 7.57–7.41 (m, 4H), 7.38–7.25 (m, 4H), 7.20 (s, 1H), 7.14 (ddd, *J* = 7.9, 7.0, 1.0 Hz, 1H), 3.93 (s, 3H), 3.46 (s, 3H). ^13^C{^1^H} NMR (75.4 MHz, CDCl_3_): δ 142.3 (C), 140.0 (C), 136.6 (C), 130.3 (CH), 129.7 (C), 127.9 (CH), 125.8 (CH), 124.0 (C), 123.1 (C), 122.2 (CH), 120.5 (CH), 120.2 (CH), 119.9 (CH), 119.3 (CH), 119.1 (CH), 118.9 (CH), 118.3 (C), 114.9 (C), 109.4 (CH), 109.0 (CH), 33.1 (CH_3_), 31.7 (CH_3_). LRMS (EI): *m*/*z* (%) 310 (M^+^, 100), 279 (17), 147 (20). HRMS (ESI+) *m*/*z*, calcd for C_22_H_18_N_2_ [M + H]^+^ 311.2383, found 311.2378.

#### 1-(1-Methyl-1H-indol-2-yl)-4,4-bis(1-methyl-1H-indol-3-yl)butan-1-one (**S3**)

Isolated by flash column chromatography (hexane/EtOAc, 5/1) as a pink oil (20 mg, 31%): *R*_*f*_ = 0.15 (hexane/EtOAc, 5/1). ^1^H NMR (300 MHz, CDCl_3_): δ 7.70 (dd, *J* = 8.0, 1.0 Hz, 2H), 7.61 (dd, *J* = 8.0, 1.0 Hz, 1H), 7.41–7.36 (m, 2H), 7.34–7.20 (m, 5H), 7.18–7.02 (m, 4H), 6.93 (s, 2H), 4.82–4.50 (m, 1H), 4.07 (d, *J* = 1.0 Hz, 3H), 3.74 (d, *J* = 1.0 Hz, 6H), 3.10 (t, *J* = 7.5 Hz, 2H), 2.81–2.63 (m, 2H). ^13^C{^1^H} NMR (75.4 MHz, CDCl_3_): δ 194.9 (C), 140.0 (C), 137.5 (C), 127.6 (C), 126.6 (2 × CH), 125.9 (2 × C), 125.8 (CH), 122.9 (CH), 121.5 (2 × CH), 120.7 (CH), 120.0 (2 × CH), 118.7 (2 × CH), 118.4 (2 × C), 111.4 (CH), 110.4 (CH), 109.3 (2 × CH), 39.0 (CH_2_), 33.8 (CH), 32.7 (2 × CH_3_), 32.3 (CH_3_), 31.5 (CH_2_). LRMS (EI): *m*/*z* (%) could not be recorded. HRMS (ESI+) *m*/*z*, calcd for C_31_H_30_N_3_O [M + H]^+^ 460.2383, found 460.2385.

### General Procedure IV for the Synthesis of Tetrahydrocarbazol-1-ones **10** from **1a**

To a stirred solution of 4-(1-methyl-1*H*-indol-2-yl)-4-oxobutanal (**1a**) (86 mg, 0.4 mmol) in anhydrous HFIP (4 mL) were added the corresponding nucleophile (0.4 mmol) and *p*-TsOH (7.6 mg), and the resulted solution was stirred at rt for 2 h (monitored by TLC). Then, the resulting mixture was quenched with water (2 mL) and extracted with Et_2_O (3 × 5 mL). The combined organic layers were dried over anhydrous Na_2_SO_4_, filtered and concentrated in vacuo. The residue was purified by flash column chromatography using silica gel with mixtures of hexane/EtOAc as eluent to afford the corresponding tetrahydrocarbazolones **10a**,**c–f**,**h**,**j–m**.

### General Procedure V for the Synthesis of Tetrahydrocarbazol-1-ones **10** from **4b**

To a stirred solution of 1-(1*H*-indol-2-yl)-4,4-dimethoxybutan-1-one (**4b**) (99 mg, 0.3 mmol) in anhydrous HFIP (3 mL) were added the corresponding nucleophile (0.3 mmol) and *p*-TsOH (5.7 mg), and the resulted solution was stirred at rt for 2 h (monitored by TLC). Then, the resulting mixture was quenched with water (2 mL) and extracted with ether (3 × 5 mL). The combined organic layers were dried over anhydrous Na_2_SO_4_, filtered and concentrated in vacuo. The residue was purified by flash column chromatography using silica gel and mixtures of hexane/EtOAc as eluent to afford the corresponding tetrahydrocarbazolones **10b**,**g**,**i**,**n**.

#### 9-Methyl-4-(2,4,6-trimethoxyphenyl)-2,3,4,9-tetrahydro-1H-carbazol-1-one (**10a**)

General procedure IV was followed using 1,3,5-trimethoxybenzene (67 mg, 0.4 mmol) obtaining **10a**, which was isolated by flash column chromatography (hexane/EtOAc, 5/1) as a pink solid (110 mg, 75%): mp 171–175 °C. *R*_*f*_ = 0.25 (hexane/EtOAc, 5/1). ^1^H NMR (300 MHz, CDCl_3_): δ 7.27 (dt, *J* = 3.9, 1.4 Hz, 2H), 6.88–6.71 (m, 2H), 6.18 (s, 2H), 5.14–4.95 (m, 1H), 4.10 (s, 3H), 3.85 (s, 3H), 3.55 (bs, 6H), 2.92–2.61 (m, 3H), 2.25–2.07 (m, 1H). ^13^C{^1^H} NMR (75.4 MHz, CDCl_3_): δ 192.8 (C), 160.2 (C), 139.8 (2 × C), 133.7 (C), 130.0 (C), 126.0 (CH), 124.8 (C), 121.9 (CH), 119.3 (CH), 112.0 (C), 109.9 (CH), 91.4 (2 × CH), 55.8 (CH_3_), 55.4 (2 × CH_3_), 40.5 (CH_2_), 31.6 (CH), 31.0 (CH_3_), 30.5 (CH_2_), one quaternary carbon is missing due to overlapping of signals. HRMS (ESI+) *m*/*z*, calcd for C_22_H_24_NO_4_^+^ [M + H]^+^ 366.1700, found 366.1708.

#### 4-(2,4,6-Trimethoxyphenyl)-2,3,4,9-tetrahydro-1H-carbazol-1-one (**10b**)

General procedure V was followed using 1,3,5-trimethoxybenzene (60.4 mg, 0.3 mmol) obtaining **10b**, which was isolated by flash column chromatography (hexane/EtOAc, 2/1) as a colorless solid (53 mg, 50%): mp 223–225 °C. *R*_*f*_ = 0.36 (hexane/EtOAc, 2/1). ^1^H NMR (300 MHz, CDCl_3_): δ 8.99 (bs, 1H), 7.41–7.34 (m, 1H), 7.26–7.20 (m, 1H), 6.95–6.69 (m, 2H), 6.18 (bs, 2H), 5.22–4.83 (m, 1H), 3.86 (s, 3H), 3.56 (bs, 6H), 3.01–2.66 (m, 3H), 2.33–2.11 (m, 1H). ^13^C{^1^H} NMR (75.4 MHz, CDCl_3_): δ 191.8 (C), 160.3 (C), 138.1 (2 × C), 134.2 (C), 130.8 (C), 126.5 (CH), 126.1 (C), 121.9 (CH), 119.7 (CH), 112.3 (CH), 111.7 (C), 91.4 (2 × CH), 55.9 (2 × CH_3_), 55.4 (CH_3_), 38.8 (CH_2_), 30.8 (CH_2_), 30.6 (CH), one quaternary carbon is missing due to overlapping of signals. LRMS (EI): *m*/*z* (%) 351 (M^+^, 100), 350 (20), 292 (25). HRMS (ESI+) *m*/*z*, calcd for C_21_H_22_NO_4_^+^ [M + H]^+^ 352.1543, found 352.1546.

#### 4-(2,4-Dimethoxyphenyl)-9-methyl-2,3,4,9-tetrahydro-1H-carbazol-1-one (**10c**)

General procedure IV was followed using 1,3-dimethoxybenzene (83 mg, 0.4 mmol) obtaining **10c**, which was isolated by flash column chromatography (hexane/EtOAc, 5/1) as a yellow oil (95 mg, 71%): *R*_*f*_ = 0.26 (hexane/EtOAc, 5/1). ^1^H NMR (300 MHz, CDCl_3_): δ 7.48–7.26 (m, 2H), 7.21–7.03 (m, 1H), 6.97 (ddd, *J* = 8.0, 4.8, 3.0 Hz, 1H), 6.69 (d, *J* = 8.4 Hz, 1H), 6.58 (d, *J* = 2.4 Hz, 1H), 6.30 (dd, *J* = 8.4, 2.4 Hz, 1H), 4.91 (t, *J* = 5.5 Hz, 1H), 4.15 (s, 3H), 3.91 (s, 3H), 3.79 (s, 3H), 2.78–2.44 (m, 3H), 2.34–2.18 (m, 1H). ^13^C{^1^H} NMR (75.4 MHz, CDCl_3_): δ 193.0 (C), 159.7 (C), 158.0 (C), 140.1 (C), 131.3 (C), 131.0 (C), 129.5 (CH), 126.6 (CH), 124.5 (C), 123.2 (C), 122.4 (CH), 120.0 (CH), 110.2 (C), 103.9 (CH), 98.7 (CH), 55.6 (CH_3_), 55.4 (CH_3_), 37.6 (CH_2_), 32.3 (CH_3_), 32.0 (CH), 31.7 (CH_2_). LRMS (EI): *m*/*z* (%) 335 (M^+^, 100), 306 (36), 276 (29). HRMS (ESI+) *m*/*z*, calcd for C_21_H_22_NO_3_^+^ [M + H]^+^ 336.1594, found 336.1596.

#### 4-(2-Hydroxy-4,5-dimethoxyphenyl)-9-methyl-2,3,4,9-tetrahydro-1H-carbazol-1-one (**10d**)

General procedure IV was followed using 3,4-dimethoxyphenol (62 mg, 0.4 mmol) obtaining **10d**, which was isolated by flash column chromatography (hexane/EtOAc (1/2) as a brown solid (108 mg, 77%): mp 105–107 °C. *R*_*f*_ = 0.5 (hexane/EtOAc, 1/2). ^1^H NMR (300 MHz, CDCl_3_): δ 7.33 (d, *J* = 3.5 Hz, 2H), 7.09–6.97 (m, 1H), 6.96–6.82 (m, 1H), 6.52 (d, *J* = 13.8 Hz, 2H), 5.84 (bs, 1H), 4.85–4.67 (m, 1H), 4.12 (s, 3H), 3.82 (s, 3H), 3.61 (s, 3H), 2.82–2.59 (m, 2H), 2.45–2.38 (m, 2H). ^13^C{^1^H} NMR (75.4 MHz, CDCl_3_): δ 192.9 (C), 148.7 (C), 147.9 (C), 143.0 (C), 140.1 (C), 130.8 (C), 130.6 (C), 126.9 (CH), 124.4 (C), 122.5 (CH), 120.3 (CH), 113.6 (CH), 110.3 (CH), 101.4 (CH), 56.9 (CH_3_), 56.0 (CH_3_), 38.8 (CH_2_), 34.9 (CH), 32.5 (CH_2_), 31.8 (CH_3_), one quaternary carbon is missing due to overlapping of signals. LRMS (EI): *m*/*z* (%) could not be recorded. HRMS (ESI+) *m*/*z*, calcd for C_21_H_22_NO_4_^+^ [M + H]^+^ 352.1543, found 352.1546.

#### 4-(3,5-Dimethyl-1H-pyrrol-2-yl)-9-methyl-2,3,4,9-tetrahydro-1H-carbazol-1-one (**10e**)

General procedure IV was followed using 2,5-dimethylpyrrol (38 mg, 0.4 mmol) obtaining **10e**, which was isolated by flash column chromatography (hexane/EtOAc, 3/1) as a yellow solid (78 mg, 67%): mp 220–222 °C. *R*_*f*_ = 0.4 (hexane/EtOAc, 3/1). ^1^H NMR (300 MHz, CDCl_3_): δ 7.52–7.27 (m, 3H), 7.12–6.90 (m, 2H), 5.75 (d, *J* = 2.5 Hz, 1H), 4.54 (dd, *J* = 9.1, 4.7 Hz, 1H), 4.08 (s, 3H), 2.88–2.59 (m, 2H), 2.47–2.35 (m, 1H), 2.32–2.23 (m, 1H), 2.15 (s, 3H), 2.11 (s, 3H). ^13^C{^1^H} NMR (75.4 MHz, CDCl_3_): δ 192.3 (C), 139.9 (C), 130.6 (C), 129.8 (C), 126.8 (CH), 126.7 (C), 125.9 (C), 124.6 (C), 122.5 (CH), 120.6 (CH), 114.9 (C), 110.2 (CH), 108.0 (CH), 39.1 (CH_2_), 33.3 (CH_2_), 32.0 (CH), 31.7 (CH_3_), 13.1 (CH_3_), 11.1 (CH_3_). LRMS (EI): *m*/*z* (%) 292 (M^+^, 100), 249 (51), 235 (45). HRMS (ESI+) *m*/*z*, calcd for C_19_H_21_N_2_O^+^ [M + H]^+^ 293.1648, found 293.1650.

#### 9-Methyl-4-(5-methylfuran-2-yl)-2,3,4,9-tetrahydro-1H-carbazol-1-one (**10f**)

General procedure IV was followed using 2-methylfurane (49 mg, 0.4 mmol) obtaining **10f**, which was isolated by flash column chromatography (hexane/EtOAc, 6/1) as a colorless oil (90.5 mg, 81%): *R*_*f*_ = 0.39 (hexane/EtOAc, 6/1). ^1^H NMR (300 MHz, CDCl_3_): δ 7.47–7.33 (m, 3H), 7.08 (ddd, *J* = 8.0, 5.8, 2.2 Hz, 1H), 5.90–5.85 (m, 1H), 5.81 (d, *J* = 3.0 Hz, 1H), 4.55 (t, *J* = 5.1 Hz, 1H), 4.11 (s, 3H), 2.95–2.72 (m, 1H), 2.65–2.39 (m, 3H), 2.28 (s, 3H). ^13^C{^1^H} NMR (75.4 MHz, CDCl_3_): δ 192.1 (C), 153.6 (C), 151.3 (C), 139.8 (C), 130.3 (C), 128.0 (C), 126.6 (CH), 124.6 (C), 121.9 (CH), 120.4 (CH), 110.3 (CH), 107.6 (CH), 106.1 (CH), 37.4 (CH_2_), 33.3 (CH), 31.7 (CH_3_), 30.6 (CH_2_), 13.7 (CH_3_). LRMS (EI): *m*/*z* (%) could not be recorded. HRMS (ESI+) *m*/*z*, calcd for C_18_H_18_NO_2_^+^ [M + H]^+^ 280.1332, found 280.1332.

#### 4-(5-Methylfuran-2-yl)-2,3,4,9-tetrahydro-1H-carbazol-1-one (**10g**)

General procedure V was followed using 2-methylfuran (24.6 mg, 0.3 mmol) obtaining **10g**, which was isolated by flash column chromatography (hexane/EtOAc, 5/1) as a brown solid (40 mg, 50%): mp 201–203 °C. *R*_*f*_ = 0.31 (hexane/EtOAc, 5/1). ^1^H NMR (300 MHz, CDCl_3_): δ 9.27 (bs, 1H), 7.52–7.29 (m, 3H), 7.08 (ddd, *J* = 8.1, 6.9, 1.1 Hz, 1H), 5.99–5.62 (m, 2H), 4.55 (t, *J* = 5.2 Hz, 1H), 2.93–2.76 (m, 1H), 2.68–2.47 (m, 3H), 2.27 (d, *J* = 1.0 Hz, 3H). ^13^C{^1^H} NMR (75.4 MHz, CDCl_3_): 191.3 (C), 153.3 (C), 151.4 (C), 138.0 (C), 131.2 (C), 128.4 (C), 127.1 (CH), 125.8 (C), 122.1 (CH), 120.8 (CH), 112.7 (CH), 107.6 (CH), 106.2 (CH), 36.0 (CH_2_), 33.1 (CH_2_), 31.0 (CH), 13.8 (CH_3_). LRMS (EI): *m*/*z* (%) 265 (M^+^, 100), 222 (70), 194 (63). HRMS (ESI+) *m*/*z*, calcd for C_17_H_16_NO_2_^+^ [M + H]^+^ 267.1208, found 267.1208.

#### 9-Methyl-4-(5-methylthiophen-2-yl)-2,3,4,9-tetrahydro-1H-carbazol-1-one (**10h**)

General procedure IV was followed using 2-methylthiophene (59 mg, 0.4 mmol) obtaining **10h**, which was isolated by flash column chromatography (hexane/EtOAc, 5/1) as a brown oil (92 mg, 78%): *R*_*f*_ = 0.25 (hexane/EtOAc, 5/1). ^1^H NMR (300 MHz, CDCl_3_): δ 7.68–7.31 (m, 3H), 7.15–7.00 (m, 1H), 6.58 (d, *J* = 0.9 Hz, 2H), 4.74 (t, *J* = 5.2 Hz, 1H), 4.13 (s, 3H), 2.97–2.76 (m, 1H), 2.64–2.51 (m, 2H), 2.44 (s, 3H), 2.41–2.26 (m, 1H). ^13^C{^1^H} NMR (75.4 MHz, CDCl_3_): δ 192.0 (C), 144.2 (C), 139.8 (C), 138.5 (C), 130.0 (C), 129.7 (C), 126.7 (CH), 125.1 (CH), 124.7 (CH), 124.5 (C), 122.1 (CH), 120.4 (CH), 110.4 (CH), 37.3 (CH_2_), 35.0 (CH), 34.5 (CH_2_), 31.7 (CH_3_), 15.5 (CH_3_). LRMS (EI): *m*/*z* (%) 295 (M^+^, 100), 266 (46), 97 (24). HRMS (ESI+) *m*/*z*, calcd for C_18_H_18_NOS^+^ [M + H]^+^ 296.1104, found 296.1105.

#### 4-(5-Methylthiophen-2-yl)-2,3,4,9-tetrahydro-1H-carbazol-1-one (**10i**)

General procedure V was followed using 2-methylthiophene (29.4 mg, 0.3 mmol) obtaining **10i**, which was isolated by flash column chromatography (hexane/EtOAc, 5/1) as a colorless solid (45 mg, 53%): mp 191–193 °C. *R*_*f*_ = 0.3 (hexane/EtOAc, 5/1). ^1^H NMR (300 MHz, CDCl_3_): δ 9.64 (bs, 1H), 7.63–7.45 (m, 1H), 7.43–7.30 (m, 2H), 7.06 (ddd, *J* = 8.0, 6.9, 1.0 Hz, 1H), 6.59 (dd, *J* = 4.5, 1.0 Hz, 2H), 4.74 (t, *J* = 5.4 Hz, 1H), 2.95–2.78 (m, 1H), 2.76–2.56 (m, 2H), 2.55–2.36 (m, 4H). ^13^C{^1^H} NMR (75.4 MHz, CDCl_3_): δ 191.3 (C), 143.9 (C), 138.6 (C), 138.2 (C), 131.0 (C), 130.2 (C), 127.1 (CH), 125.7 (C), 125.2 (CH), 124.8 (CH), 122.1 (CH), 120.8 (CH), 112.8 (CH), 35.9 (CH_2_), 35.0 (CH_2_), 34.9 (CH), 15.5 (CH_3_). LRMS (EI): *m*/*z* (%) 257 (M^+^, 100), 116 (95), 77 (53). HRMS (ESI+) *m*/*z*, calcd for C_17_H_16_NOS^+^ [M + H]^+^ 282.0947, found 282.0947.

#### 4-(3-Methoxythiophen-2-yl)-9-methyl-2,3,4,9-tetrahydro-1H-carbazol-1-one (**10j**, major) and 4-(4-methoxythiophen2-yl)-9-methyl-2,3,4,9-tetrahydro-1H-carbazol-1-one (**10j′**, minor)

General procedure IV was followed using 3-methoxythiophene (46 mg, 0.4 mmol) obtaining **10j** as a c.a. 5/1 mixture of regioisomers, which were isolated as a c.a. 4/1 mixture of regioisomers by flash column chromatography (hexane/EtOAc, 5/1) as a brown oil (87 mg, 70%): *R*_*f*_ = 0.3 (hexane/EtOAc, 5/1). Data for both regioisomers: ^1^H NMR (500 MHz, CDCl_3_): δ 7.49–7.35 (m, 2H **10j** + **10j′**), 7.34–7.18 (m, 1H **10j**), 7.10–6.99 (m, 2H **10j** + **10j′**), 6.93 (d, *J* = 5.5 Hz, 1H **10j**), 6.50 (dd, *J* = 1.7, 0.9 Hz, 1H **10j′**), 6.11 (d, *J* = 1.7 Hz, 1H **10j′**), 4.95 (dd, *J* = 6.8, 4.9 Hz, 1H **10j**), 4.73 (t, *J* = 5.1 Hz, 1H **10j′**), 4.14 (d, *J* = 1.1 Hz, 3H **10j** + **10j′**), 3.94 (s, 3H **10j**), 3.78 (s, 3H **10j′**), 2.95–2.80 (m, 1H **10j** + **10j′**), 2.69–2.40 (m, 3H **10j** + **10j′**). ^13^C{^1^H} NMR (75.4 MHz, CDCl_3_): δ 192.3 (C), 191.8 (C), 157.6 (C), 153.7 (C), 145.8 (C), 139.9 (C), 130.3 (C), 130.2 (C), 128.9 (C), 126.8 (CH), 126.6 (CH), 124.5 (C), 124.47 (C), 123.4 (C), 122.0 (CH), 121.6 (CH), 120.6 (CH), 120.4 (CH), 118.1 (CH), 116.4 (CH), 110.5 (CH), 110.3 (CH), 95.1 (CH), 59.0 (CH_3_), 57.1 (CH_3_), 38.1 (CH_2_), 37.1 (CH_2_), 35.4 (CH), 34.2 (CH_2_), 33.0 (CH_2_), 31.8 (CH), 31.7 (CH_3_), 31.2 (CH_3_). LRMS (EI): *m*/*z* (%) 311 (M^+^, 100), 282 (17), 252 (32). HRMS (ESI+) *m*/*z*, calcd for C_18_H_18_NO_2_S^+^ [M + H]^+^ 312.1053, found 312.1056.

#### 4-(5-Bromo-4-methylthiophen-2-yl)-9-methyl-2,3,4,9-tetrahydro-1H-carbazol-1-one (**10k**)

General procedure IV was followed using 2-bromo-3-methylthiophene (71 mg, 0.4 mmol) obtaining **10k**, which was isolated by flash column chromatography (hexane/EtOAc, 5/1) as a brown oil (102 mg, 68%): *R*_*f*_ = 0.32 (hexane/EtOAc, 5/1). ^1^H NMR (300 MHz, CDCl_3_): δ 7.62–7.34 (m, 3H), 7.10 (ddd, *J* = 7.9, 5.6, 2.5 Hz, 1H), 6.45 (d, *J* = 0.9 Hz, 1H), 4.69 (t, *J* = 4.8 Hz, 1H), 4.12 (s, 3H), 2.96–2.65 (m, 1H), 2.69–2.51 (m, 2H), 2.45–2.28 (m, 1H), 2.09 (s, 3H). ^13^C{^1^H} NMR (75.4 MHz, CDCl_3_): δ 191.7 (C), 145.9 (C), 139.8 (C), 136.9 (C), 130.2 (C), 128.4 (C), 127.5 (CH), 126.9 (CH), 124.4 (C), 121.9 (CH), 120.7 (CH), 110.5 (CH), 107.3 (C), 36.9 (CH_2_), 34.9 (CH), 34.2 (CH_2_), 31.8 (CH_3_), 15.4 (CH_3_). LRMS (EI): *m*/*z* (%) 375 (M^+^, 100), 294 (58), 266 (52). HRMS (ESI+) *m*/*z*, calcd for C_18_H_18_BrNOS^+^ [M + H]^+^ 375.0240, found 375.0239.

#### 9-Methyl-4-(phenylsulfonyl)-2,3,4,9-tetrahydro-1H-carbazol-1-one (**10l**)

General procedure IV was followed using benzenesulfinic acid sodium salt (197 mg, 0.4 mmol) obtaining **10l** with a 65% of conversion, which was isolated by flash column chromatography (hexane/EtOAc, 3/1) as a brown solid (71 mg, 52%): mp 210–212 °C. *R*_*f*_ = 0.43 (hexane/EtOAc, 5/1). ^1^H NMR (300 MHz, CDCl_3_): δ 7.88–7.73 (m, 2H), 7.75–7.59 (m, 1H), 7.54–7.44 (m, 2H), 7.47–7.33 (m, 2H), 7.17 (dt, *J* = 8.3, 1.0 Hz, 1H), 7.04 (dt, *J* = 8.1, 4.0 Hz, 1H), 4.81 (dd, *J* = 5.7, 1.8 Hz, 1H), 4.13 (s, 3H), 3.40–3.16 (m, 1H), 2.98–2.76 (m, 1H), 2.65–2.42 (m, 2H). ^13^C{^1^H} NMR (75.4 MHz, CDCl_3_): δ 190.5 (C), 139.4 (C), 138.2 (C), 134.2 (CH), 131.6 (CH), 129.4 (2 × CH), 129.2 (2 × CH), 126.9 (CH), 124.9 (C), 121.9 (CH), 121.4 (CH), 117.3 (C), 110.5 (CH), 60.0 (CH), 35.4 (CH_2_), 32.0 (CH_3_), 25.6 (CH_2_). LRMS (EI): *m*/*z* (%) could not be recorded. HRMS (ESI+) *m*/*z*, calcd for C_19_H_18_NO_3_S^+^ [M + H]^+^ 341.1033, found 341.1033.

#### 4-(2,2-Diphenylvinyl)-9-methyl-2,3,4,9-tetrahydro-1H-carbazol-1-one (**10m**)

General procedure IV was followed using 1,1-diphenylethylene (72 mg, 0.4 mmol) obtaining **10m**, which was isolated by flash column chromatography (hexane/EtOAc, 5/1) as a yellow solid (121 mg, 80%): mp 171–173 °C. *R*_*f*_ = 0.42 (hexane/EtOAc, 5/1). ^1^H NMR (300 MHz, CDCl_3_): δ 7.79 (d, *J* = 8.1 Hz, 1H), 7.51–7.35 (m, 7H), 7.32–7.25 (m, 5H), 7.10 (ddd, *J* = 8.1, 6.1, 1.8 Hz, 1H), 6.31 (d, *J* = 10.3 Hz, 1H), 4.09 (s, 3H), 4.08–3.99 (m, 1H), 2.86–2.69 (m, 1H), 2.68–2.48 (m, 1H), 2.38–2.11 (m, 2H). ^13^C{^1^H} NMR (75.4 MHz, CDCl_3_): δ 192.1 (C), 142.4 (C), 142.1 (C), 140.0 (C), 139.9 (C), 131.3 (CH), 130.6 (C), 129.9 (2 × CH), 129.8 (C), 128.7 (2 × CH), 128.4 (2 × CH), 127.6 (2 × CH), 127.53 (CH), 127.47 (CH), 126.6 (CH), 125.0 (C), 122.4 (CH), 120.4 (CH), 110.4 (CH), 38.6 (CH_2_), 35.3 (CH), 32.3 (CH_2_), 31.7 (CH_3_). LRMS (EI): *m*/*z* (%) 377 (M^+^, 100), 272 (23), 167 (51). HRMS (ESI+) *m*/*z*, calcd for C_27_H_24_NO^+^ [M + H]^+^ 378.1852, found 378.1854.

#### 4-(2,2-Diphenylvinyl)2,3,4,9-tetrahydro-1H-carbazol-1-one (**10n**)

General procedure V was followed using 1,1-diphenylethylene (54 mg, 0.3 mmol) obtaining **10n**, which was isolated by flash column chromatography (hexane/EtOAc, 5/1) as an orange solid (49 mg, 45%): mp 264–266 °C. *R*_f_ = 0.24 (hexane/EtOAc, 5/1). ^1^H NMR (300 MHz, CDCl_3_): δ 9.11 (bs, 1H), 7.83 (d, *J* = 8.2 Hz, 1H), 7.55–7.39 (m, 6H), 7.37–7.25 (m, 6H), 7.14 (ddd, *J* = 8.2, 6.8, 1.1 Hz, 1H), 6.34 (d, *J* = 10.3 Hz, 1H), 4.25–3.96 (m, 1H), 2.79 (dt, *J* = 17.1, 4.9 Hz, 1H), 2.62 (ddd, *J* = 16.8, 11.1, 4.9 Hz, 1H), 2.36–2.22 (m, 2H). ^13^C{^1^H} NMR (75.4 MHz, CDCl_3_): δ 191.1 (C), 142.7 (C), 142.0 (C), 139.8 (C), 138.1 (C), 130.9 (CH), 130.8 (C), 130.7 (C), 129.9 (2 × CH), 128.8 (2 × CH), 128.4 (2 × CH), 127.63 (CH), 127.60 (CH), 127.5 (2 × CH), 127.0 (CH), 126.2 (C), 122.4 (CH), 120.8 (CH), 112.7 (CH), 37.0 (CH_2_), 35.1 (CH), 32.7 (CH_2_). LRMS (EI): *m*/*z* (%) 363 (M^+^, 100), 167 (78), 152 (32). HRMS (ESI+) *m*/*z*, calcd for C_26_H_22_NO^+^ [M + H]^+^ 364.1696, found 364.1697.

### 2 mmol-Scale Synthesis of Tetrahydrocarbazol-1-one **10a**

To a stirred solution of 4-(1-methyl-1*H*-indol-2-yl)-4-oxobutanal (**1a**) (430 mg, 2 mmol) in anhydrous HFIP (10 mL) were added 1,3,5-trimethoxybenzene (337 mg, 2 mmol) and *p*-TsOH (38 mg, 0.2 mmol), and the resulted solution was stirred at rt for 2 h (monitored by TLC). Then, the resulting mixture was quenched with water (5 mL) and extracted with Et_2_O (3 × 10 mL). The combined organic layers were dried over anhydrous Na_2_SO_4_, filtered and concentrated in vacuo. The residue was purified by flash column chromatography using silica gel and a 5/1 mixture of hexane/EtOAc as eluent to afford the tetrahydrocarbazolone **10a** as a pink solid (534 mg, 73%). Characterization data have been reported above.

### General Procedure VI for the Synthesis of Diones **11**

To a stirred solution of 4-(1-methyl-1*H*-indol-2-yl)-4-oxobutanal (**1a**) (107 mg, 0.5 mmol) in anhydrous DCM (5 mL) was added the corresponding ylide (0.55 mmol), and the resulting solution was stirred at rt for 16 h. The residue was purified by flash column chromatography using silica gel and mixtures of hexane/EtOAc as eluent to afford the corresponding diones **11a**–**b**.

#### (E)-1-(1-Methyl-1H-indol-2-yl)hept-4-ene-1,6-dione (**11a**)

General procedure VI was followed using 1-(tryphenylphosphoranylidene)-2-propanone (175 mg, 0.55 mmol) obtaining **11a**, which was isolated by flash column chromatography (hexane/EtOAc, 3/1) as a brown oil (87 mg, 68%): *R*_*f*_ = 0.30 (hexane/EtOAc, 3/1). ^1^H NMR (300 MHz, CDCl_3_): δ 7.69 (dt, *J* = 8.1, 1.0 Hz, 1H), 7.46–7.35 (m, 2H), 7.30 (s, 1H), 7.16 (ddd, *J* = 8.0, 4.6, 3.3 Hz, 1H), 6.89 (dt, *J* = 16.0, 6.7 Hz, 1H), 6.15 (dt, *J* = 16.0, 1.6 Hz, 1H), 4.07 (d, *J* = 1.0 Hz, 3H), 3.31–3.10 (m, 2H), 2.76–2.54 (m, 2H), 2.25 (s, 3H). ^13^C{^1^H} NMR (75.4 MHz, CDCl_3_): δ 198.5 (C), 192.2 (C), 146.6 (CH), 140.2 (C), 134.4 (C), 131.9 (CH), 126.2 (CH), 125.8 (C), 123.0 (CH), 120.9 (CH), 111.5 (CH), 110.5 (CH), 37.8 (CH_2_), 32.3 (CH_3_), 27.1 (CH_2_), 27.0 (CH_3_). LRMS (EI): *m*/*z* (%) 255 (M^+^, 25), 198 (100), 170 (62). HRMS (ESI+) *m*/*z*, calcd for C_16_H_18_NO_2_^+^ [M + H]^+^ 256.1332, found 256.1332.

#### (E)-6-(1-Methyl-1H-indol-2-yl)-1-phenylhex-2-ene-1,6-dione (**11b**)

General procedure VI was followed using 2-(triphenylphosphoranylidene)acetophenone (209 mg, 0.55 mmol) obtaining **11b**, which was isolated by flash column chromatography (hexane/EtOAc, 5/1) as a brown oil (103 mg, 65%): *R*_*f*_ = 0.28 (hexane/EtOAc, 5/1). ^1^H NMR (300 MHz, CDCl_3_): δ 8.09–7.90 (m, 2H), 7.80–7.68 (m, 1H), 7.61–7.53 (m, 1H), 7.50–7.39 (m, 4H), 7.34 (s, 1H), 7.24–7.09 (m, 2H), 7.07–6.90 (m, 1H), 4.10 (s, 3H), 3.24 (t, *J* = 7.3 Hz, 2H), 2.94–2.72 (m, 2H). ^13^C{^1^H} NMR (75.4 MHz, CDCl_3_): δ 192.3 (C), 190.7 (C), 147.9 (CH), 140.2 (C), 137.9 (C), 134.5 (C), 132.8 (CH), 128.63 (2 × CH), 128.61 (2 × CH), 126.7 (CH), 126.2 (CH), 125.8 (C), 123.0 (CH), 120.9 (CH), 111.5 (CH), 110.5 (CH), 37.9 (CH_2_), 32.3 (CH_3_), 27.7 (CH_2_). LRMS (EI): *m*/*z* (%) 317 (M^+^, 10), 158 (58), 89 (100). HRMS (ESI+) *m*/*z*, calcd for C_21_H_20_NO_2_^+^ [M + H]^+^ 318.1489, found 318.1487.

### General Procedure VII for the Synthesis of Tetrahydrocarbazol-1-ones **12**

To a stirred solution of the corresponding dione **11** (0.3 mmol) in MeCN (1 mL) was added gold(III) chloride (4.5 mg, 0.015 mmol), and the resulting solution was stirred at rt for 16 h. The resulting mixture was quenched with water (2 mL) and extracted with DCM (3 × 5 mL). The combined organic layers were dried over anhydrous Na_2_SO_4_, filtered and concentrated in vacuo. The residue, when necessary, was purified by flash column chromatography using silica gel and mixtures of hexane/EtOAc as eluent to afford the corresponding tetrahydrocarbazolones **12a**,**b**.

#### 9-Methyl-4-(2-oxopropyl)-2,3,4,9-tetrahydro-1H-carbazol-1-one (**12a**)

General procedure VII was followed using (*E*)-1-(1-methyl-1*H*-indol-2-yl)hept-4-ene-1,6-dione (**11a**) (76.5 mg, 0.3 mmol) obtaining **12a**, which was obtained in pure form as a brown solid (61 mg, 80%): mp 129–131 °C. ^1^H NMR (300 MHz, CDCl_3_): δ 7.65 (d, *J* = 8.1 Hz, 1H), 7.47–7.31 (m, 2H), 7.22–7.09 (m, 1H), 4.07 (s, 3H), 3.92 (dd, *J* = 8.6, 4.1 Hz, 1H), 3.03–2.81 (m, 2H), 2.77–2.63 (m, 1H), 2.55 (dt, *J* = 17.3, 4.4 Hz, 1H), 2.44–2.33 (m, 1H), 2.19 (s, 3H), 2.13–2.04 (m, 1H). ^13^C{^1^H} NMR (75.4 MHz, CDCl_3_): δ 207.4 (C), 191.8 (C), 139.9 (C), 131.2 (C), 130.0 (C), 126.9 (CH), 123.9 (C), 121.6 (CH), 120.5 (CH), 110.6 (CH), 47.1 (CH_2_), 35.9 (CH_2_), 31.7 (CH_3_), 30.8 (CH), 29.0 (CH_2_), 27.7 (CH_3_). LRMS (EI): *m*/*z* (%) 255 (M^+^, 25), 198 (100), 170 (62). HRMS (ESI+) *m*/*z*, calcd for C_16_H_18_NO_2_^+^ [M + H]^+^ 256.1332, found 256.1334.

#### 9-Methyl-4-(2-oxo-2-phenylethyl)-2,3,4,9-tetrahydro-1H-carbazol-1-one (**12b**)

General procedure VII was followed using (*E*)-6-(1-methyl-1*H*-indol-2-yl)-1-phenylhex-2-ene-1,6-dione (**11b**) (95 mg, 0.3 mmol) obtaining **12b**, which was isolated by flash column chromatography (hexane/EtOAc, 4/1) as a brown solid (78 mg, 82%): mp 125–127 °C. *R*_*f*_ = 0.29 (hexane/EtOAc, 4/1). ^1^H NMR (300 MHz, CDCl_3_): δ 8.08–7.90 (m, 2H), 7.68 (dt, *J* = 8.1, 1.1 Hz, 1H), 7.61–7.55 (m, 1H), 7.53–7.33 (m, 4H), 7.14 (ddd, *J* = 8.0, 6.4, 1.6 Hz, 1H), 4.16–4.12 (m, 1H), 4.09 (s, 3H), 3.57–3.25 (m, 2H), 2.85–2.74 (m, 1H), 2.69–2.33 (m, 2H), 2.21–2.13 (m, 1H). ^13^C{^1^H} NMR (75.4 MHz, CDCl_3_): δ 198.6 (C), 191.9 (C), 139.9 (C), 136.9 (C), 133.4 (CH), 131.4 (C), 130.0 (C), 128.8 (2 × CH), 128.1 (2 × CH), 126.8 (CH), 123.9 (C), 121.5 (CH), 120.4 (CH), 110.5 (CH), 41.8 (CH_2_), 35.9 (CH_2_), 31.6 (CH_3_), 28.7 (CH_2_), 28.0 (CH). LRMS (EI): *m*/*z* (%) 317 (M^+^, 33), 198 (100), 170 (27). HRMS (ESI+) *m*/*z*, calcd for C_21_H_20_NO_2_^+^ [M + H]^+^ 318.1489, found 318.1492.

### Synthesis of Dihydrocarbazole **13**

To a solution of 9-methyl-4-(2,4,6-trimethoxyphenyl)-2,3,4,9-tetrahydro-1*H*-carbazol-1-one (**10a**) (184 mg, 0.5 mmol) in anhydrous THF (1 mL) was added EtMgCl (0.33 mL, 0.65 mmol, 2 M solution in THF) at 0 °C, and the resulting mixture was stirred at rt for 16 h. Then, the reaction was quenched with aq. NH_4_Cl (2 mL). THF was removed under reduced pressure, and the aqueous layer was extracted with Et_2_O (3 × 5 mL). The combined organic layers were dried over anhydrous Na_2_SO_4_, filtered and concentrated in vacuo. The residue was purified by flash column chromatography using a 3/1 mixture of hexane/EtOAc as eluent affording **13** as a yellowish solid (68 mg, 36%).

#### 1-Ethyl-9-methyl-4-(2,4,6-trimethoxyphenyl)-4,9-dihydro-3H-carbazole (**13**)

Mp 96–98 °C. *R*_*f*_ = 0.34 (hexane/EtOAc, 3/1). ^1^H NMR (300 MHz, CDCl_3_): δ 7.43–7.25 (m, 1H), 7.13 (ddd, *J* = 8.2, 6.9, 1.2 Hz, 1H), 6.93–6.78 (m, 1H), 6.75 (d, *J* = 7.9 Hz, 1H), 6.23 (s, 1H), 6.06–5.80 (m, 1H), 5.15–4.80 (m, 1H), 3.88 (s, 6H), 3.61 (bs, 6H), 3.21–2.91 (m, 1H), 2.39–2.32 (m, 2H), 2.16–2.12 (m, 1H), 1.99 (d, *J* = 6.9 Hz, 3H). ^13^C{^1^H} NMR (75.4 MHz, CDCl_3_): δ 159.7 (C), 159.5 (C), 139.2 (2 × C), 135.6 (C), 131.3 (C), 126.7 (C), 121.1 (CH), 119.2 (CH), 118.4 (CH), 117.4 (CH), 116.5 (C), 114.4 (C), 108.7 (CH), 91.7 (2 × CH), 56.1 (2 × CH_3_), 55.3 (CH_3_), 32.7 (CH), 30.5 (CH_3_), 30.4 (CH_2_), 27.6 (CH_2_), 13.9 (CH_3_). LRMS (EI): *m*/*z* (%) 378 (M^+^, 100), 349 (17), 335 (14). HRMS (ESI+) *m*/*z*, calcd for C_24_H_28_NO_3_^+^ [M + H]^+^ 378.2064, found 378.2063.

### Synthesis of Tetrahydrocarbazol-1-one **14**

To a stirred solution of 9-methyl-4-(5-methylfuran-2-yl)-2,3,4,9-tetrahydro-1*H*-carbazol-1-one (**10f**) (88.4 mg, 0.3 mmol) in anhydrous THF (0.6 mL) was added LiHDMS (0.33 mL, 0.33 mmol, 1 M in THF) at −78 °C, and the resulted solution was stirred at 0 °C for 1 h. Next, methyl iodide (47 mg, 0.33 mmol) was added at −78 °C and stirred overnight at rt. Then, the mixture was quenched with aq. NH_4_Cl (5 mL). THF was removed under reduced pressure, and the aqueous layer was extracted with EtOAc (3 × 15 mL). The combined organic layers were dried over anhydrous Na_2_SO_4_, filtered, and concentrated in vacuo. The residue was purified by flash column chromatography by using a 7/1 mixture of hexane/EtOAc as eluent, affording **14** as a ca. 3/1 mixture of diastereoisomers.

#### 2,9-Dimethyl-4-(5-methylfuran-2-yl)-2,3,4,9-tetrahydro-1H-carbazol-1-one (**14**)

Obtained and isolated as a c.a. 3/1 mixture of diastereoisomers. Yellow oil (40 mg, 45%): *R*_*f*_ = 0.30 (hexane/EtOAc, 7/1). Data for both diastereoisomers: ^1^H NMR (300 MHz, CDCl_3_): δ 7.53 (dt, *J* = 8.1, 1.1 Hz, 1H, major diast.), 7.44–7.34 (m, 2H, major+minor diast.), 7.19–7.08 (m, 1H, major+minor diast.), 7.01 (dt, *J* = 8.1, 3.9 Hz, 1H, minor diast.), 6.11 (d, *J* = 3.1 Hz, 1H minor diast.), 6.03–5.95 (m, 1H minor diast.), 5.87–5.82 (m, 1H major diast.), 5.71 (dd, *J* = 3.1, 0.9 Hz, 1H major diast.), 4.64–4.53 (m, 1H major+minor diast.), 4.13 (s, 3H major+minor diast.), 2.98–2.83 (m, 1H major diast.), 2.64–2.44 (m, 1H, major+minor diast.), 2.40–2.31 (m, 1H, major+minor diast.), 2.29 (s, 3H, major+minor diast.), 2.19 (dd, *J* = 13.4, 4.7 Hz, 1H, minor diast.), 1.41–1.14 (m, 3H, major+minor diast.). ^13^C{^1^H} NMR (75.4 MHz, CDCl_3_): δ 197.4 (C), 195.1 (C), 154.7 (C), 153.7 (C), 151.3 (C), 151.2 (C), 140.0 (C), 130.2 (C), 127.1 (C), 127.0 (C), 126.5 (CH), 126.3 (CH), 124.6 (C), 122.3 (CH), 121.8 (CH), 120.4 (CH), 120.3 (CH), 110.4 (CH), 110.3 (CH), 107.5 (CH), 107.3 (CH), 106.2 (CH), 106.1 (CH), 45.8 (CH_2_), 43.7 (CH), 39.7 (CH), 38.3 (CH_2_), 32.2 (CH), 32.0 (CH_3_), 31.7 (CH_3_), 25.3 (CH), 24.6 (CH_3_), 15.2 (CH_3_), 13.8 (CH_3_), 13.7 (CH_3_). LRMS (EI): *m*/*z* (%) 293 (M^+^, 100), 264 (20), 250 (57). HRMS (ESI+) *m*/*z*, calcd for C_19_H_20_NO_2_^+^ [M + H]^+^ 294.1489, found 294.1492.

### Synthesis of Alcohol **15**

To a solution of 4-(5-bromo-1*H*-indol-3-yl)-9-methyl-2,3,4,9-tetrahydro-1*H*-carbazol-1-one (**8f**) (69.1 mg, 0.25 mmol) in MeOH (2 mL) was added NaBH_4_ (14.2 mg, 0.375 mmol), and the resulting mixture was stirred at rt for 2 h. Then, most of MeOH was evaporated, and the residue was diluted with water (3 mL) and extracted with Et_2_O (3 × 5 mL). The combined organic layers were dried over anhydrous Na_2_SO_4_, filtered, and concentrated in vacuo. The residue was purified by flash column chromatography in deactivated silica using a 1/2 mixture of hexane/EtOAc as eluent, affording **15** as a ca. 1.5/1 mixture of diastereoisomers.

#### 4-(5-Bromo-1H-indol-3-yl)-9-methyl-2,3,4,9-tetrahydro-1H-carbazol-1-ol (**15**)

Obtained and isolated as a c.a. 1.5/1 mixture of diastereoisomers. Brown oil (49 mg, 50%): *R*_*f*_ = 0.36 (hexane/EtOAc, 1/2). Data for both diastereoisomers: ^1^H NMR (300 MHz, (CD_3_)_2_CO): δ 10.20 (bs, 1H, major diast.), 10.05 (bs, 1H, minor diast.), 7.86 (d, *J* = 1.9 Hz, 1H, minor diast.), 7.74 (d, *J* = 1.9 Hz, 1H, major diast.), 7.51–7.32 (m, 3H, major+minor diast.), 7.26–7.00 (m, 3H, major+minor diast.), 6.95–6.82 (m, 1H, major+minor diast.), 6.72 (ddd, *J* = 8.0, 6.9, 1.0 Hz, 1H, major diast.), 6.56 (d, *J* = 2.3 Hz, 1H, minor diast.), 5.18–4.95 (m, 1H, major+minor diast.), 4.64 (dd, *J* = 5.6, 2.7 Hz, 1H, minor diast.), 4.47–4.33 (m, 2H, major diast.), 4.19 (d, *J* = 6.9 Hz, 1H, minor diast.), 3.87 (d, *J* = 1.7 Hz, 3H, major+minor diast.), 2.90 (bs, 1H, major+minor diast.), 2.60–2.29 (m, 1H, major diast.), 2.20–2.10 (m, 2H, major+minor diast.). ^13^C{^1^H} NMR (75.4 MHz, (CD_3_)_2_CO): δ 138.5 (C), 138.1 (C), 136.8 (C), 136.6 (C), 129.7 (C), 129.6 (C), 127.2 (C), 127.1 (C), 125.9 (C), 125.4 (CH), 124.6 (CH), 124.5 (CH), 122.4 (CH), 122.2 (CH), 122.0 (CH), 121.9 (CH), 120.7 (CH), 120.13 (C), 120.07 (C), 119.4 (CH), 119.2 (CH), 118.9 (CH), 114.1 (CH), 114.04 (CH), 114.00 (C), 112.3 (C), 112.6 (C), 109.8 (CH), 109.7 (CH), 61.9 (CH), 61.5 (CH), 33.4 (CH_2_), 32.60 (CH), 30.7 (CH_2_), 30.5 (CH), 30.0 (CH_3_) 29.9 (CH_3_), 28.5 (CH_2_) 26.5 (CH_2_). Three aromatic CH are missing due to overlapping of signals. LRMS (EI): *m*/*z* (%) could not be recorded. HRMS (ESI+) *m*/*z*, calcd for C_21_H_19_BrN_2_NaO^+^ [M + Na]^+^ 418.0605, found 418.0604.

## Data Availability

The data underlying this study are available in the published article and its Supporting Information.

## Supporting Information Available

Copies of ^1^H and ^13^C{^1^H} NMR spectra for all new compounds (PDF)
FAIR data, including the primary NMR FID files, for compounds **1a**, **4a**, **4b**, **5**, **6a**–**6k**, **7i**, **8a**–**8k**, **9a**, **10a**–**10n**, **11a**, **11b**, **12a**, **12b**, **13**, **14**, **15**, **S1**, **S3** (ZIP)
