# Supplementary material for: Brønsted Acid-Catalyzed Synthesis of 4-Functionalized Tetrahydrocarbazol-1-ones from 1,4-Dicarbonylindole Derivatives
Source: J Org Chem. 2023 Dec 12;89(1):505–20. doi: 10.1021/acs.joc.3c02248 (PMC10777410; doi:10.1021/acs.joc.3c02248)
Supplement: Supplementary file 1 — jo3c02248_si_001.pdf [file jo3c02248_si_001.pdf]

Supporting Information

Brønsted Acid-Catalyzed Synthesis of 4-Functionalized Tetrahydrocarbazol-1-ones from 1,4-Dicarbonylindole Derivatives

Sara Gómez-Gil, Marta Solas, Samuel Suárez-Pantiga, and Roberto Sanz*

†Área de Química Orgánica, Departamento de Química, Facultad de Ciencias, Universidad de Burgos, Pza. Misael Bañuelos, s/n, 09001-Burgos, Spain. E-mail: rsd@ubu.es

Table of Contents

^{1}H and ^{13}C NMR SPECTRA S1
Single Crystal X-ray Diffraction Analysis of **8a** S106

^{1}H and ^{13}C NMR SPECTRA

^{1}H-NMR (300 MHz, $CDCl_3$)

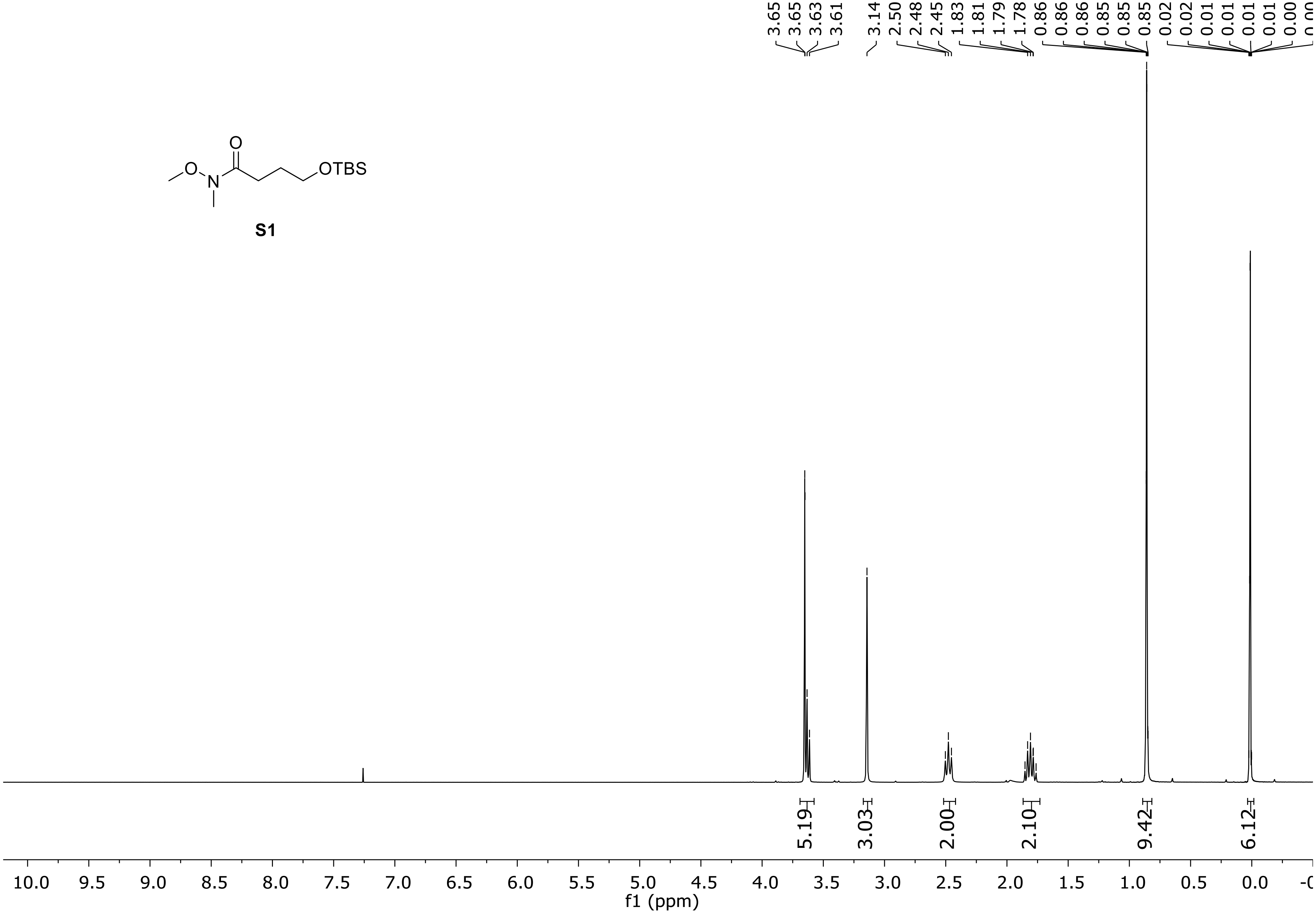

$^{13}C\{^1H\}$-NMR (75.4 MHz, $CDCl_3$)

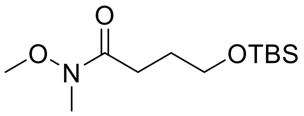

S1

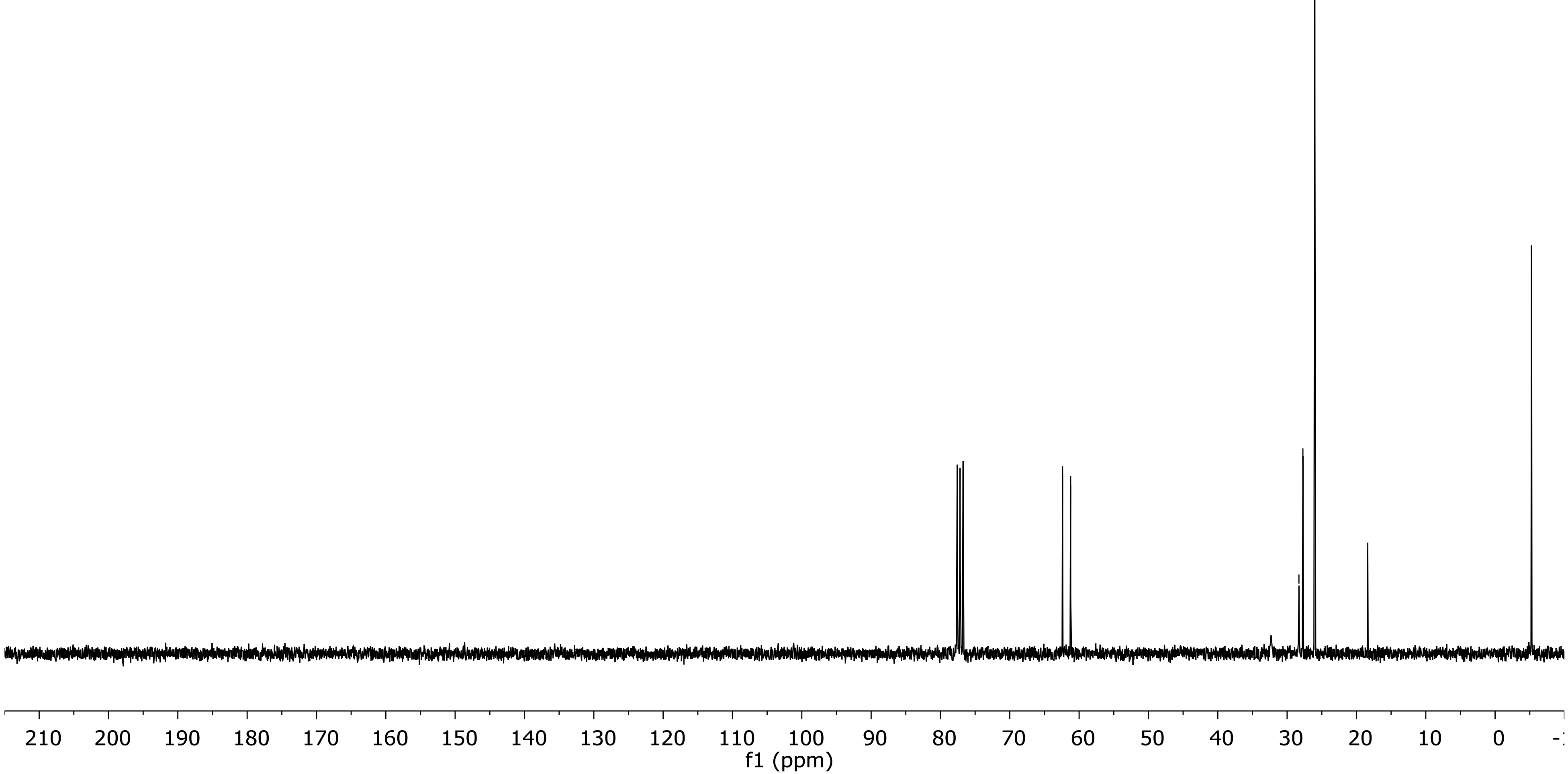

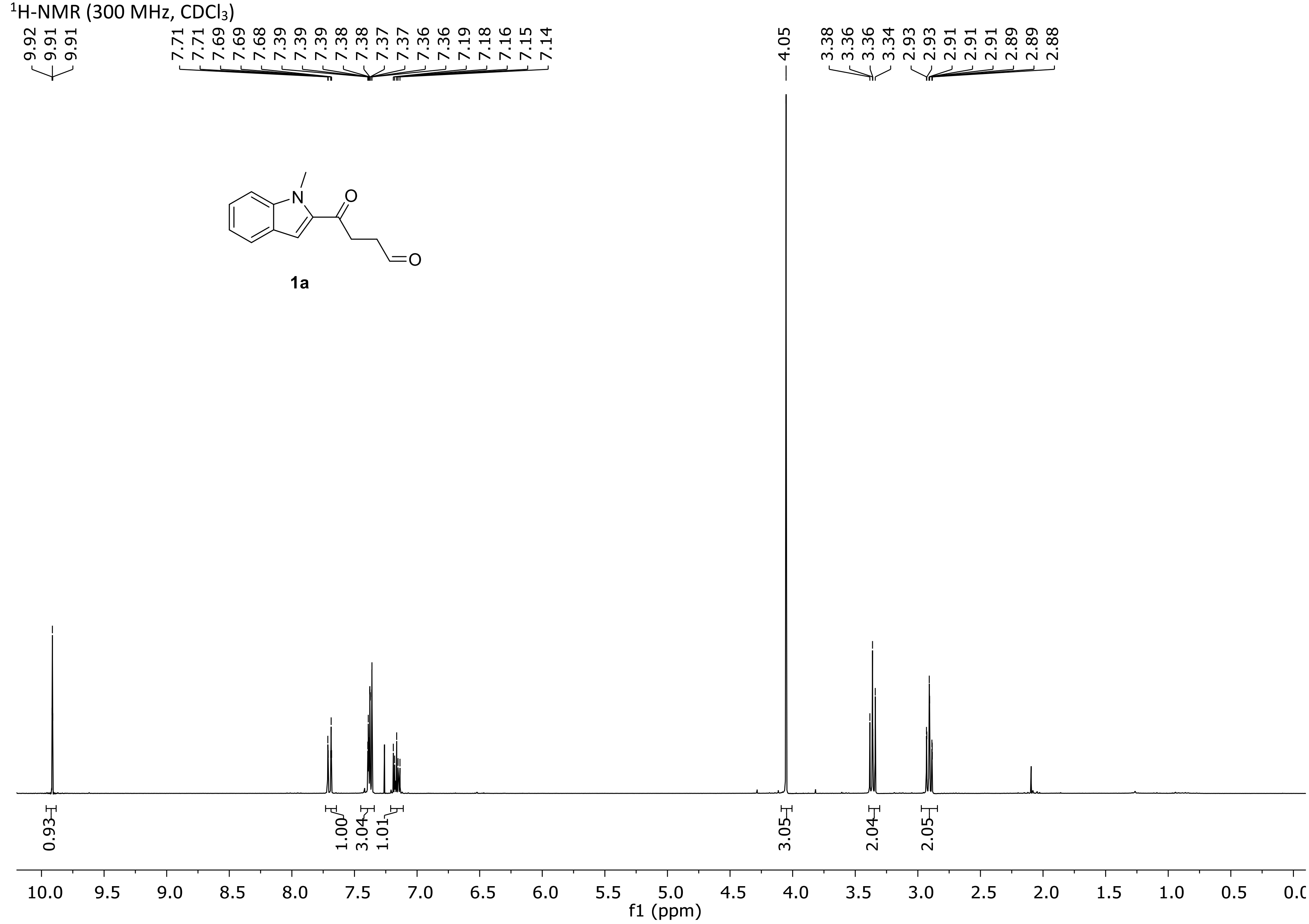
^{1}H-NMR (300 MHz, CDCl3)
9.92 9.91 9.91
7.71 7.71 7.69 7.69 7.68 7.39 7.39 7.39 7.38 7.38 7.37 7.37 7.36 7.36 7.19 7.18 7.16 7.15 7.14
4.05
3.38 3.36 3.36 3.34 2.93 2.93 2.91 2.91 2.91 2.89 2.89 2.88
O
N
O
1a
0.93
1.00
3.04
1.01
3.05
2.04
2.05
10.0 9.5 9.0 8.5 8.0 7.5 7.0 6.5 6.0 5.5 5.0 4.5 4.0 3.5 3.0 2.5 2.0 1.5 1.0 0.5 0.0
f1 (ppm)

$^{13}C\{^{1}H\}$-NMR (75.4 MHz, $CDCl_3$)

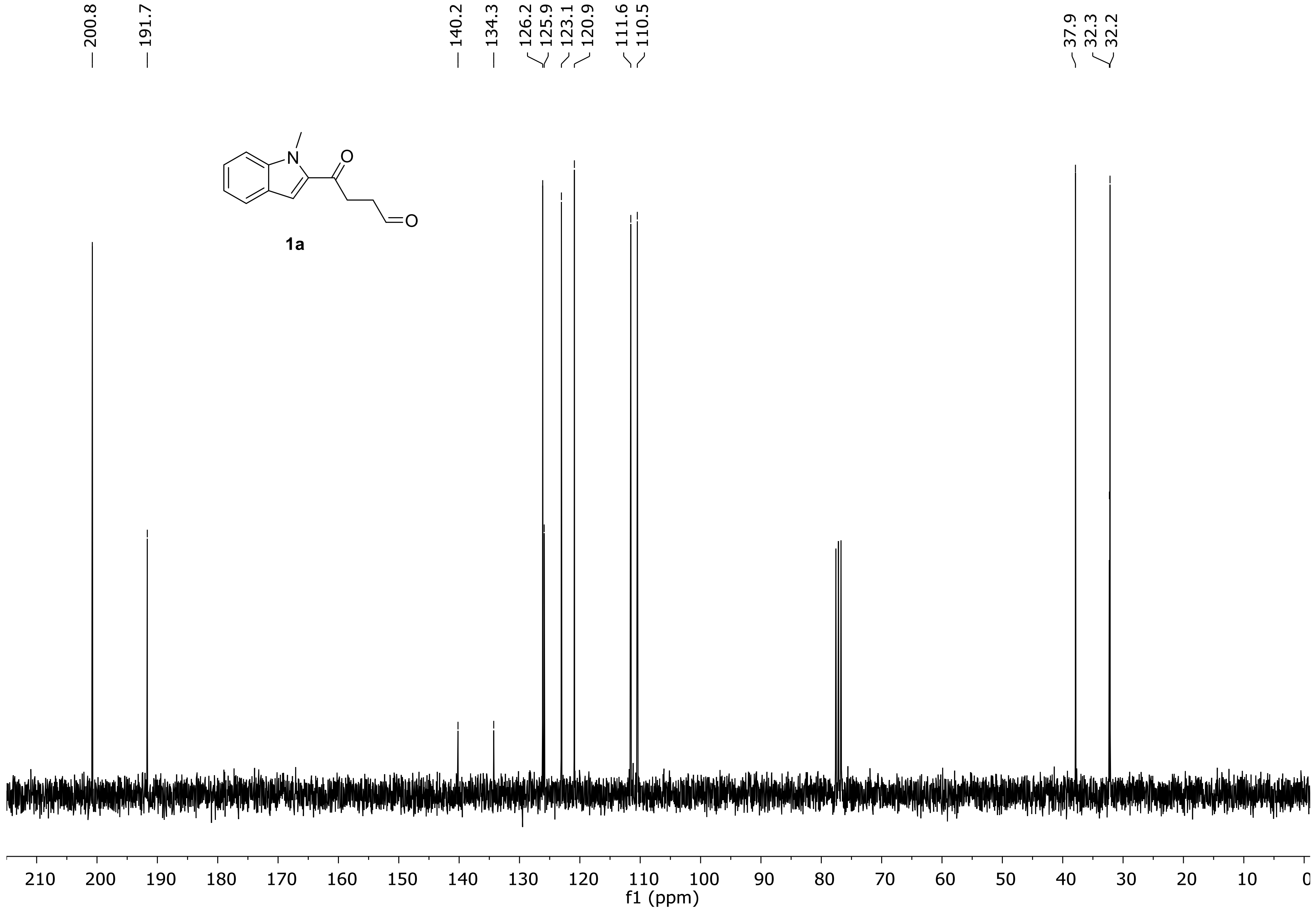

^{1}H-NMR (300 MHz, $CDCl_3$)

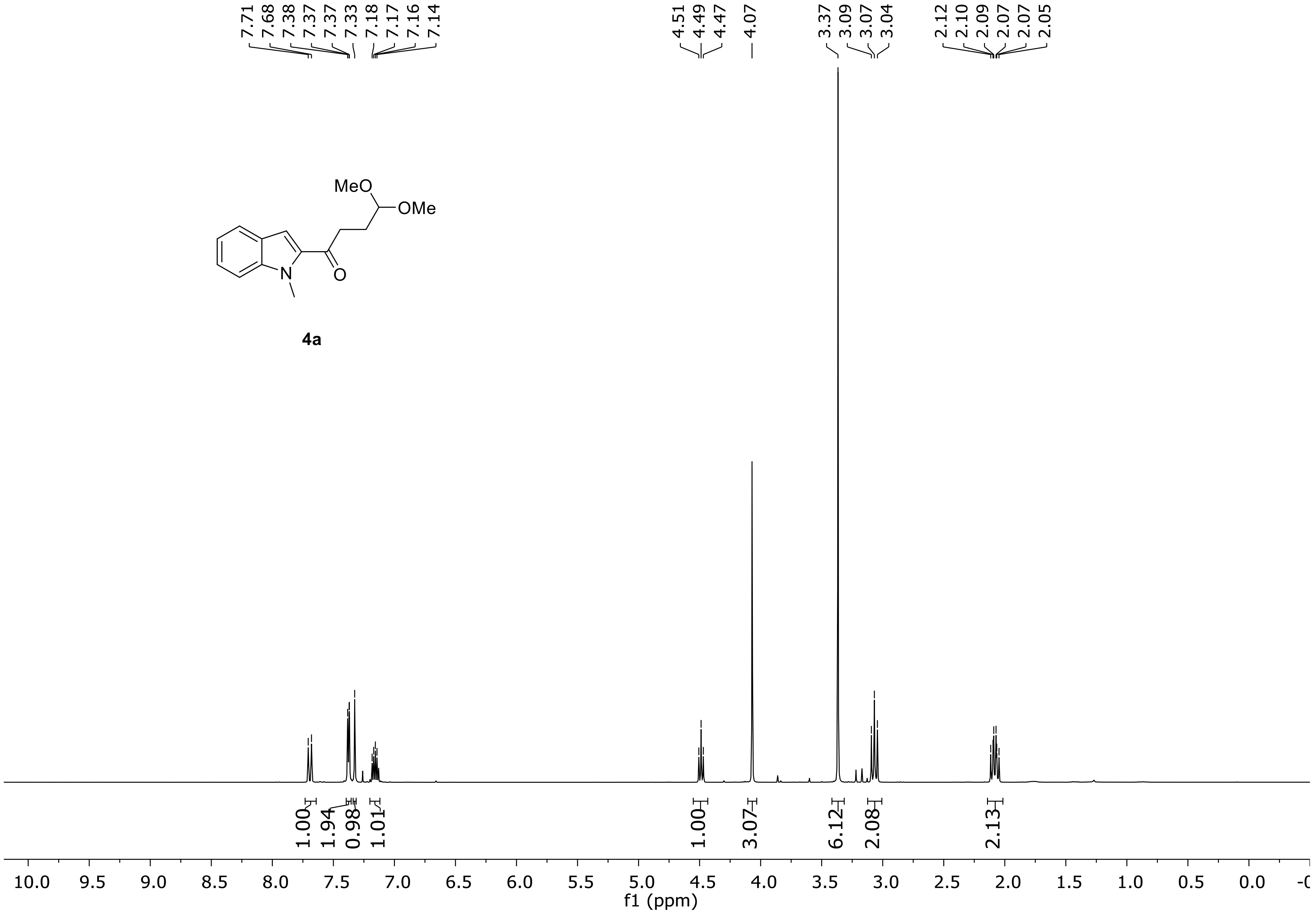

$^{13}C\{^{1}H\}$-NMR (75.4 MHz, $CDCl_3$)

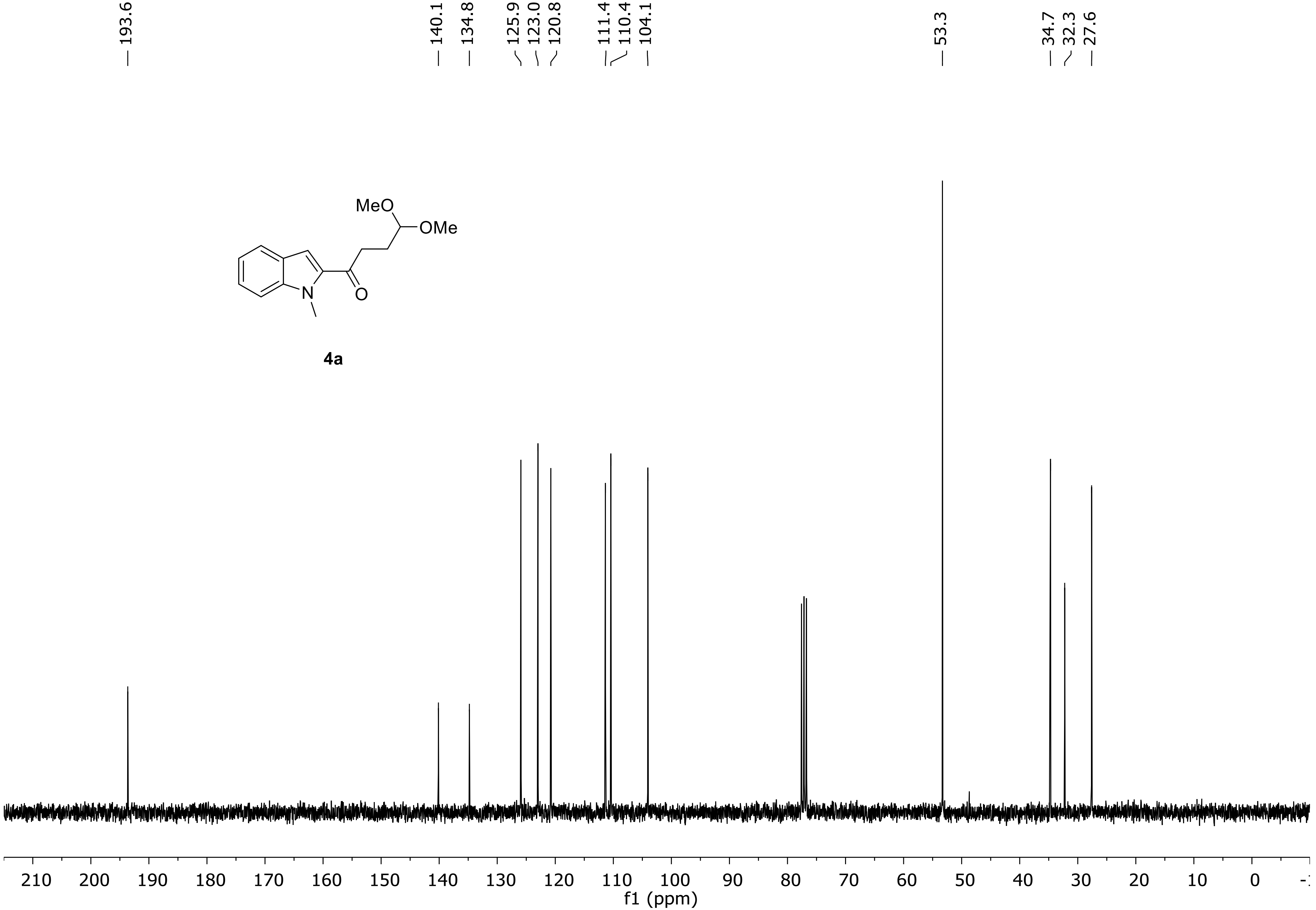

^{1}H-NMR (300 MHz, $CDCl_3$)

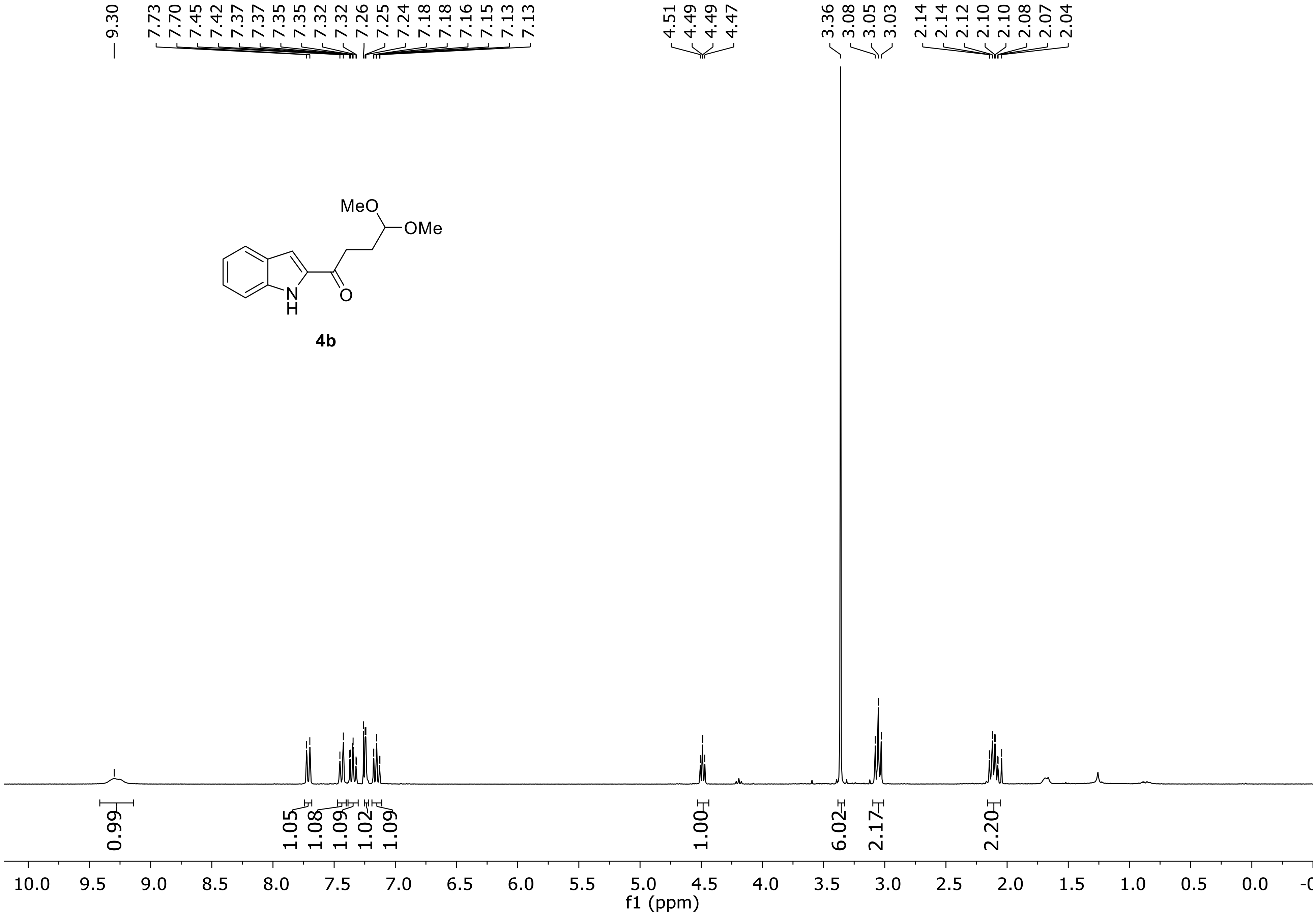

$^{13}C\{^{1}H\}$-NMR (75.4 MHz, $CDCl_3$)

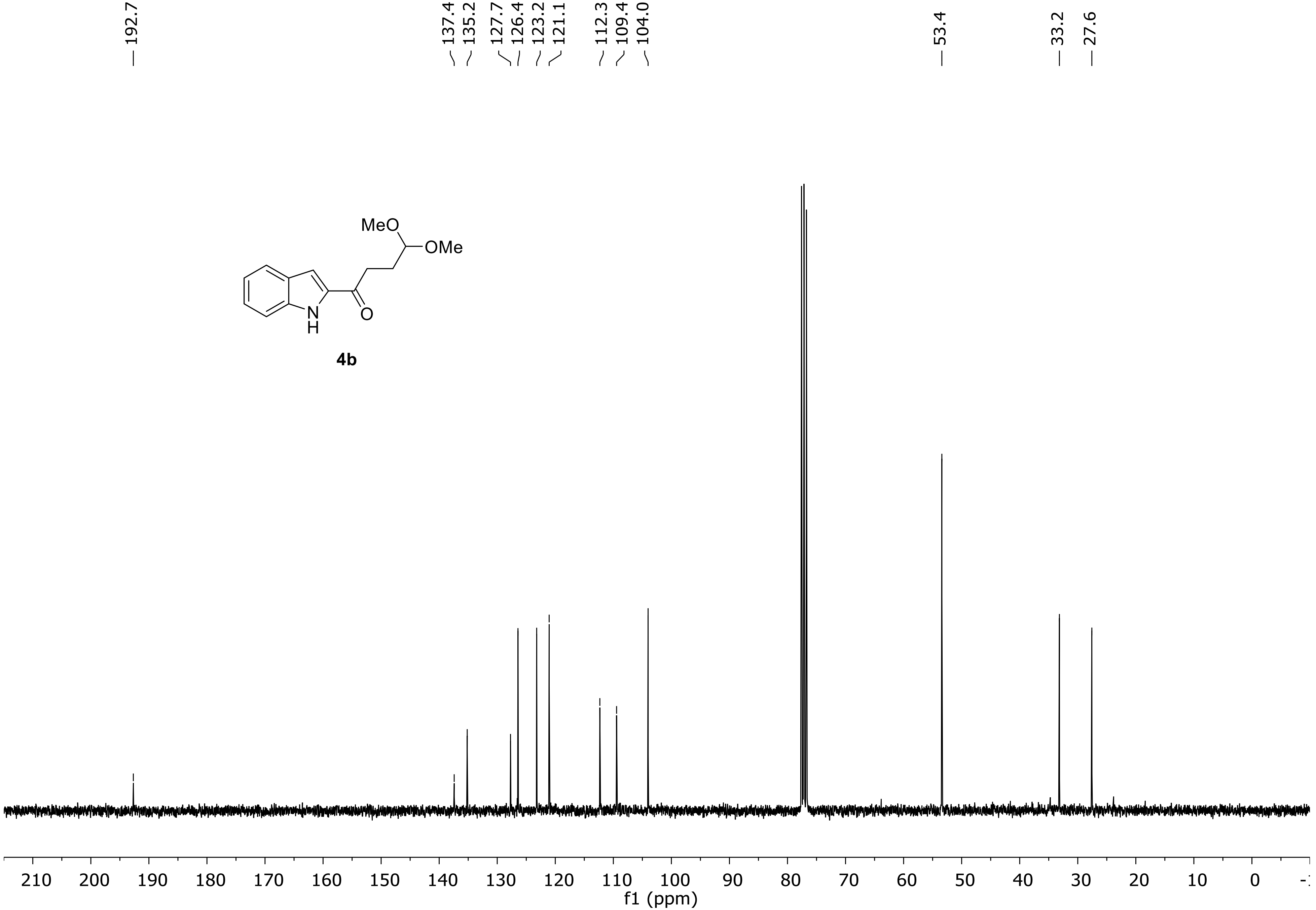

^{1}H-NMR (300 MHz, $CDCl_3$)

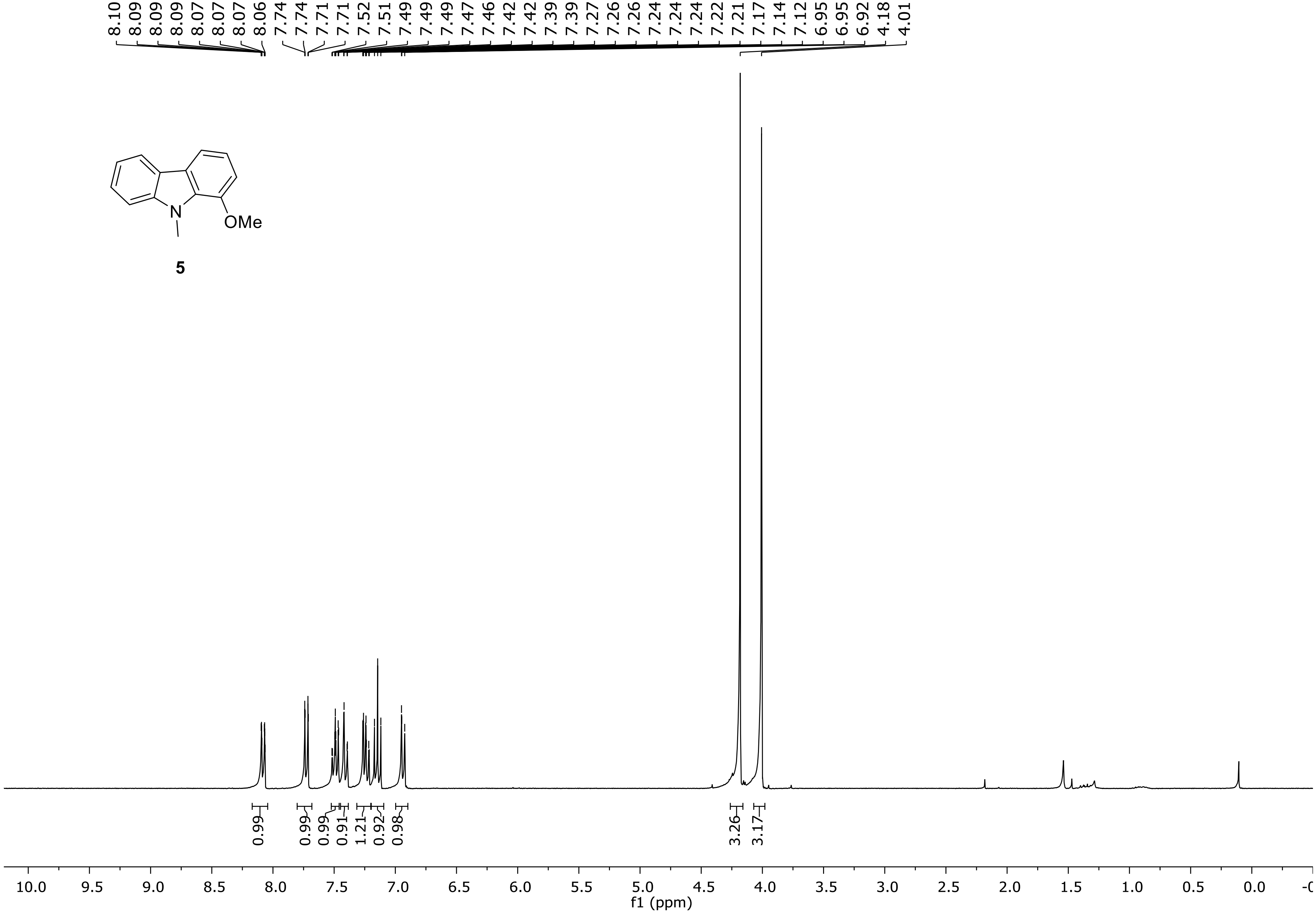

5

$^{13}C\{^{1}H\}$-NMR (75.4 MHz, $CDCl_3$)

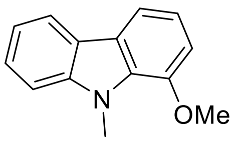

5

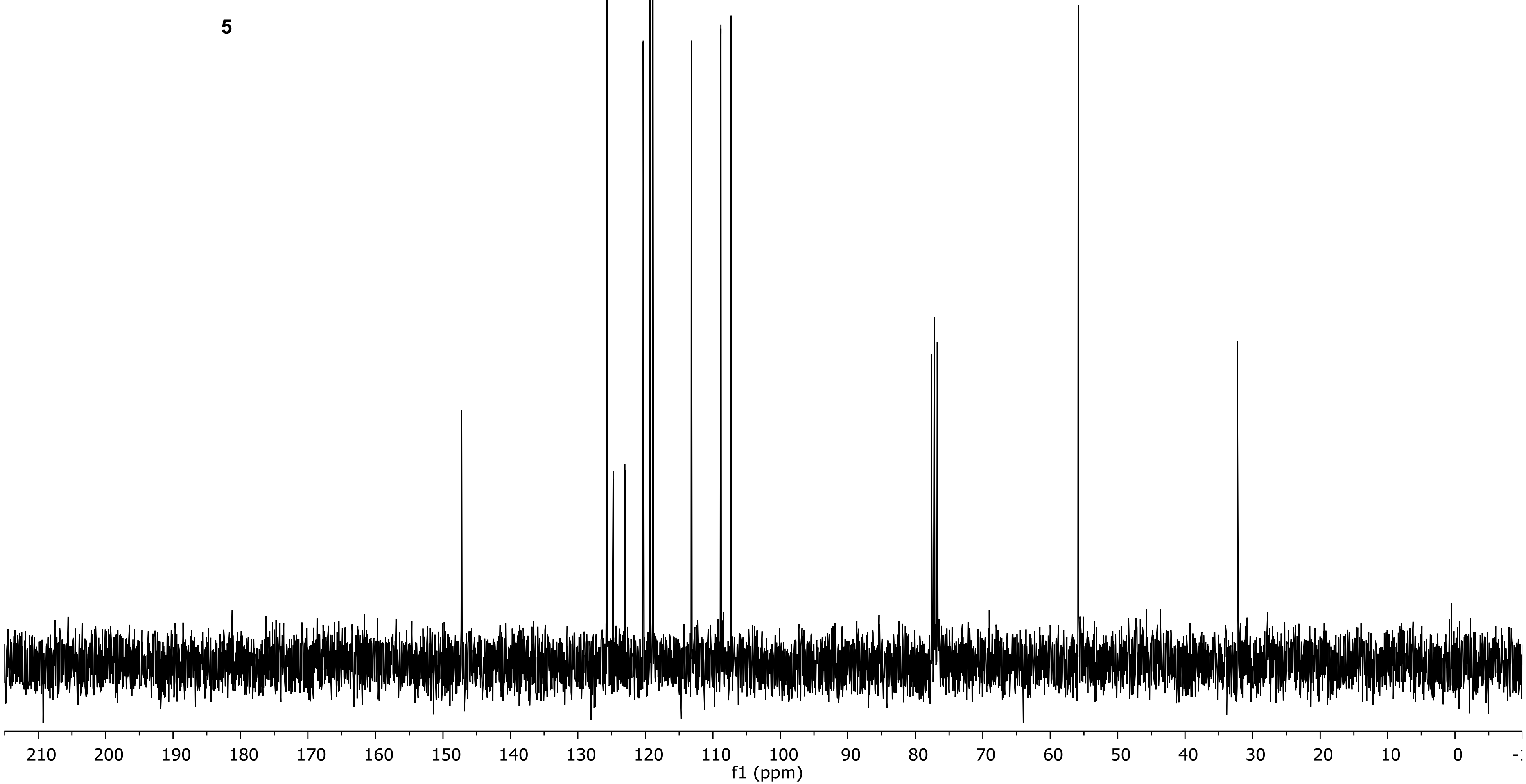

^{1}H-NMR (300 MHz, $CDCl_3$)

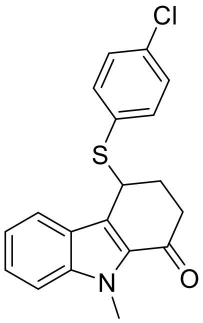

6a

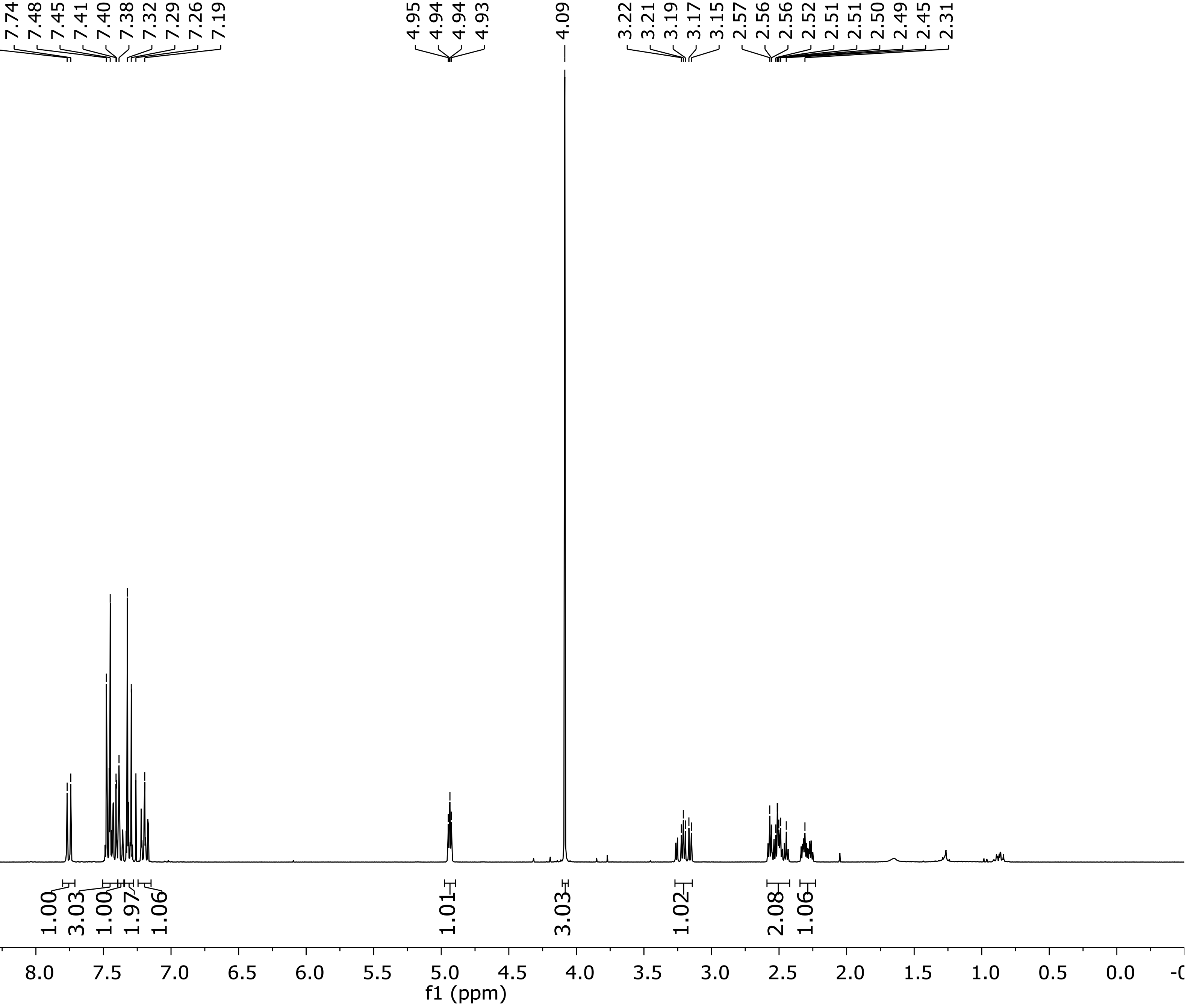

$^{13}C\{^{1}H\}$-NMR (75.4 MHz, $CDCl_3$)

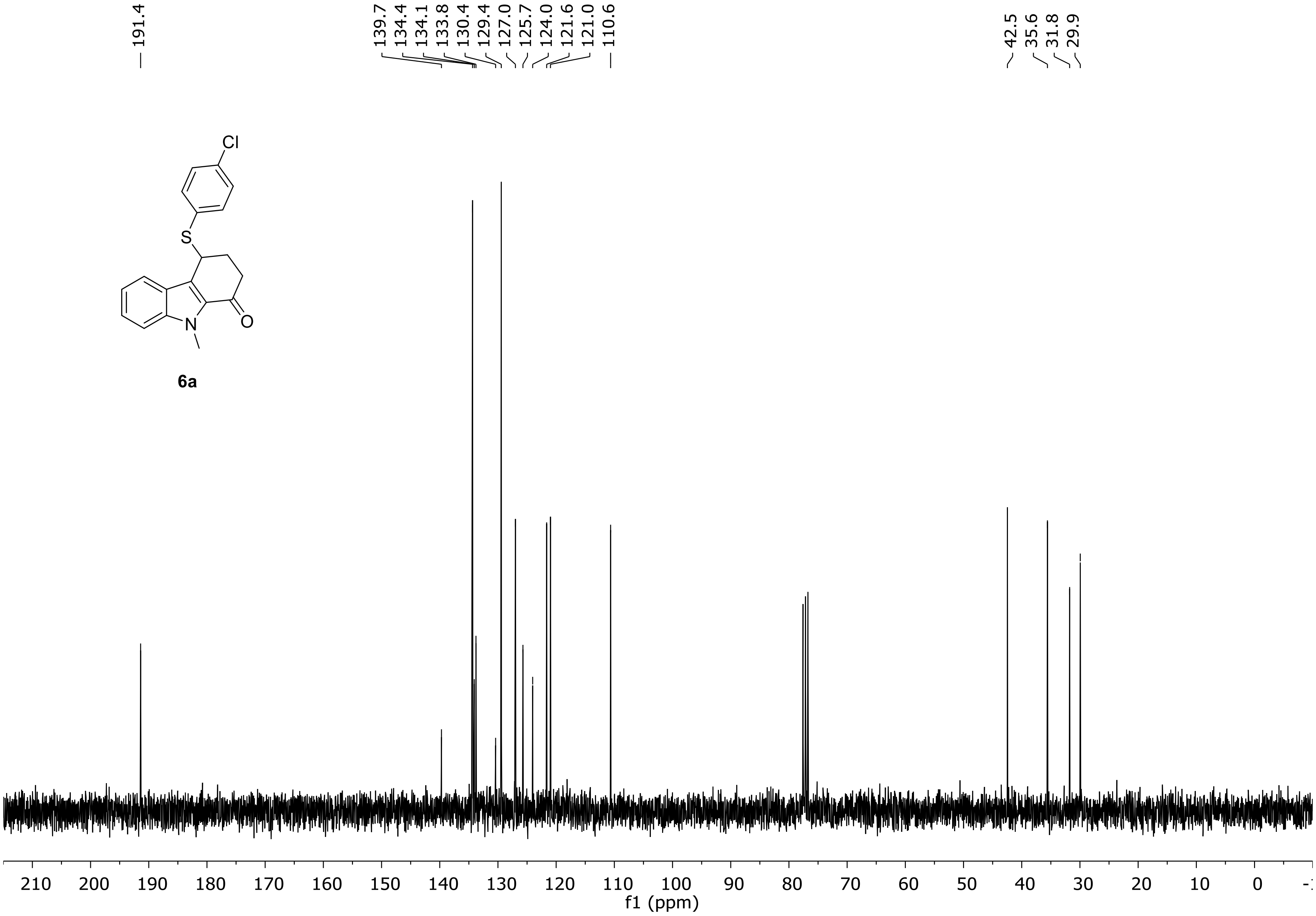

^{1}H-NMR (300 MHz, $CDCl_3$)

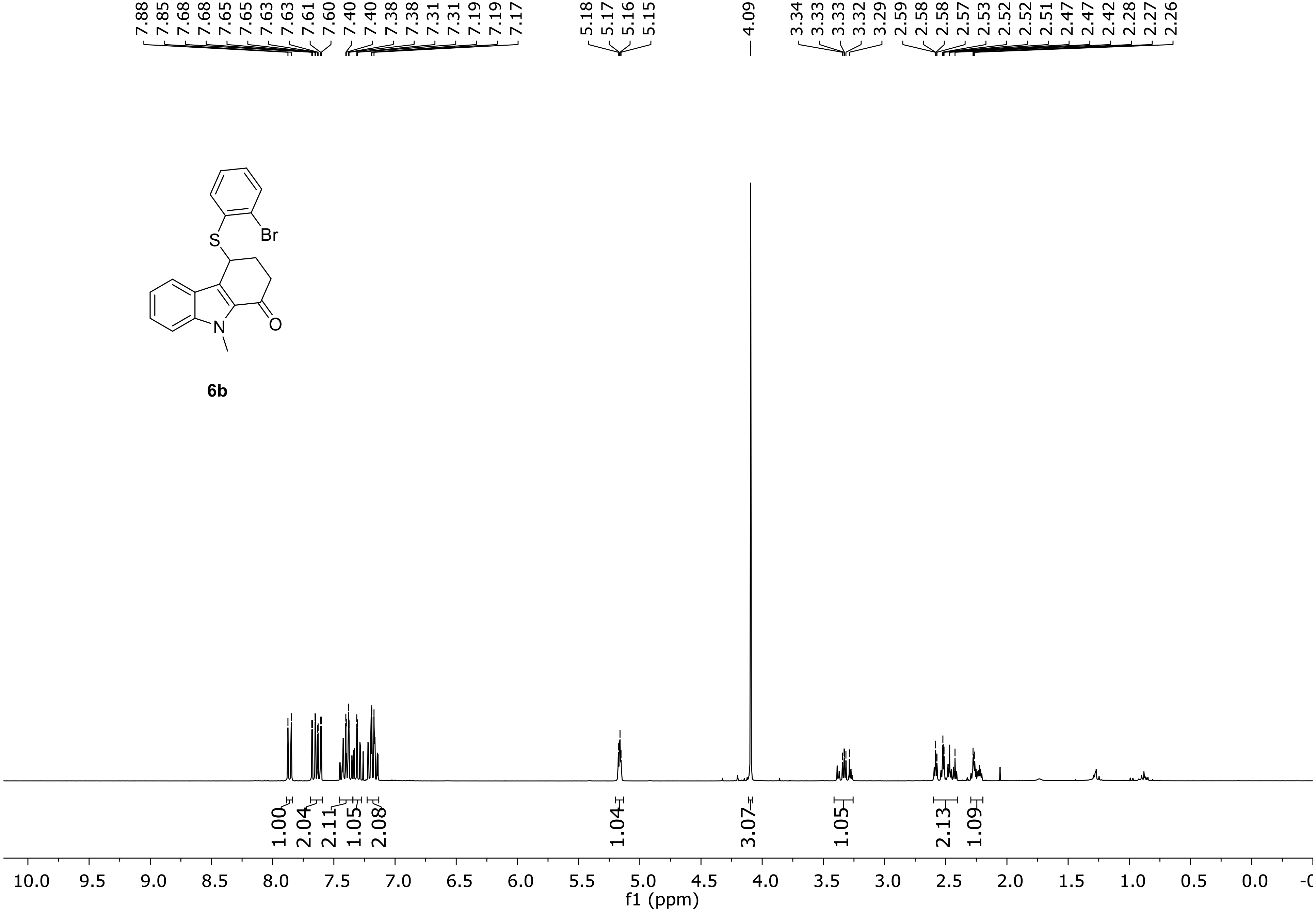

$^{13}C\{^{1}H\}$-NMR (75.4 MHz, $CDCl_3$)

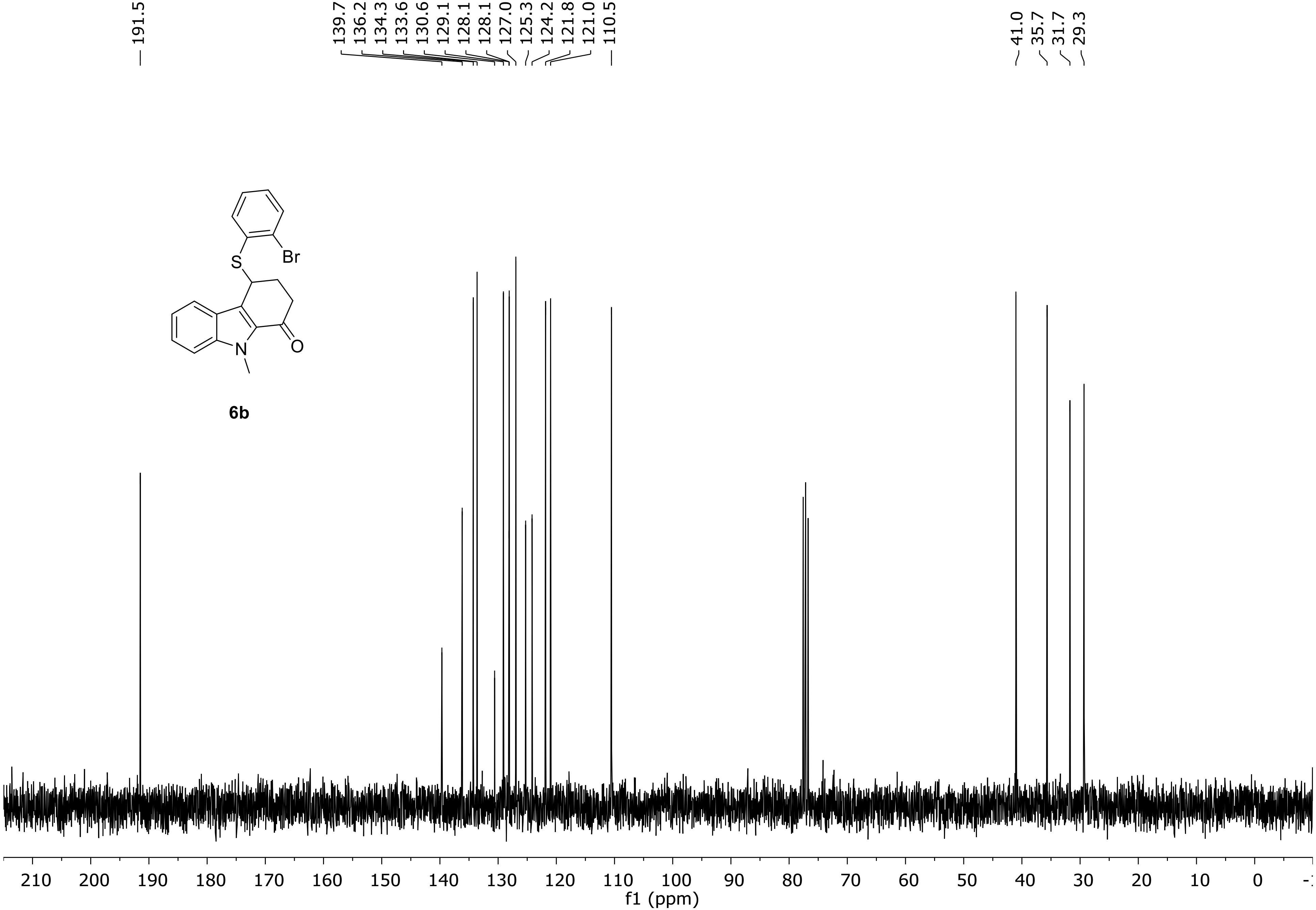

^{1}H-NMR (300 MHz, $CDCl_3$)

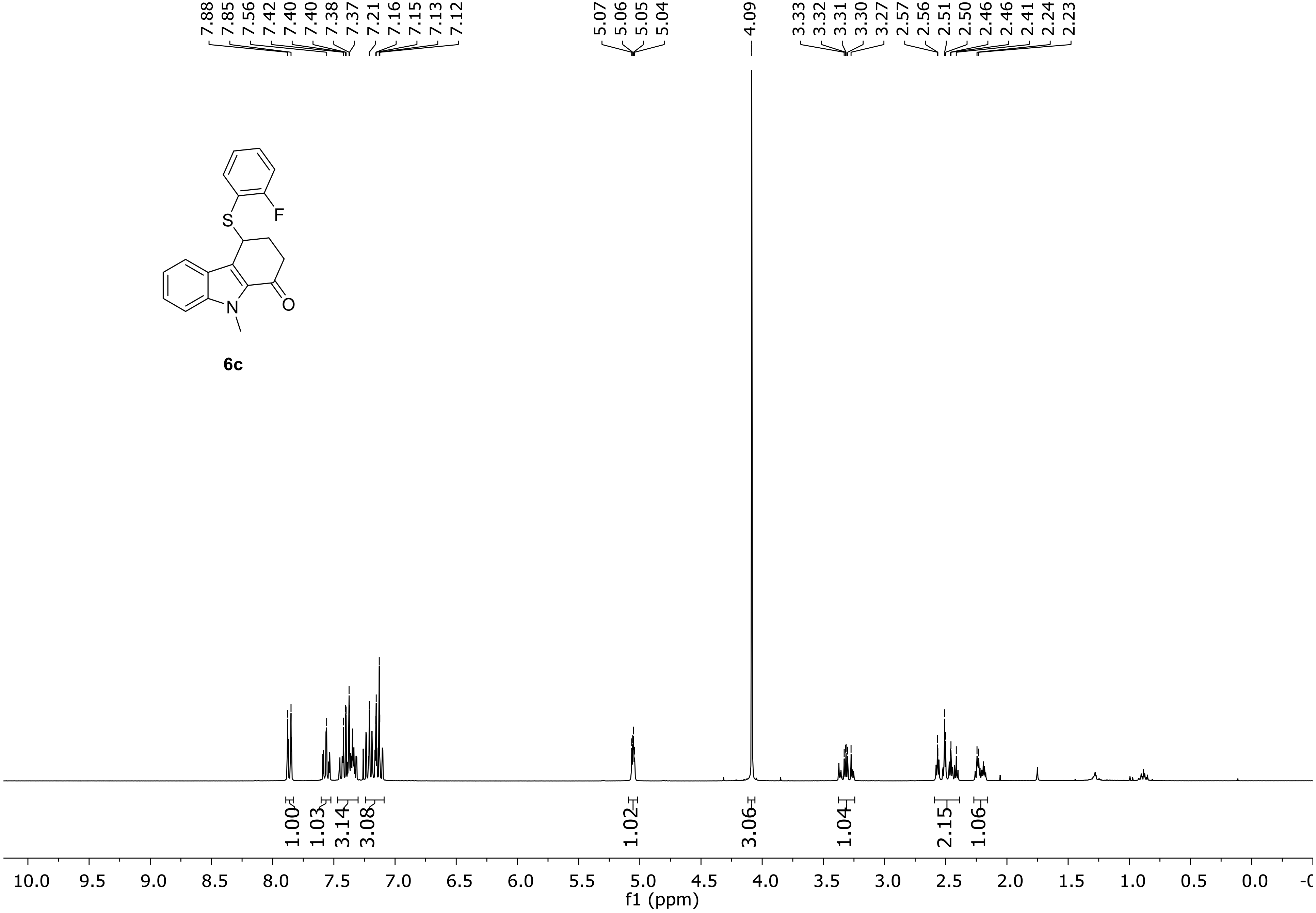

$^{13}C\{^1H\}$-NMR (75.4 MHz, $CDCl_3$)

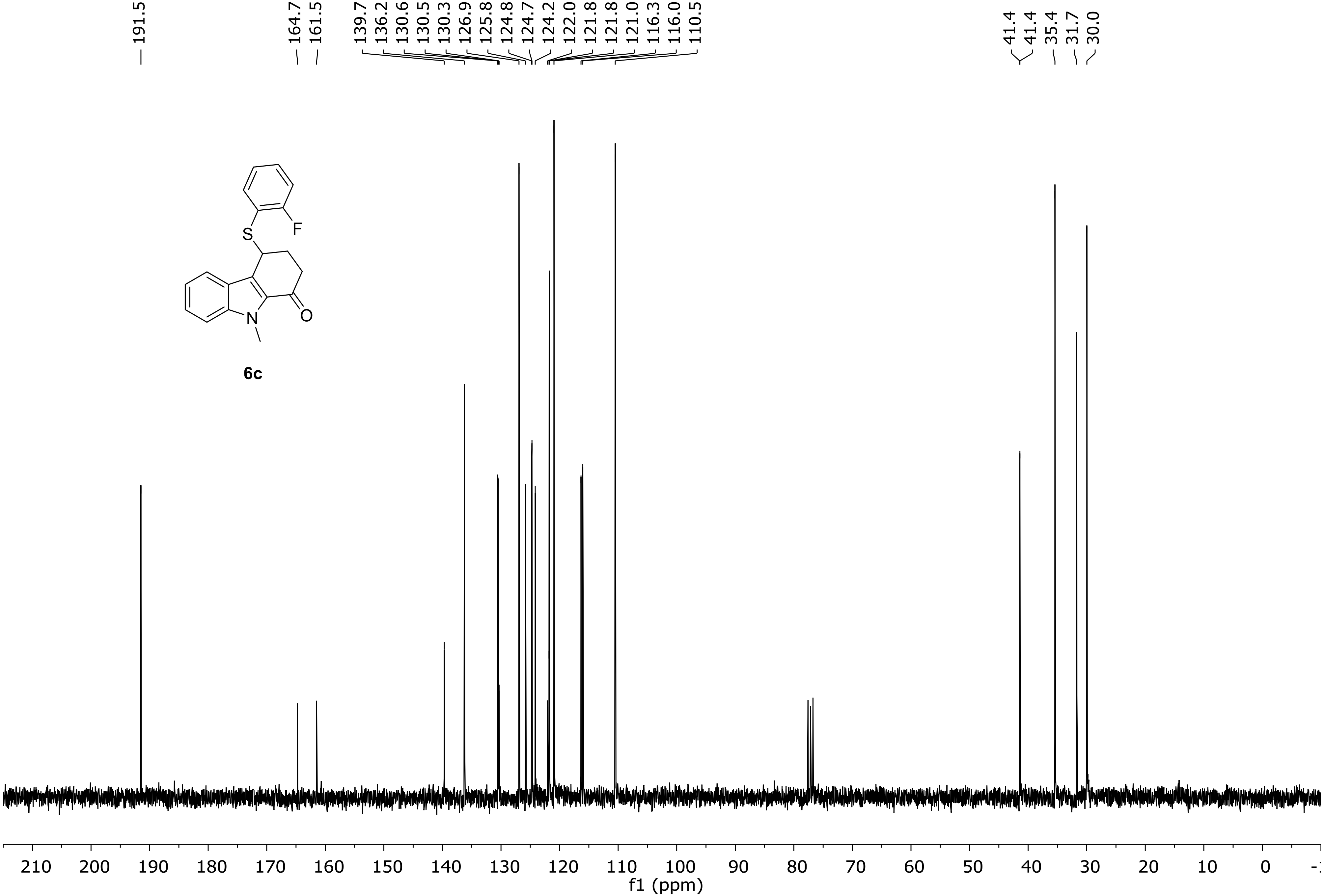

^{19}F-NMR (282 MHz, $CDCl_3$)

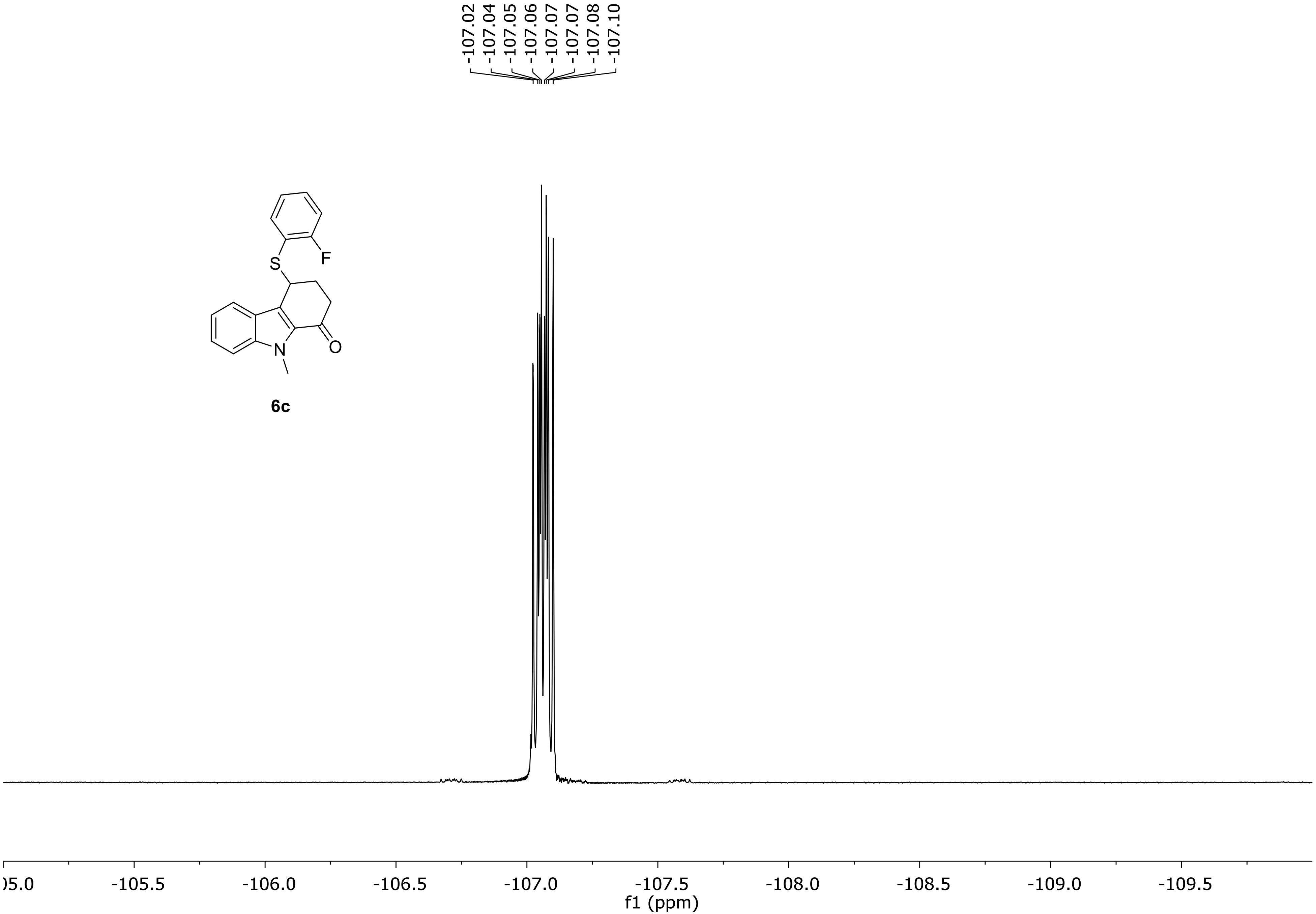

^{1}H-NMR (300 MHz, $CDCl_3$)

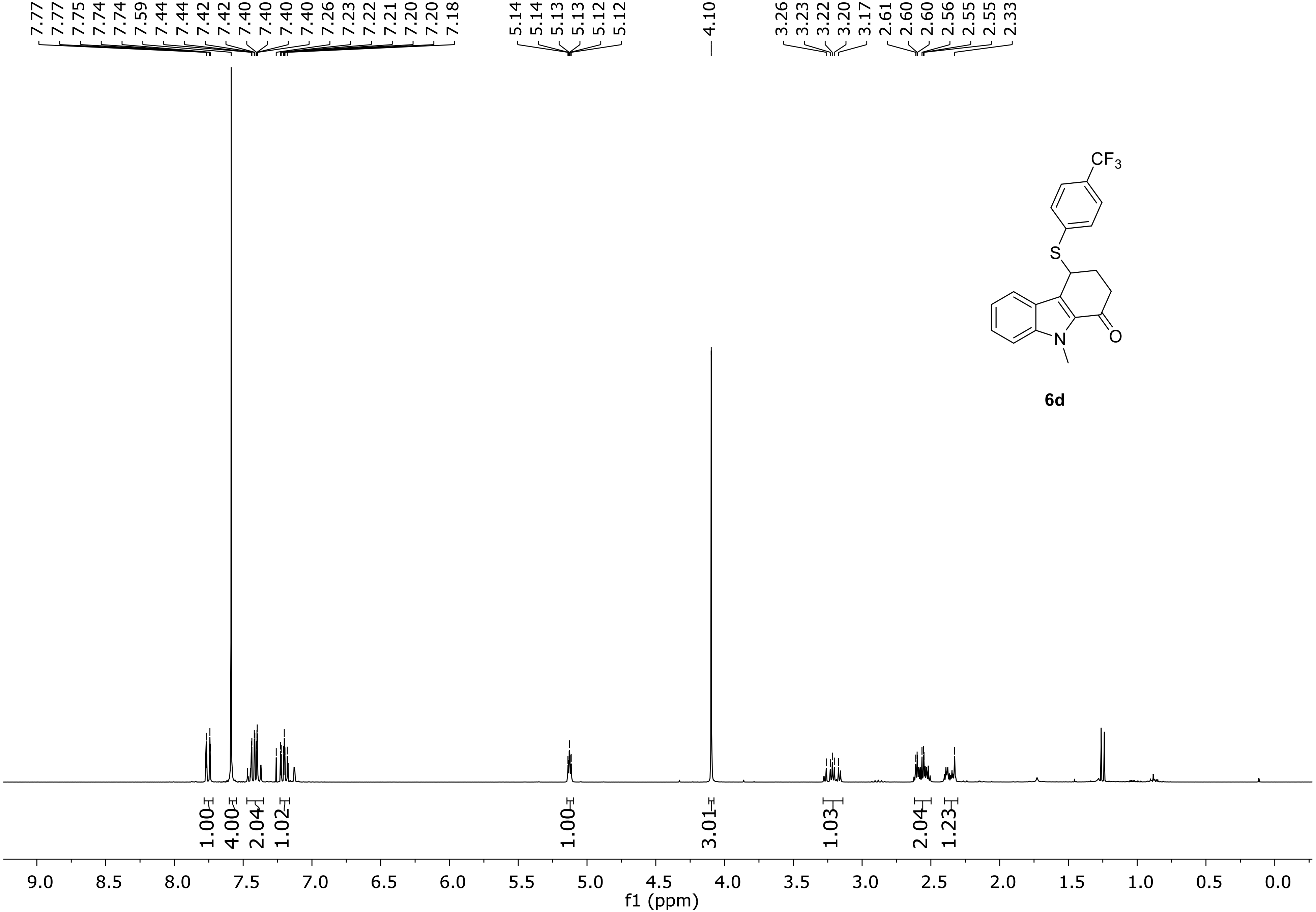

$^{13}C\{^{1}H\}$-NMR (75.4 MHz, $CDCl_3$)

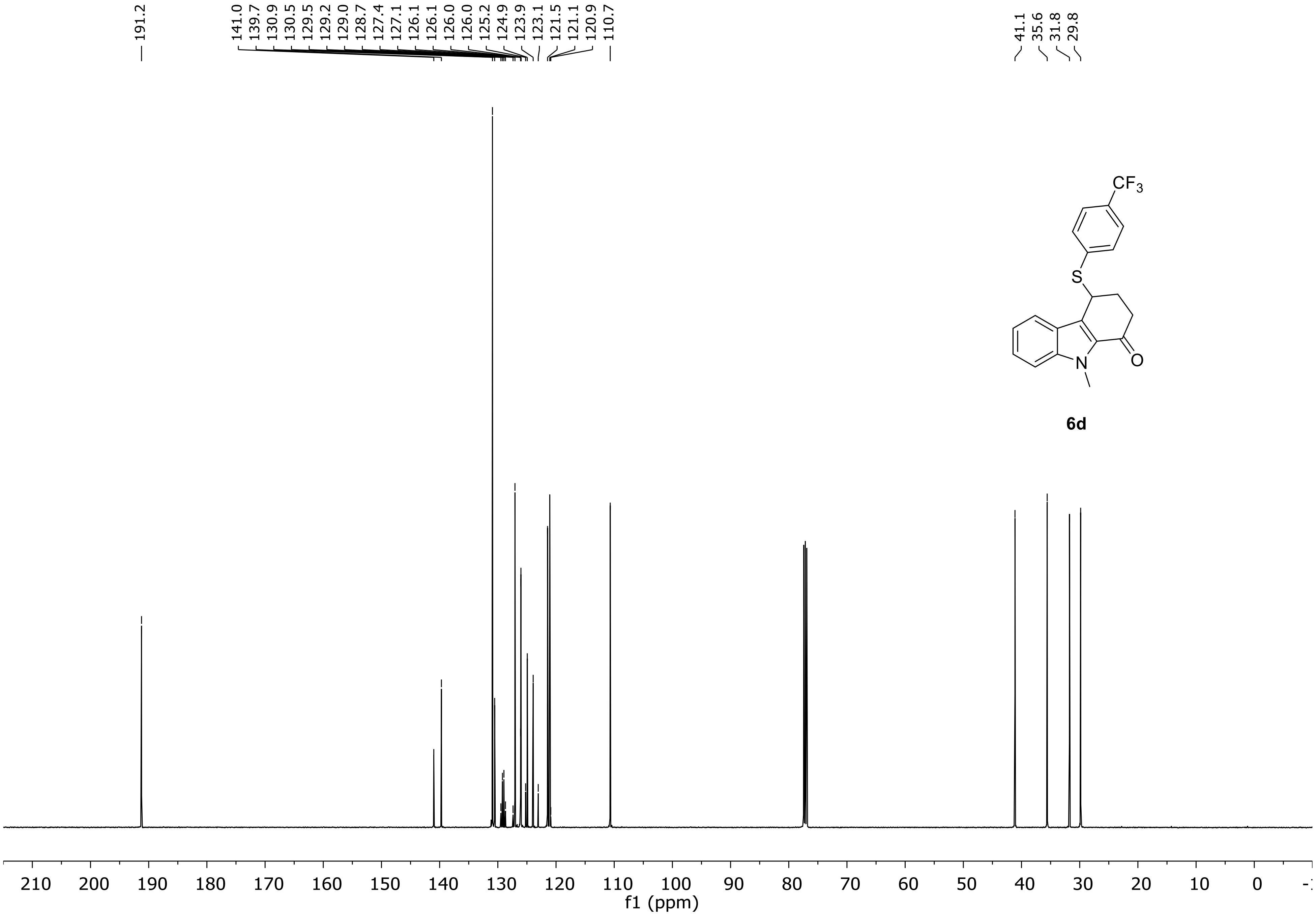

6d

^{19}F-NMR (282 MHz, $CDCl_3$)

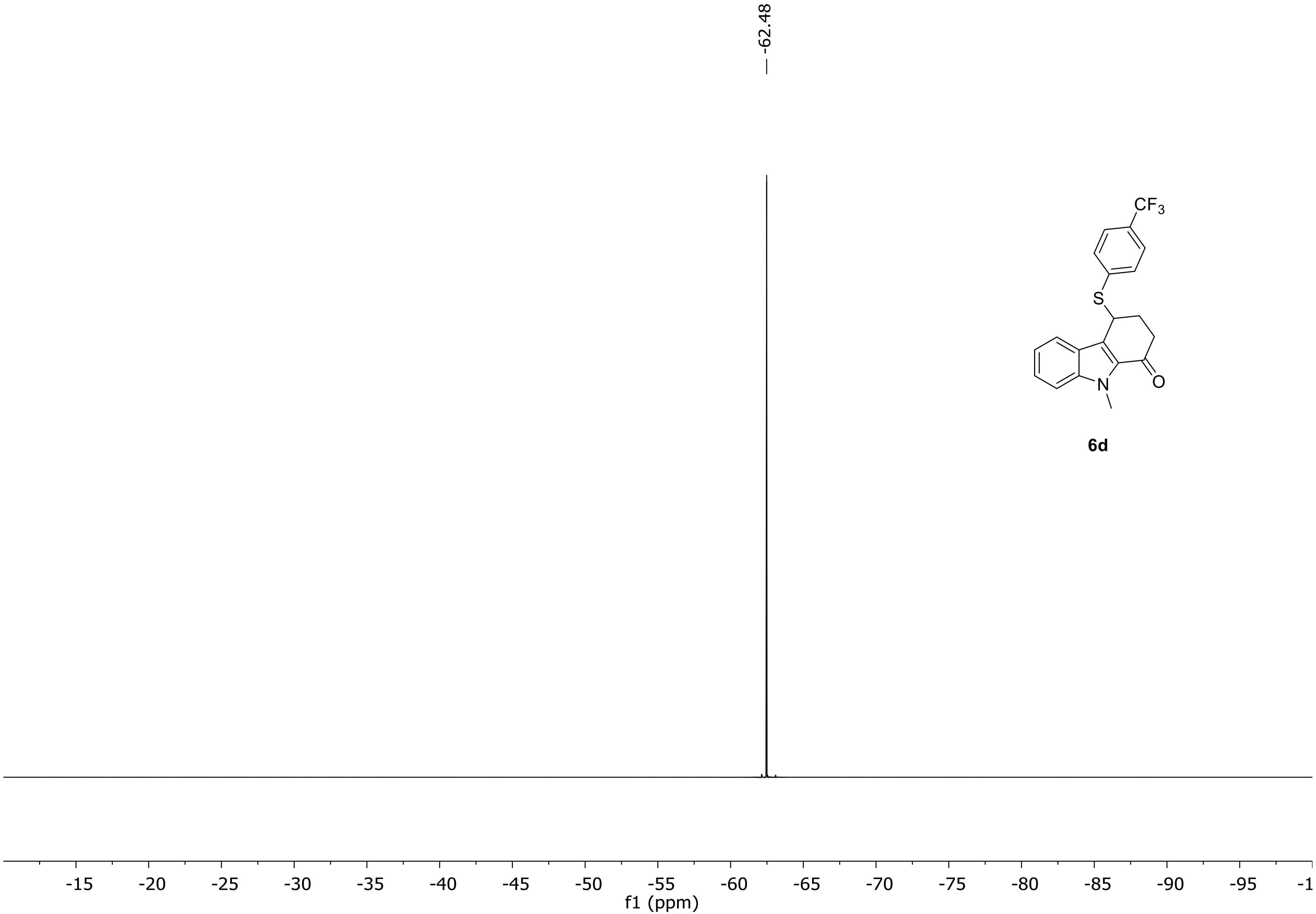

^{1}H-NMR (300 MHz, $CDCl_3$)

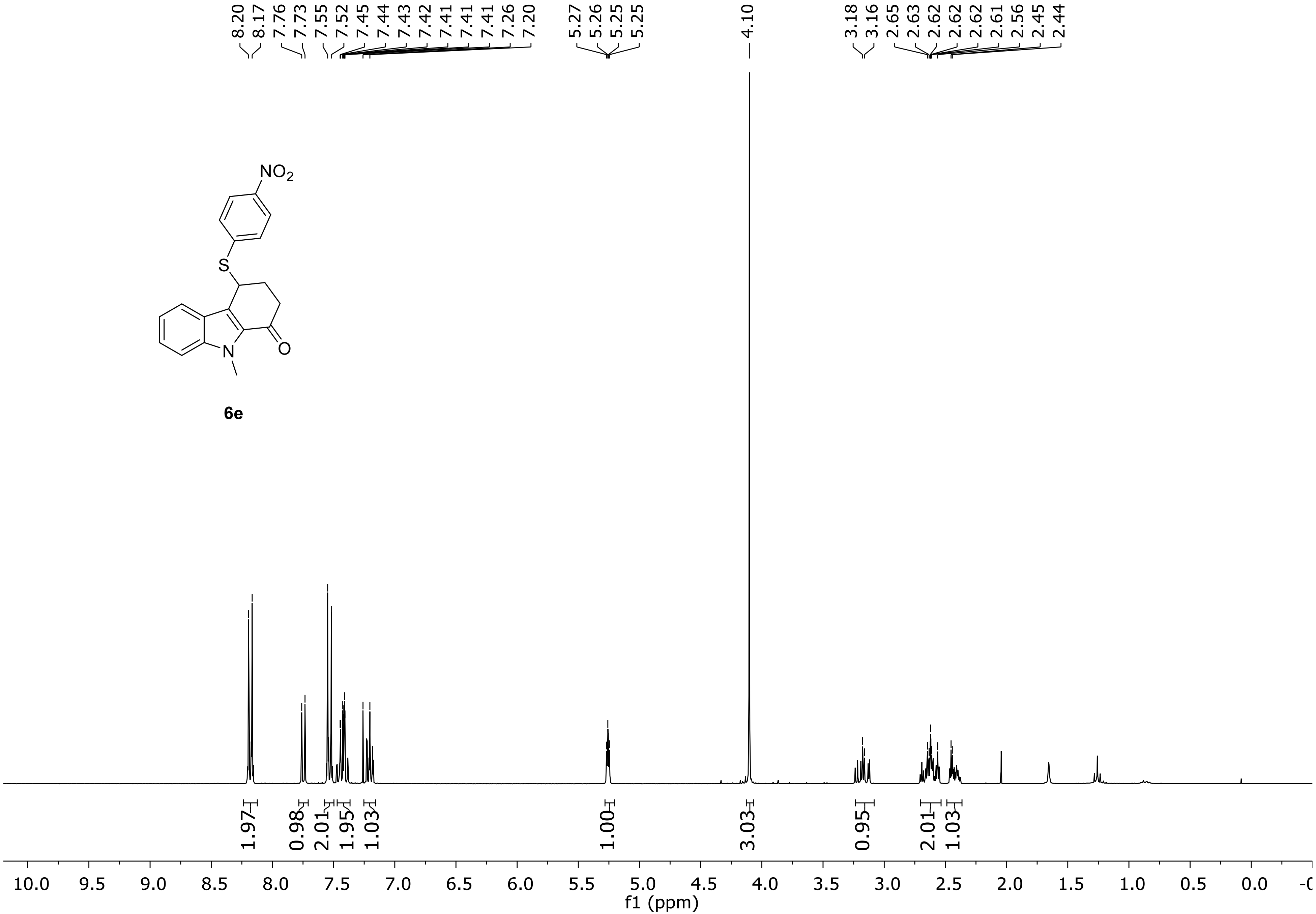

$^{13}C-\{^{1}H\}$NMR (75.4 MHz, $CDCl_3$)

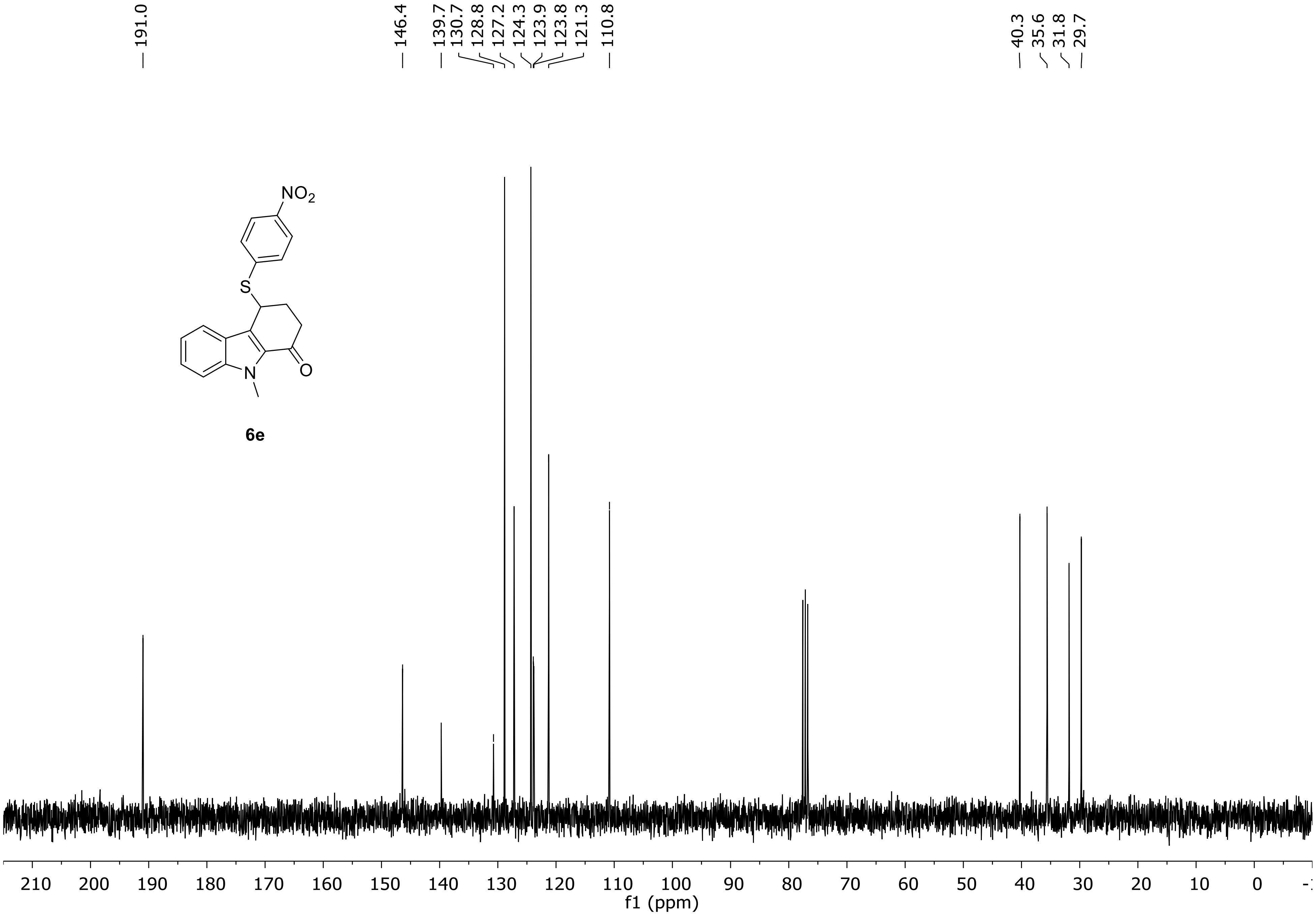

^{1}H-NMR (300 MHz, $CDCl_3$)

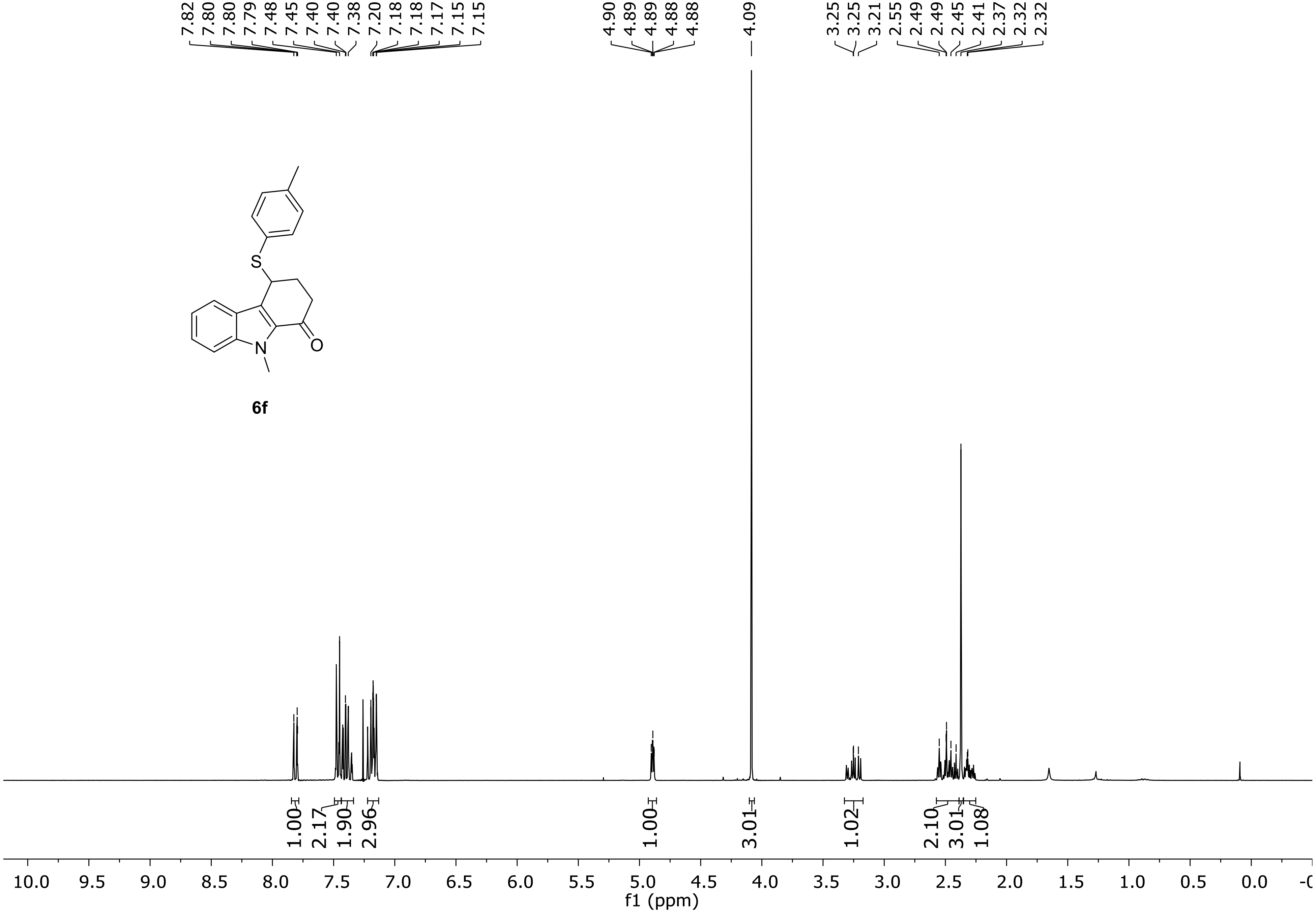

^{13}C-{^{1}H}NMR (75.4 MHz, $CDCl_3$)

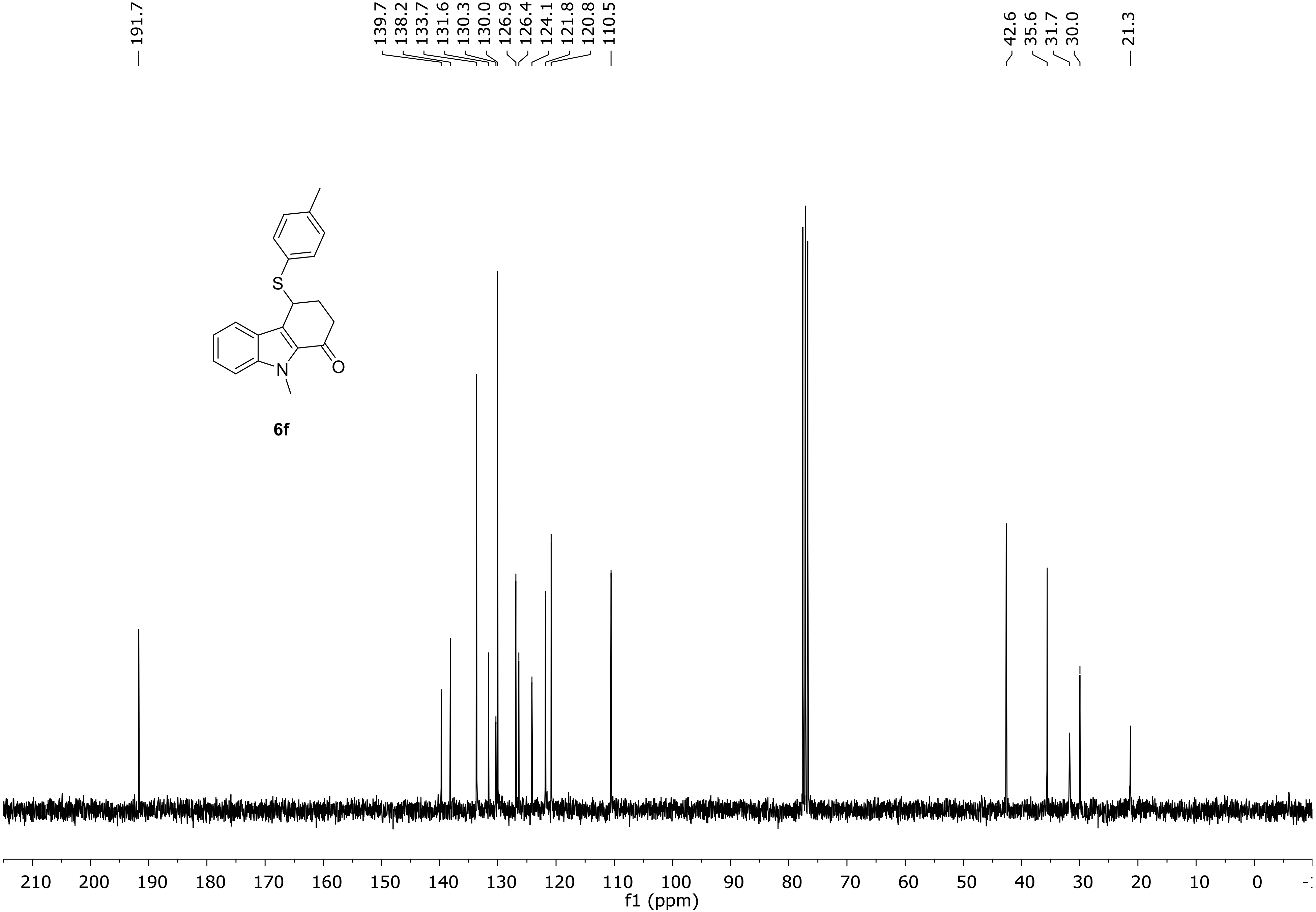

^{1}H-NMR (300 MHz, $CDCl_3$)

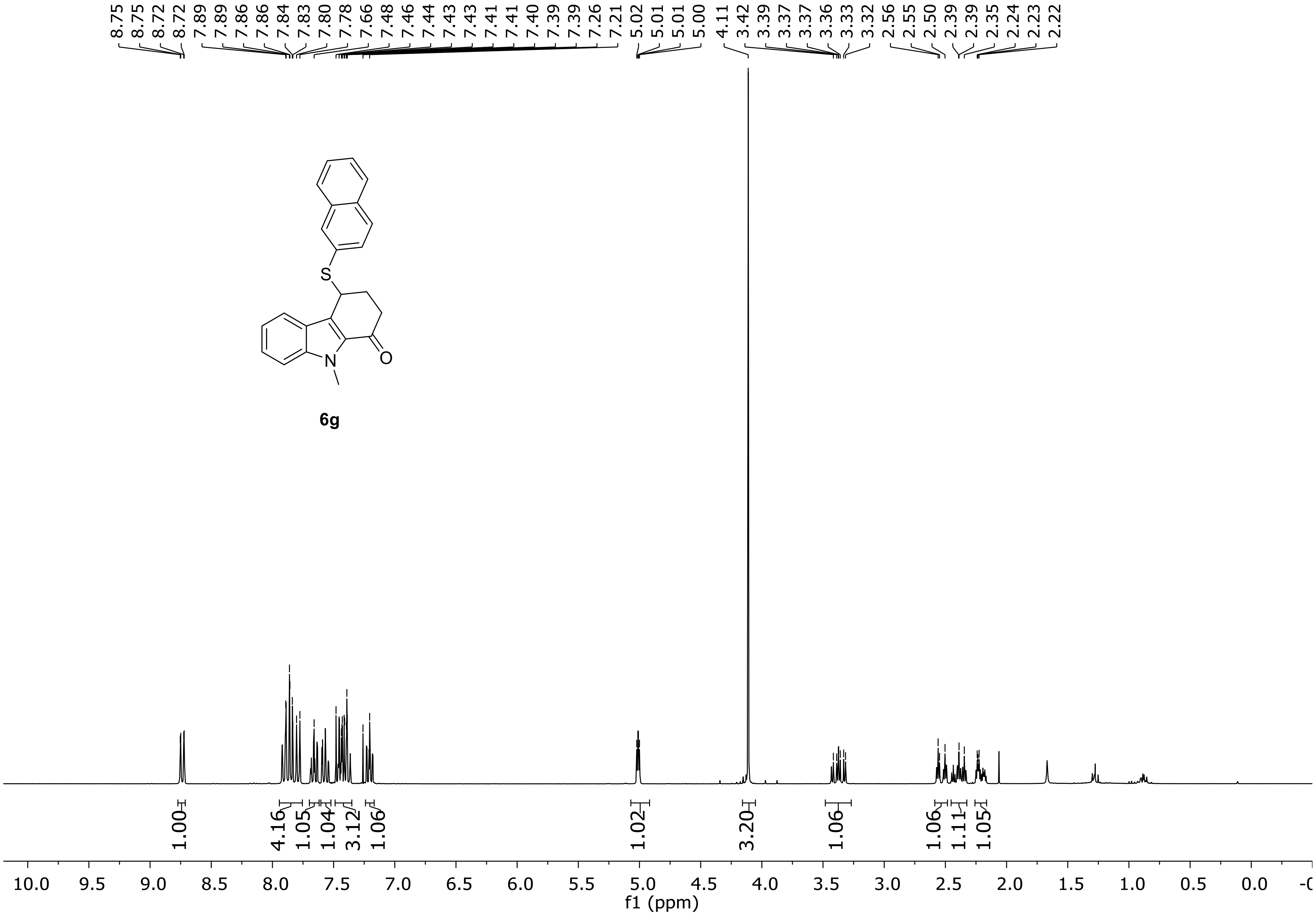

^{13}C-{^{1}H}NMR (75.4 MHz, $CDCl_3$)

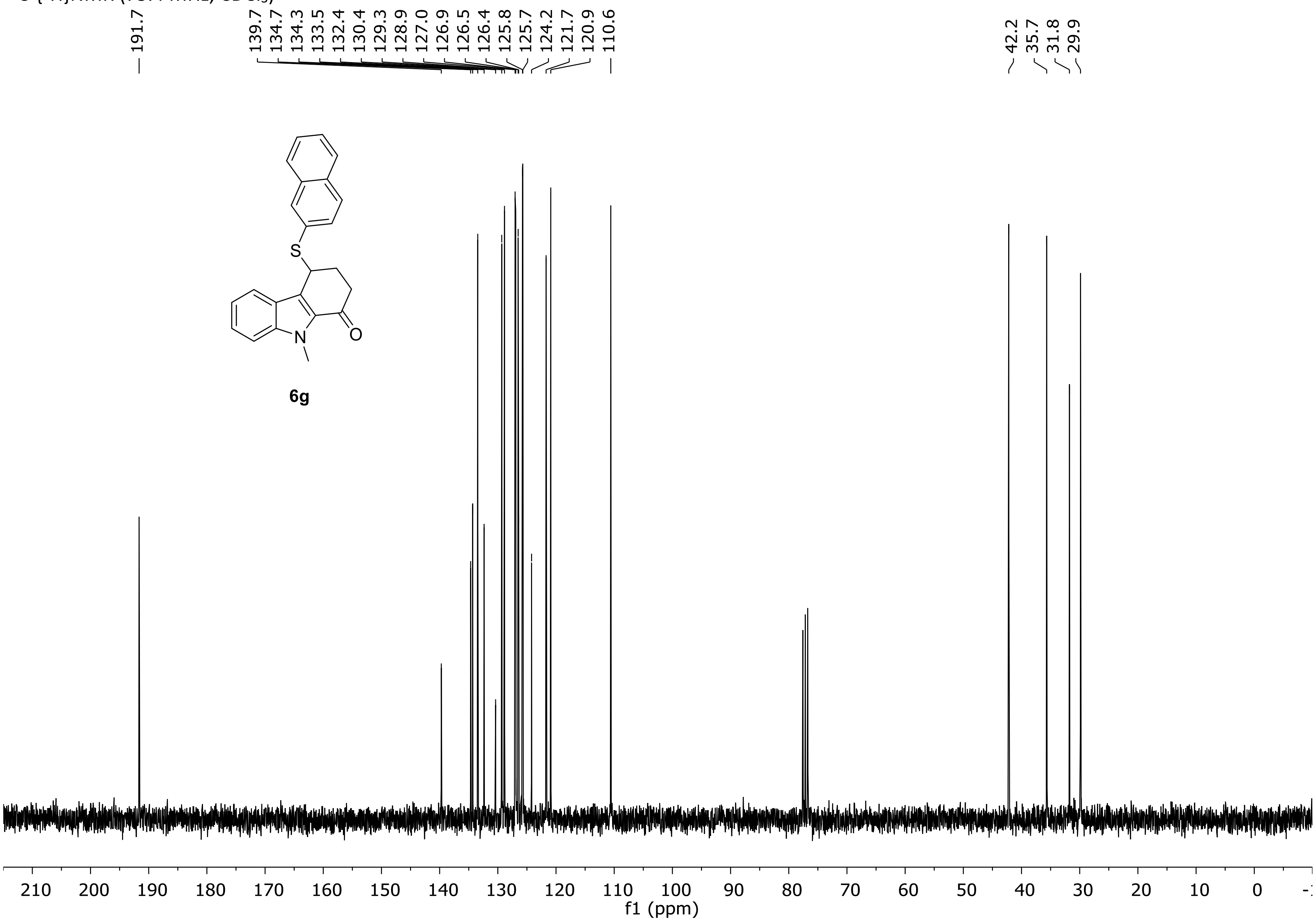

6g

^{1}H-NMR (300 MHz, $CDCl_3$)

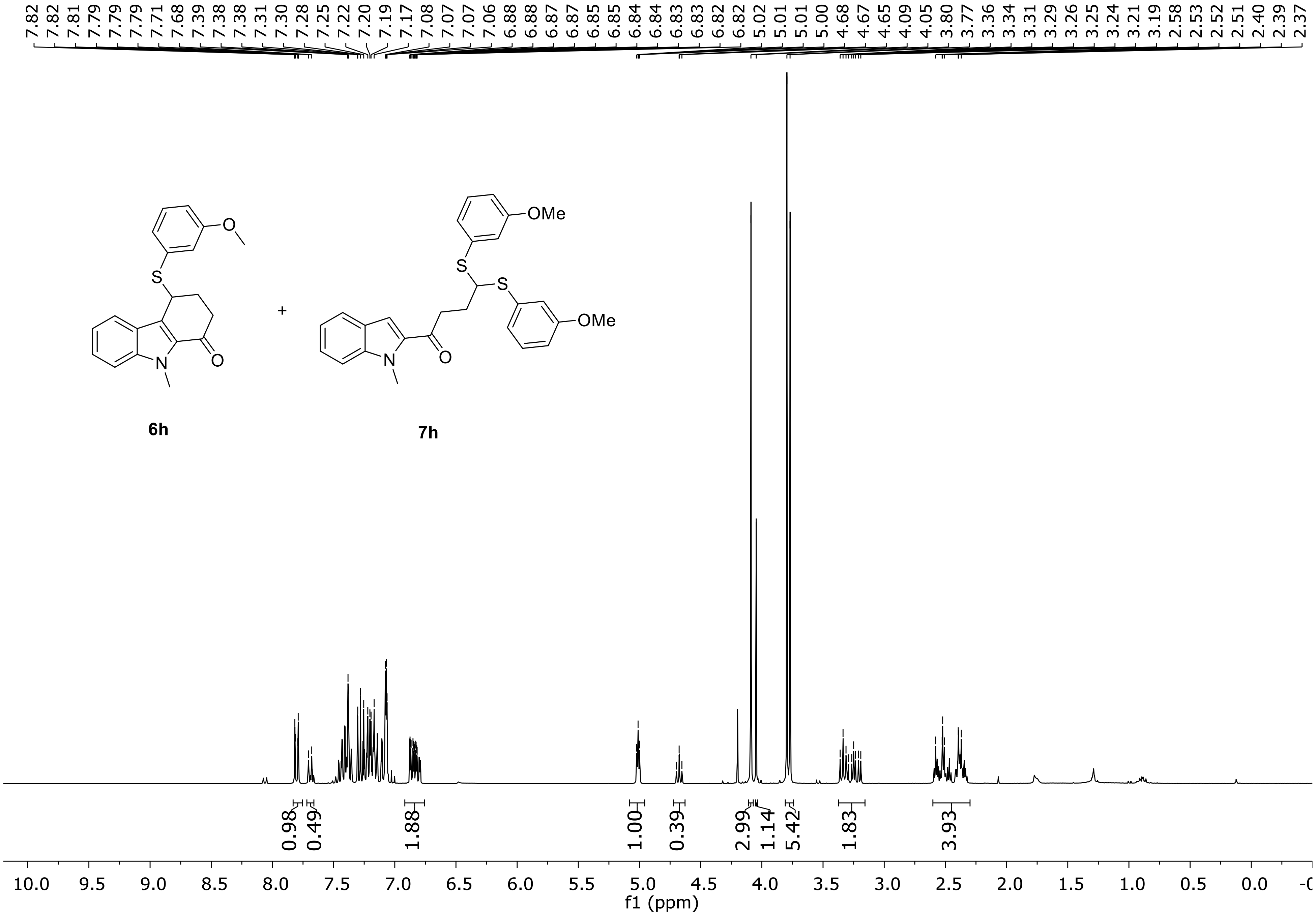

^{13}C-{^{1}H}NMR (75.4 MHz, $CDCl_3$)

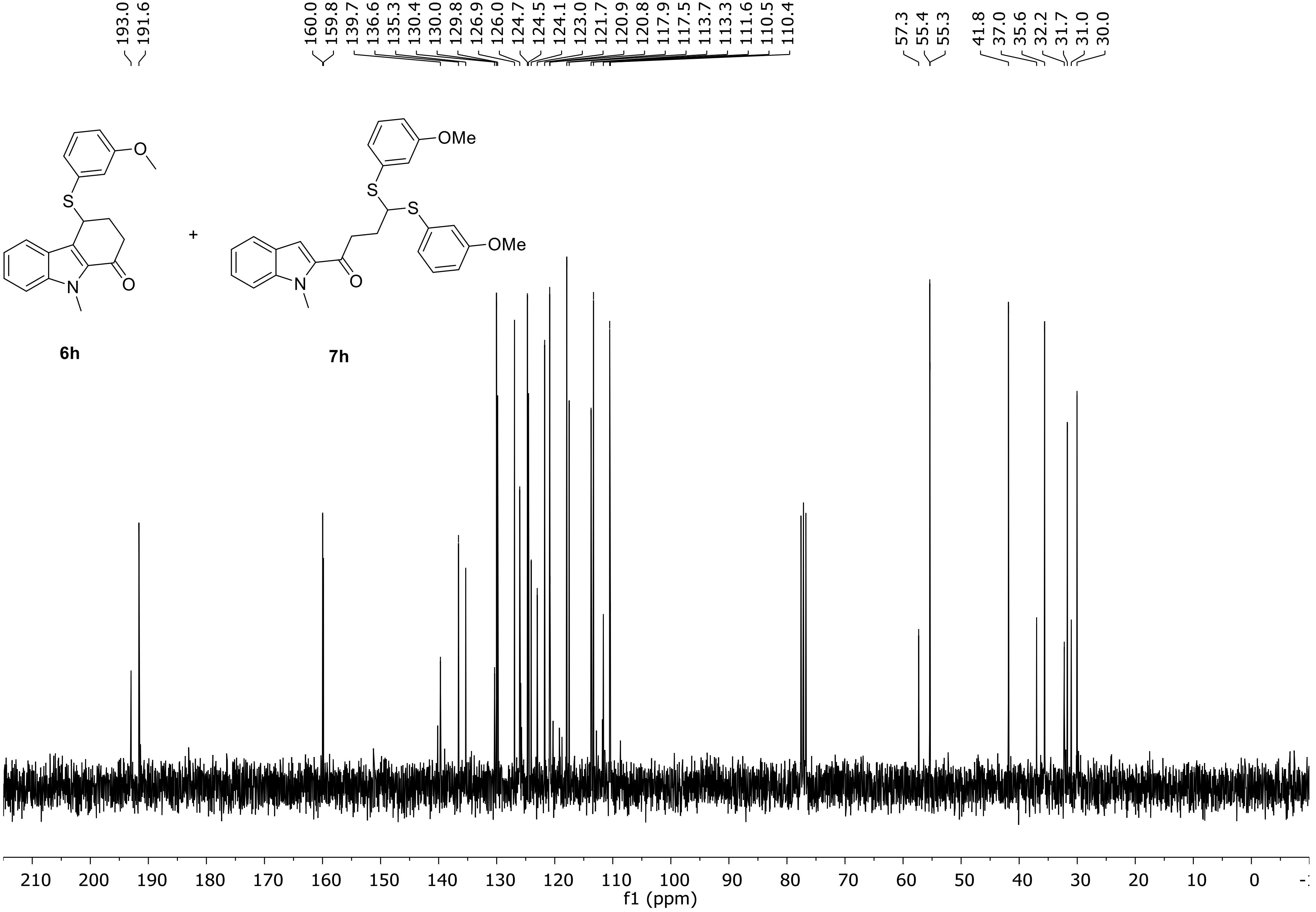

^{1}H-NMR (300 MHz, $CDCl_3$)

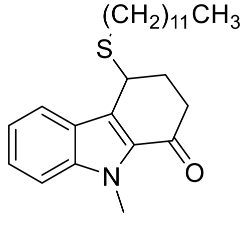

6i

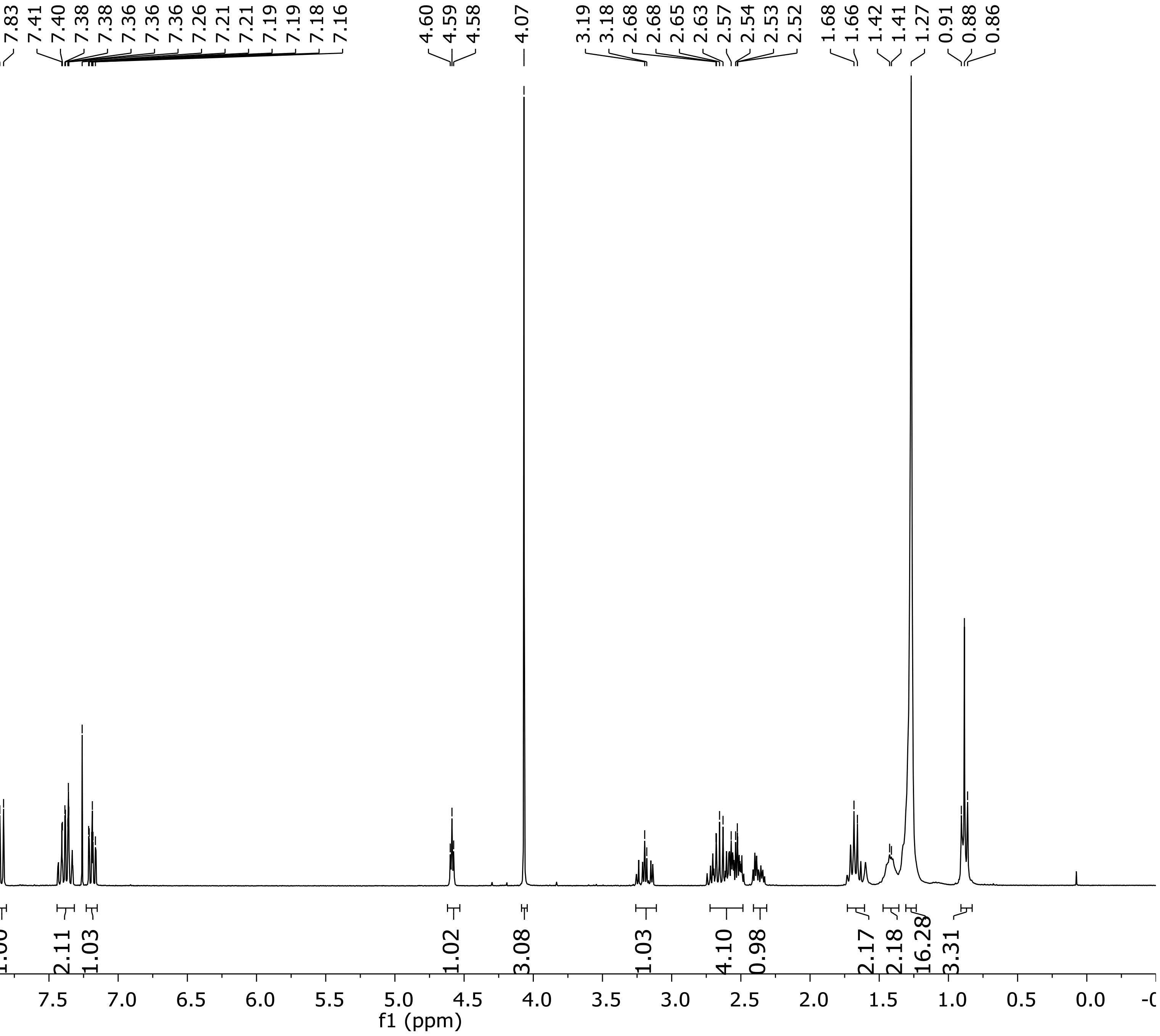

13C-{1H}NMR (75.4 MHz, CDCl3)

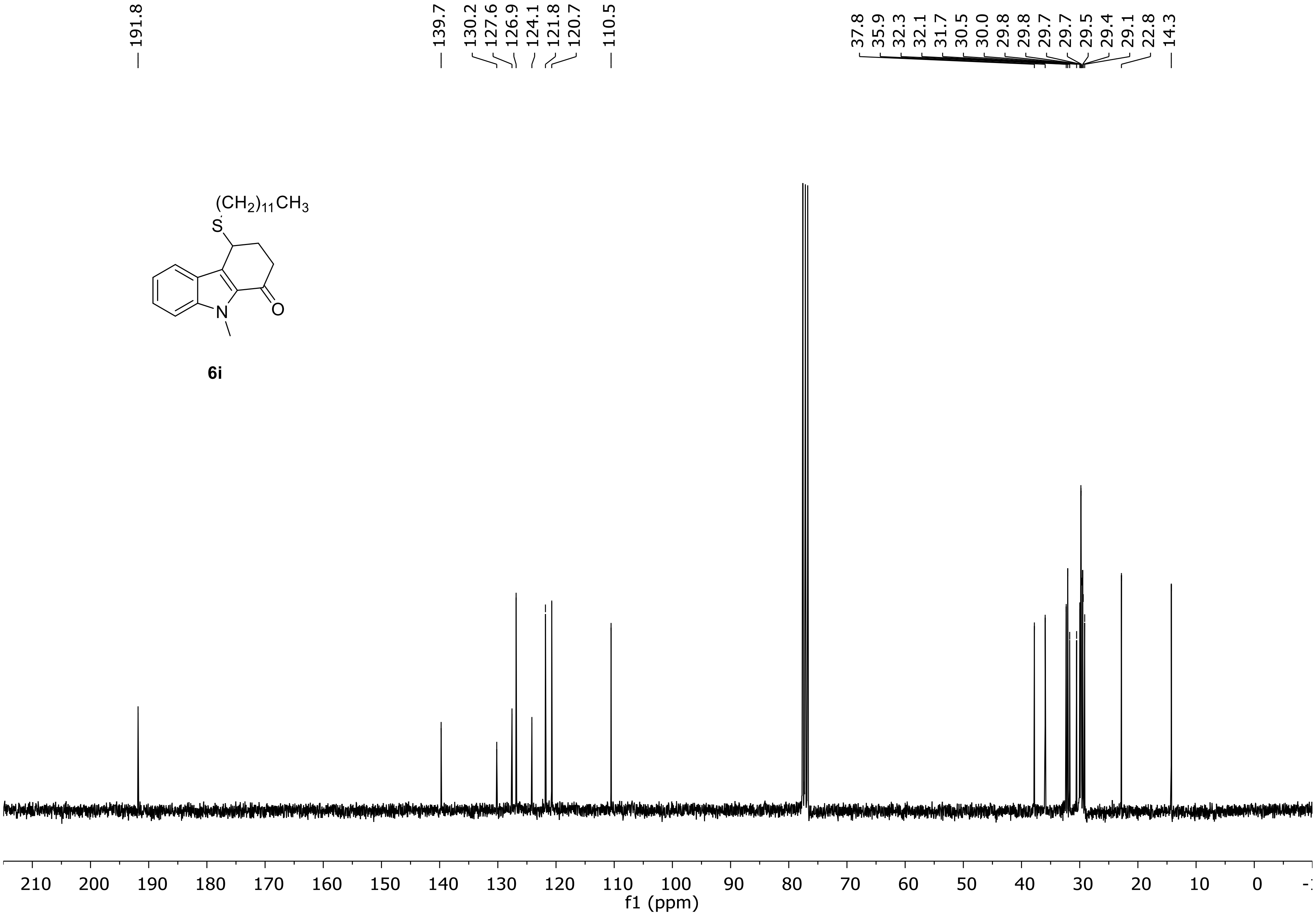

^{1}H-NMR (300 MHz, $CDCl_3$)

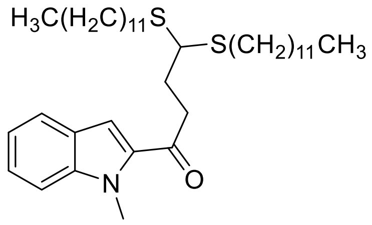

7i

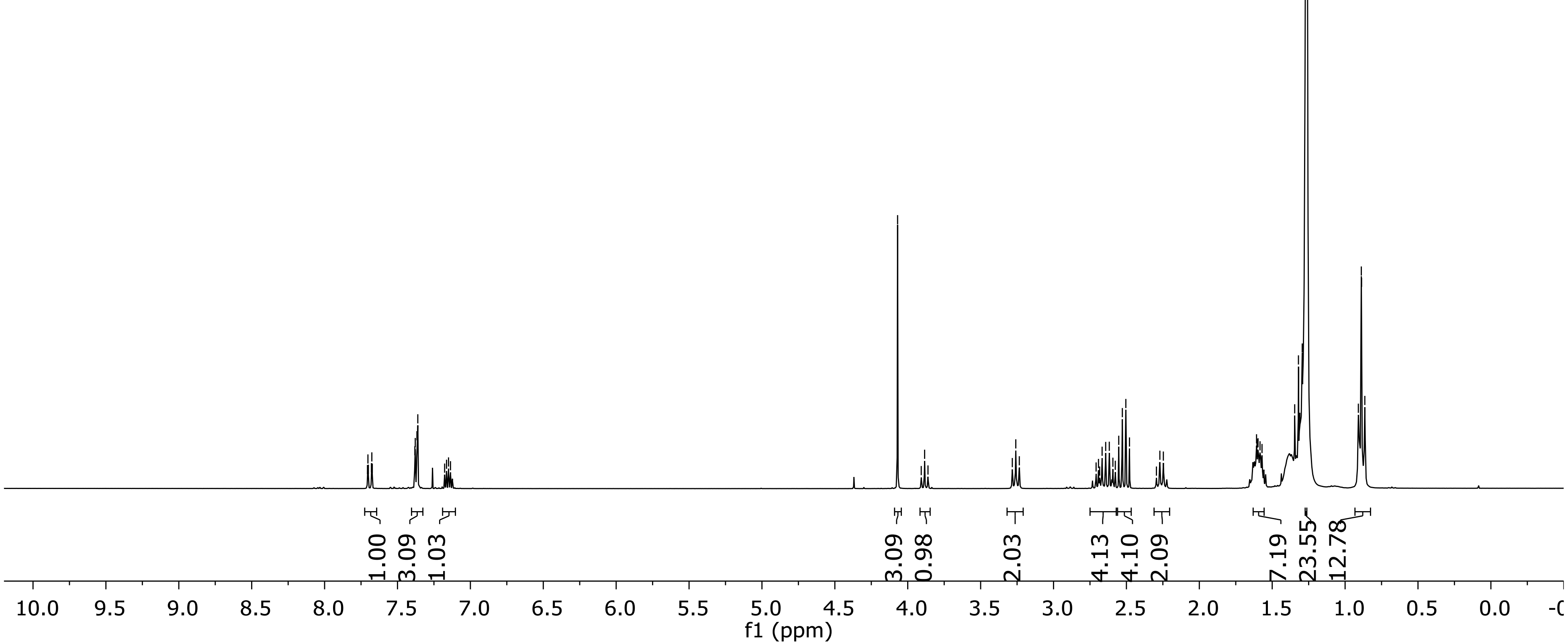

^{13}C-{^{1}H}NMR (75.4 MHz, $CDCl_3$)

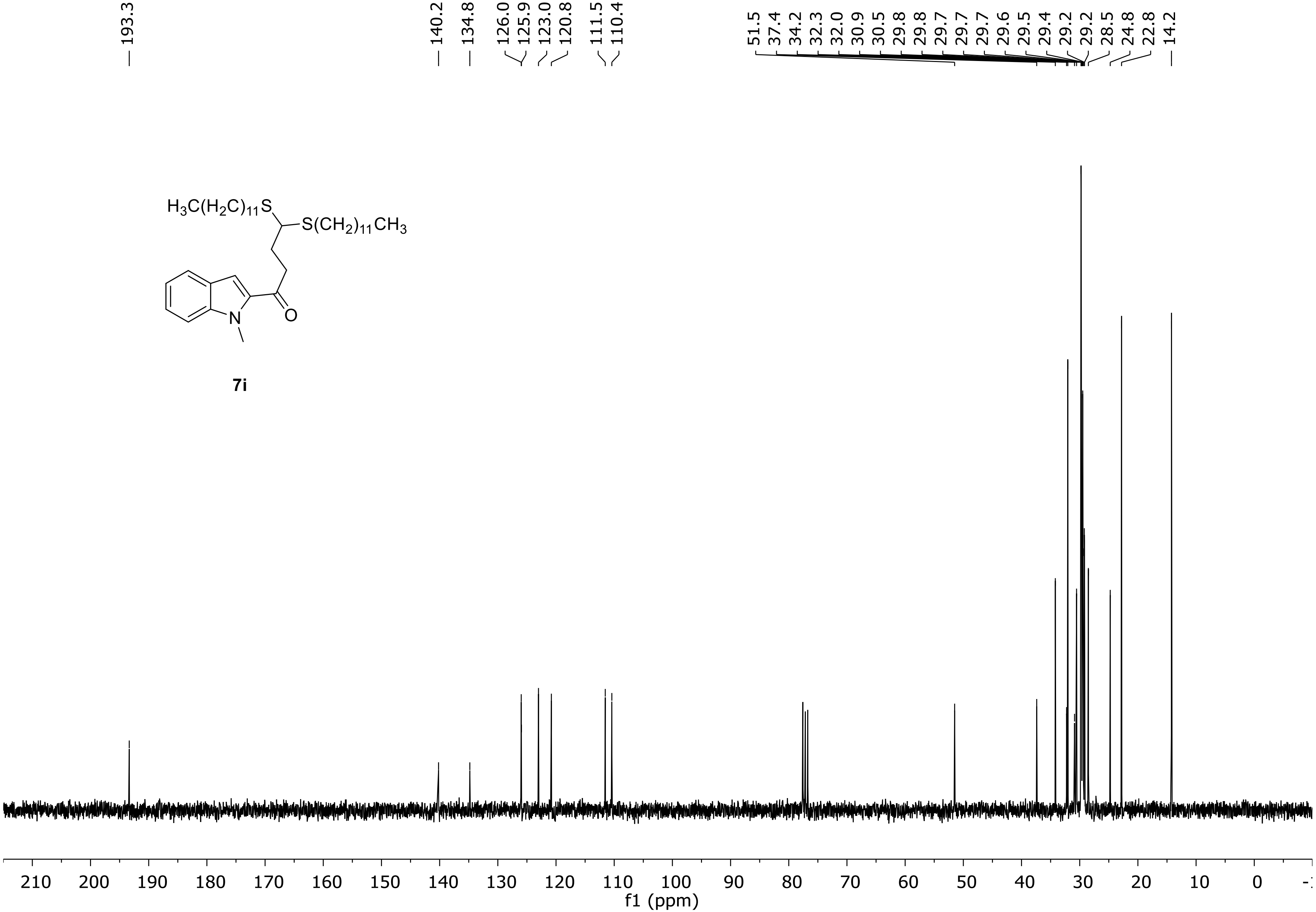

^{1}H-NMR (300 MHz, $CDCl_3$)

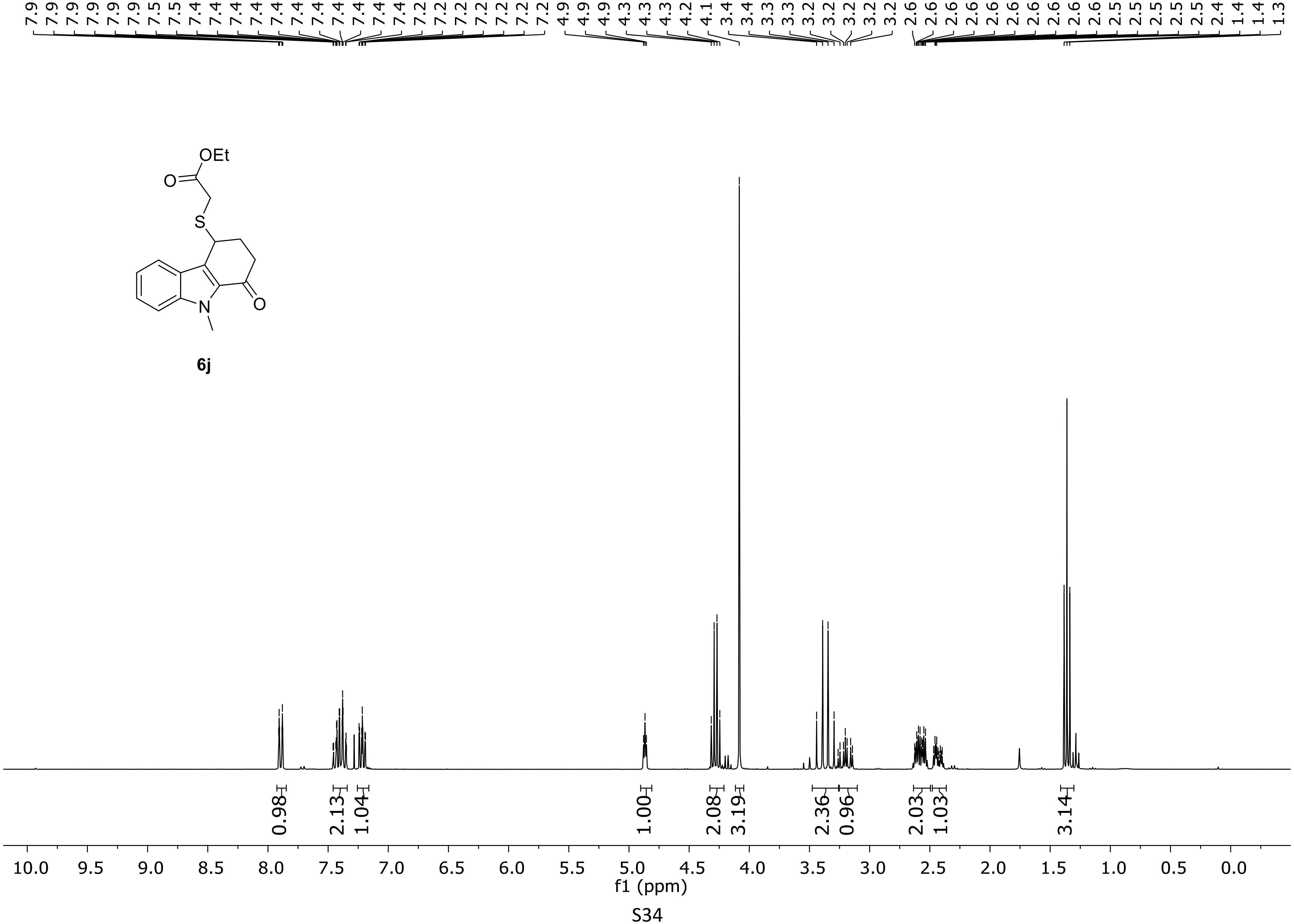

6j

$^{13}C-\{^{1}H\}$NMR (75.4 MHz, $CDCl_3$)

6j

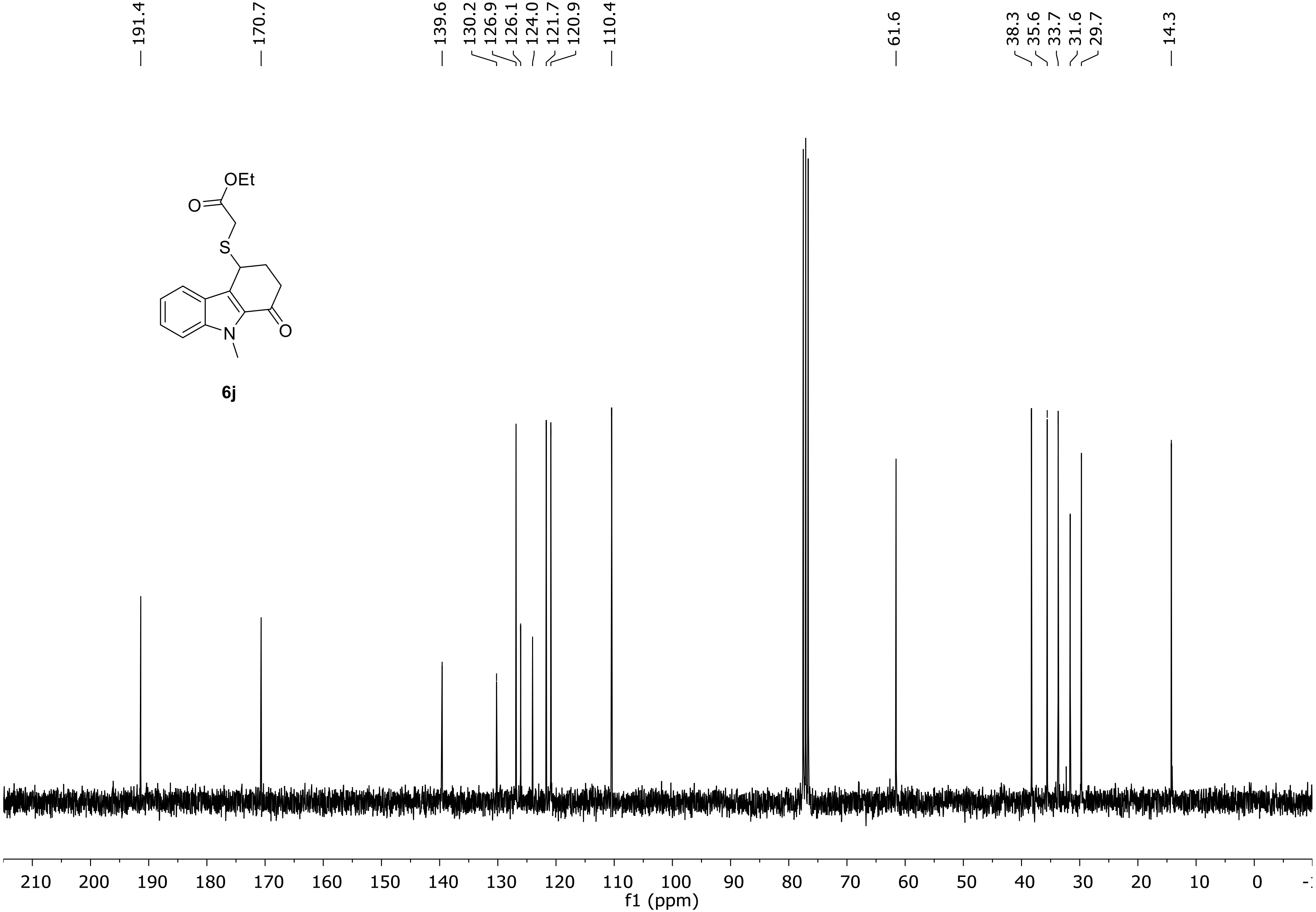

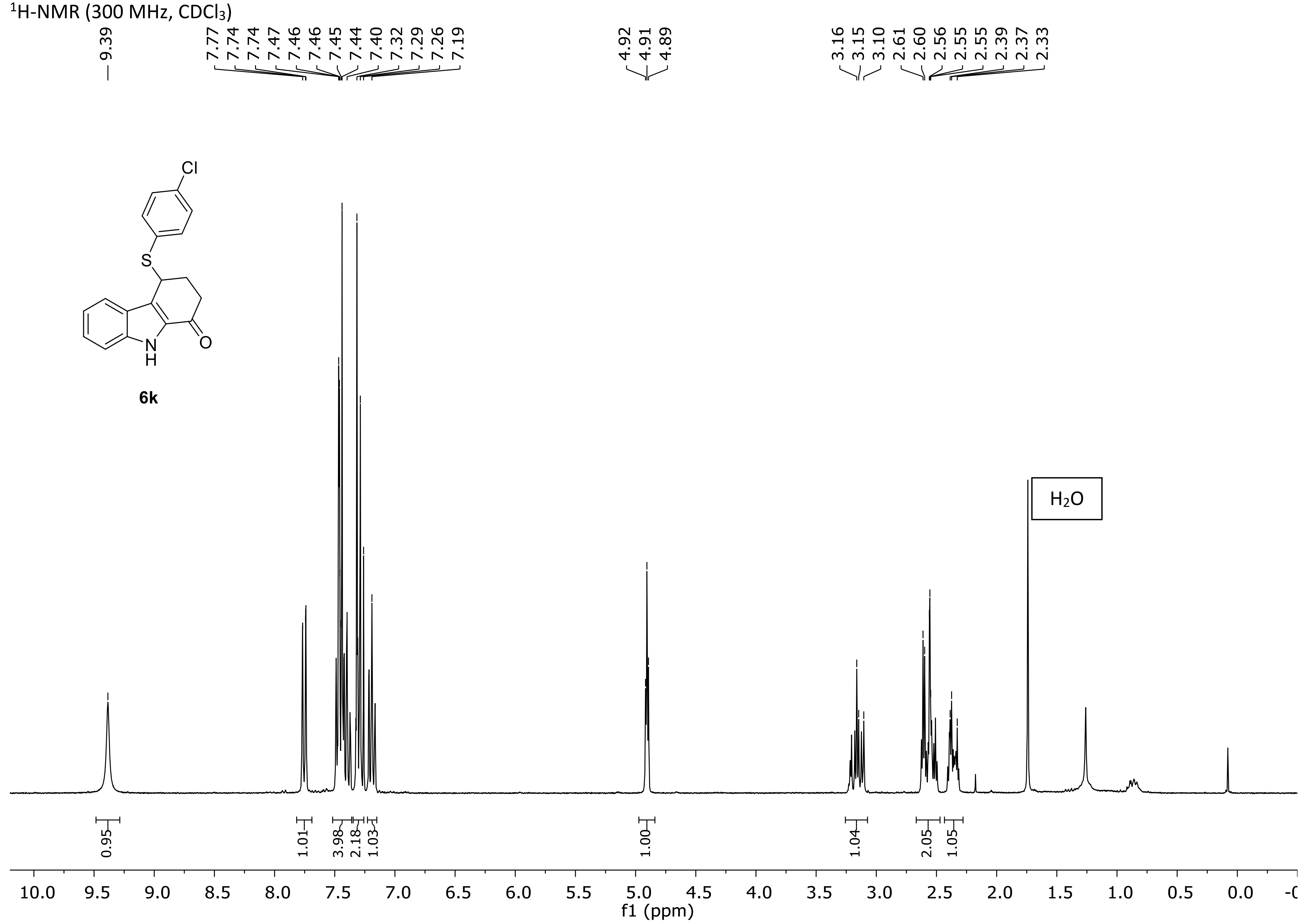

^1H-NMR (300 MHz, $CDCl_3$)
9.39
7.77
7.74
7.74
7.47
7.46
7.46
7.45
7.44
7.40
7.32
7.29
7.26
7.19
4.92
4.91
4.89
3.16
3.15
3.10
2.61
2.60
2.56
2.55
2.55
2.39
2.37
2.33
Cl
S
N
H
O
6k
H_2O
0.95
1.01
3.98
2.18
1.03
1.00
1.04
2.05
1.05
10.0
9.5
9.0
8.5
8.0
7.5
7.0
6.5
6.0
5.5
5.0
4.5
4.0
3.5
3.0
2.5
2.0
1.5
1.0
0.5
0.0
f1 (ppm)

^{13}C-{^{1}H}NMR (75.4 MHz, $CDCl_3$)

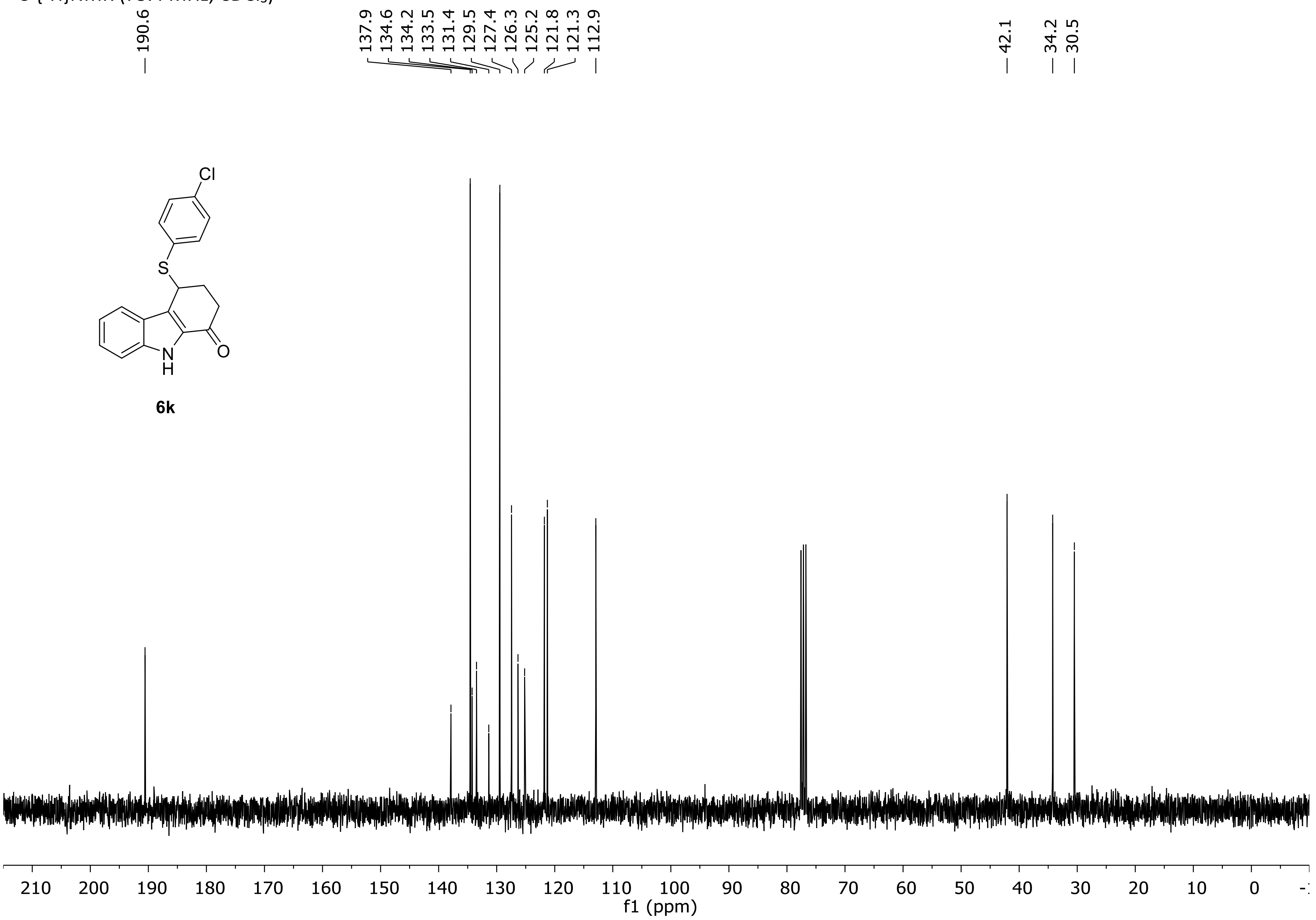

^{1}H-NMR (300 MHz, $CDCl_3$)

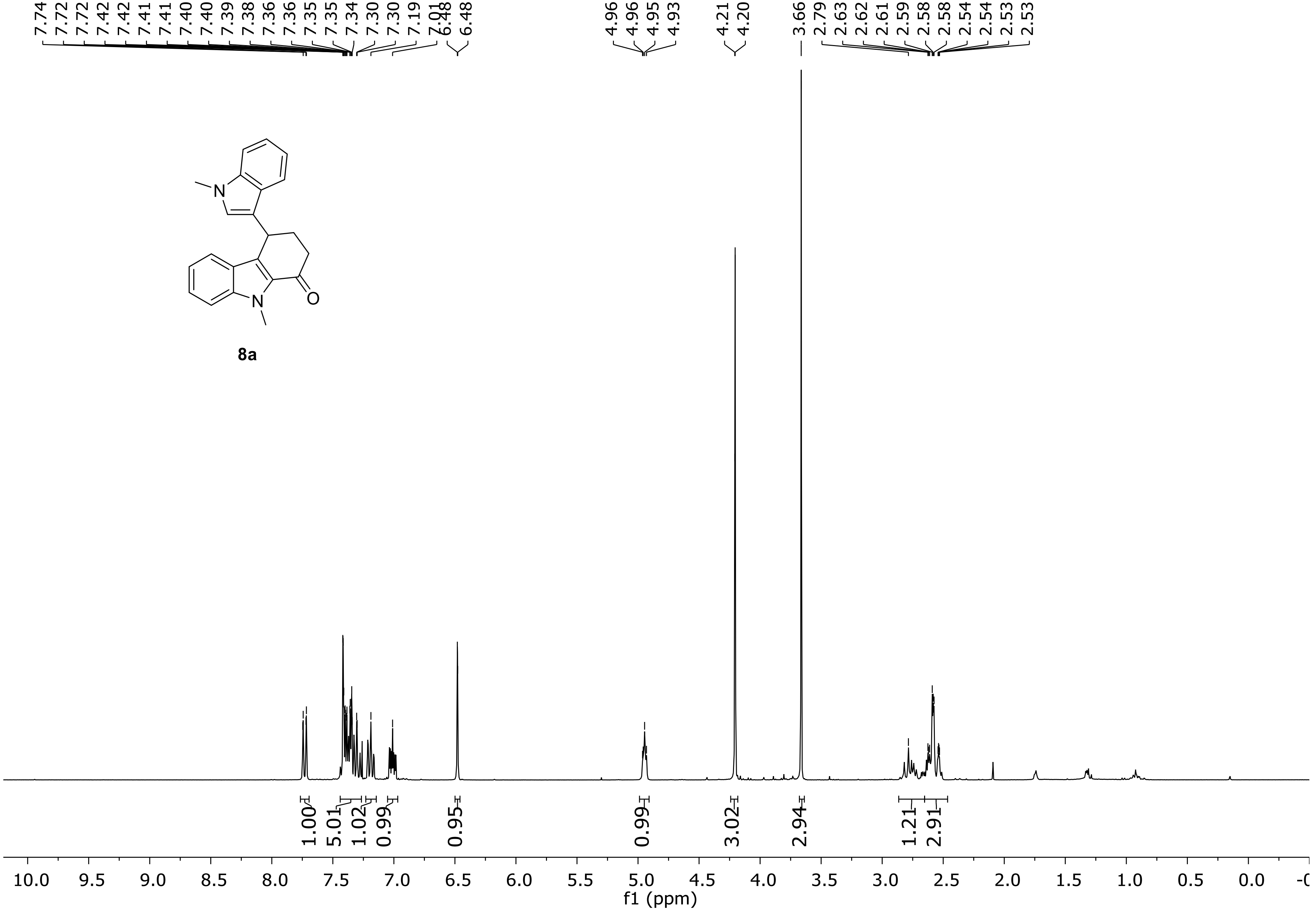

8a

^{13}C-{^1H}NMR (75.4 MHz, $CDCl_3$)

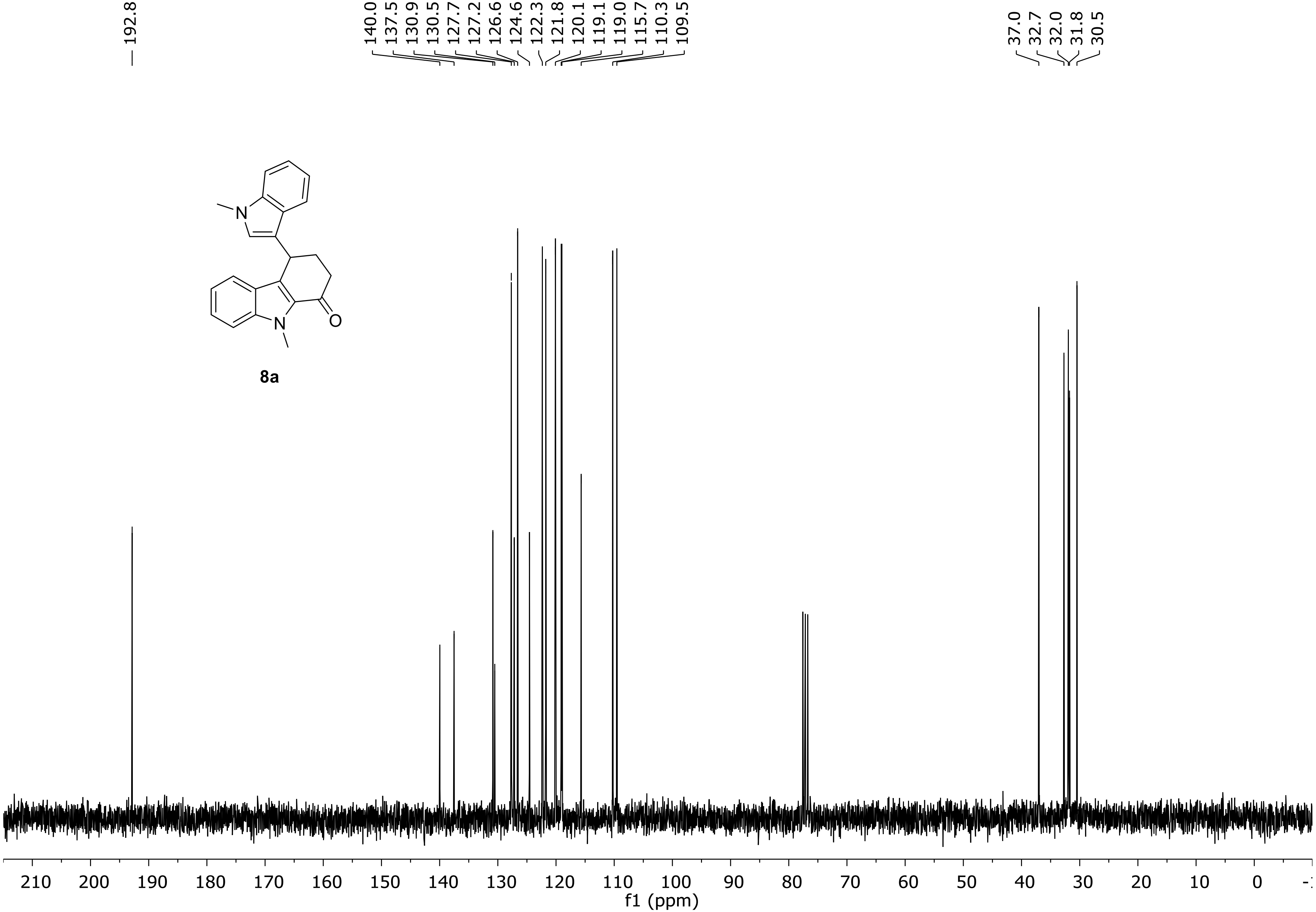

^{1}H-NMR (300 MHz, $CDCl_3$)

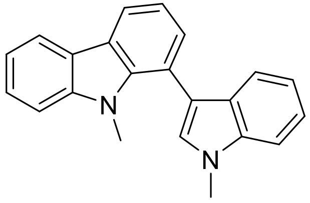

9a

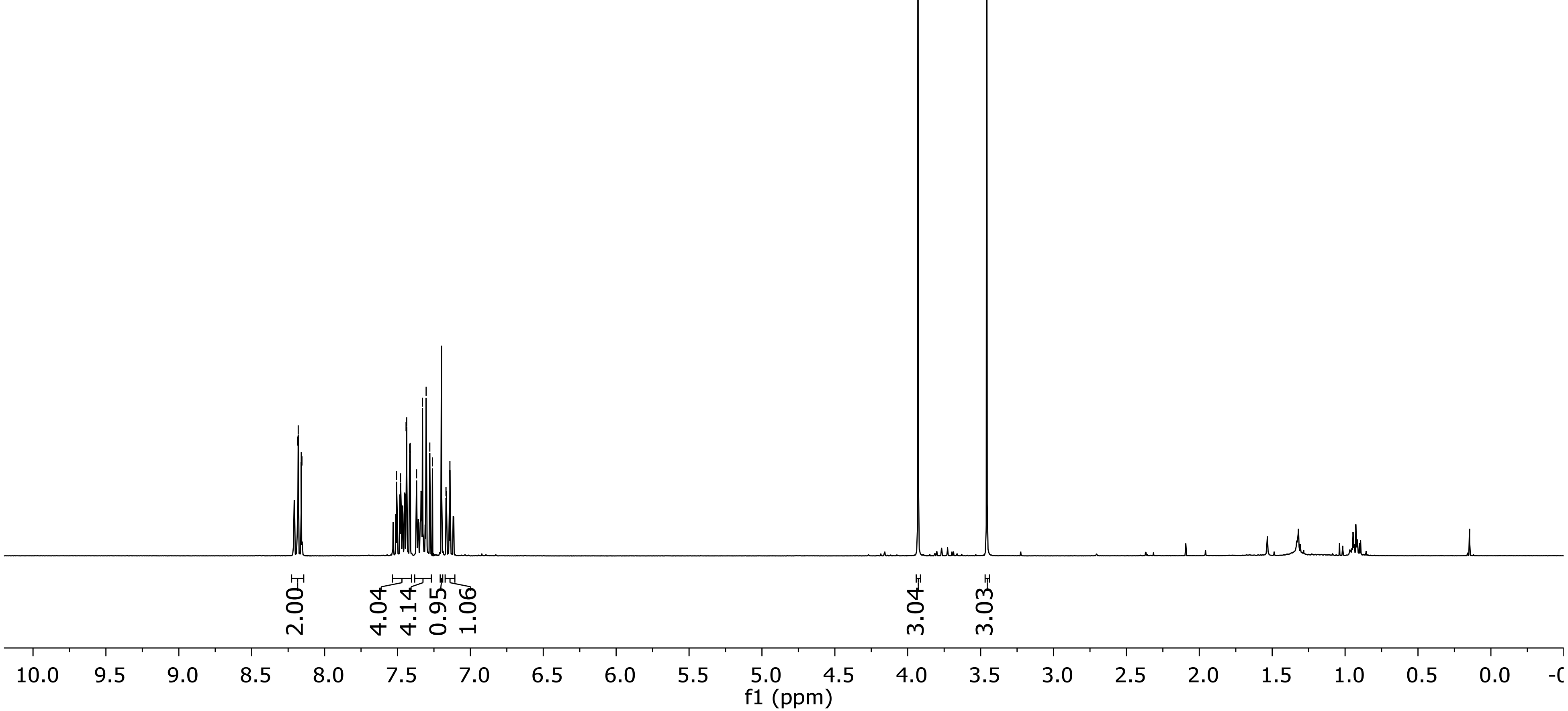

^{13}C-{^{1}H}NMR (75.4 MHz, $CDCl_3$)

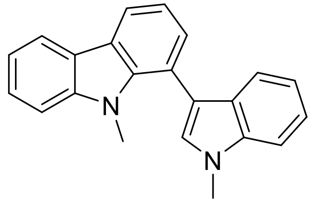

9a

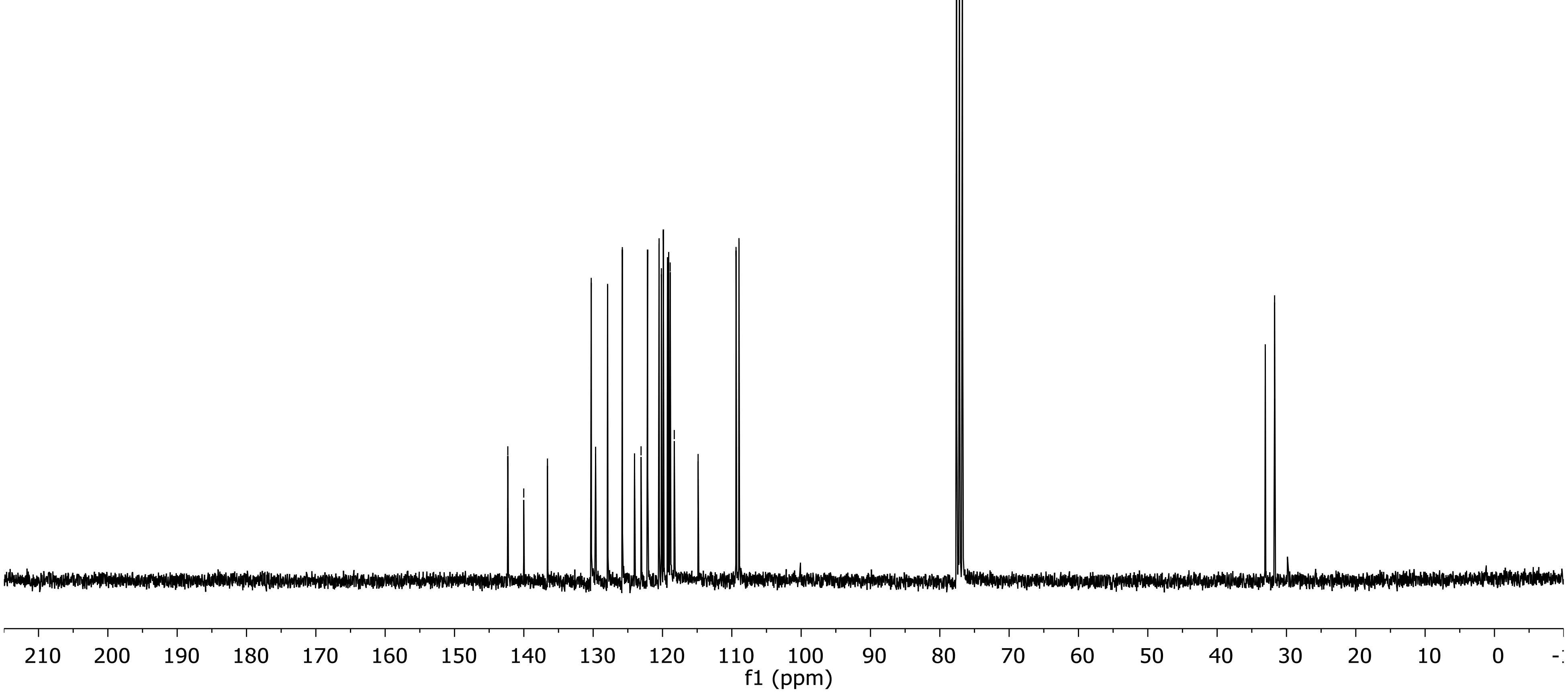

^{1}H-NMR (300 MHz, $CDCl_3$)

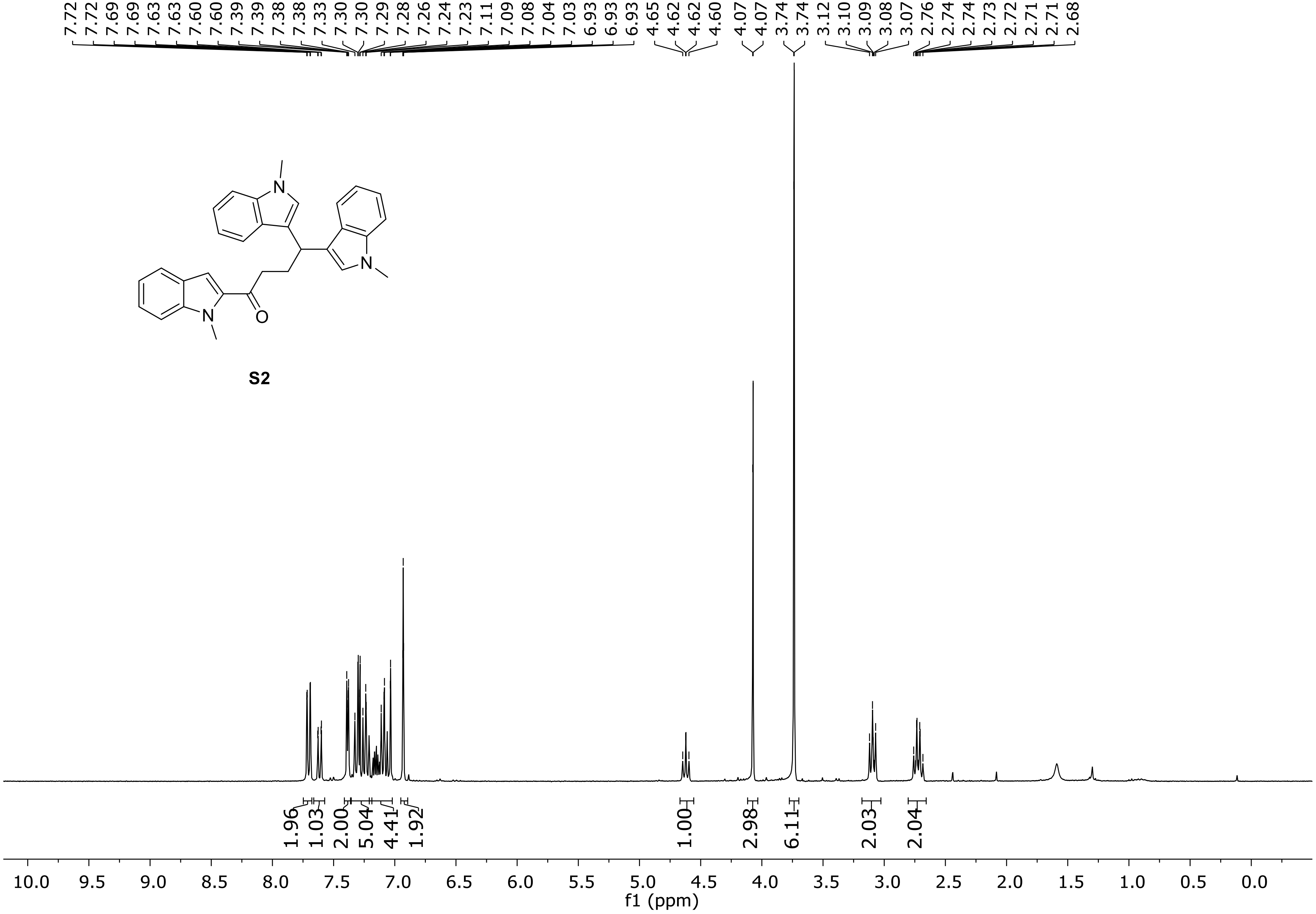

$^{13}C-\{^{1}H\}$NMR (75.4 MHz, $CDCl_3$)

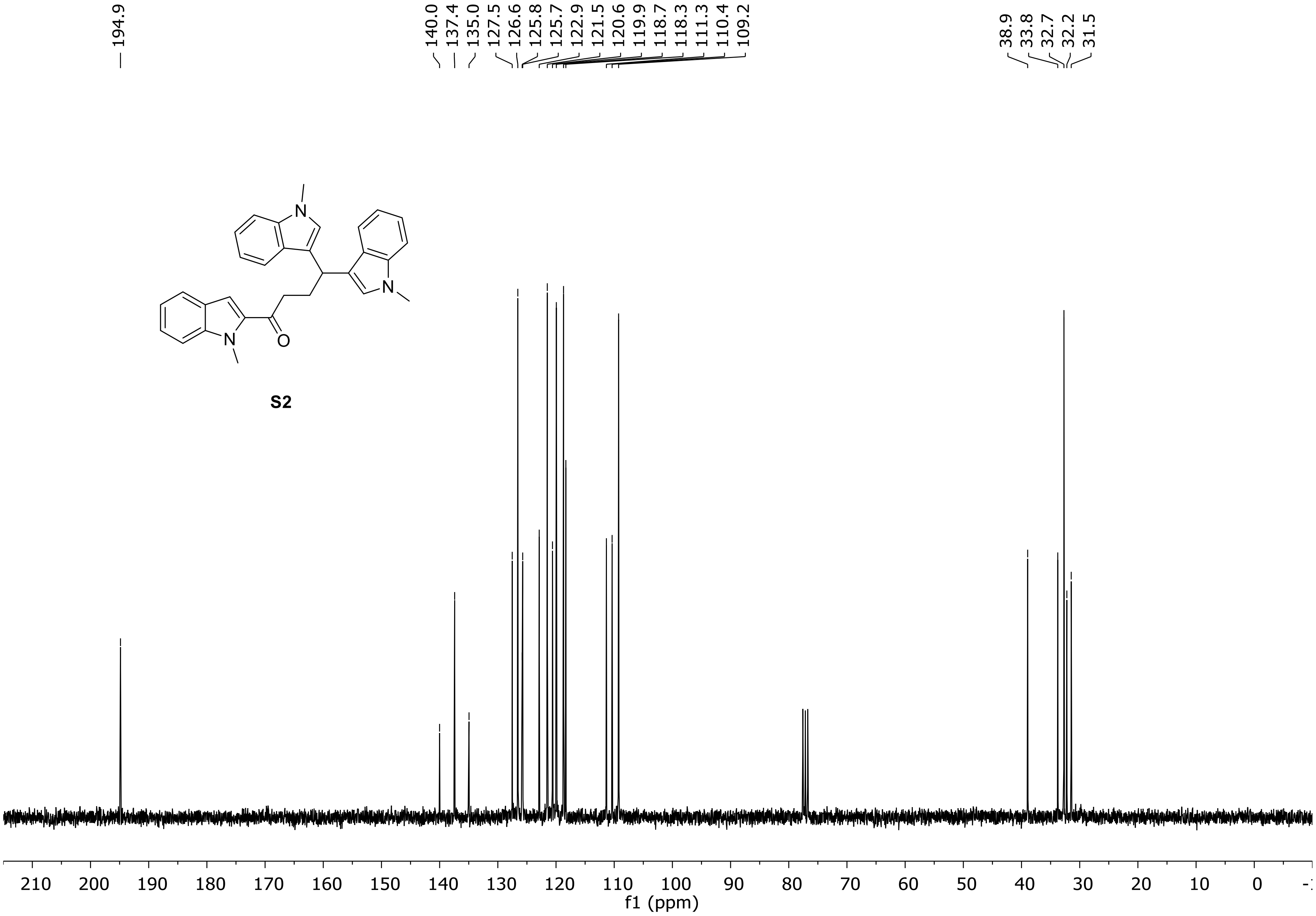

^{1}H-NMR (300 MHz, $CDCl_3$)

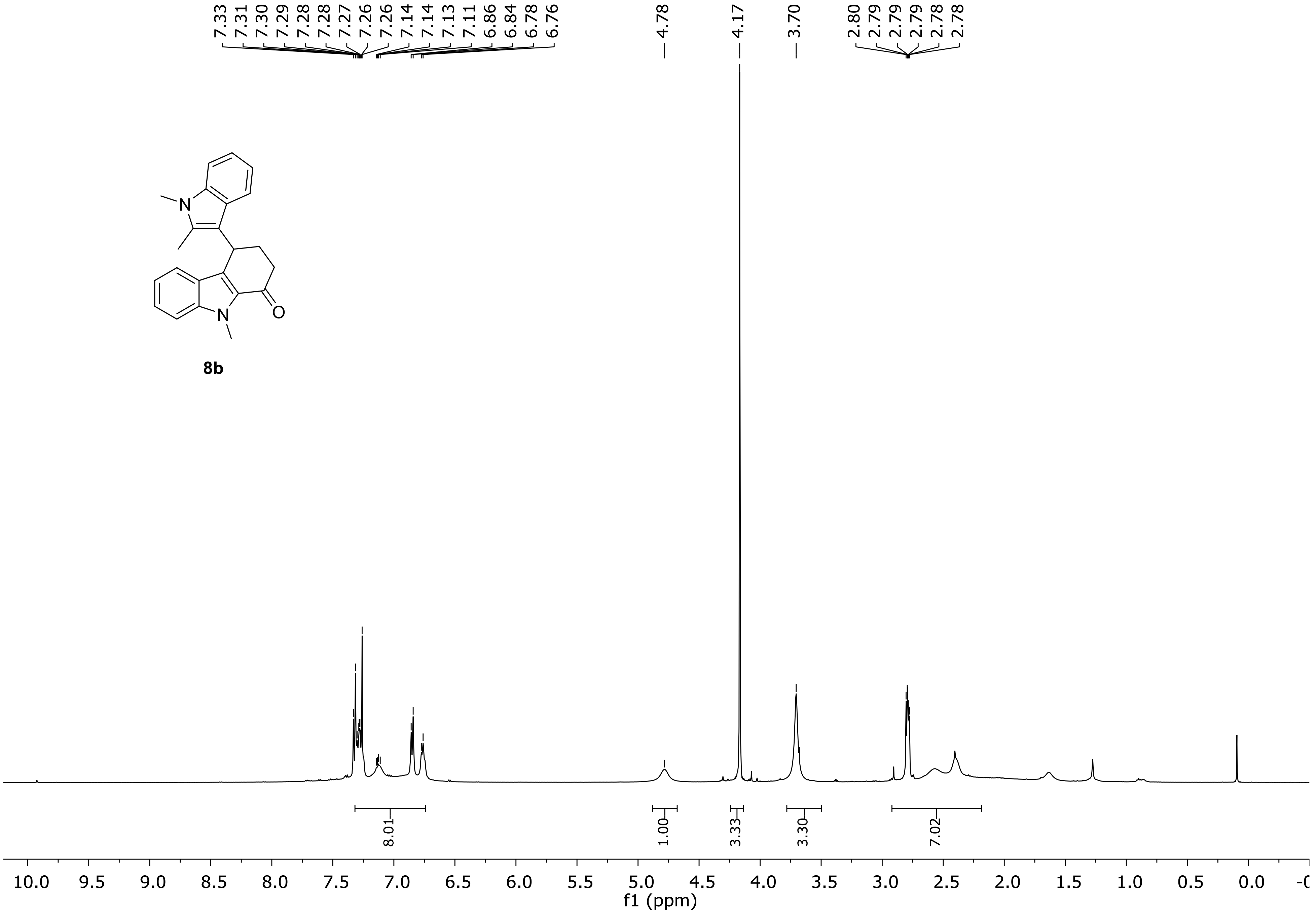

^{13}C-{^{1}H}NMR (126 MHz, $CDCl_3$)

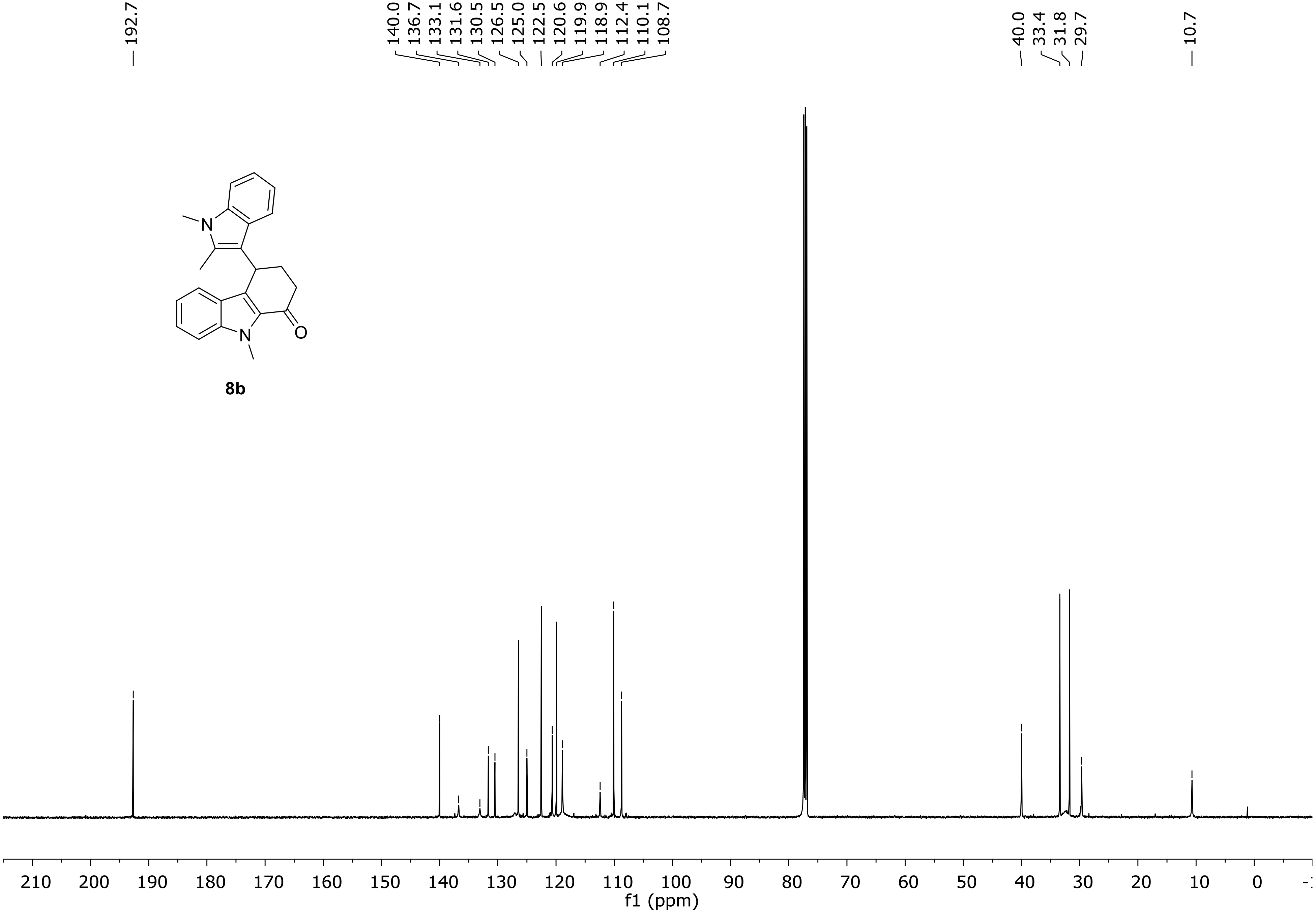

8b

^{1}H-NMR (75.4 MHz, DMSO-d$_6$)

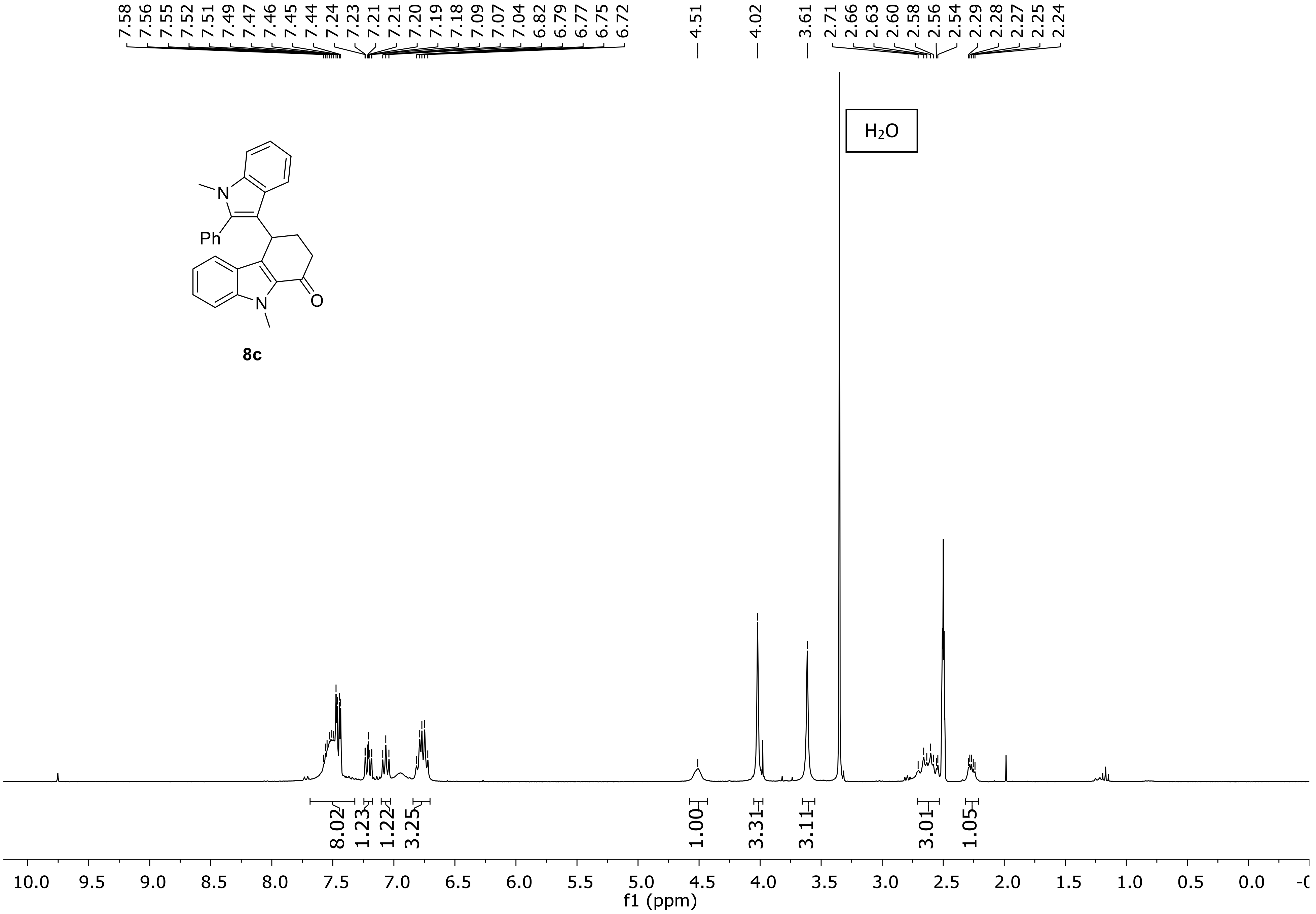

^{13}C-{^{1}H}NMR (75.4 MHz, DMSO-d_6)

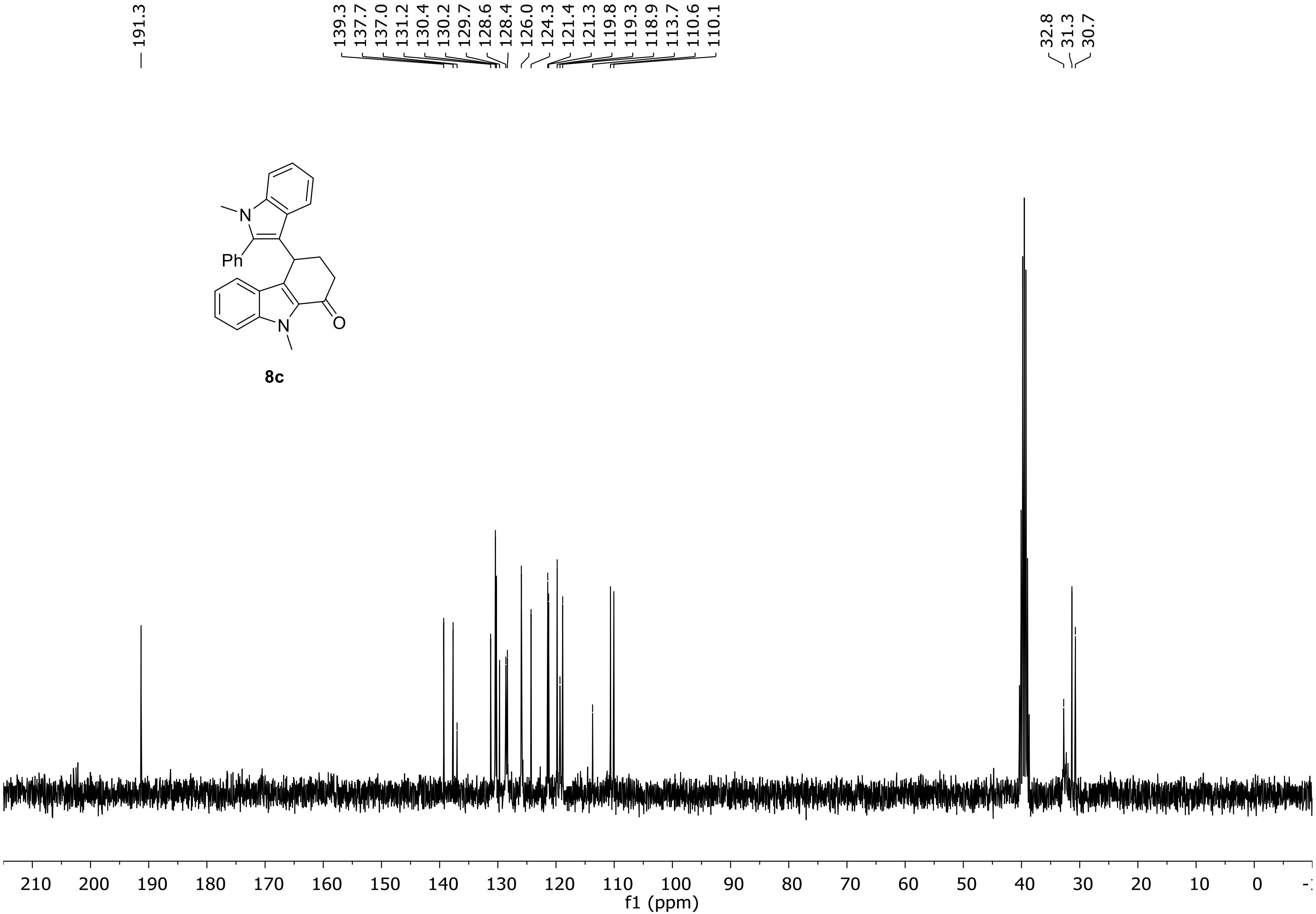

^{1}H-NMR (300 MHz, $CDCl_3$)

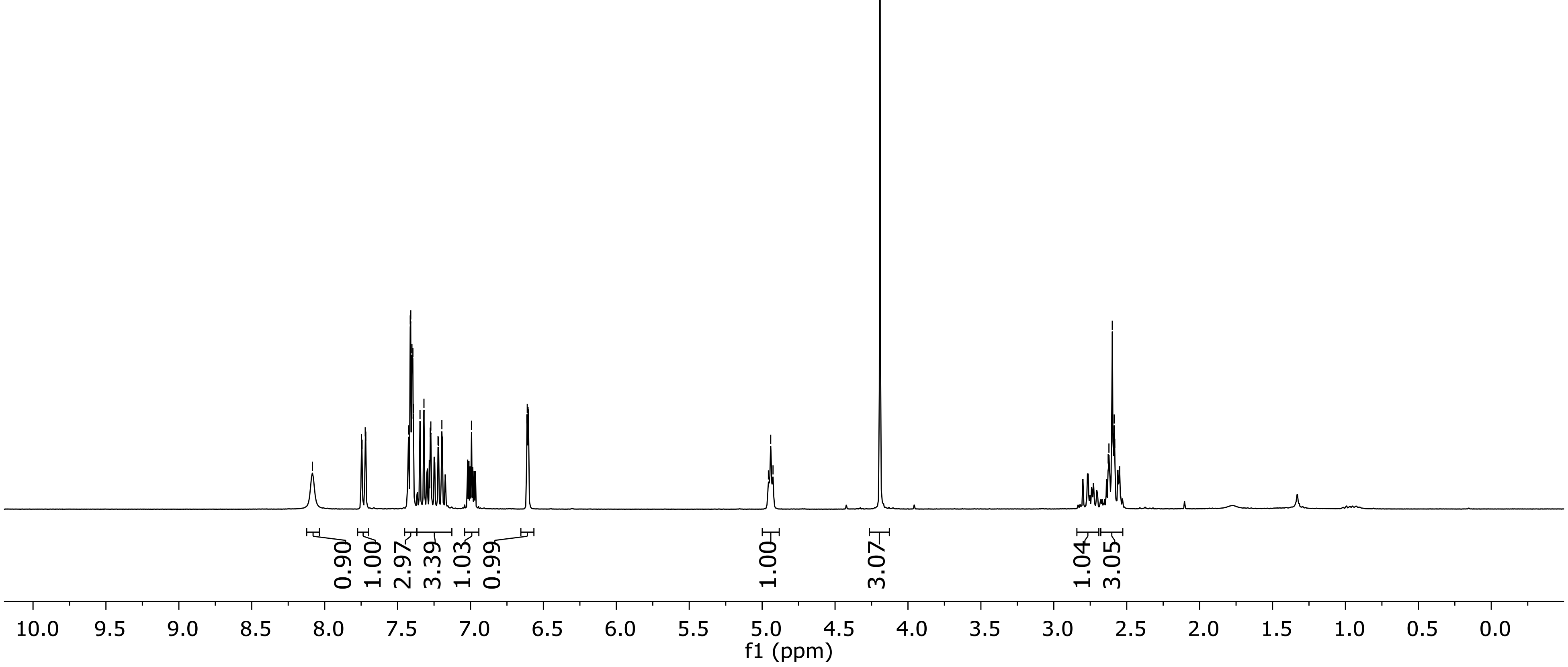

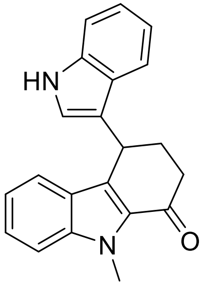

8d

$^{13}C-\{^{1}H\}$NMR (75.4 MHz, $CDCl_3$)

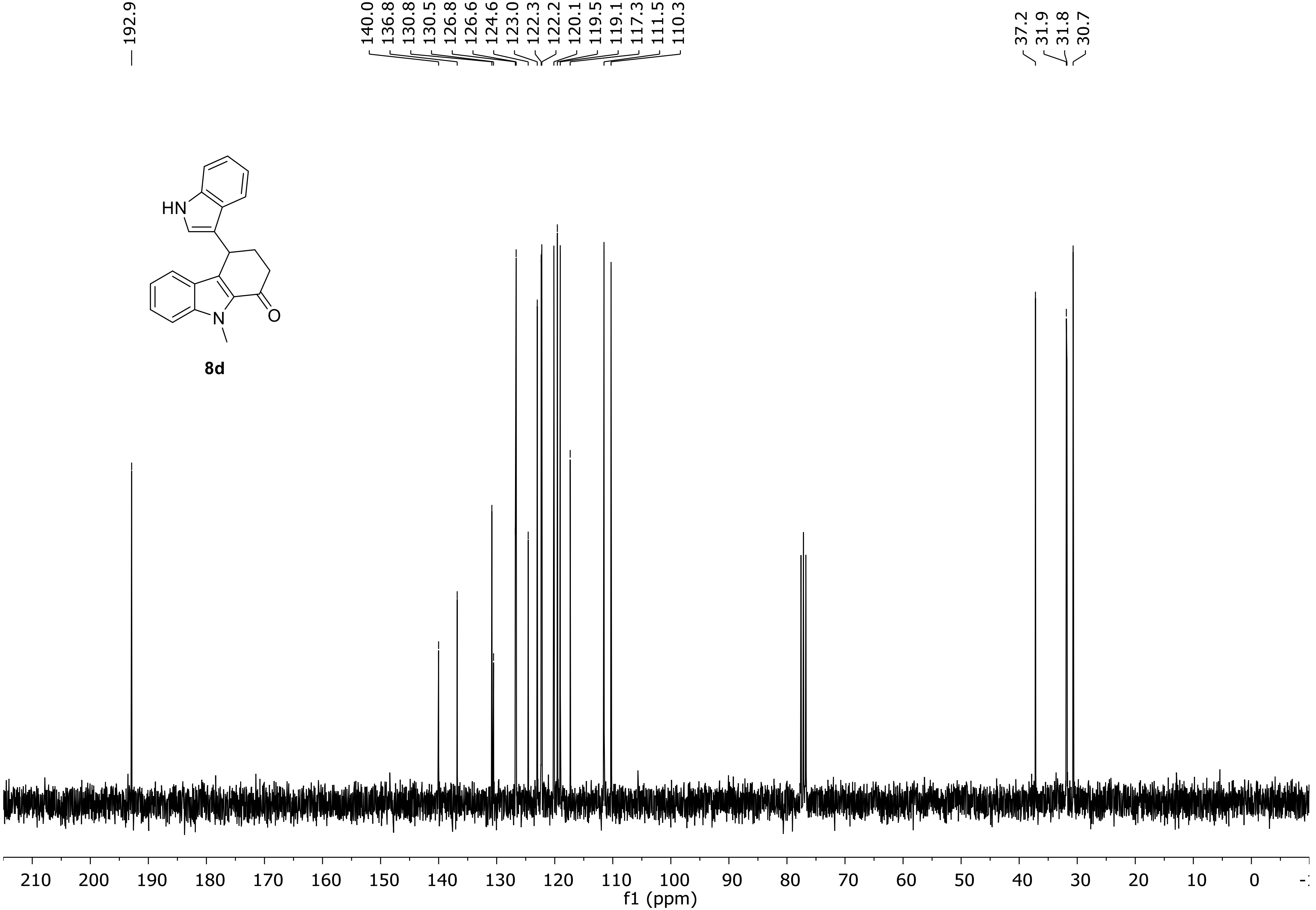

^{1}H-NMR (75.4 MHz, DMSO-d$_6$)

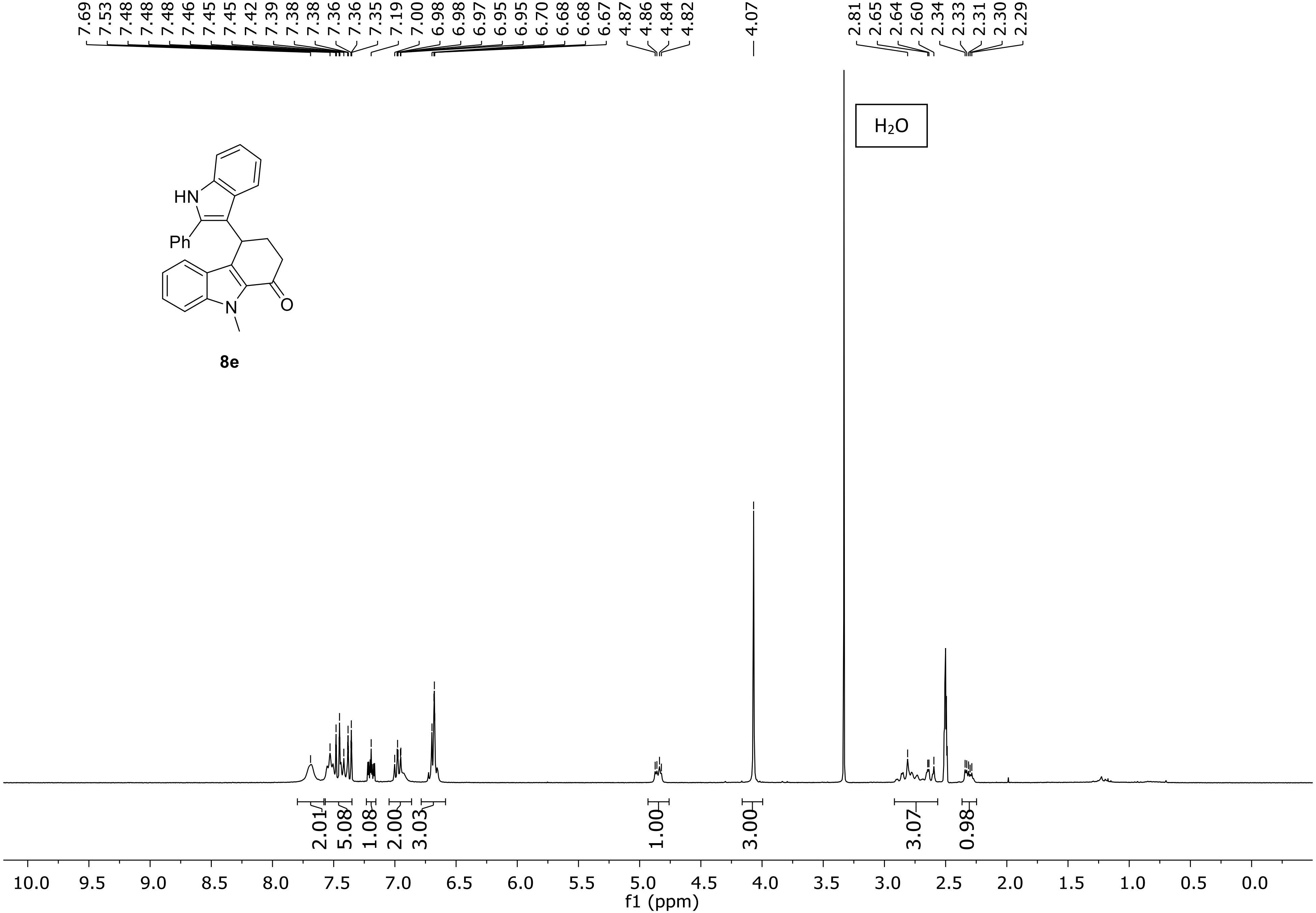

8e

^{13}C-{^{1}H}NMR (75.4 MHz, DMSO-d_6)

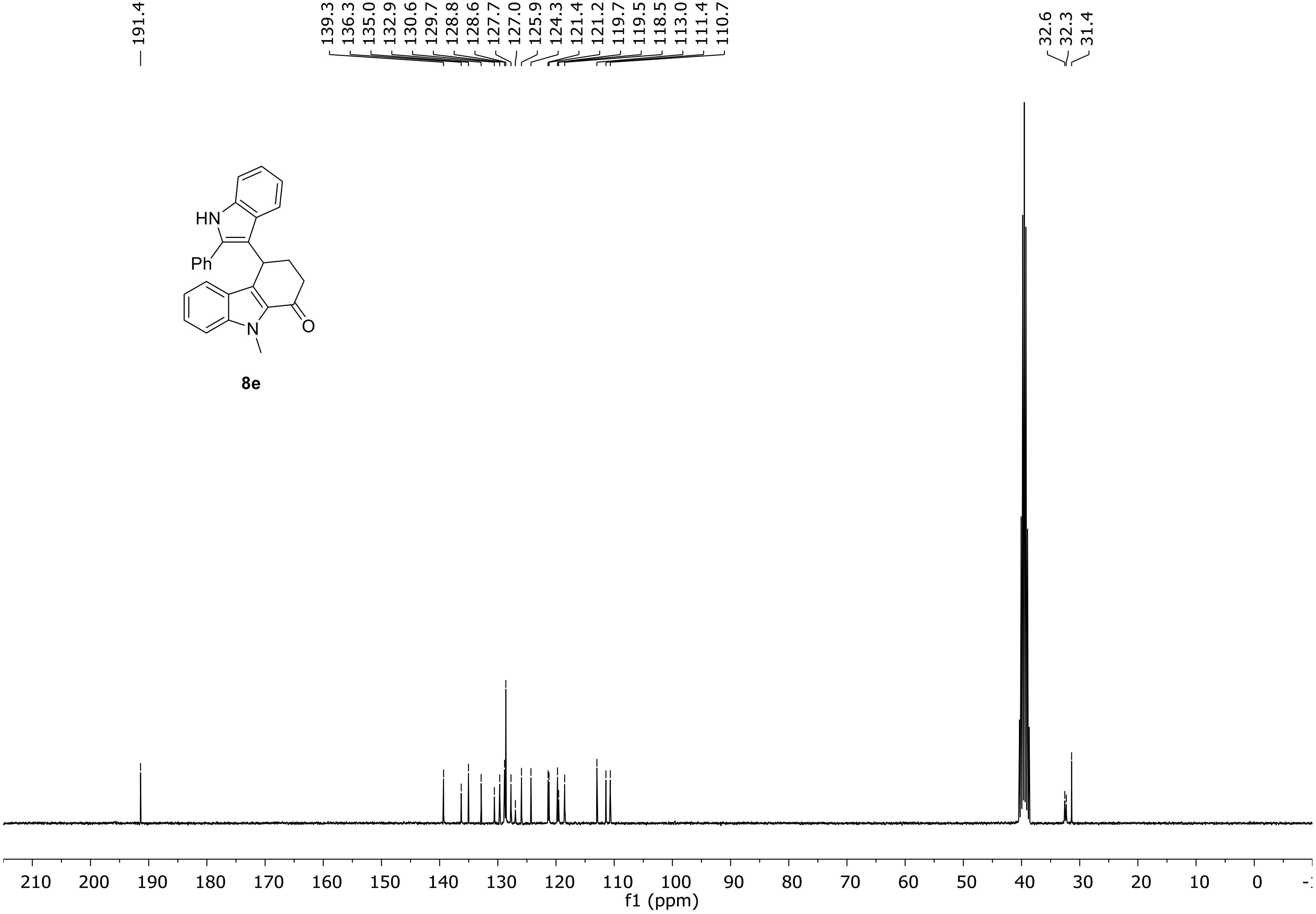

^{1}H-NMR (300 MHz, $CDCl_3$)

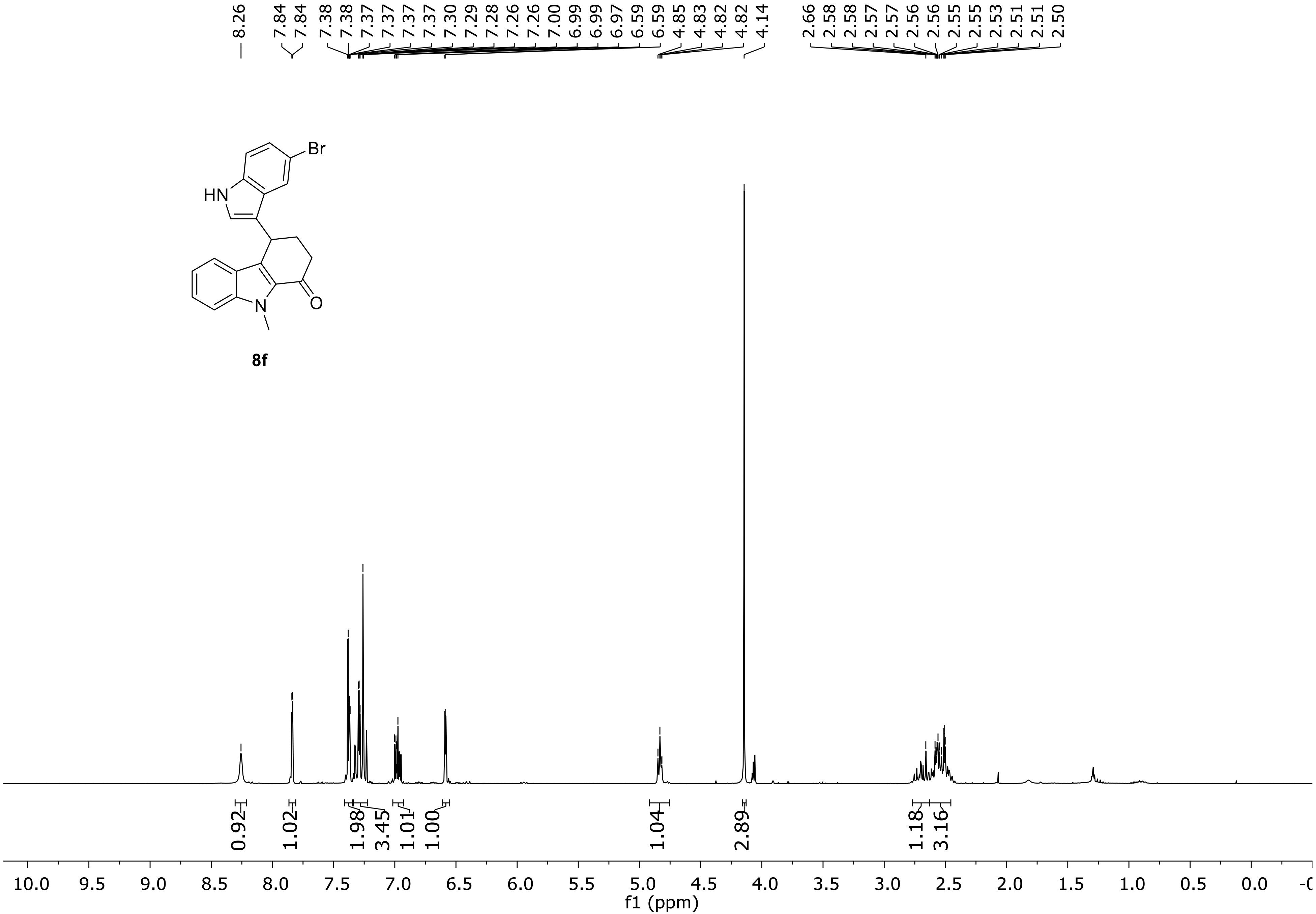

$^{13}C-\{^{1}H\}$NMR (75.4 MHz, $CDCl_3$)

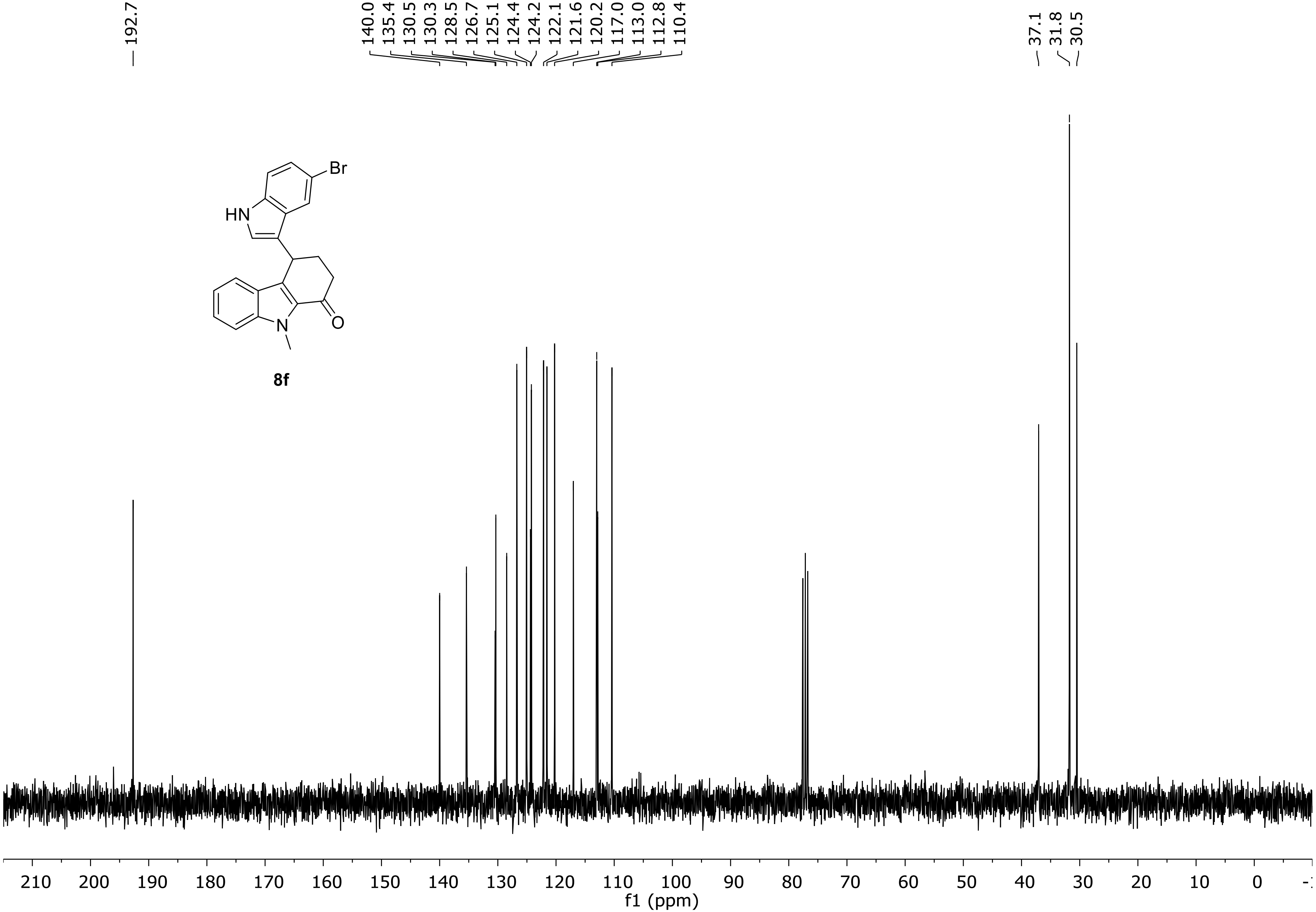

8f

^{1}H-NMR (300 MHz, $CDCl_3$)

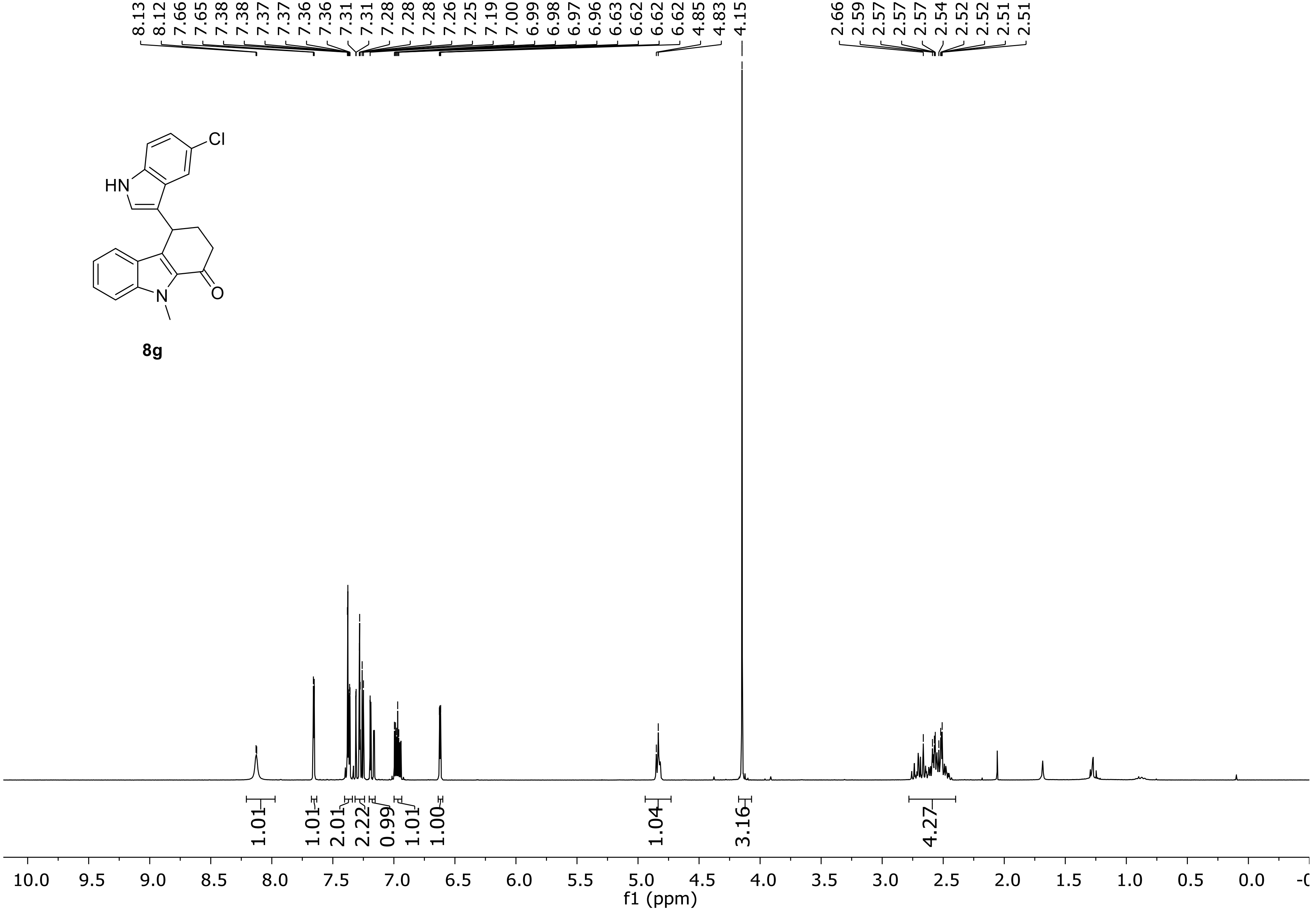

^{13}C-{^{1}H}NMR (75.4 MHz, $CDCl_3$)

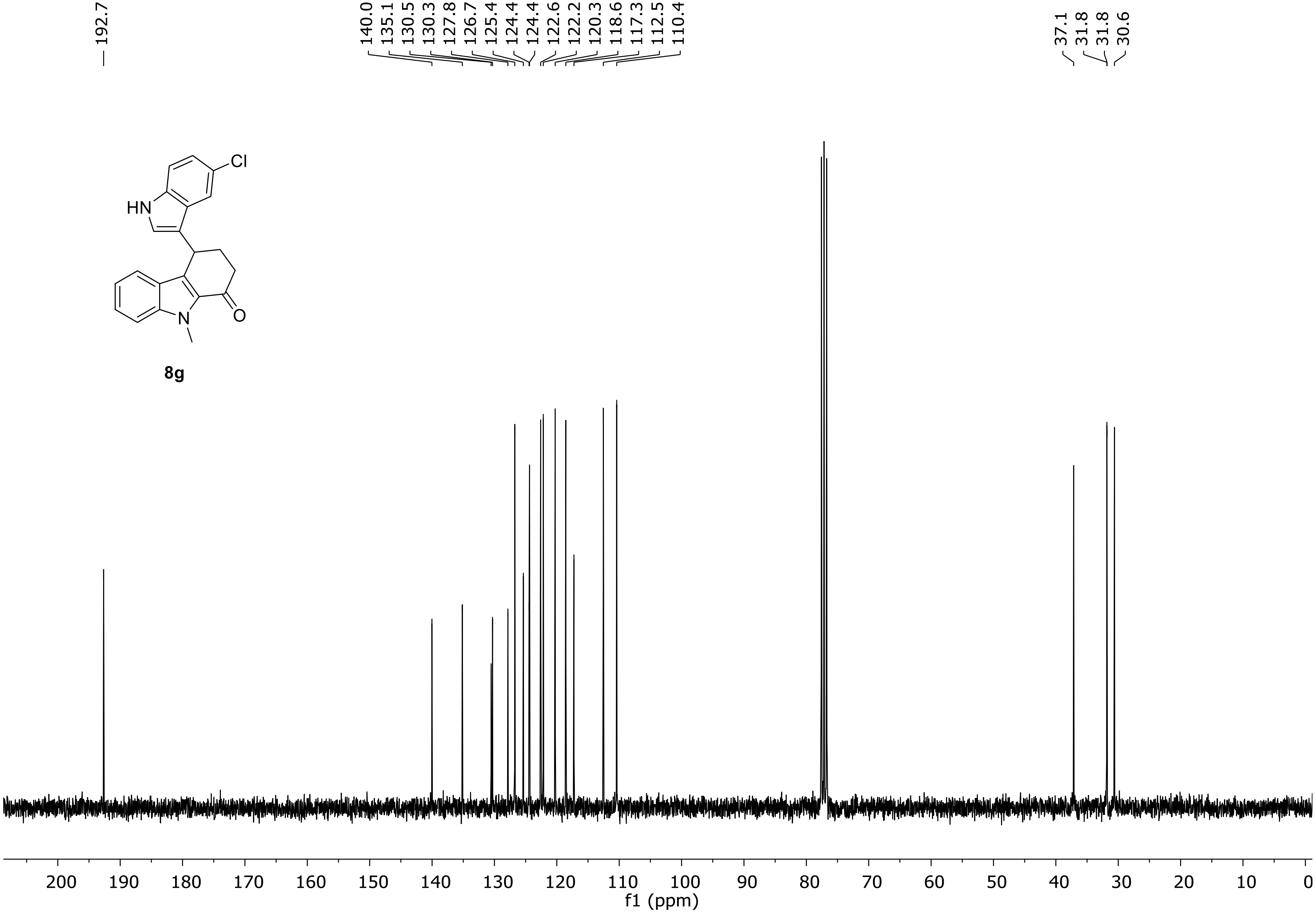

^{1}H-NMR (300 MHz, $CDCl_3$)

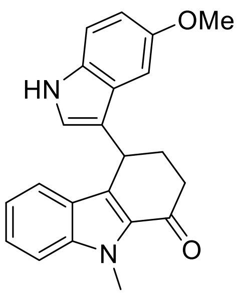

8h

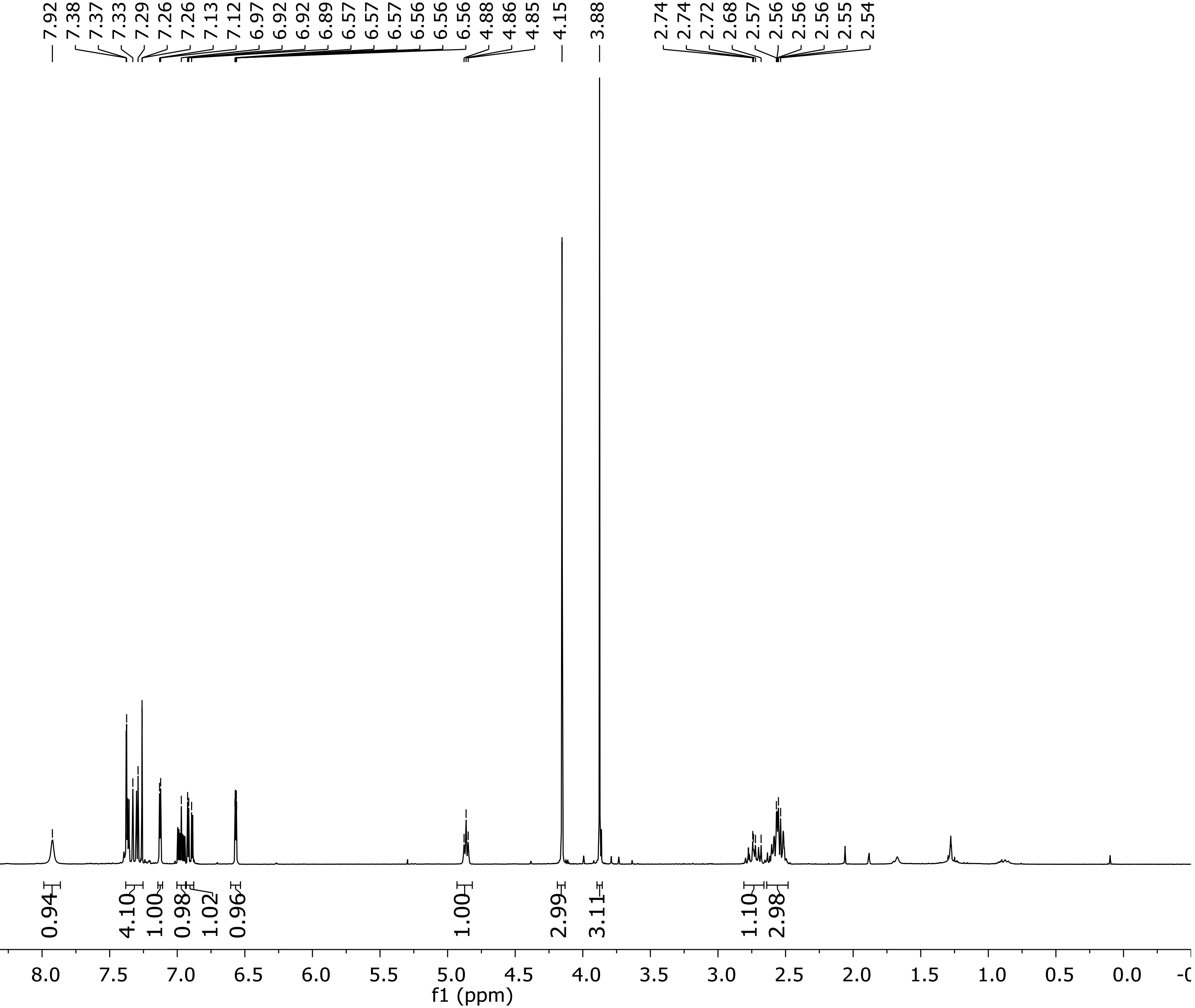

^{13}C-{^{1}H}NMR (75.4 MHz, $CDCl_3$)

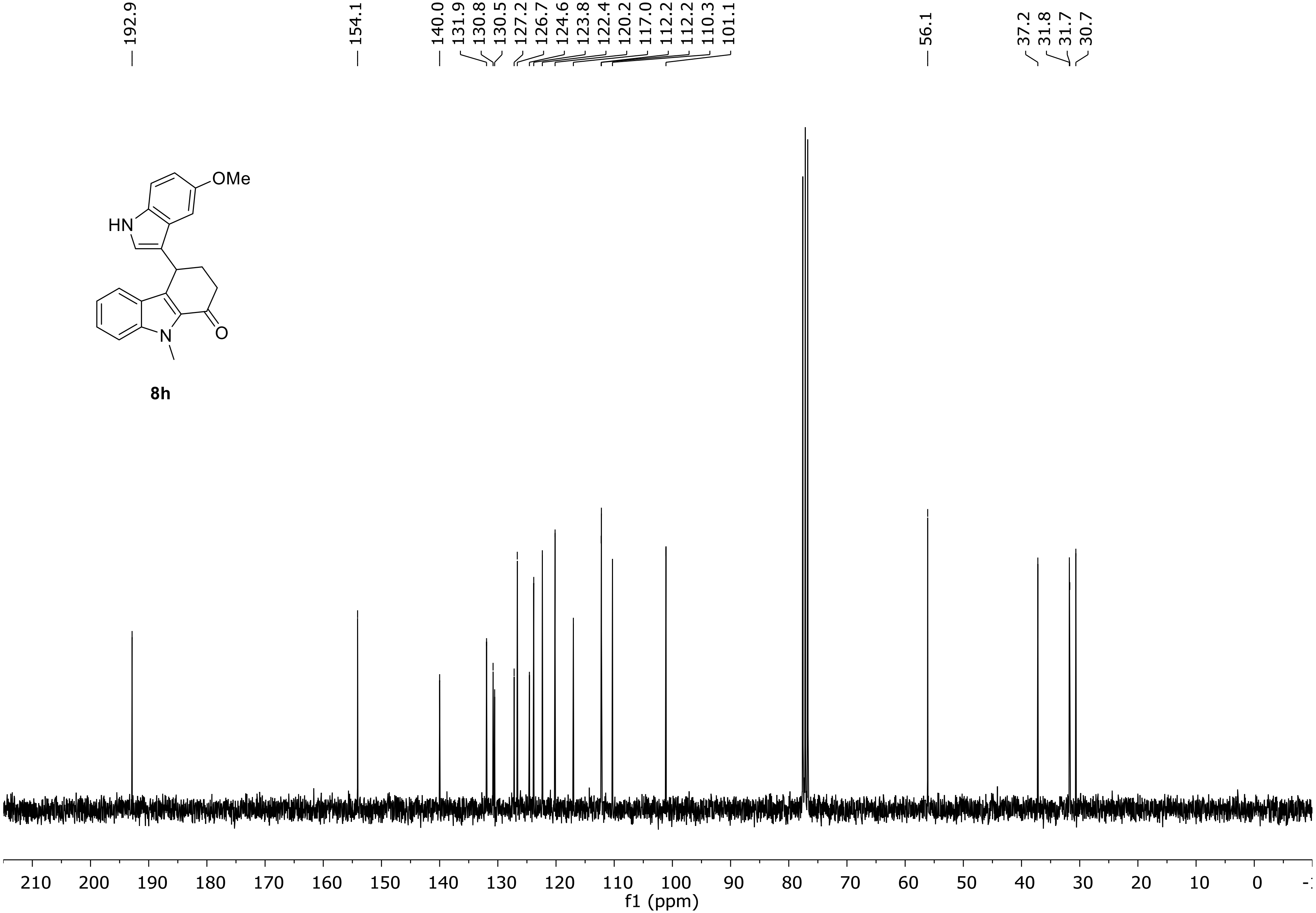

^{1}H-NMR (300 MHz, $CDCl_3$)

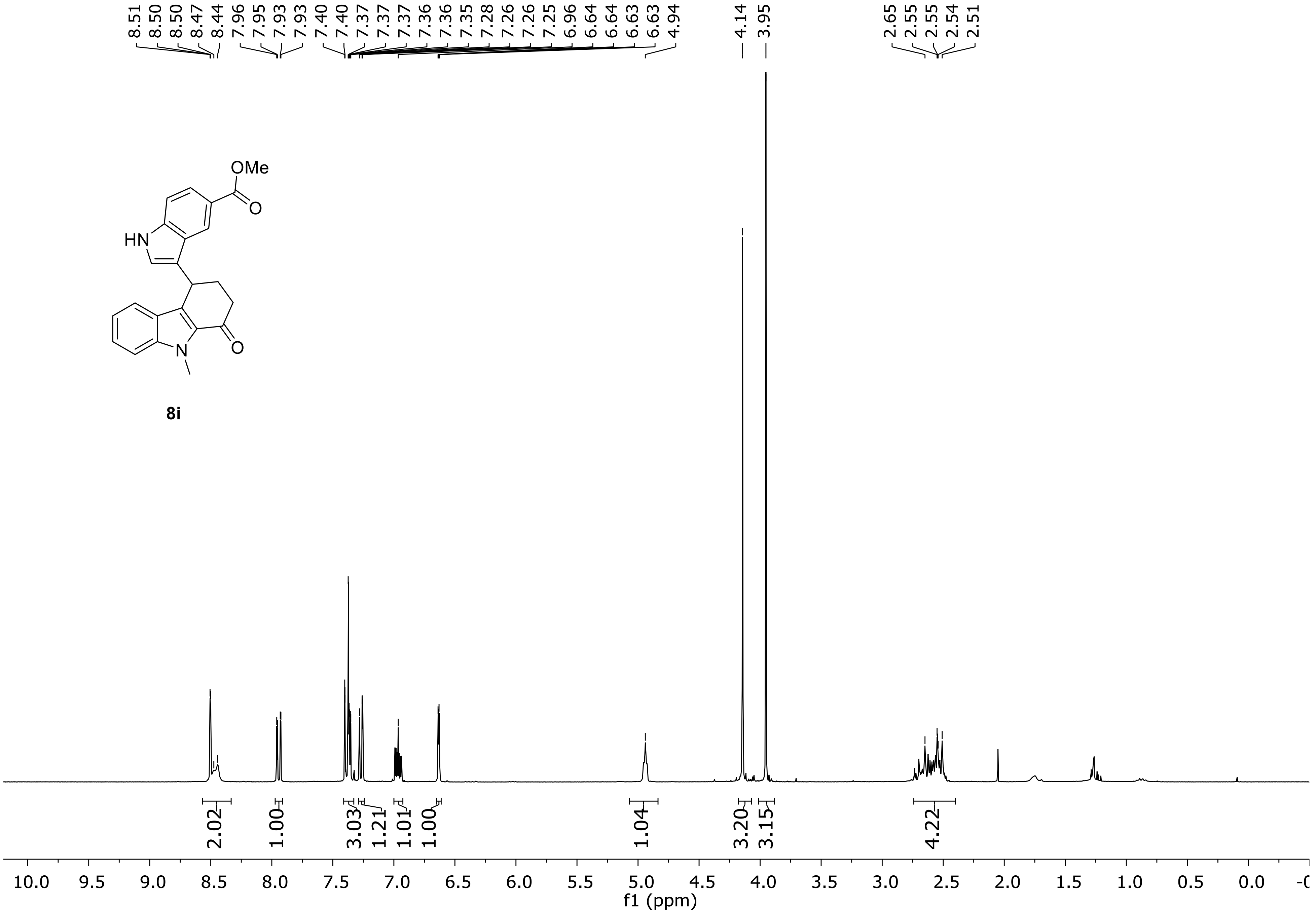

8i

$^{13}C-\{^1H\}$NMR (75.4 MHz, $CDCl_3$)

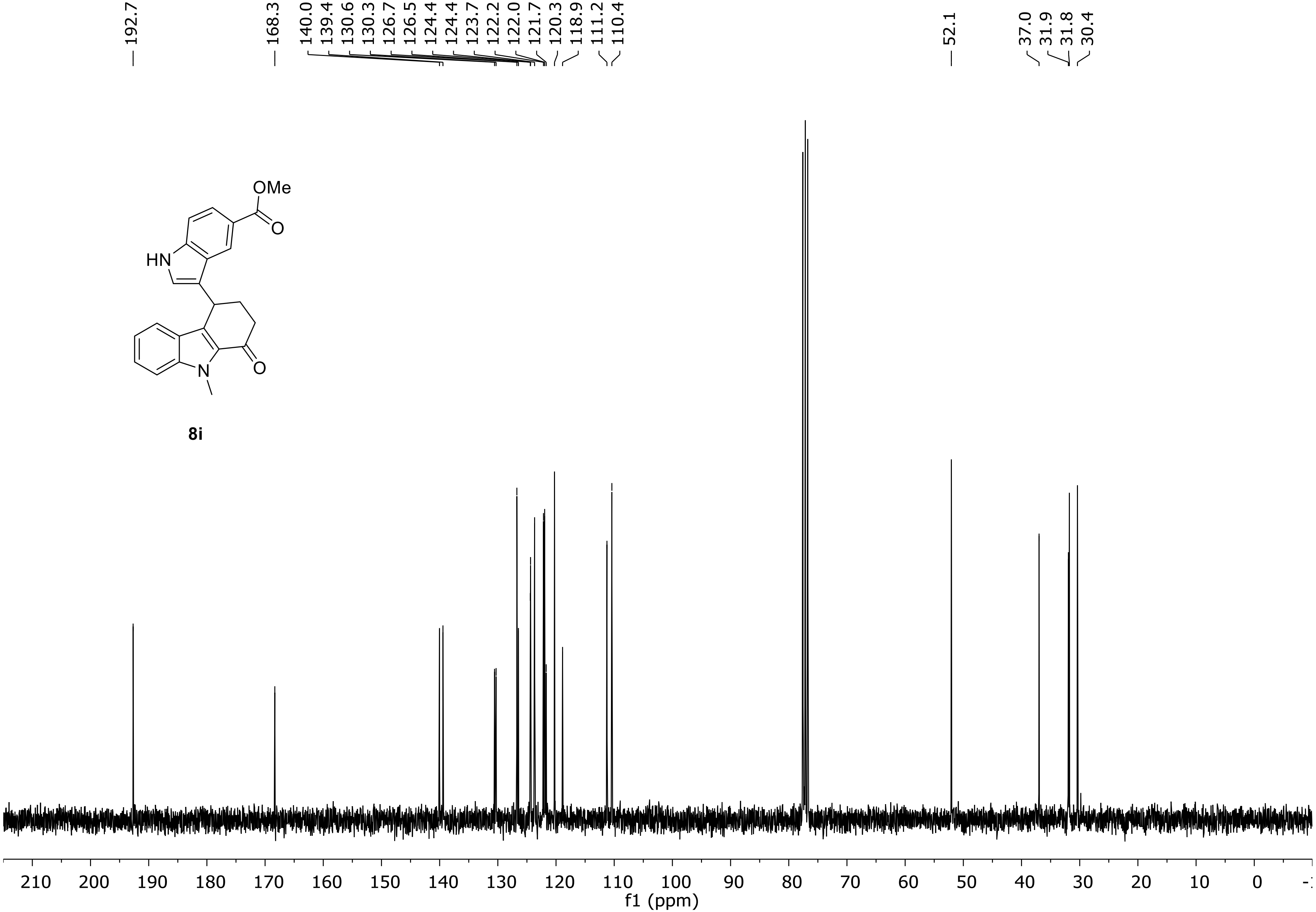

^{1}H-NMR (300 MHz, $CDCl_3$)

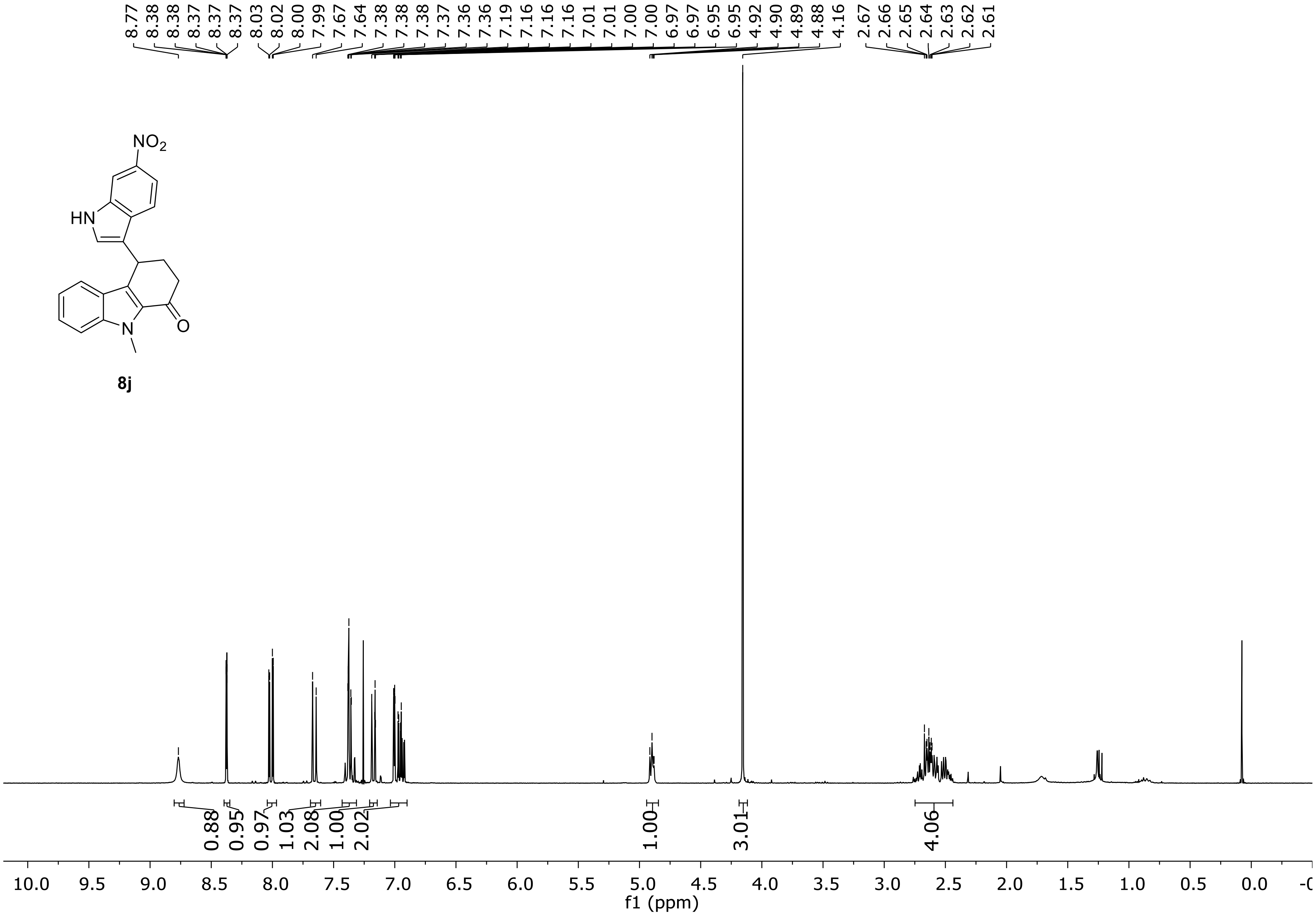

8j

$^{13}C-\{^{1}H\}$NMR (75.4 MHz, $CDCl_3$)

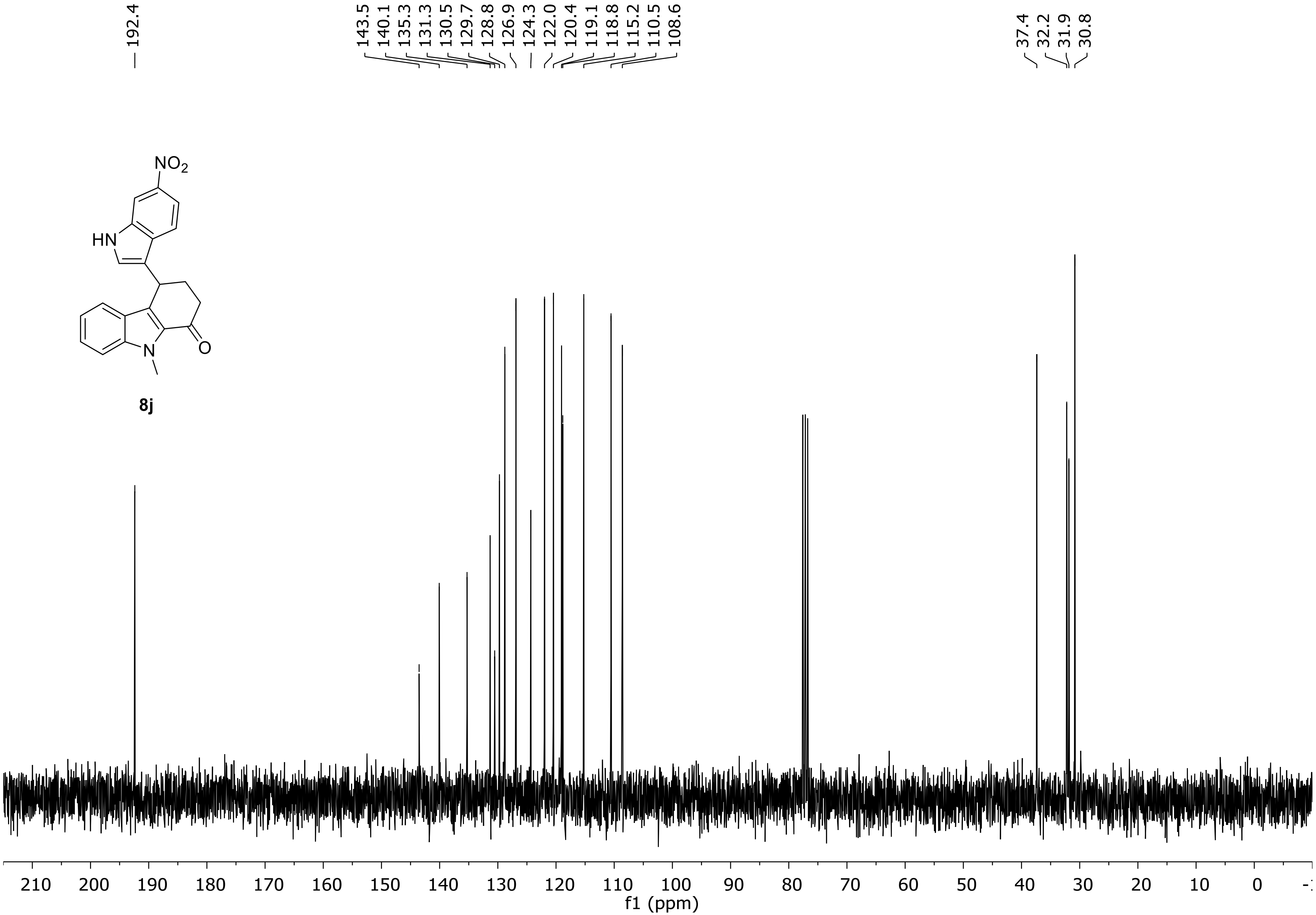

^{1}H-NMR (300 MHz, $CDCl_3$)

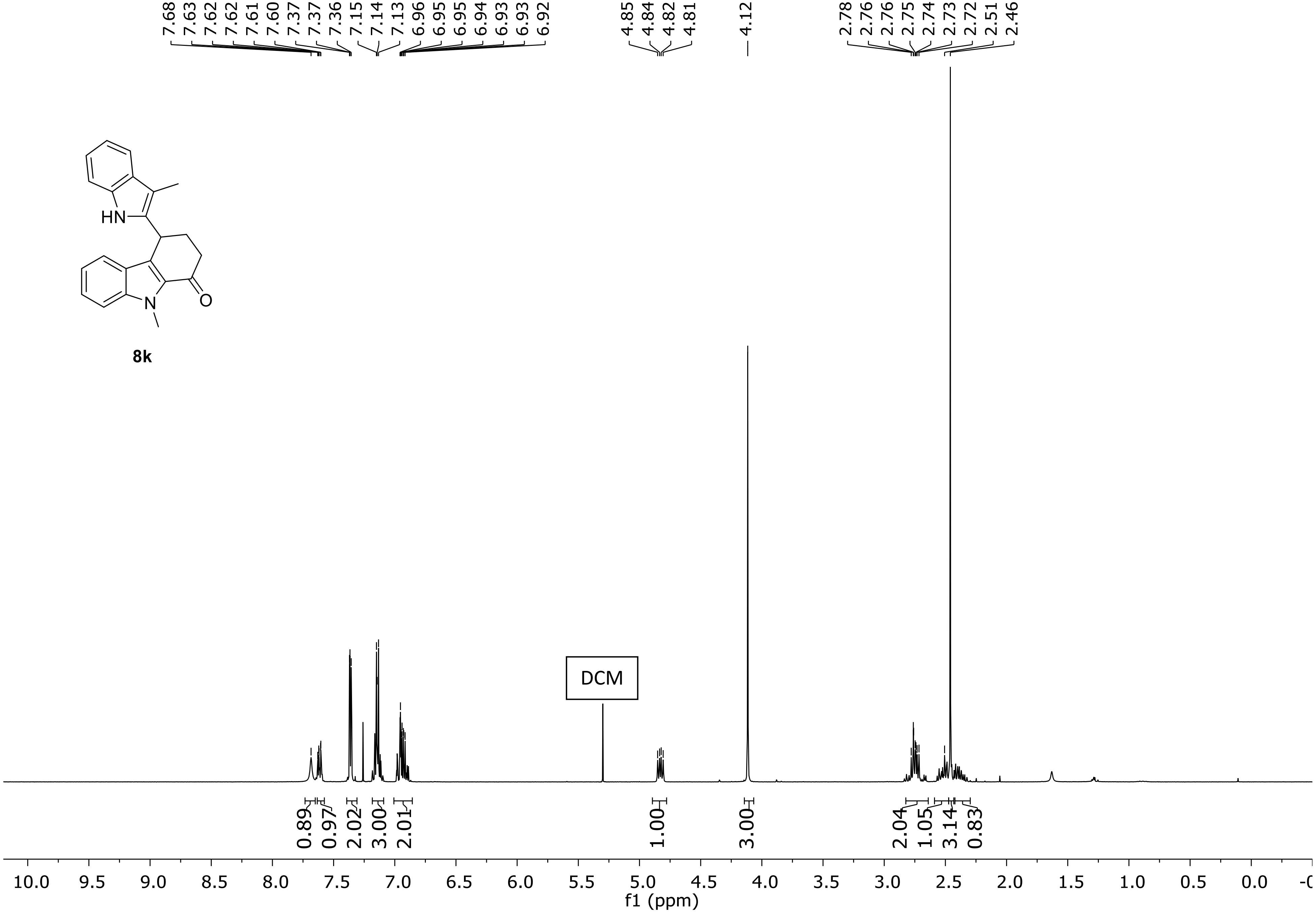

8k

^{13}C-{^{1}H}NMR (75.4 MHz, $CDCl_3$)

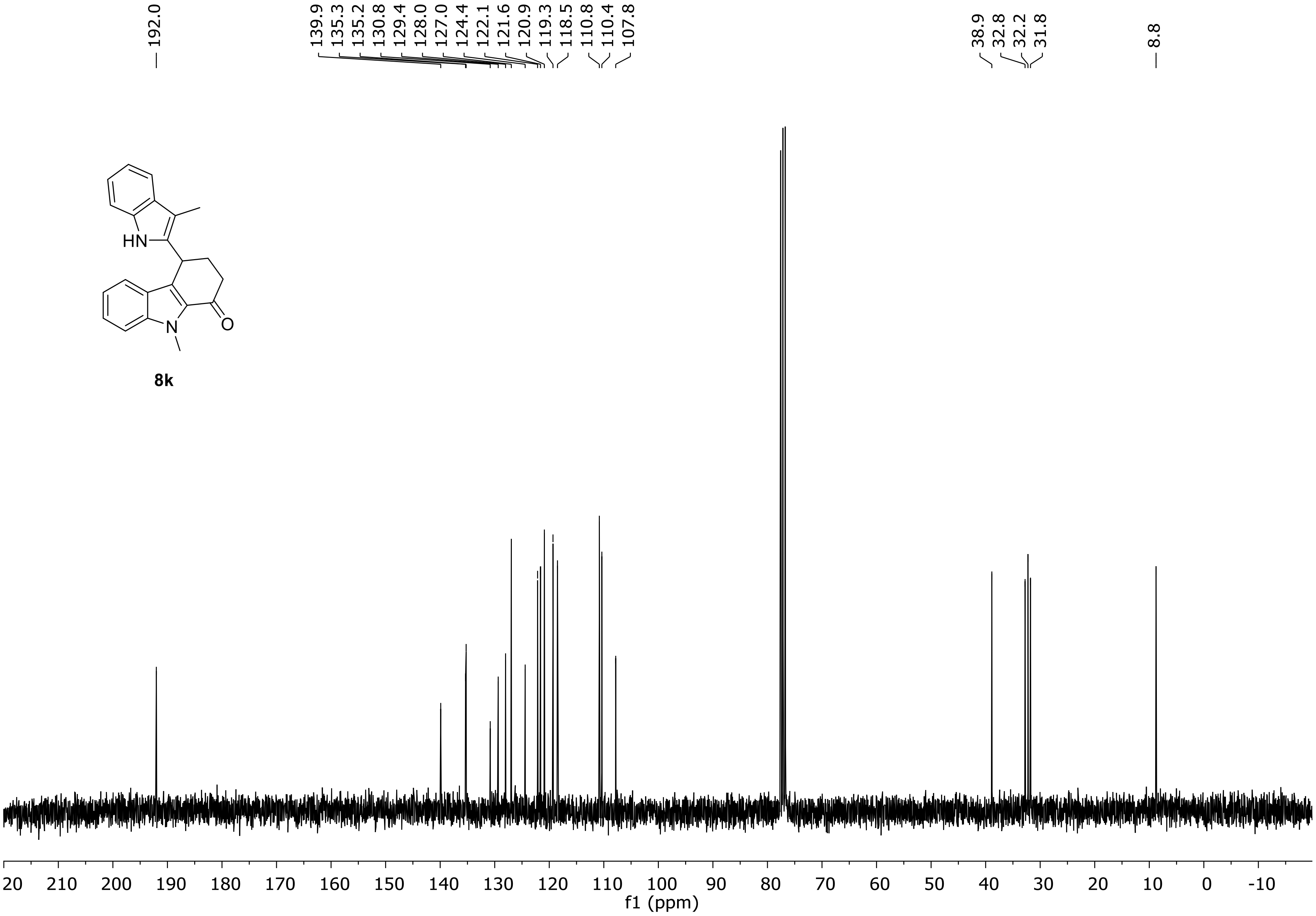

^{1}H-NMR (300 MHz, $CDCl_3$)

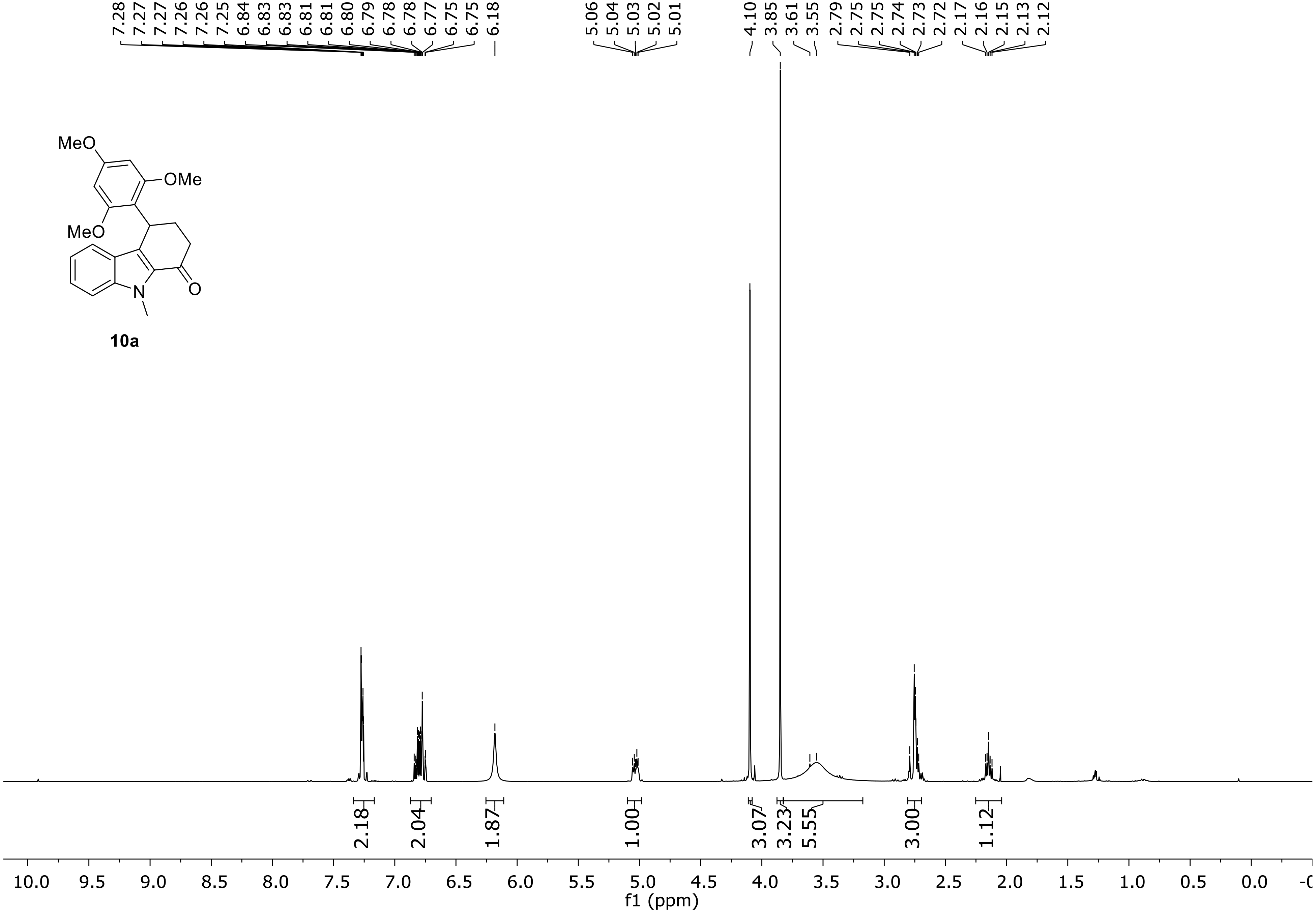

$^{13}C-\{^{1}H\}$NMR (75.4 MHz, $CDCl_3$)

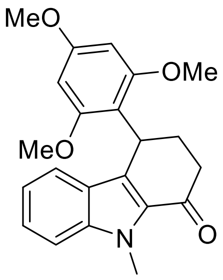

10a

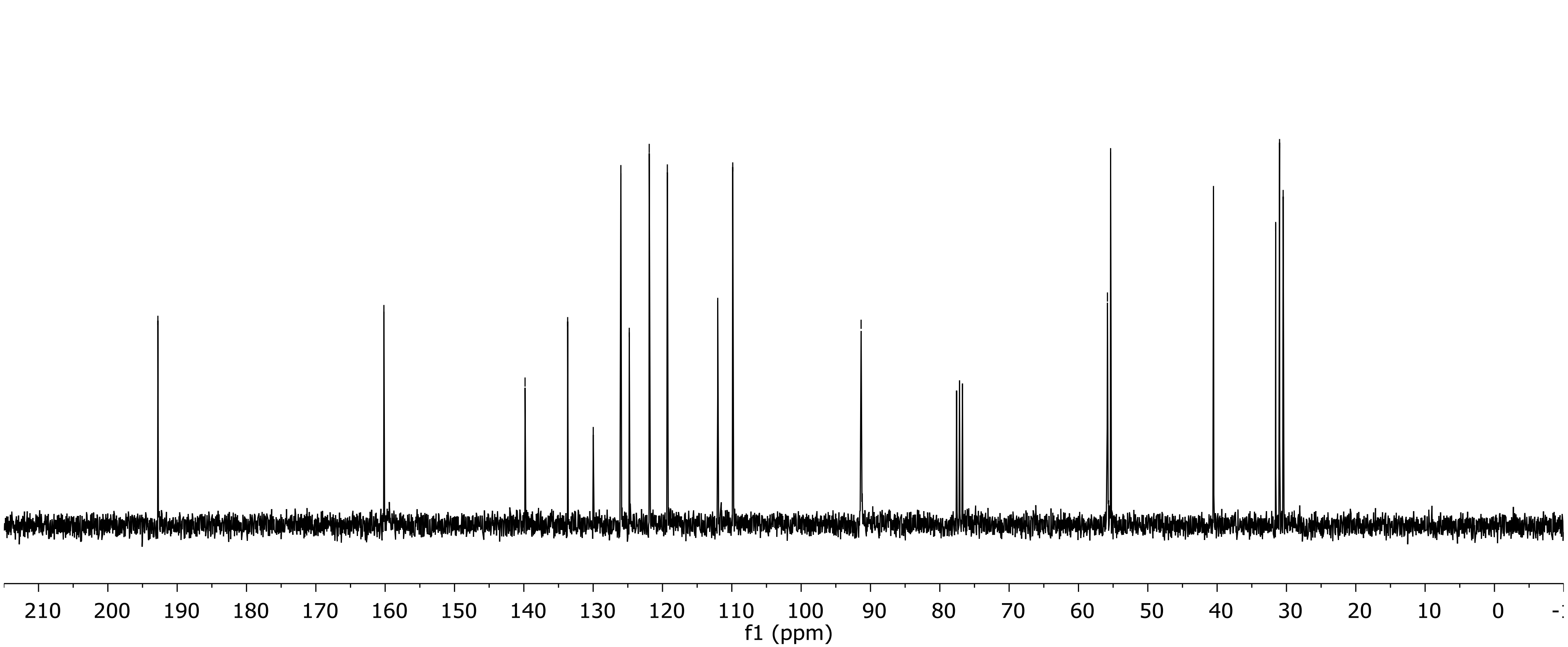

^{1}H-NMR (300 MHz, $CDCl_3$)

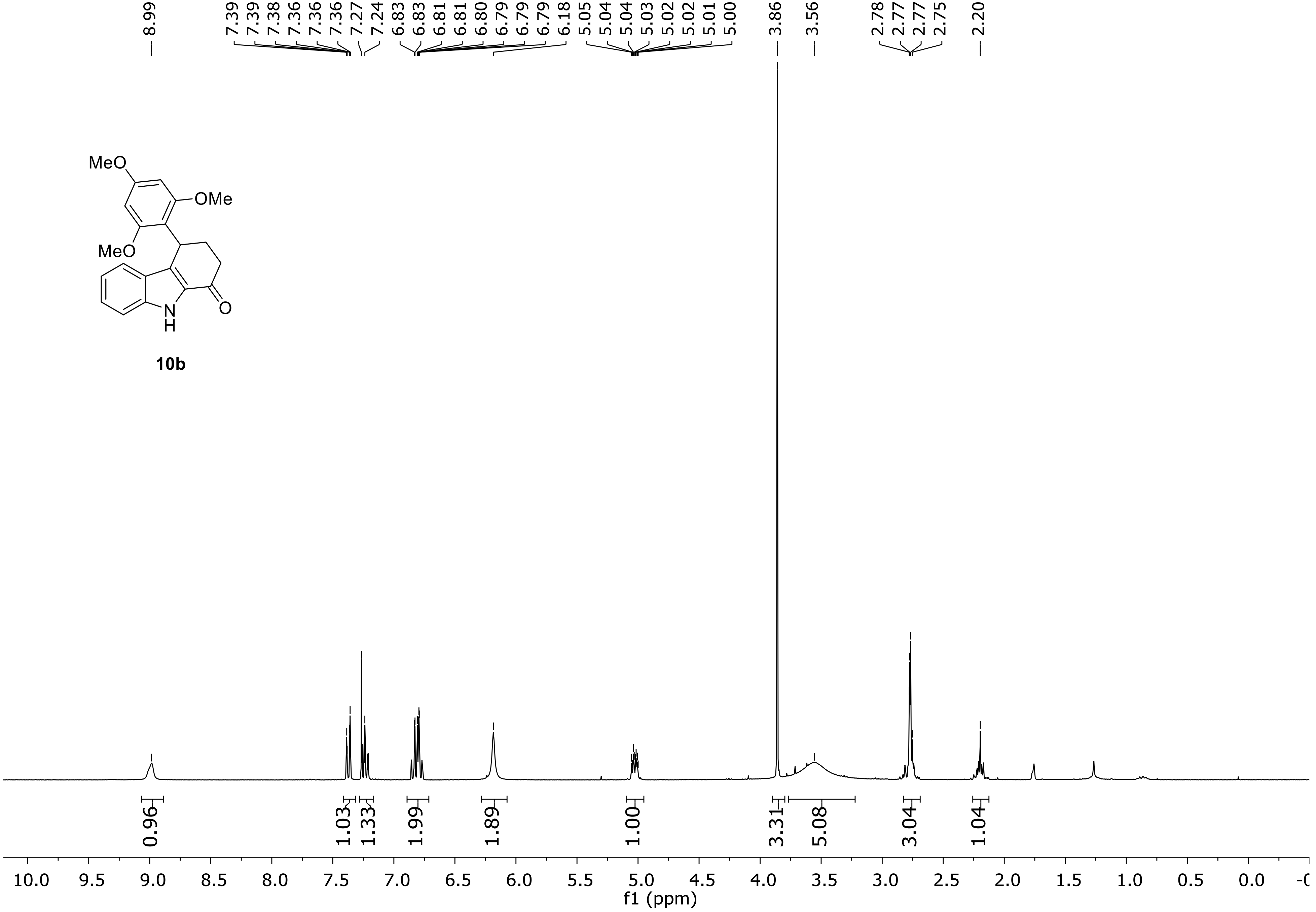

^{13}C-{^{1}H}NMR (75.4 MHz, $CDCl_3$)

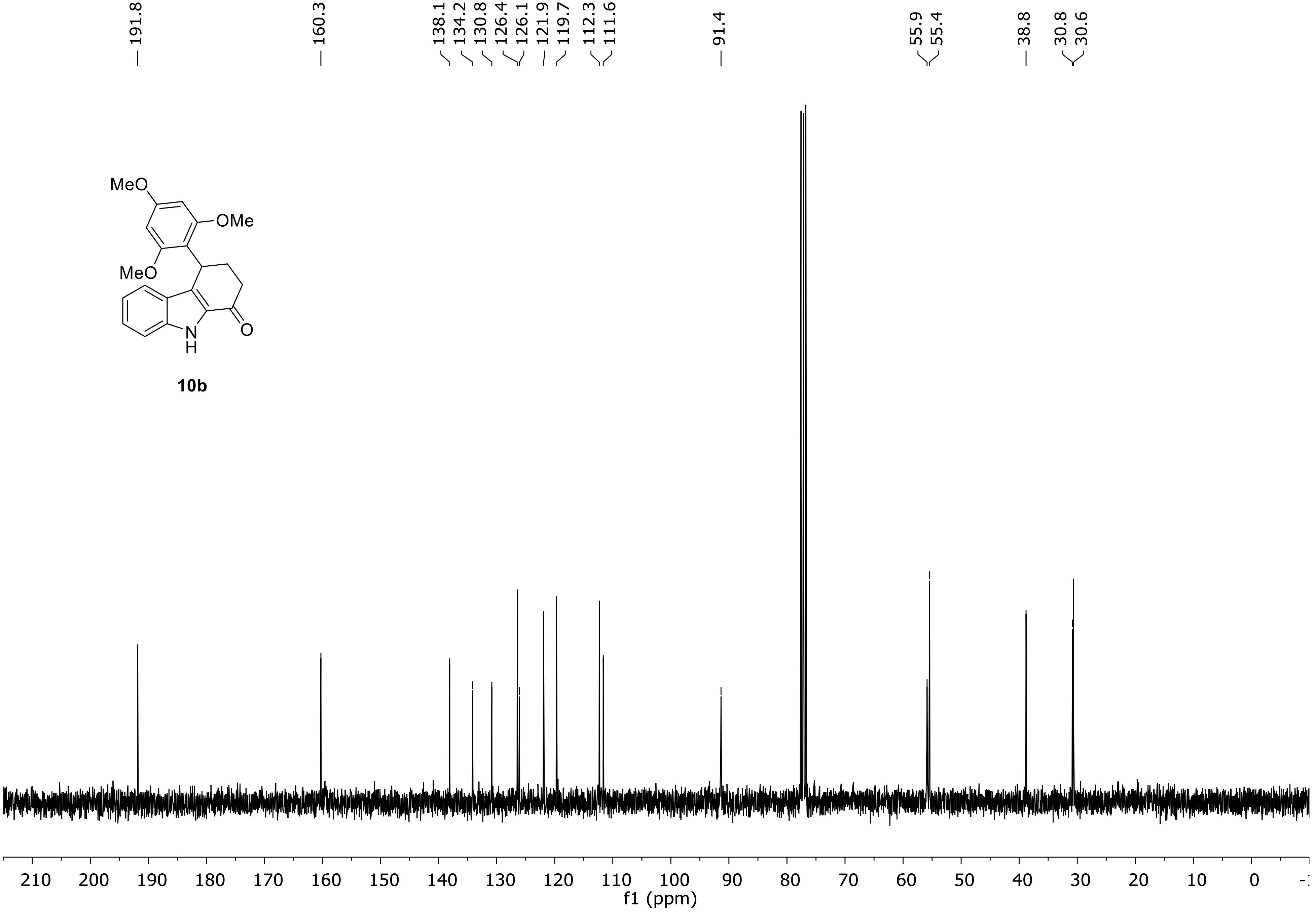

10b

^{1}H-NMR (300 MHz, $CDCl_3$)

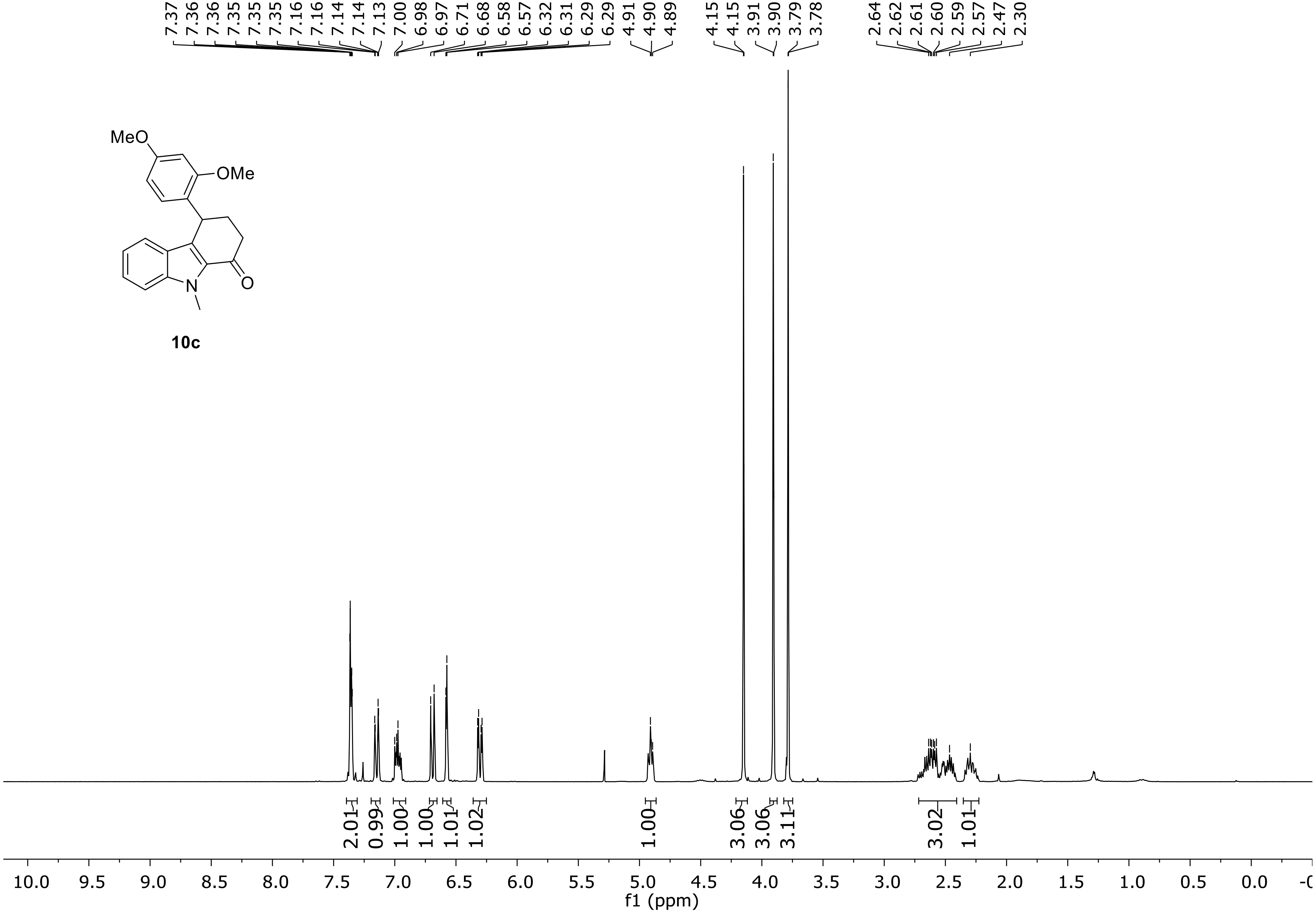

^{13}C-{^{1}H}NMR (75.4 MHz, $CDCl_3$)

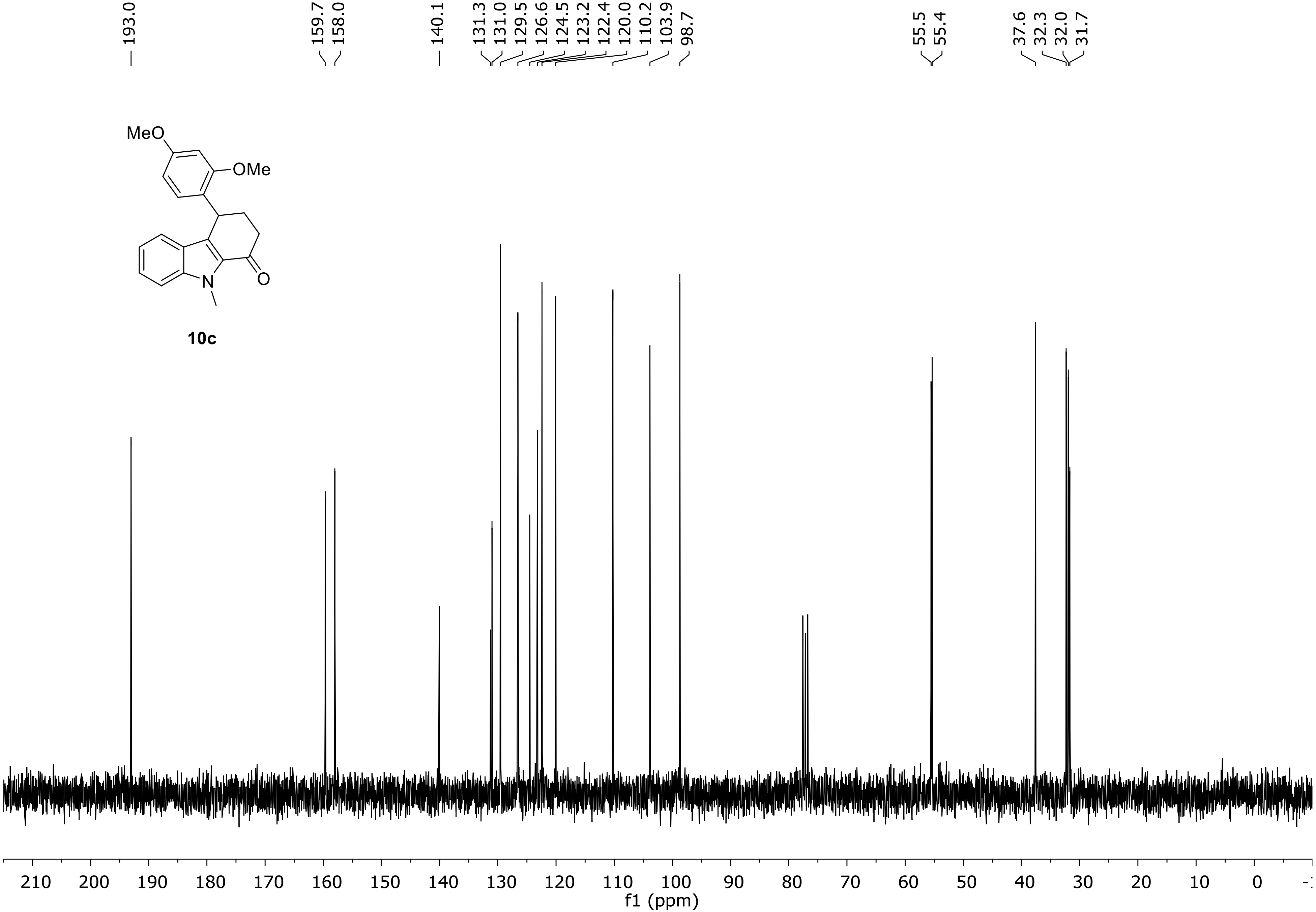

10c

^{1}H-NMR (300 MHz, $CDCl_3$)

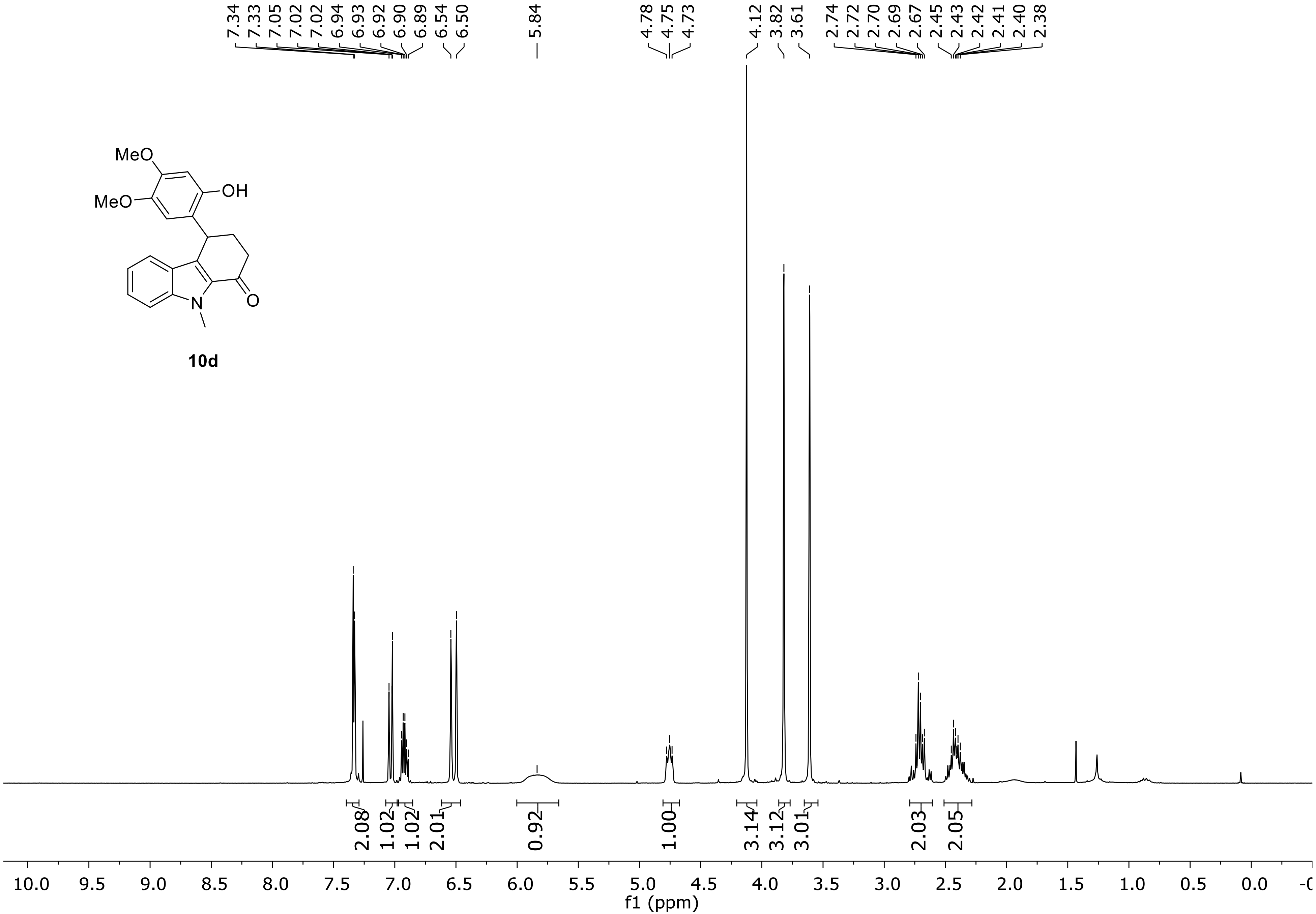

^{13}C-{^{1}H}NMR (75.4 MHz, $CDCl_3$)

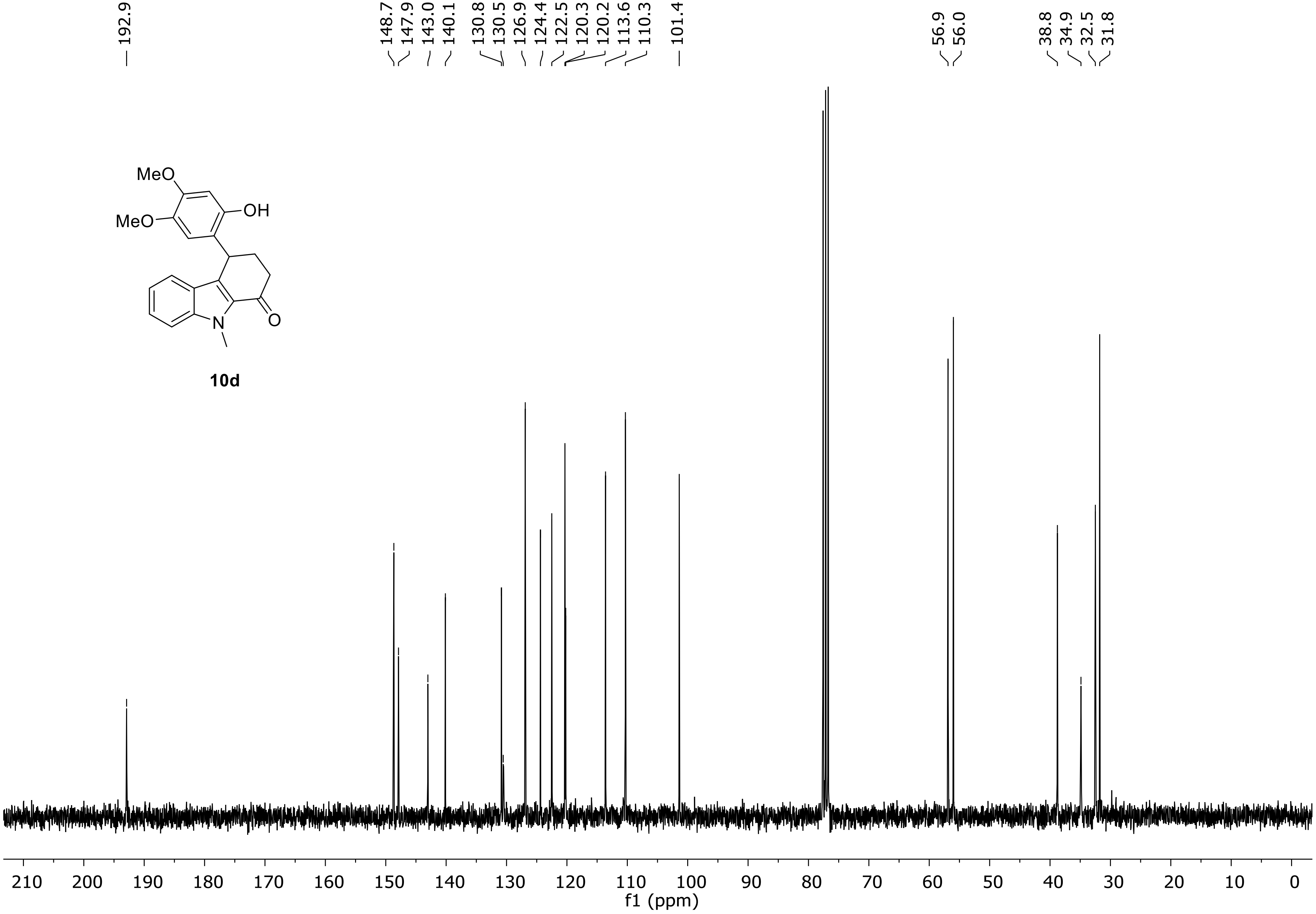

^{1}H-NMR (300 MHz, $CDCl_3$)

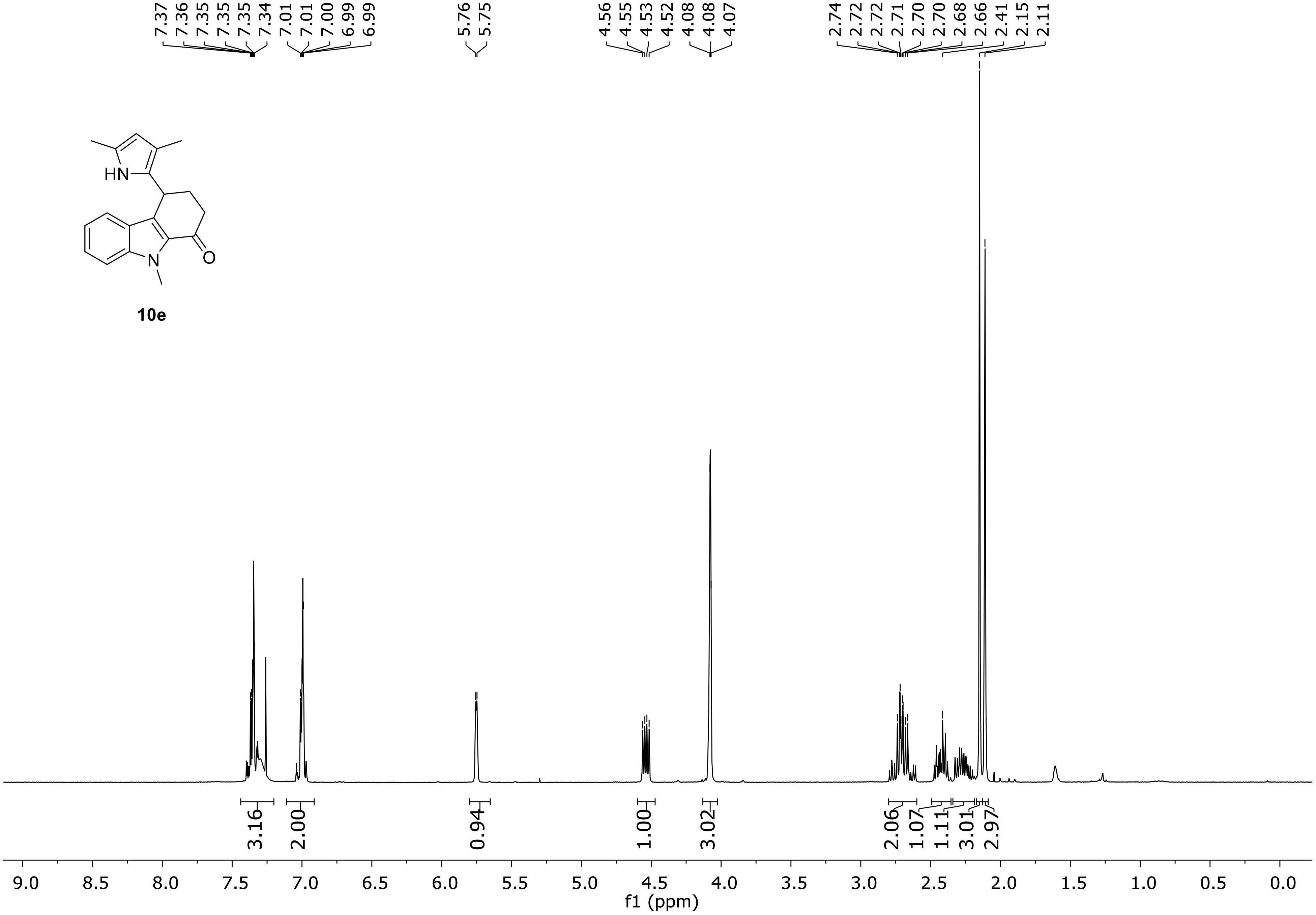

10e

$^{13}C-\{^1H\}$NMR (75.4 MHz, $CDCl_3$)

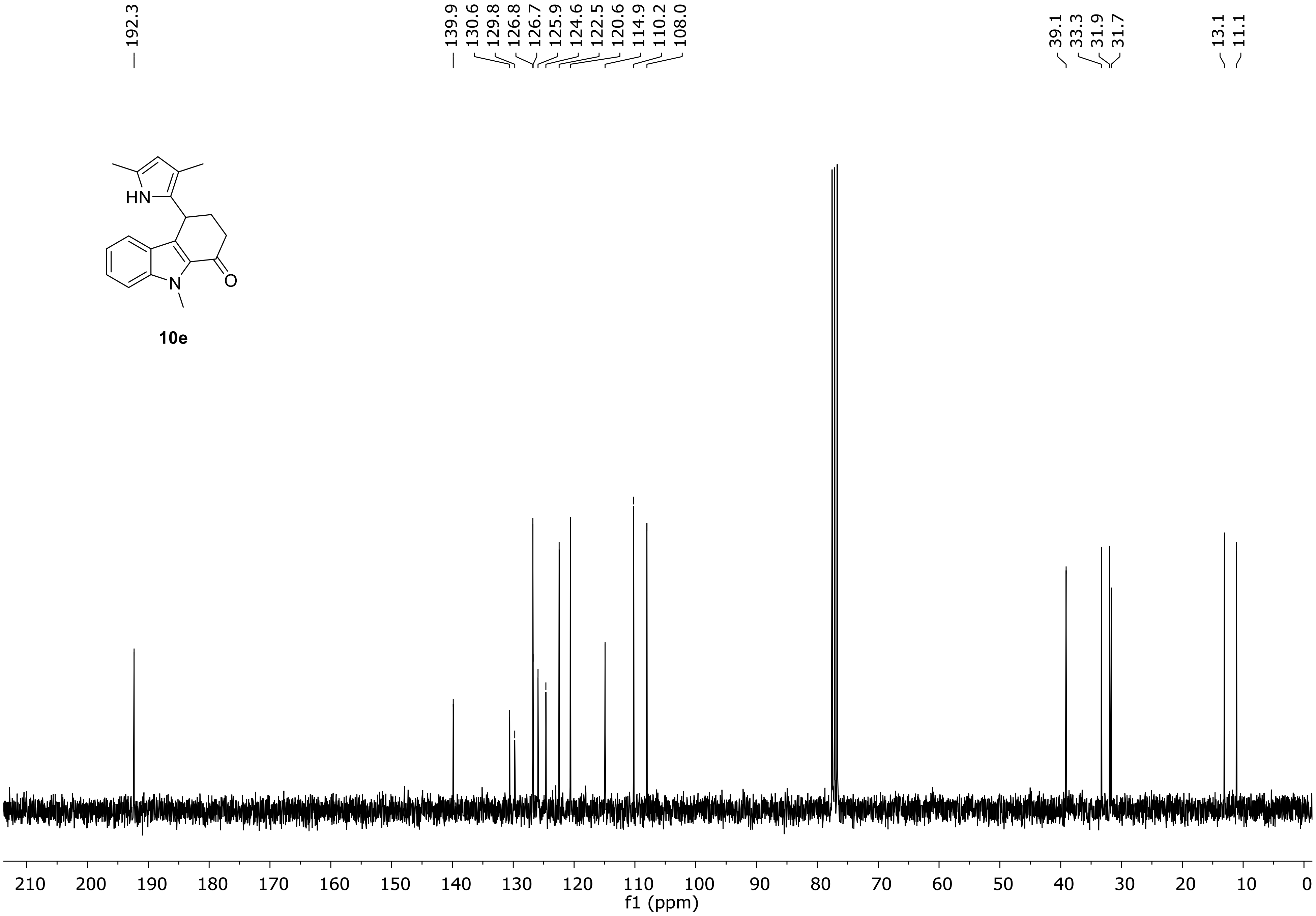

^{1}H-NMR (300 MHz, $CDCl_3$)

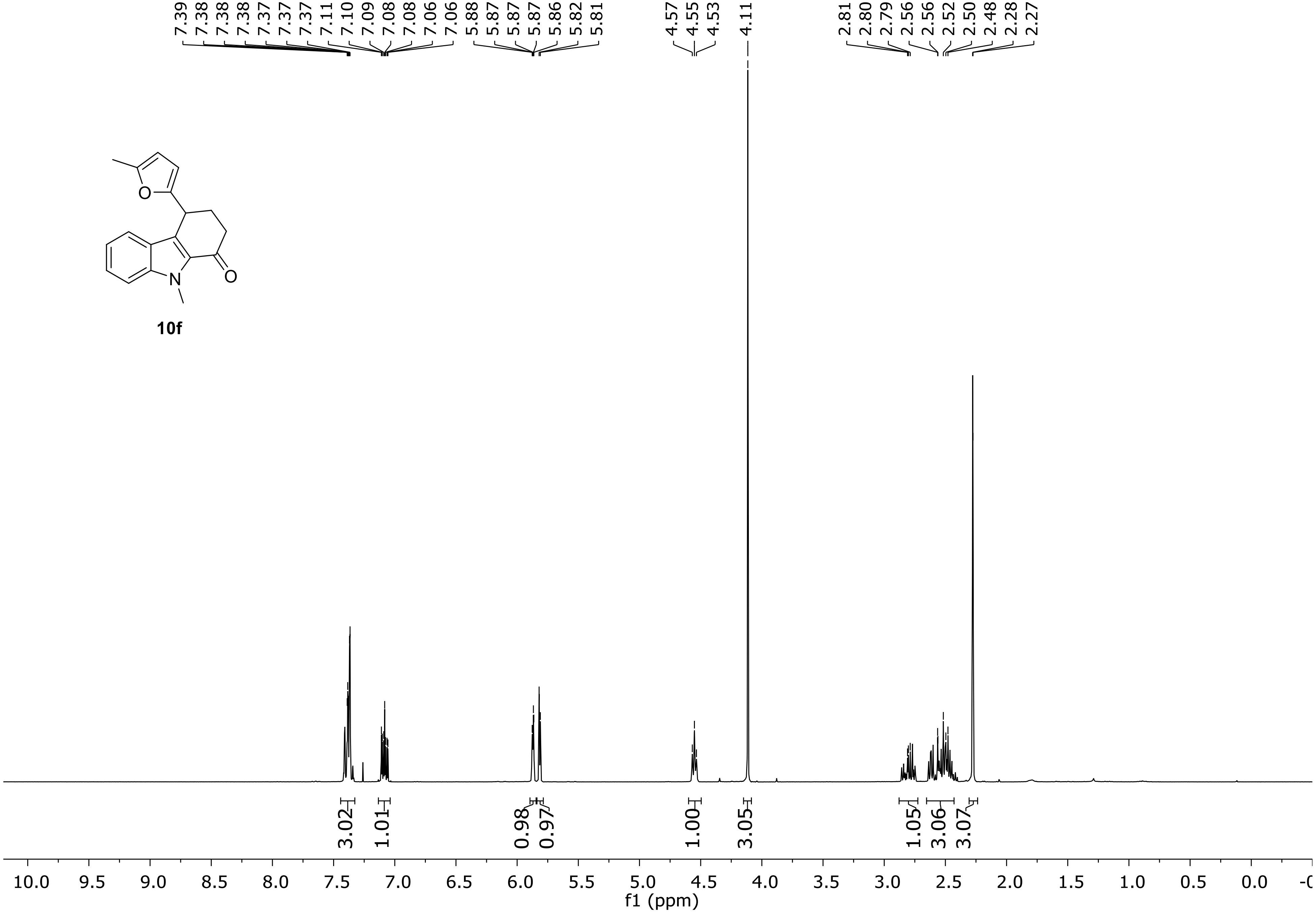

10f

^{13}C-{^{1}H}NMR (75.4 MHz, $CDCl_3$)

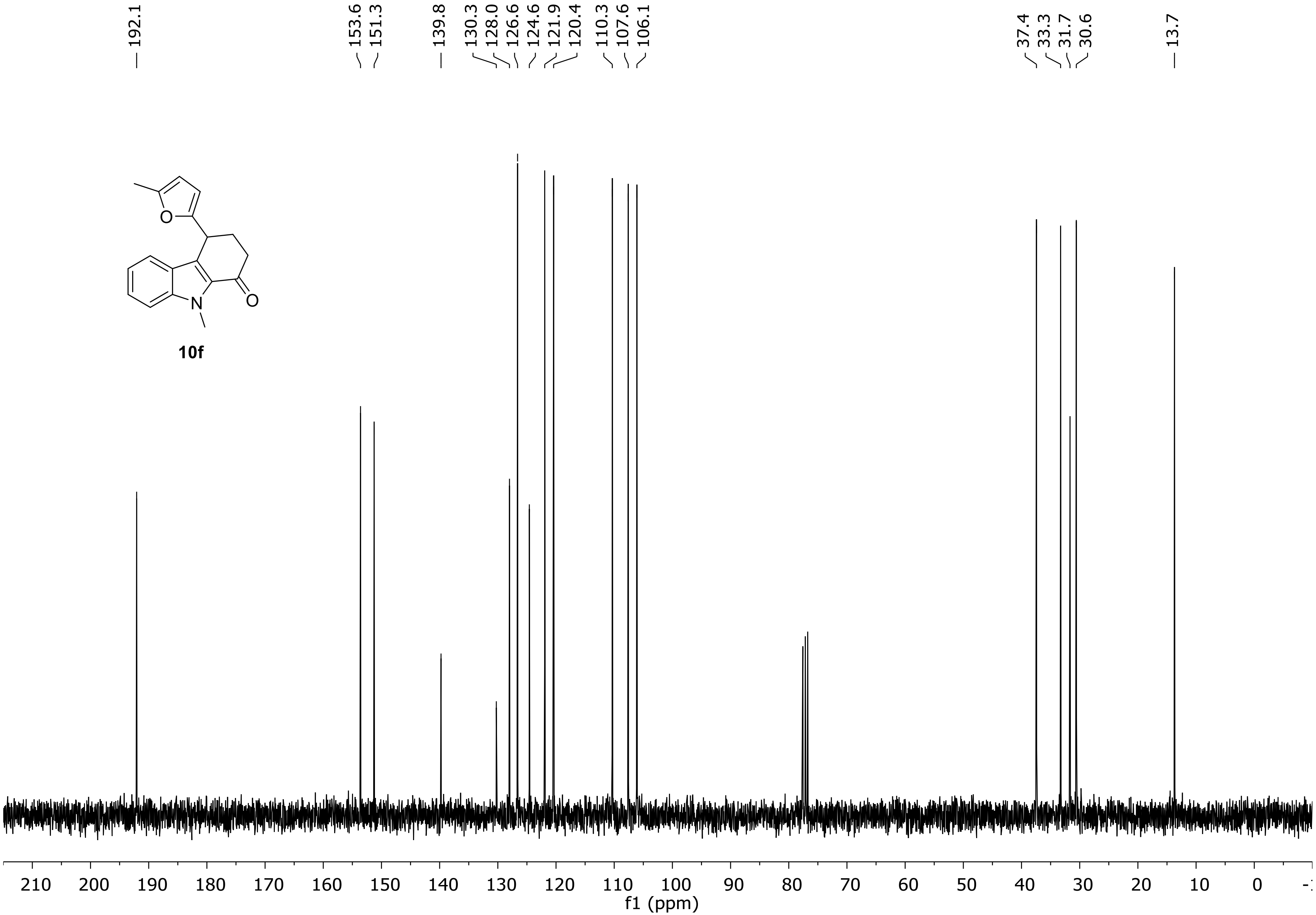

^{1}H-NMR (300 MHz, $CDCl_3$)

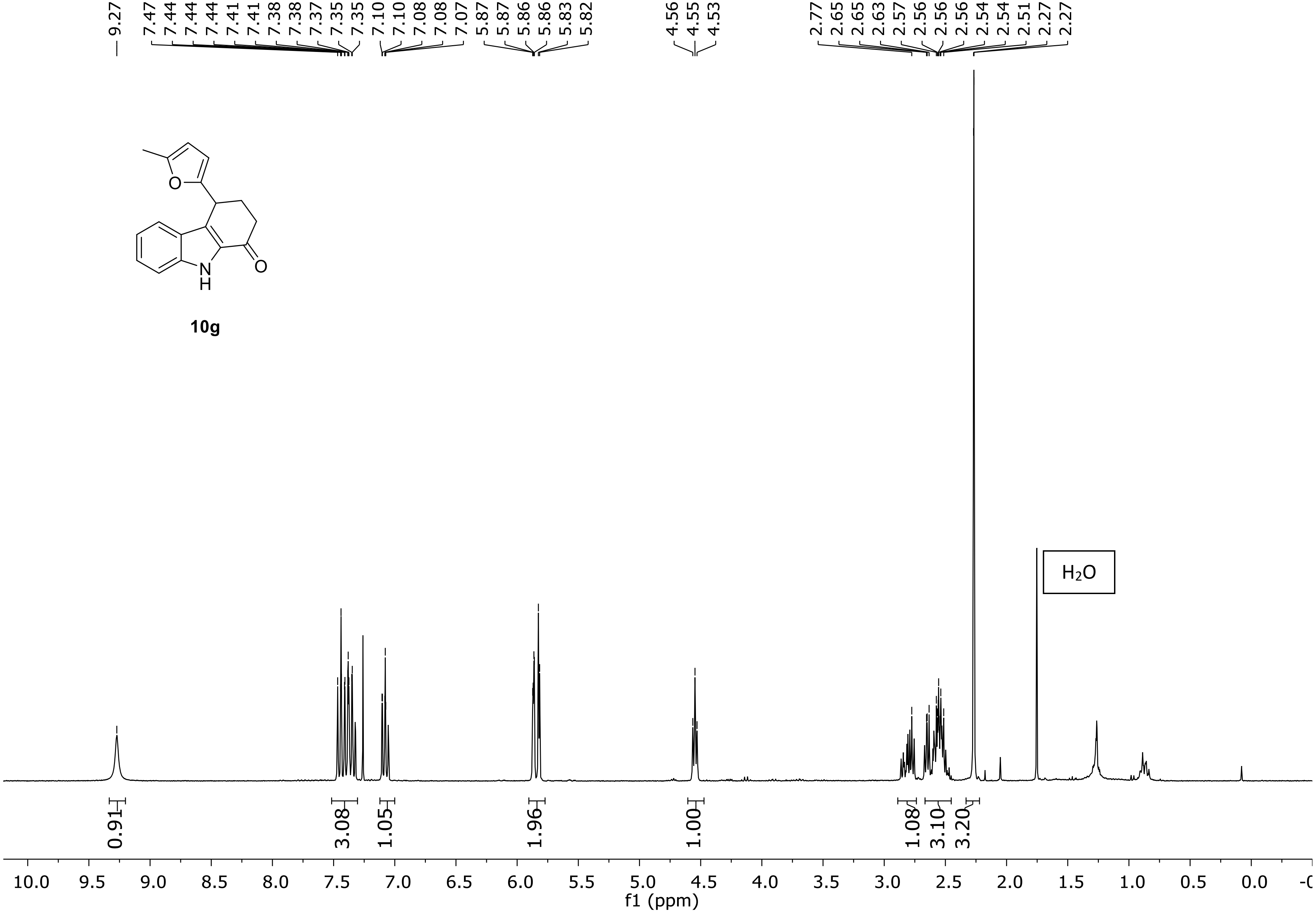

10g

$^{13}C-\{^{1}H\}$NMR (75.4 MHz, $CDCl_3$)

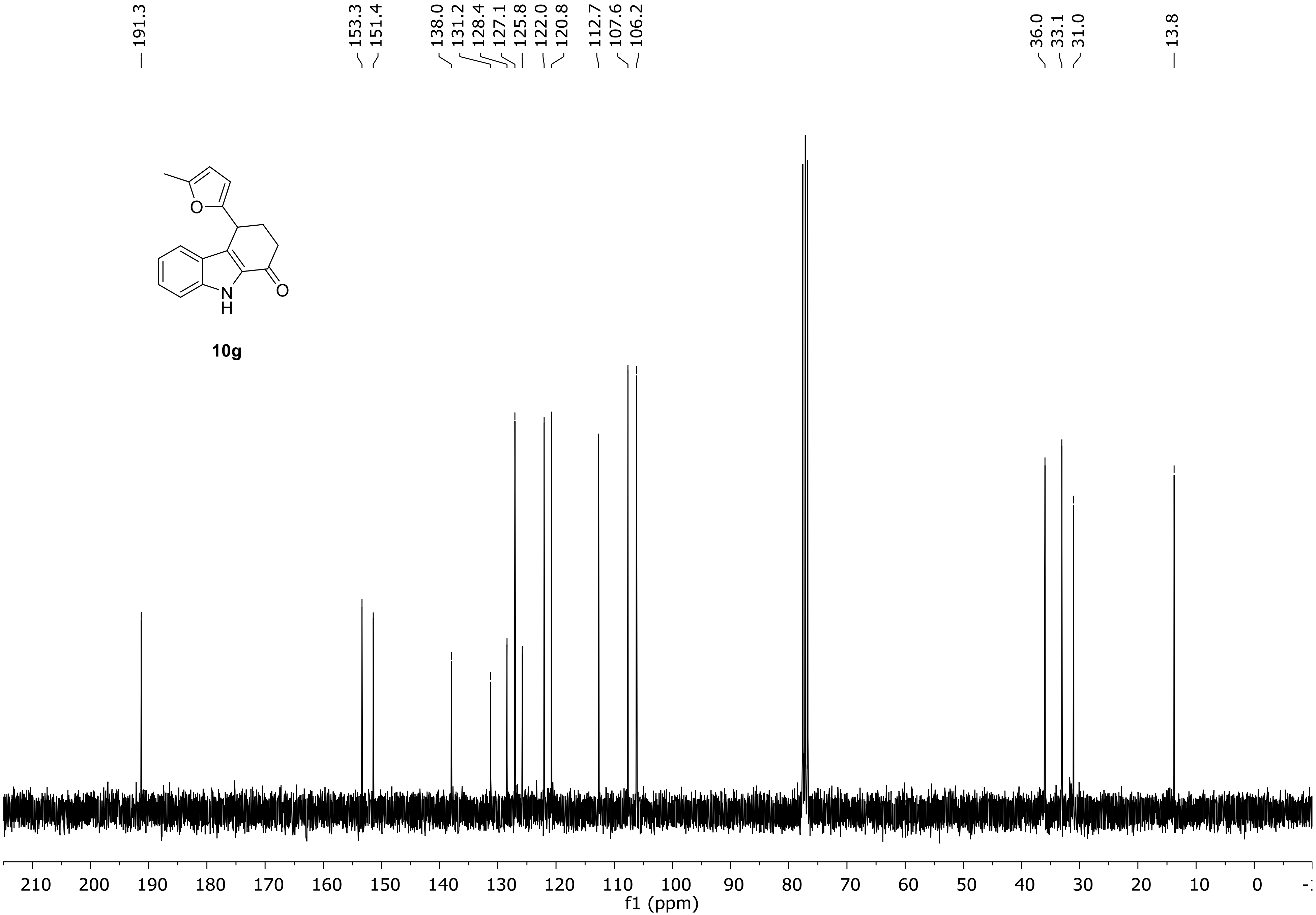

10g

^{1}H-NMR (300 MHz, $CDCl_3$)

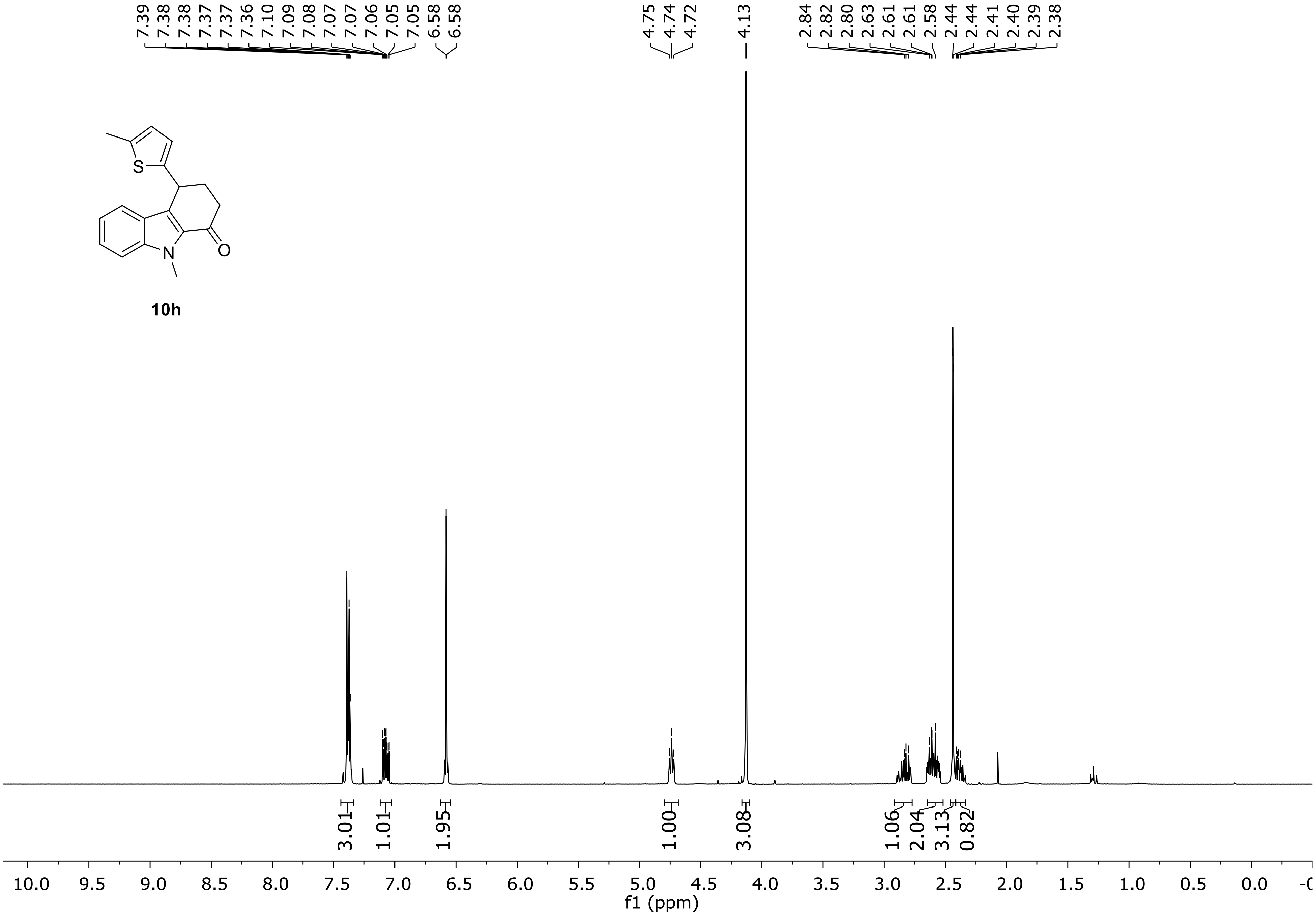

^{13}C-{^{1}H}NMR (75.4 MHz, $CDCl_3$)

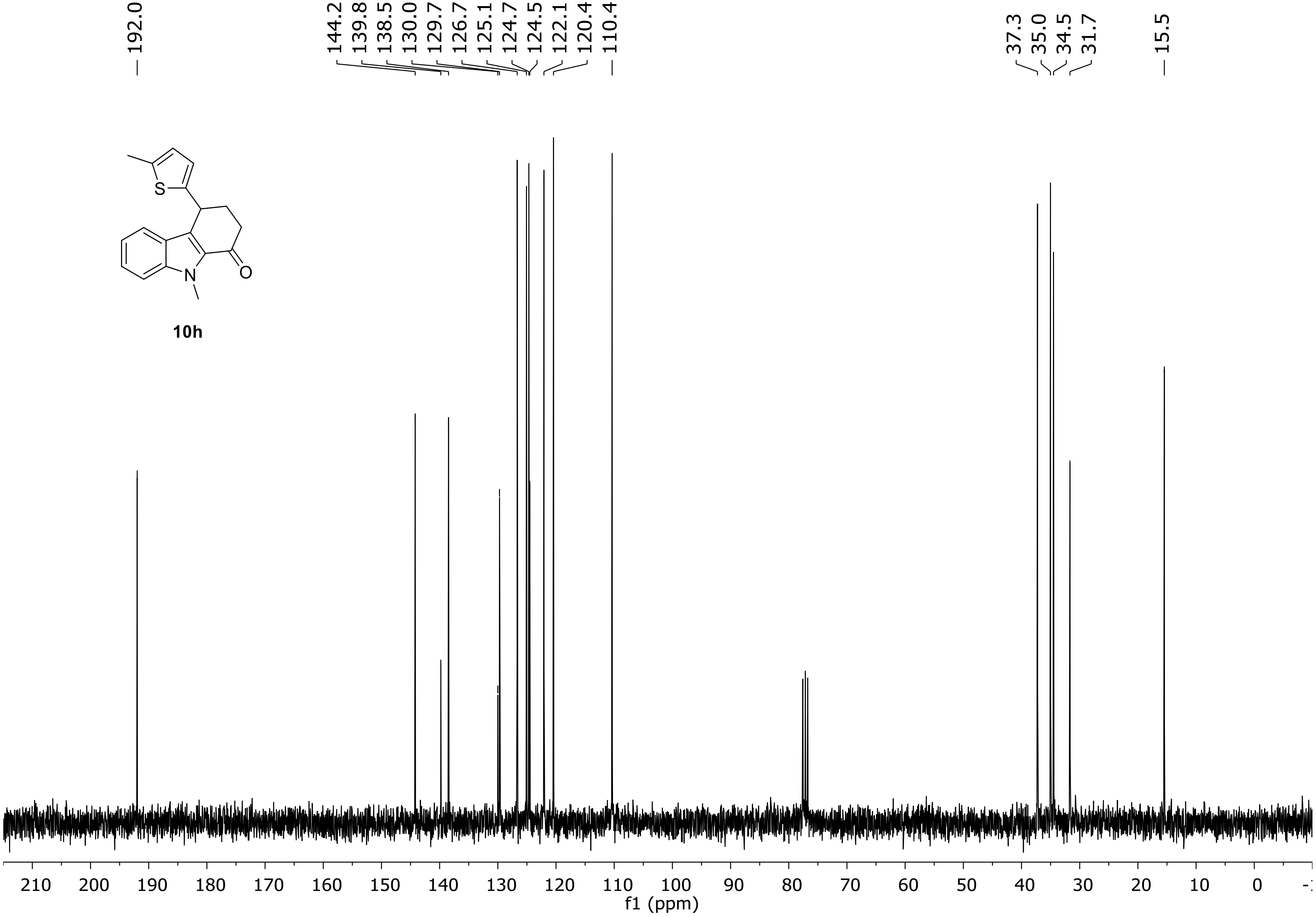

^{1}H-NMR (300 MHz, $CDCl_3$)

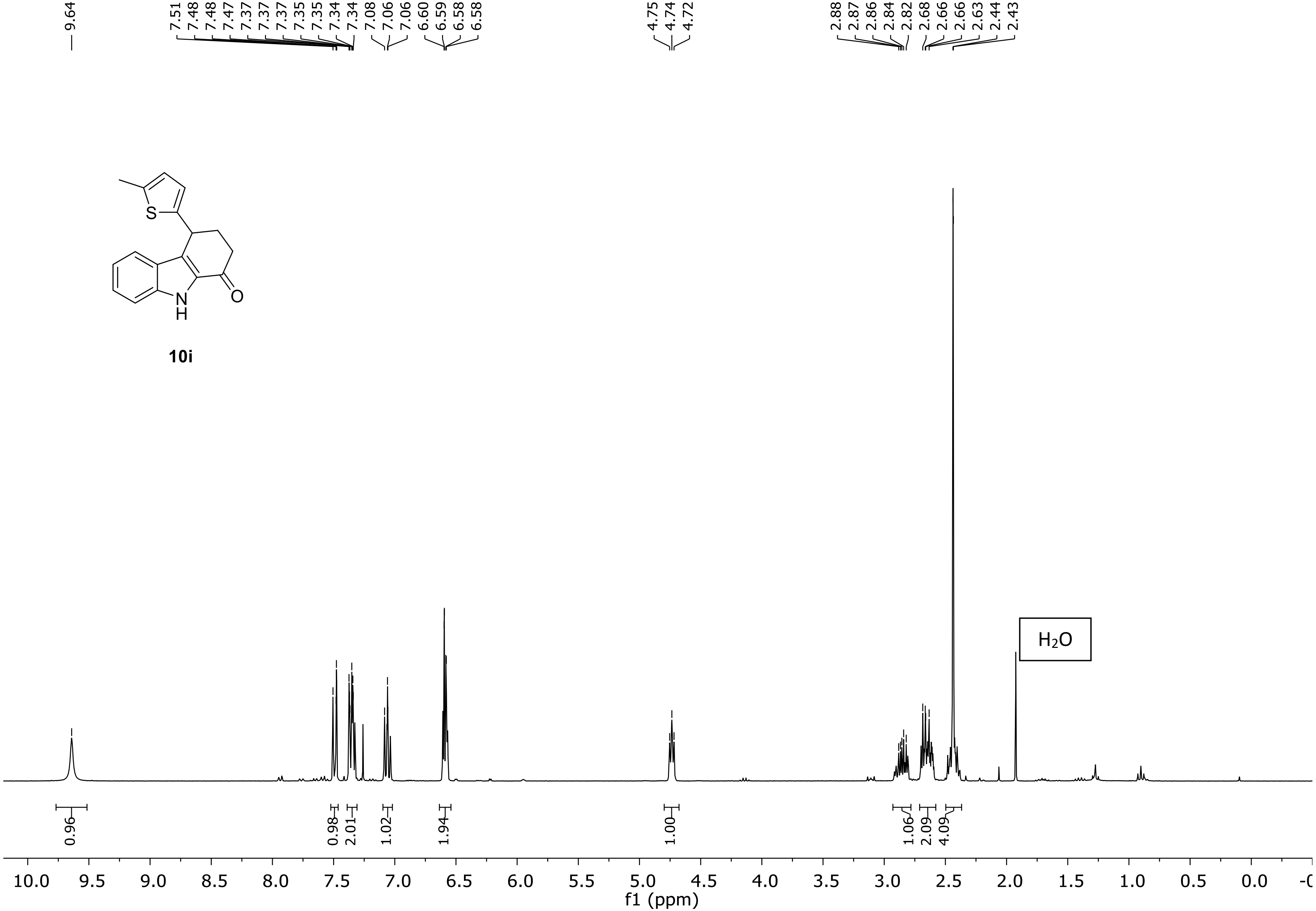

^{13}C-{^{1}H}NMR (75.4 MHz, $CDCl_3$)

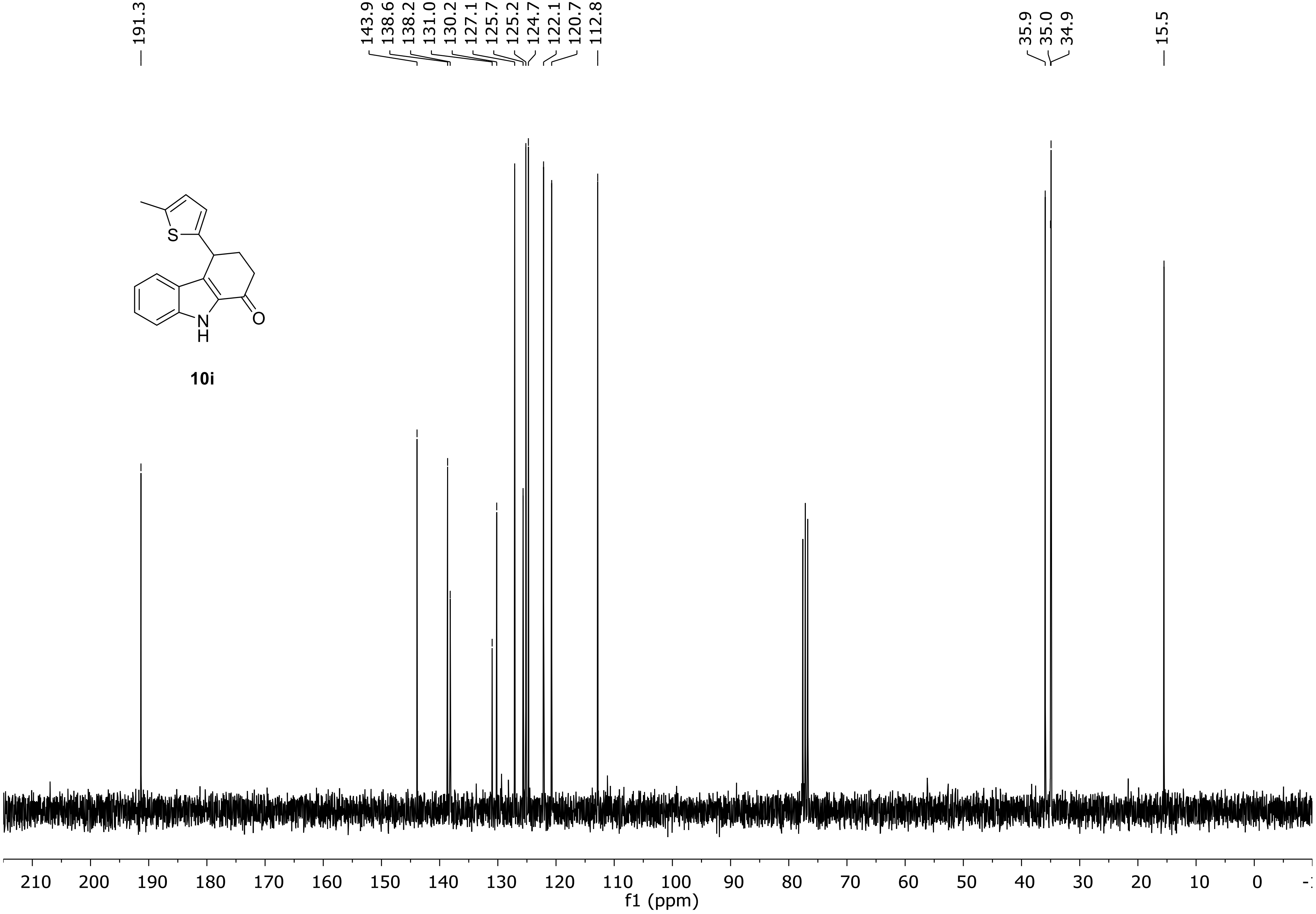

^{1}H-NMR (500 MHz, $CDCl_3$)

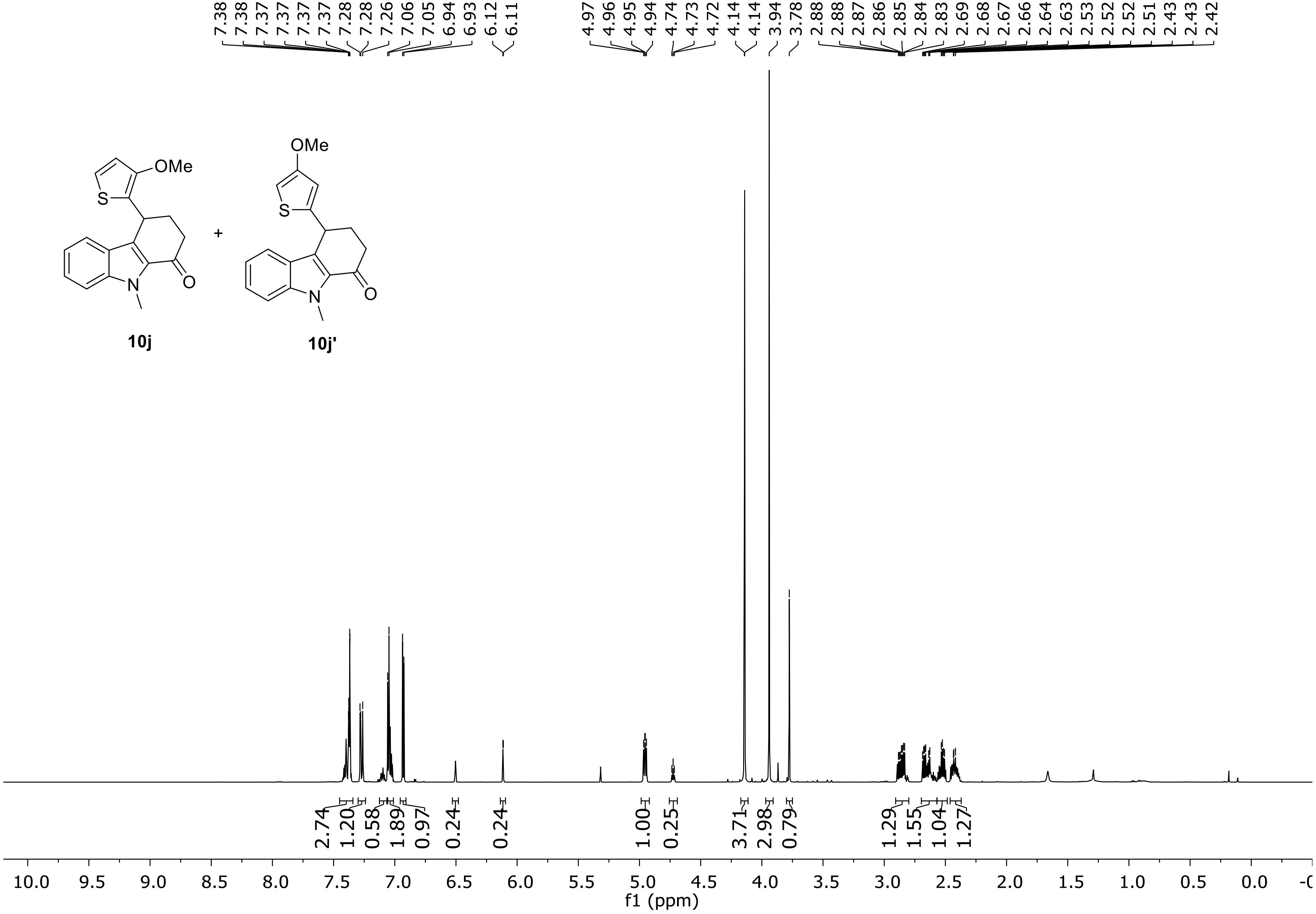

^{13}C-{^{1}H}NMR (126 MHz, $CDCl_3$)

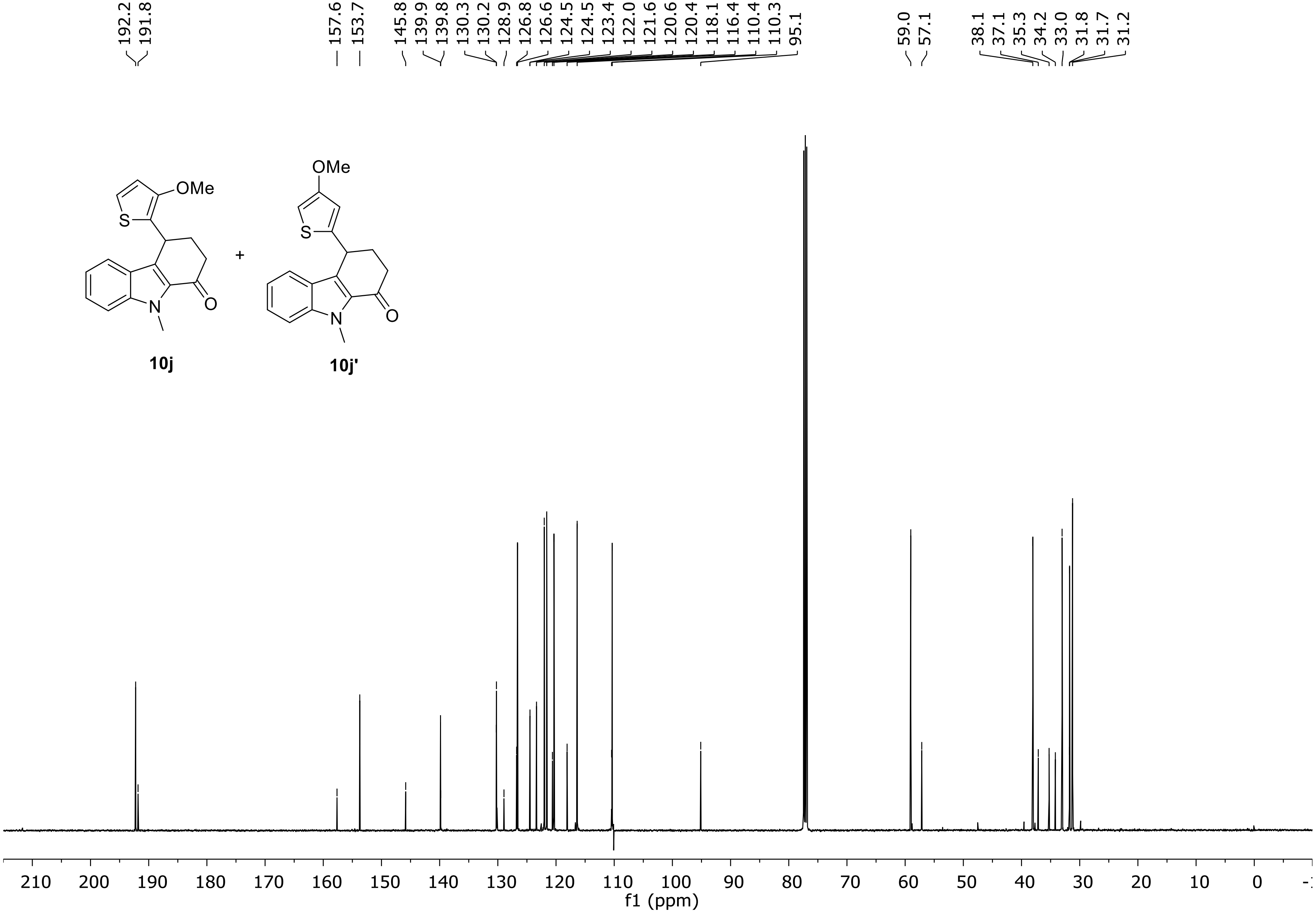

^{1}H-NMR (300 MHz, $CDCl_3$)

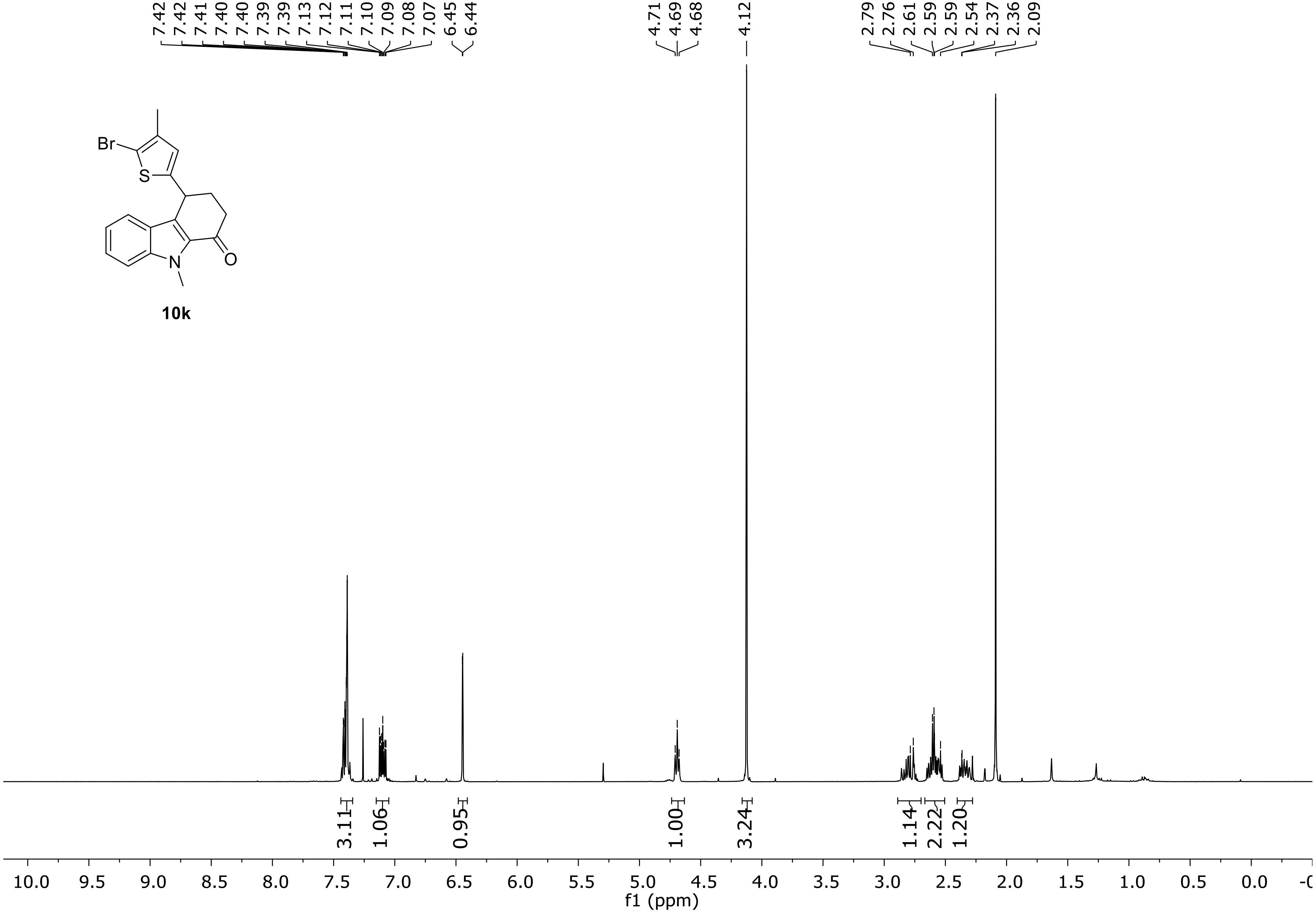

$^{13}C-\{^{1}H\}$NMR (74.5 MHz, $CDCl_3$)

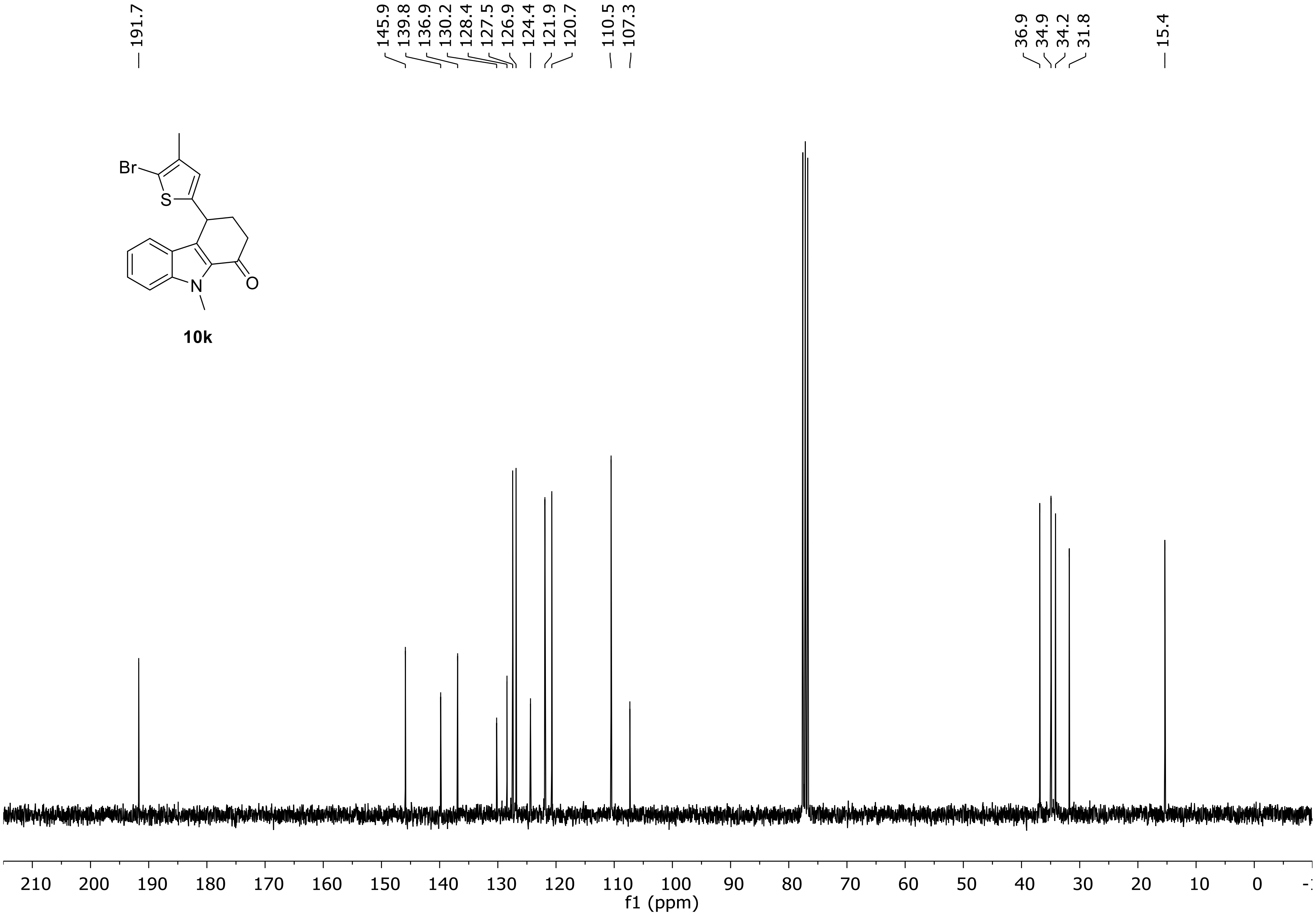

10k

^{1}H-NMR (300 MHz, $CDCl_3$)

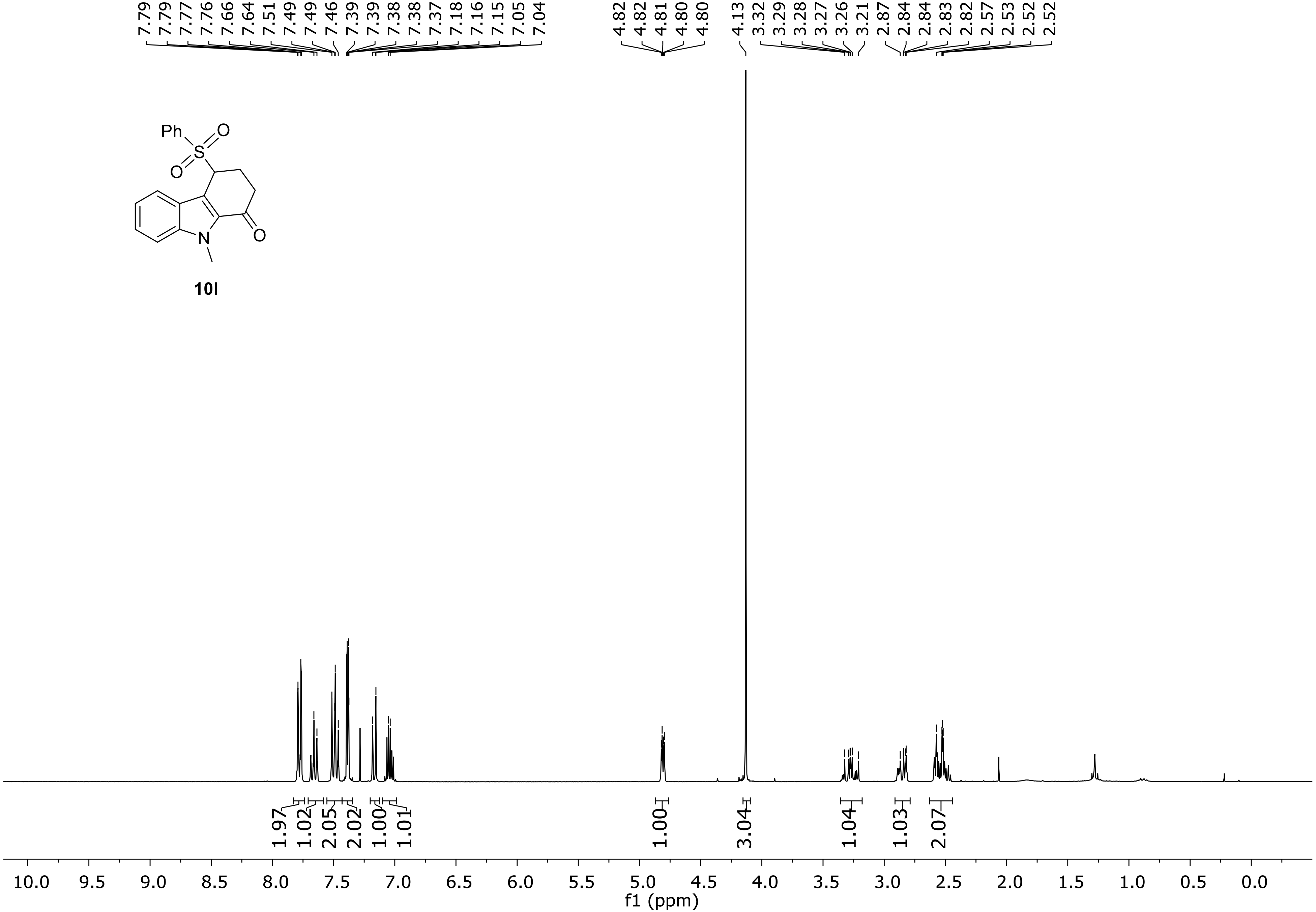

^{13}C-{^1H}NMR (74.5 MHz, $CDCl_3$)

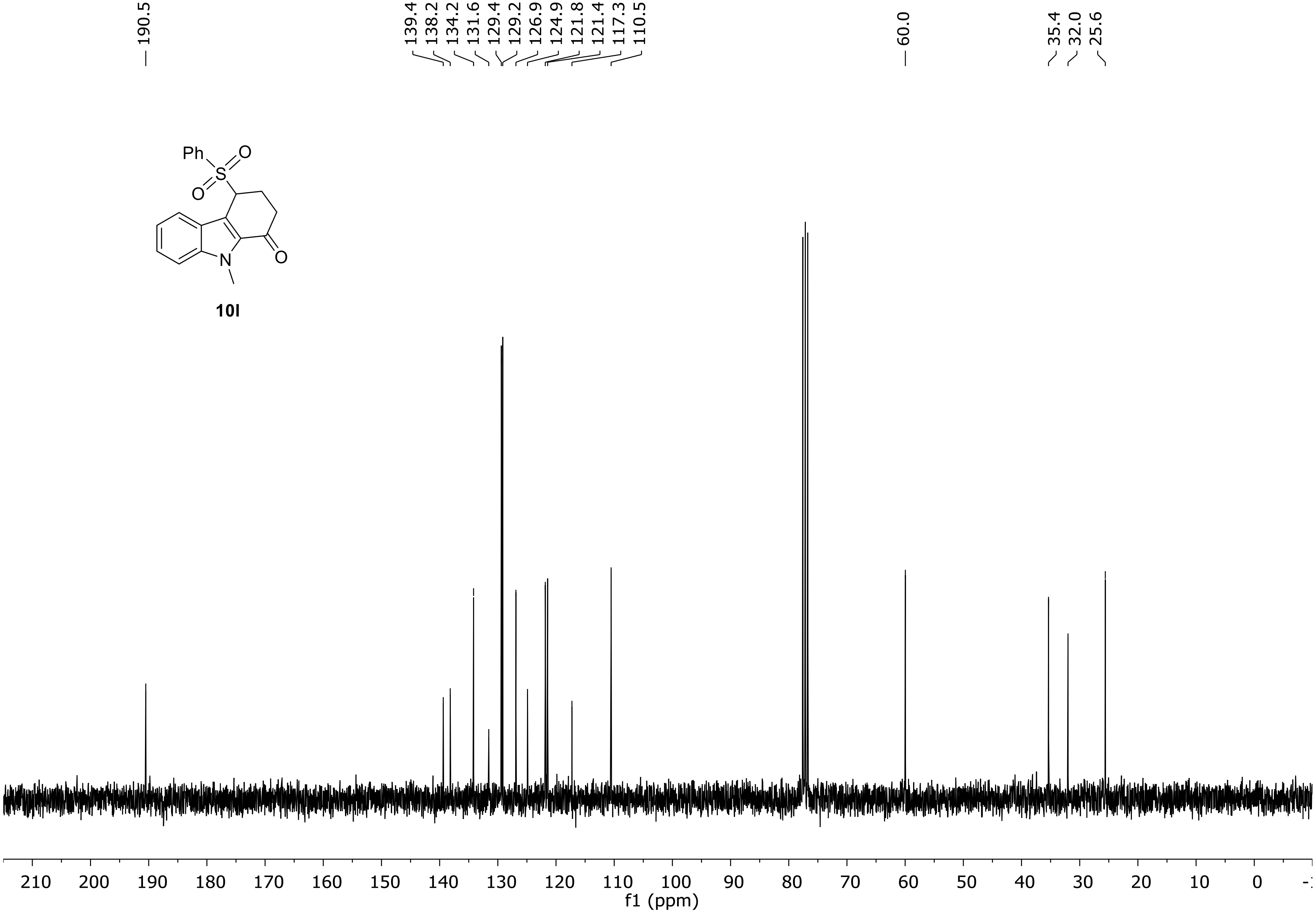

10l

^{1}H-NMR (300 MHz, $CDCl_3$)

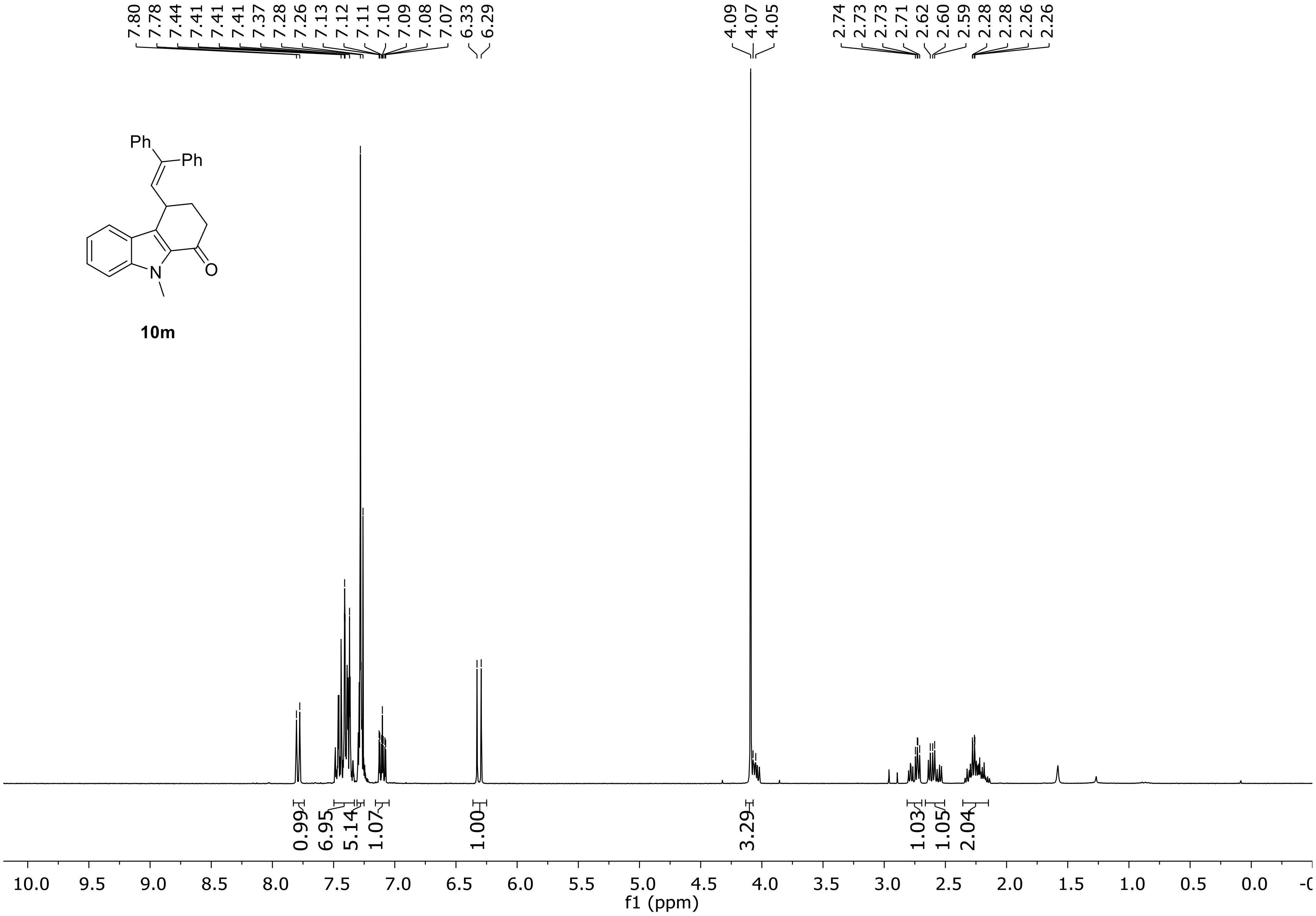

$^{13}C-\{^{1}H\}NMR$ (74.5 MHz, $CDCl_3$)

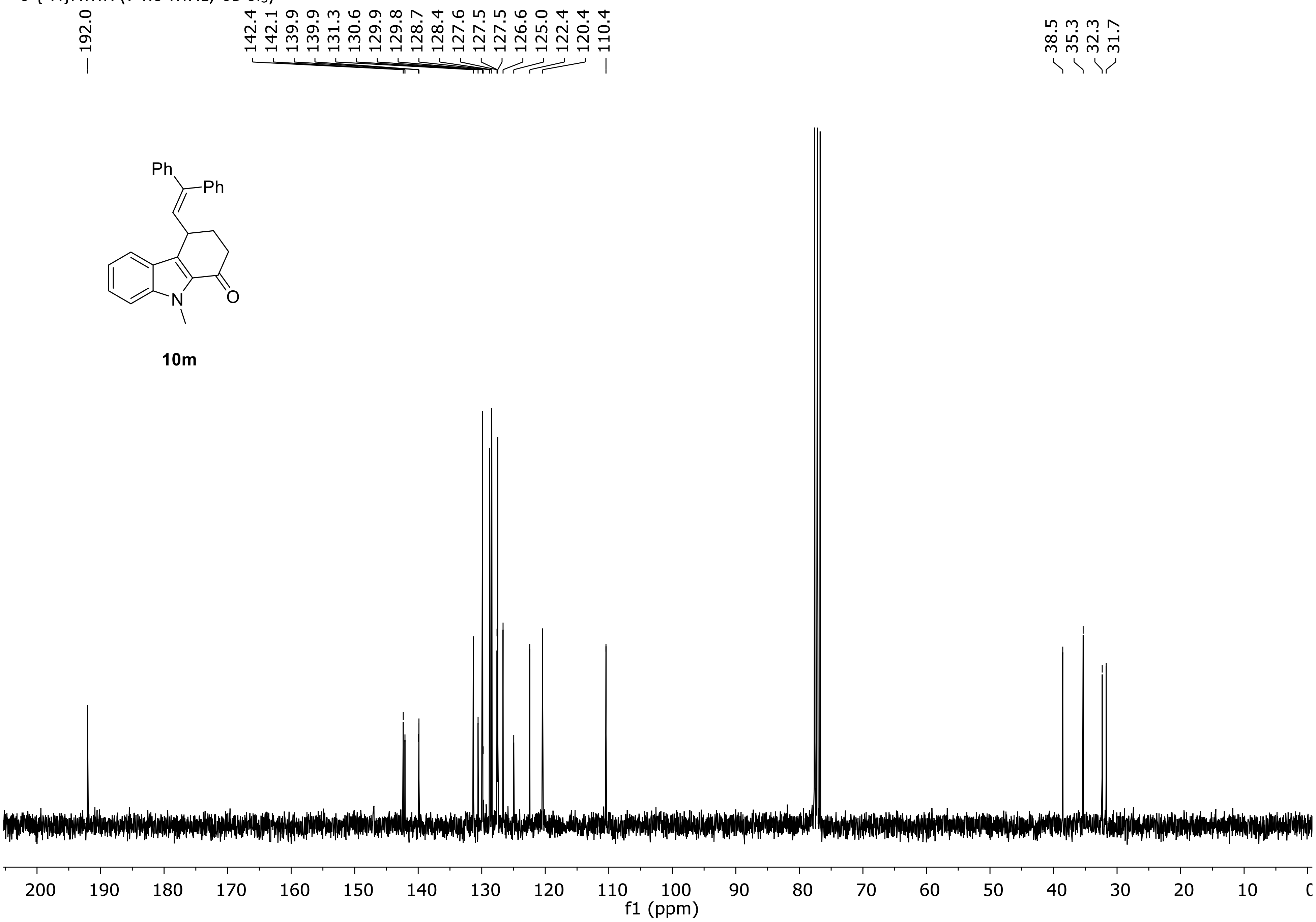

^{1}H-NMR (300 MHz, $CDCl_3$)

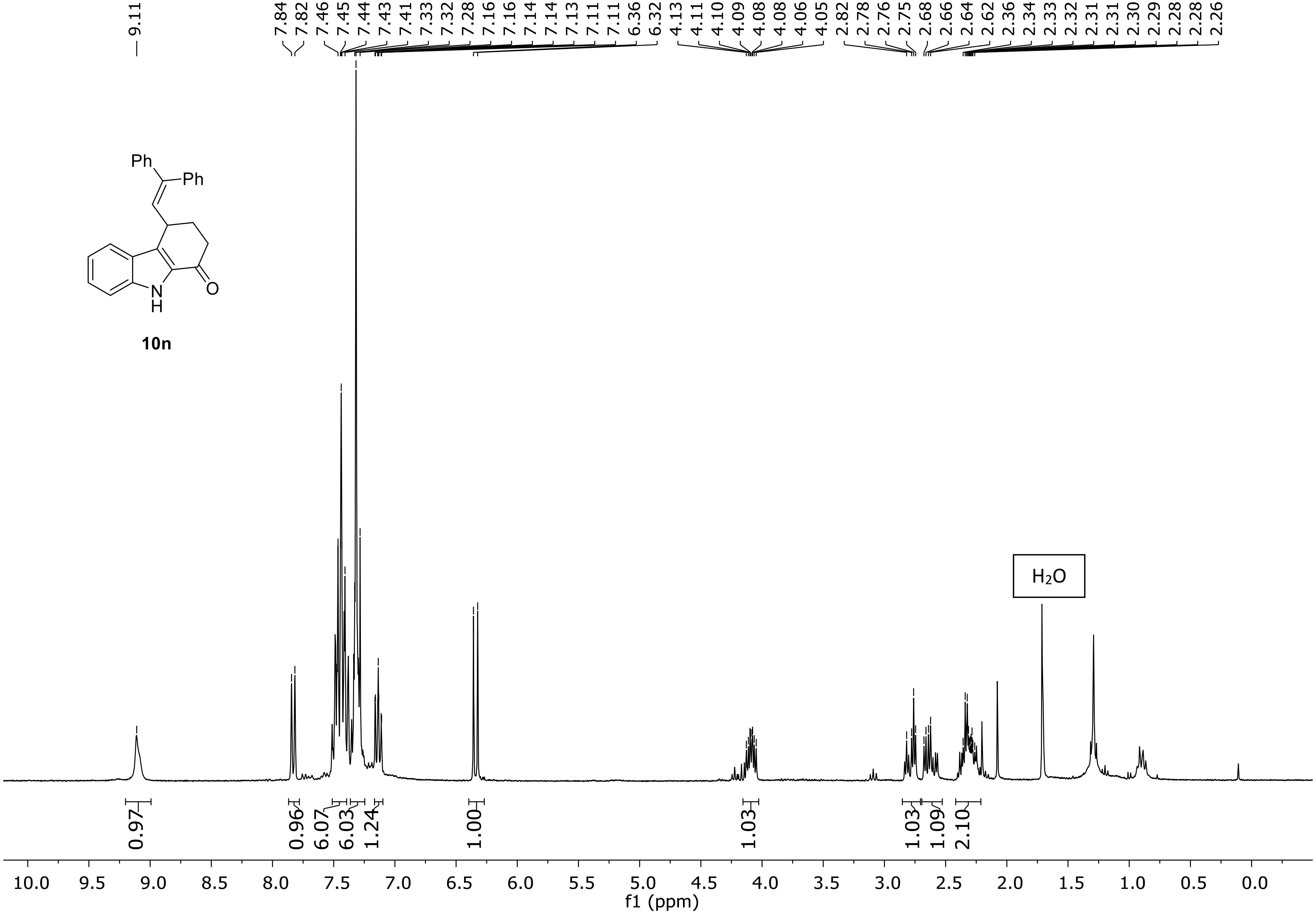

$^{13}C-\{^{1}H\}$NMR (74.5 MHz, $CDCl_3$)

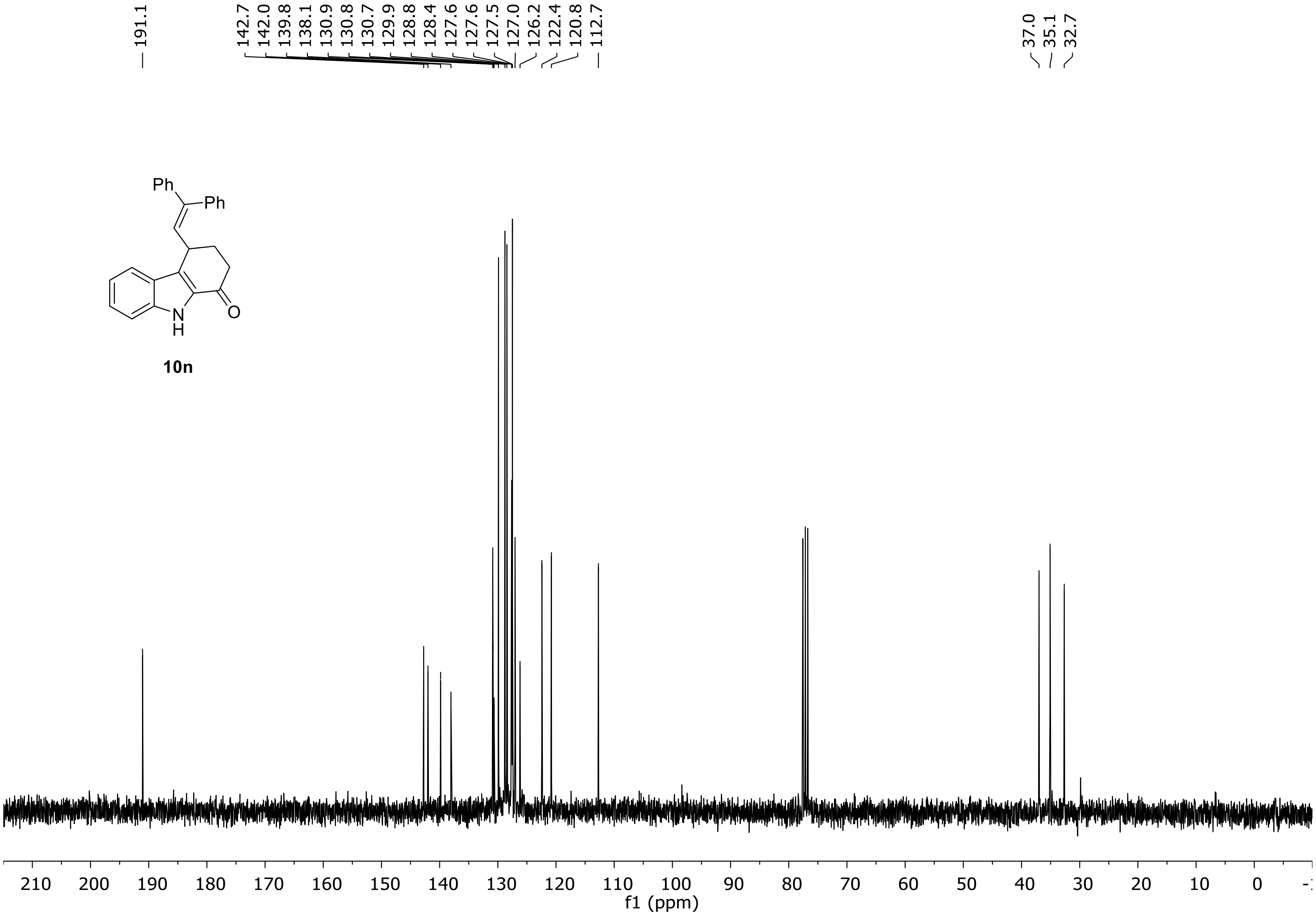

10n

^{1}H-NMR (300 MHz, $CDCl_3$)

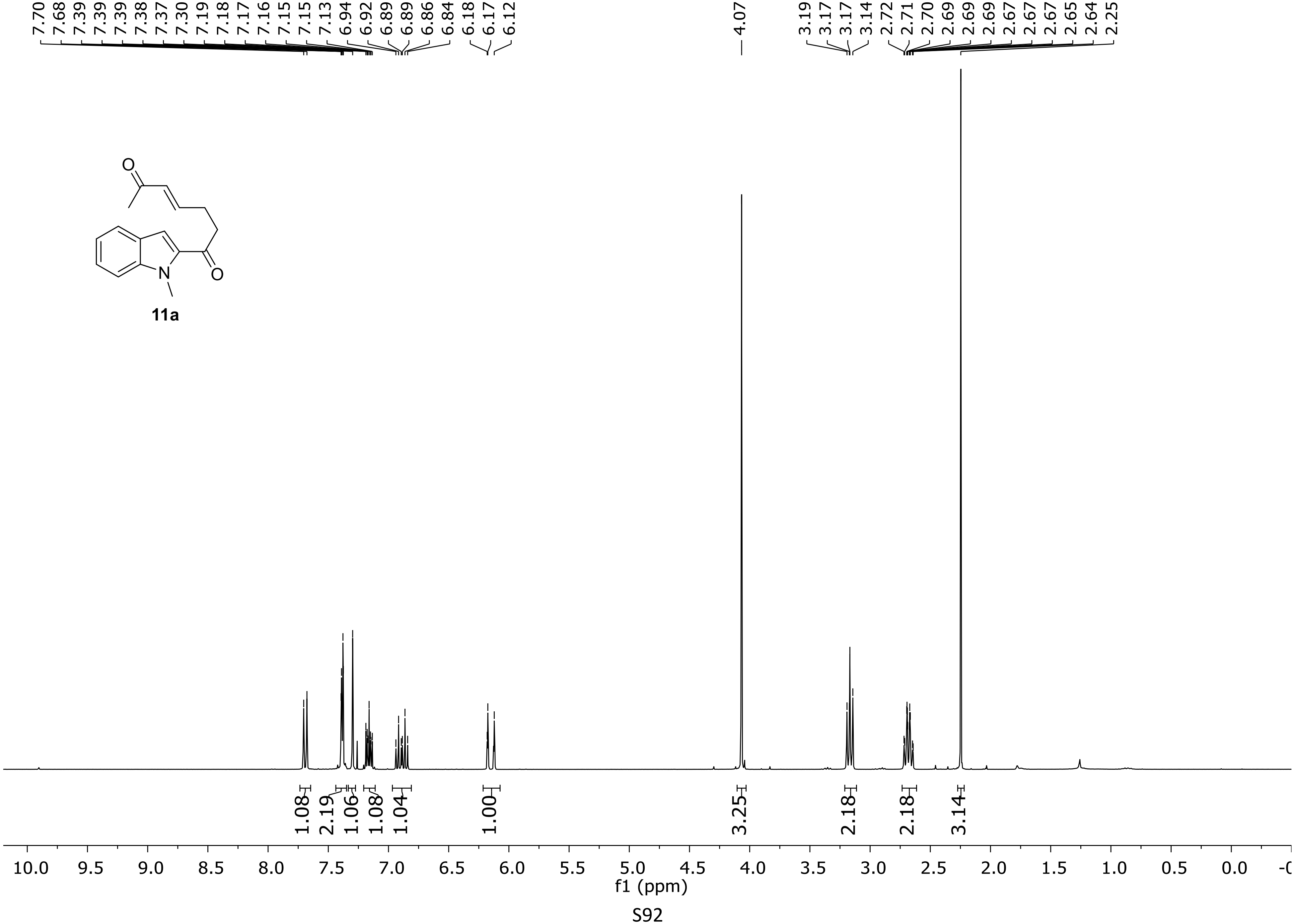

^{13}C-{^{1}H}NMR (74.5 MHz, $CDCl_3$)

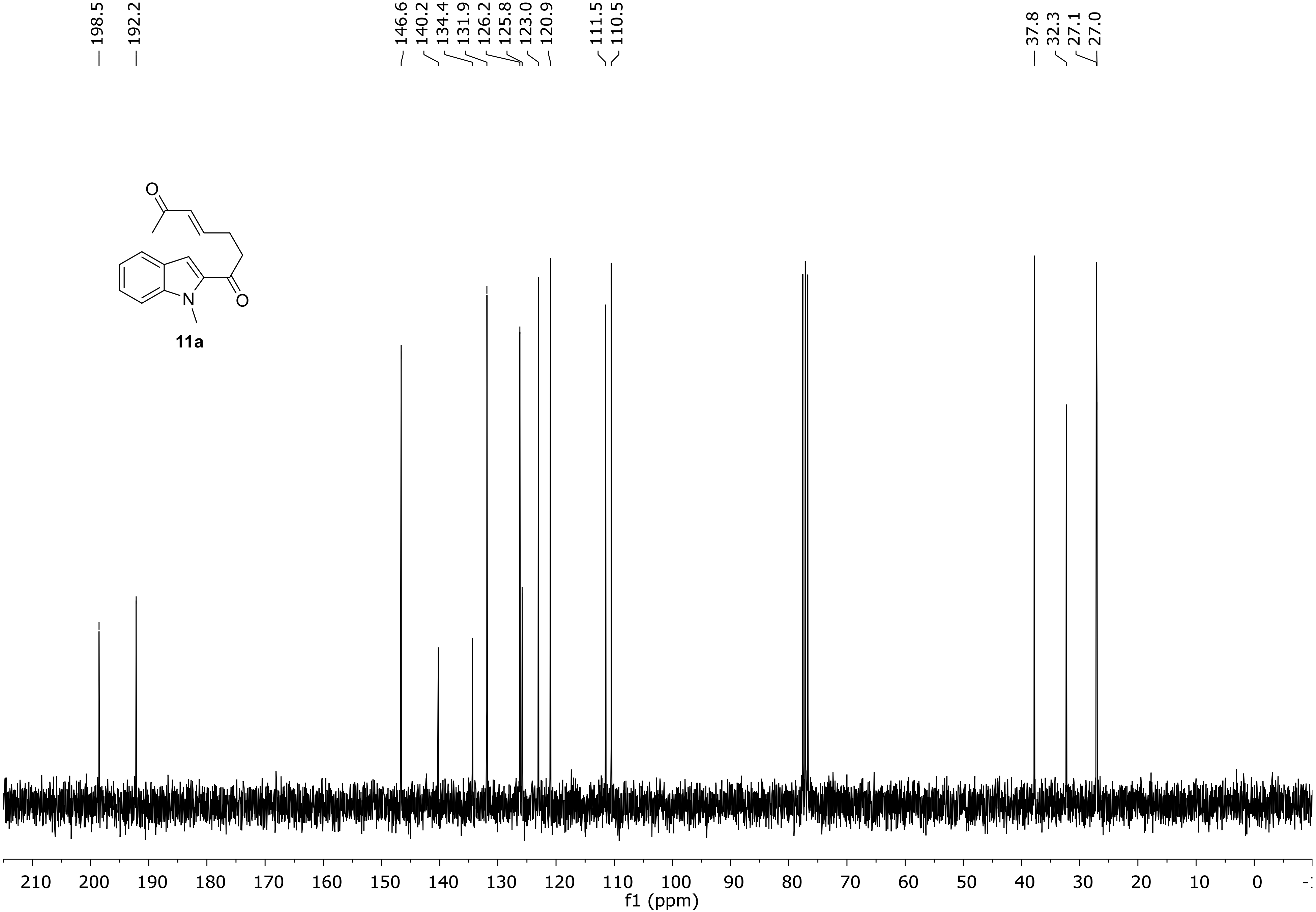

11a

^{1}H-NMR (300 MHz, $CDCl_3$)

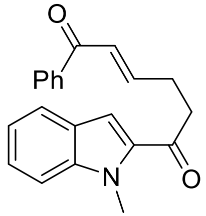

11b

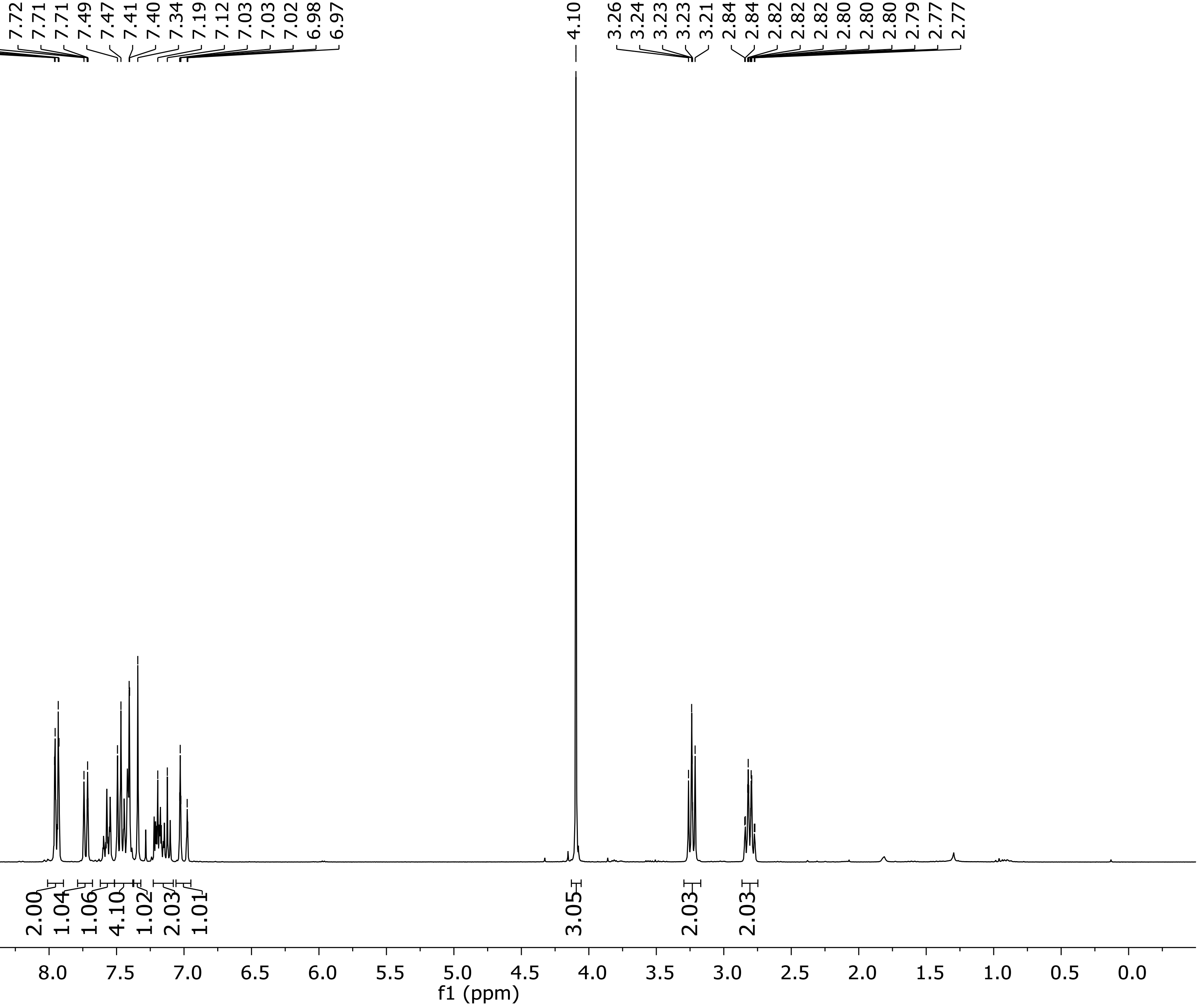

^{13}C-{^{1}H}NMR (74.5 MHz, $CDCl_3$)

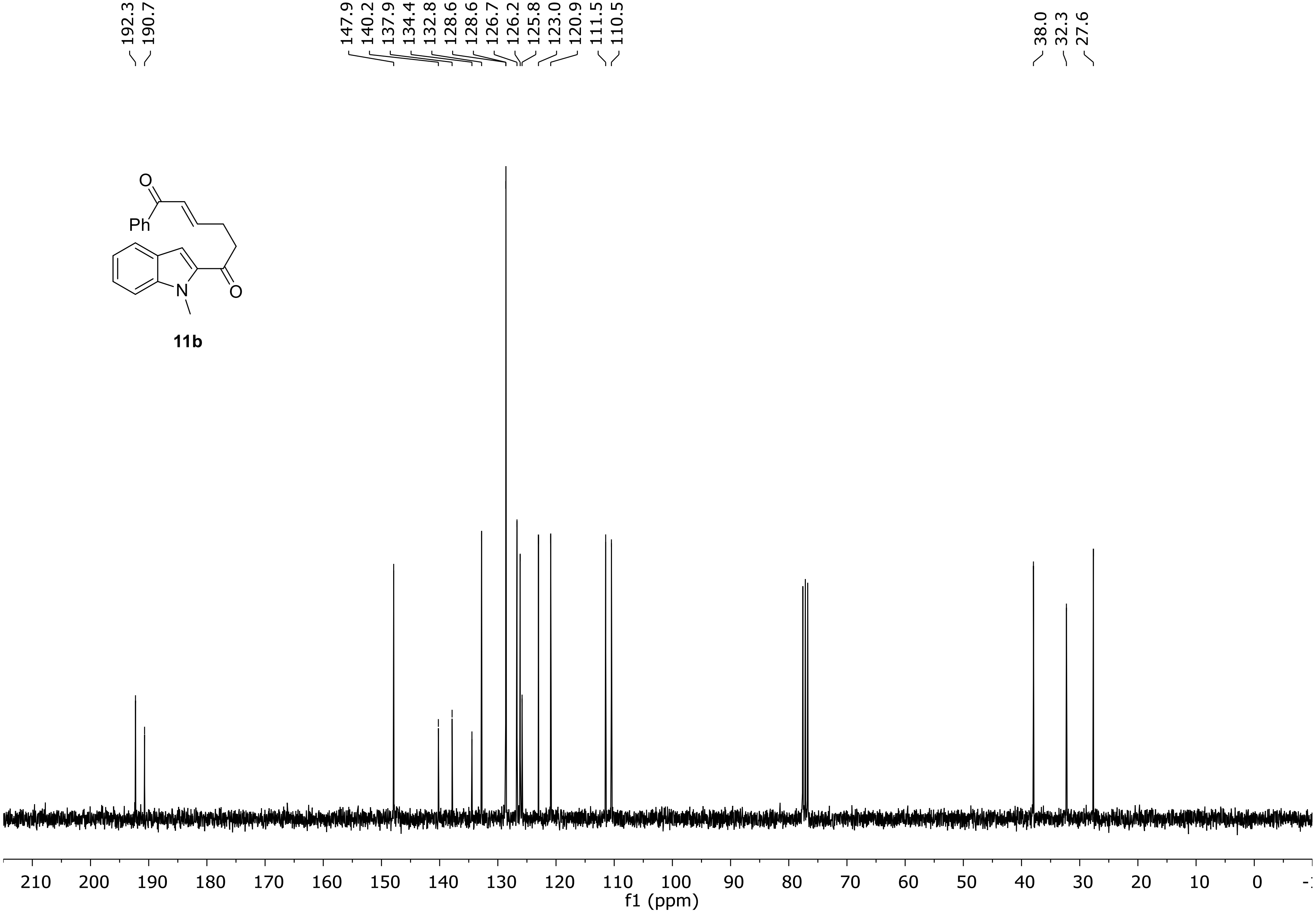

^{1}H-NMR (300 MHz, $CDCl_3$)

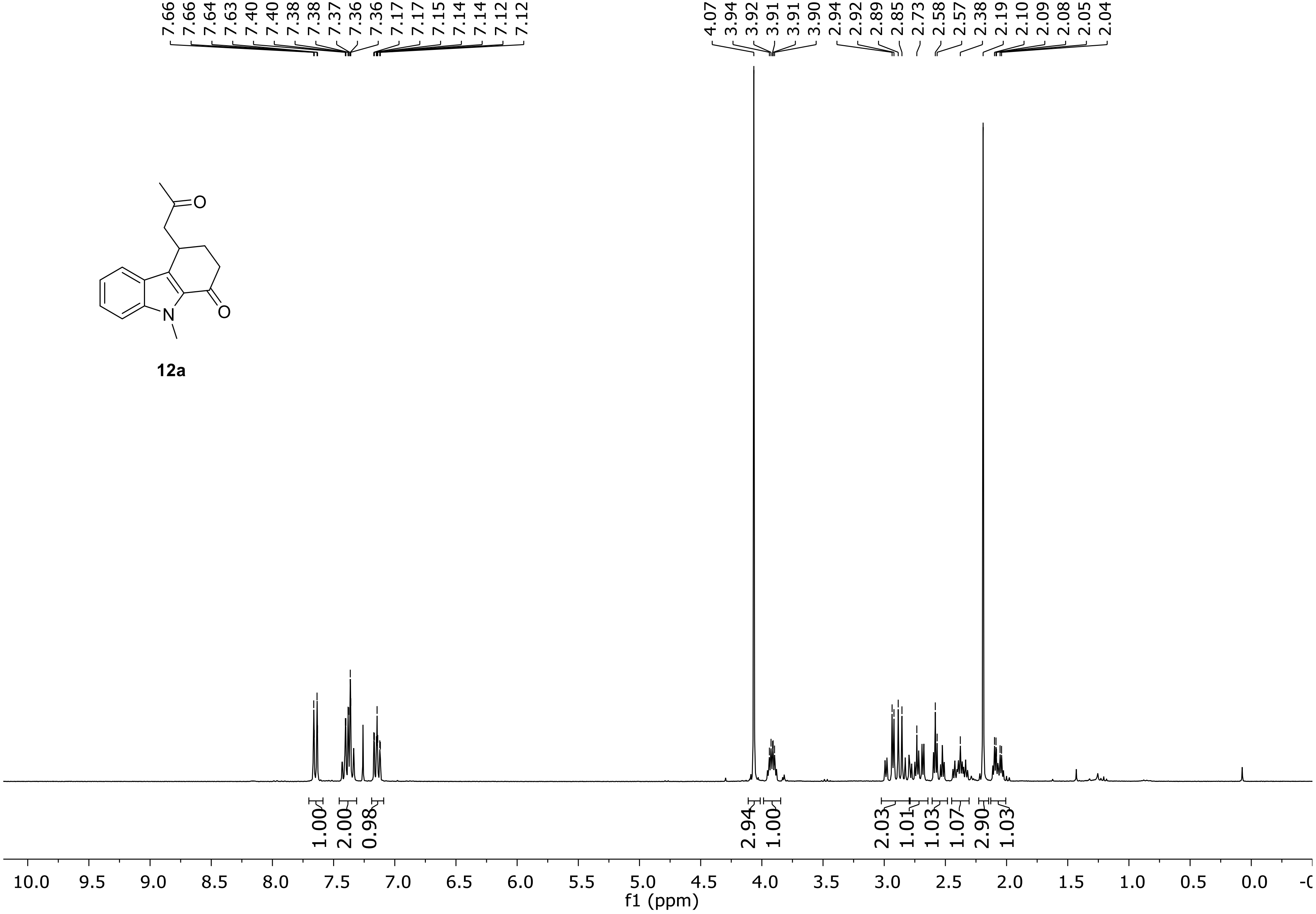

12a

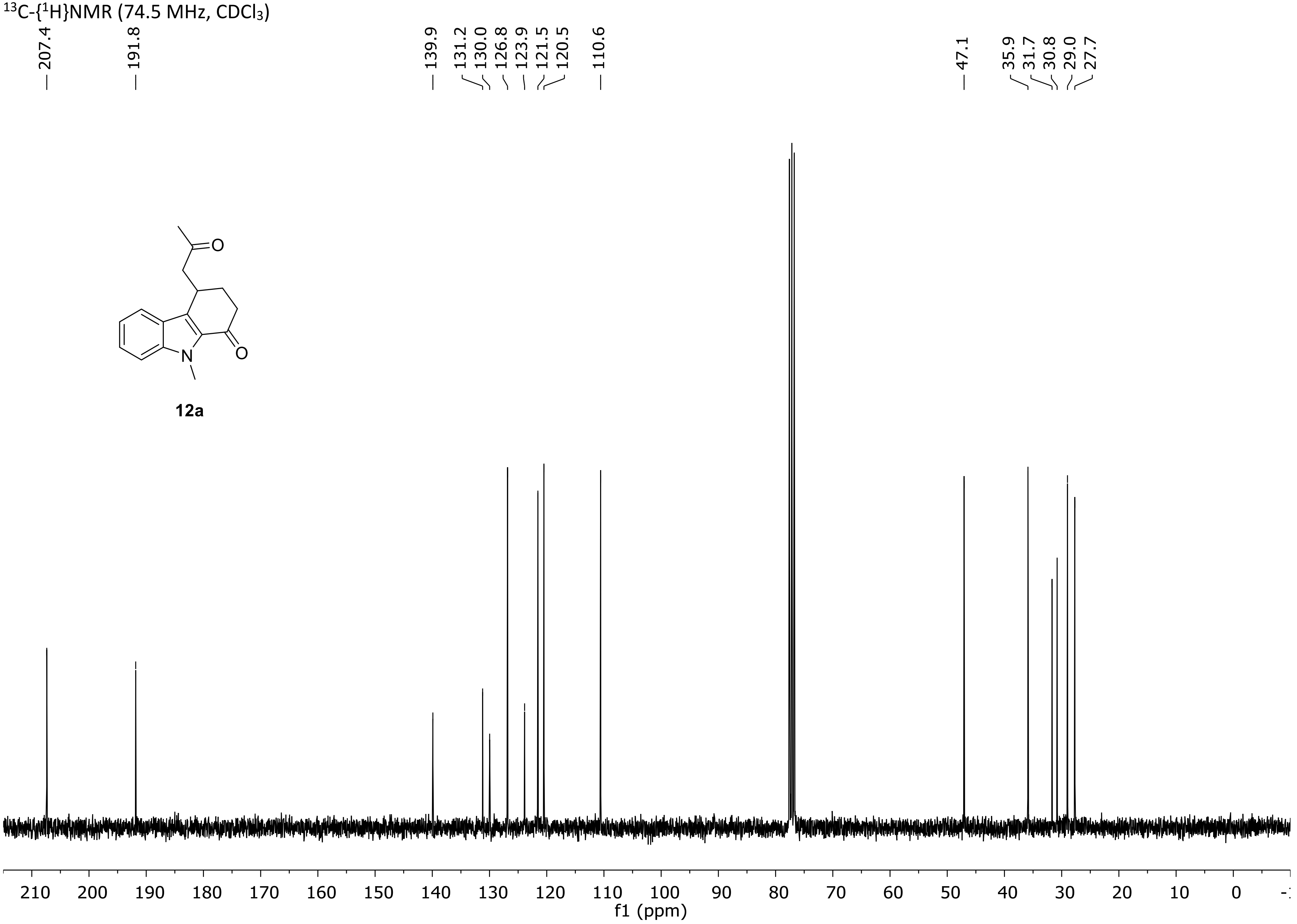
$^{13}C-\{^1H\}NMR$ (74.5 MHz, $CDCl_3$)
207.4
191.8
139.9
131.2
130.0
126.8
123.9
121.5
120.5
110.6
47.1
35.9
31.7
30.8
29.0
27.7
210 200 190 180 170 160 150 140 130 120 110 100 90 80 70 60 50 40 30 20 10 0
f1 (ppm)
O
N
O

12a

^{1}H-NMR (300 MHz, $CDCl_3$)

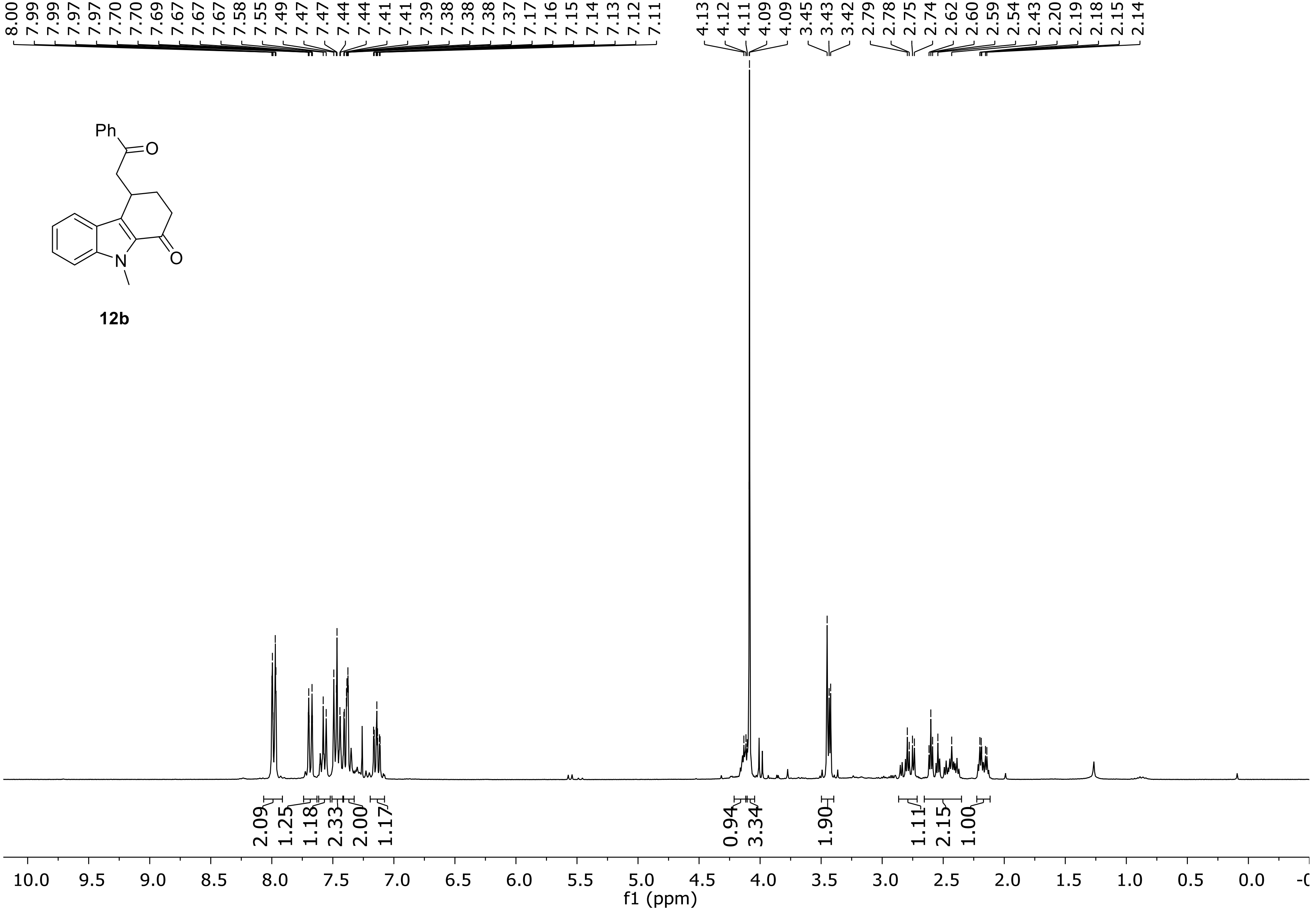

^{13}C-{^{1}H}NMR (74.5 MHz, $CDCl_3$)

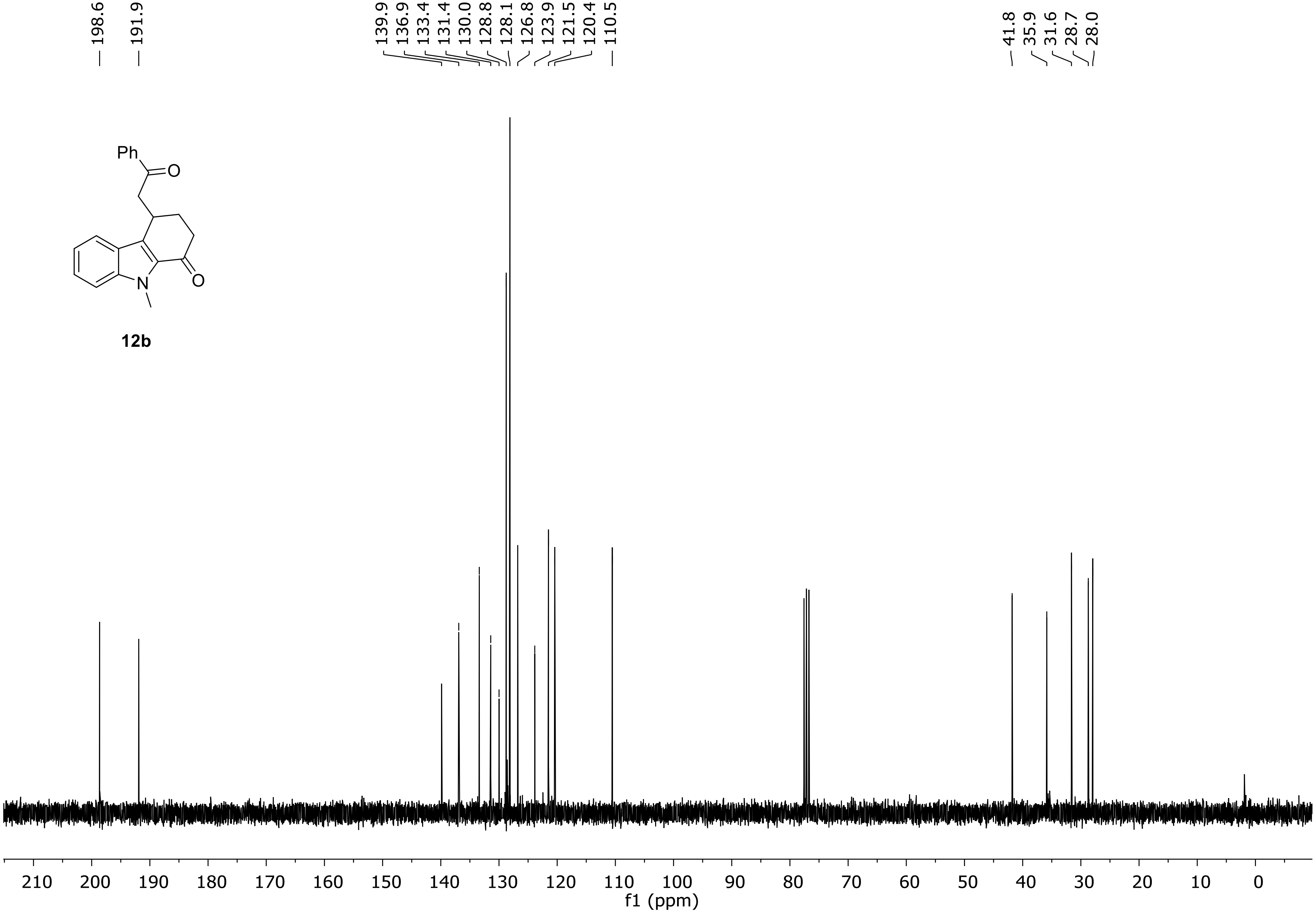

^{1}H-NMR (300 MHz, $CDCl_3$)

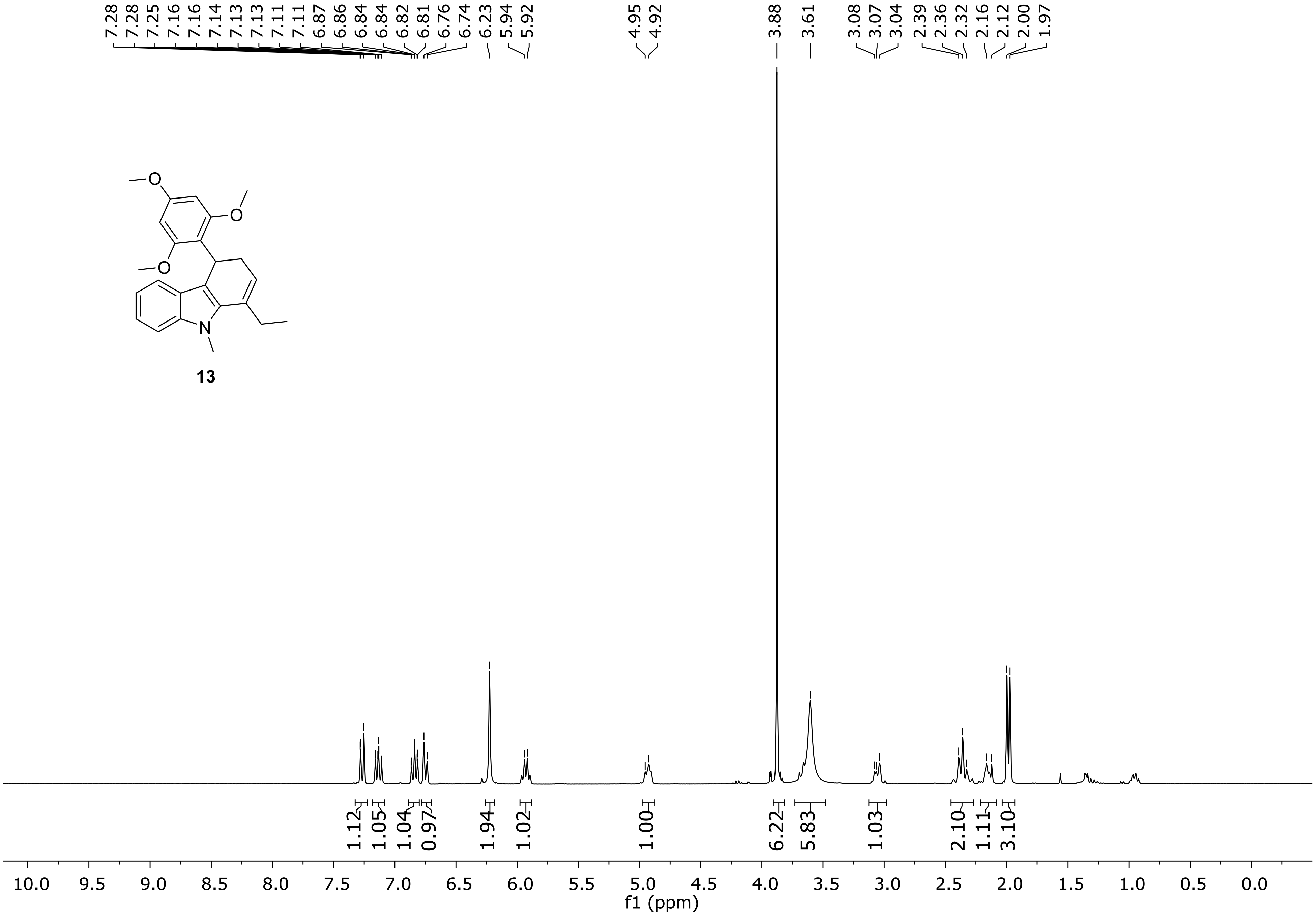

^{13}C-{^{1}H}NMR (74.5 MHz, $CDCl_3$)

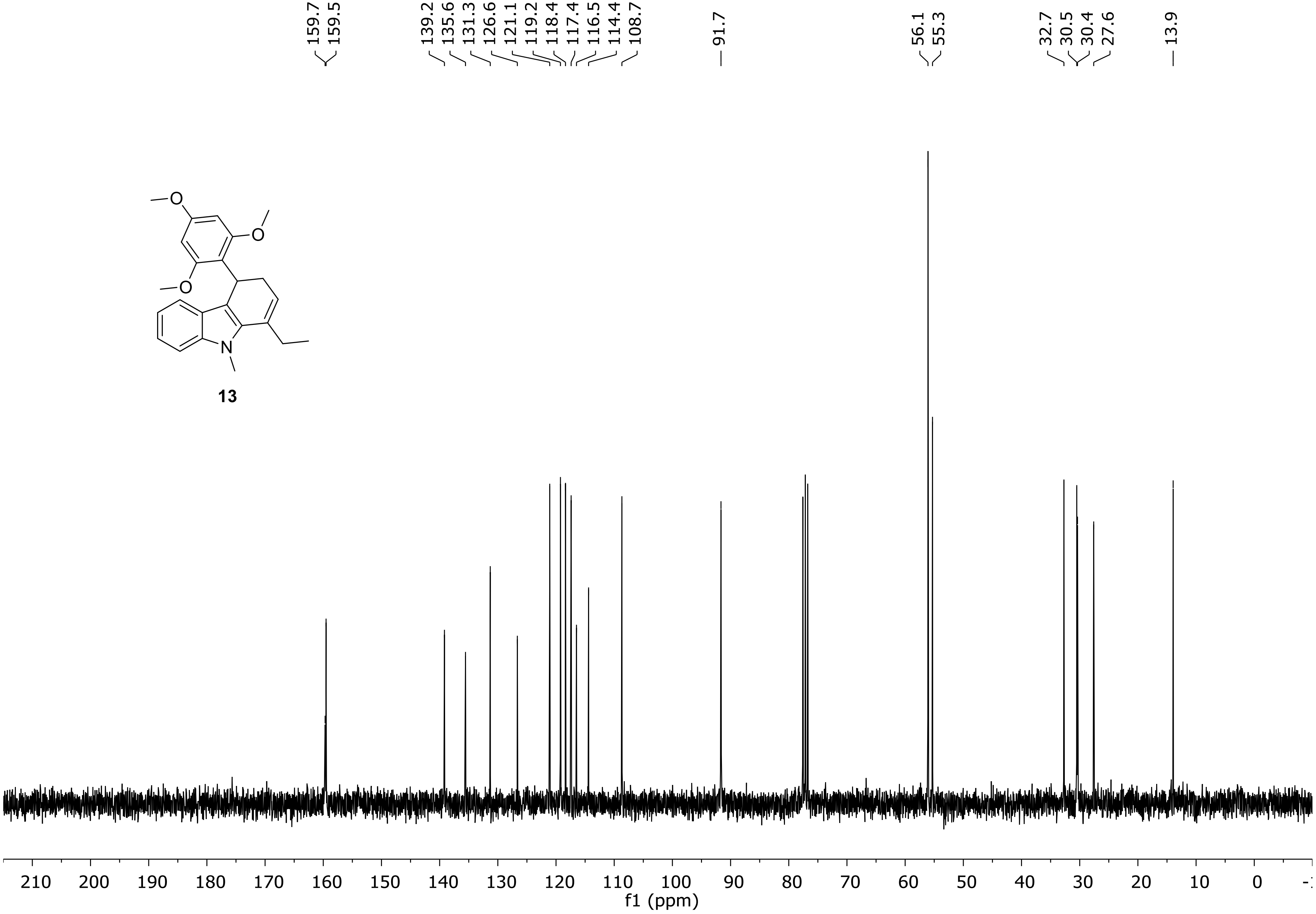

^{1}H-NMR (300 MHz, $CDCl_3$)

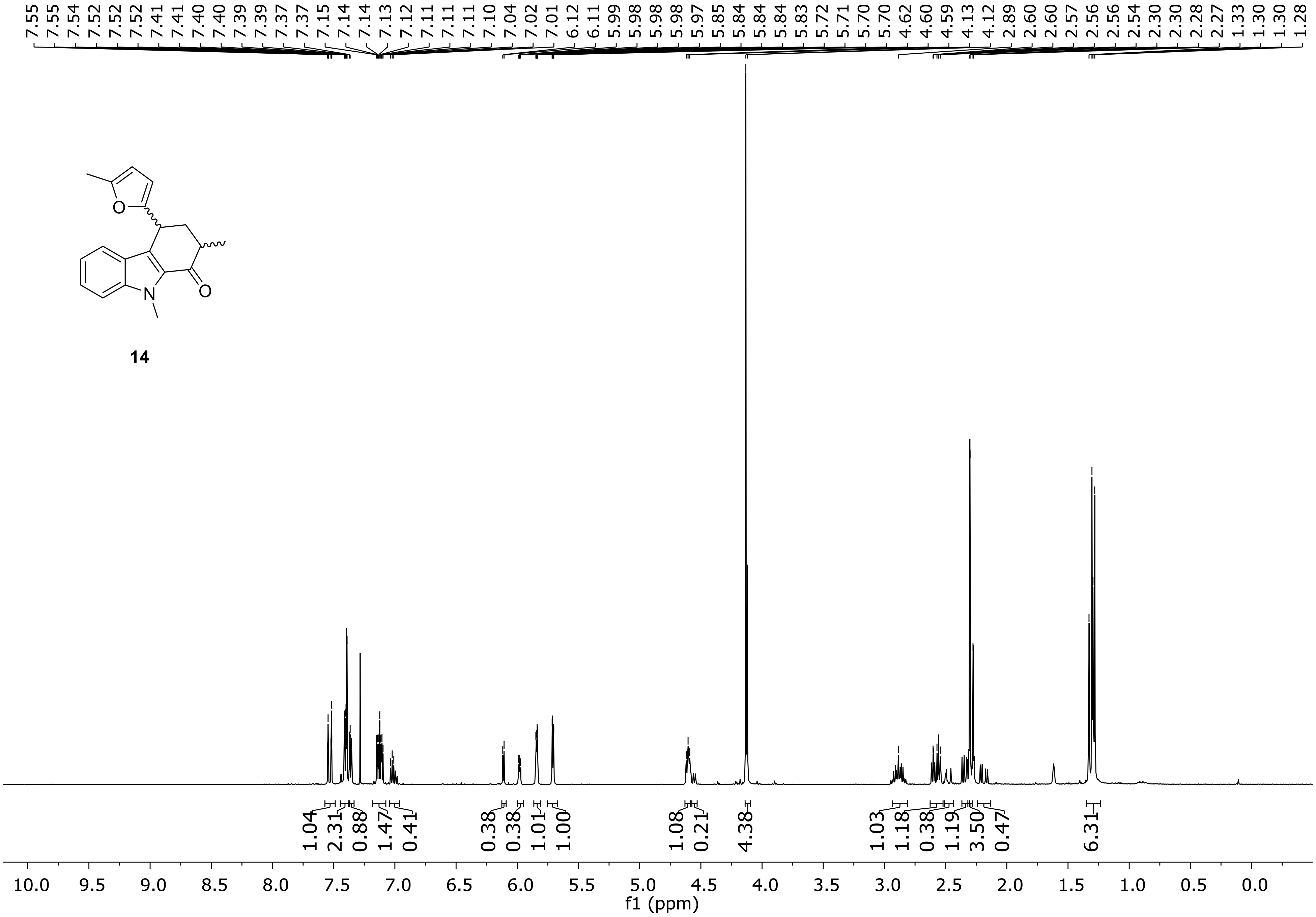

$^{13}C-\{^{1}H\}$NMR (74.5 MHz, $CDCl_3$)

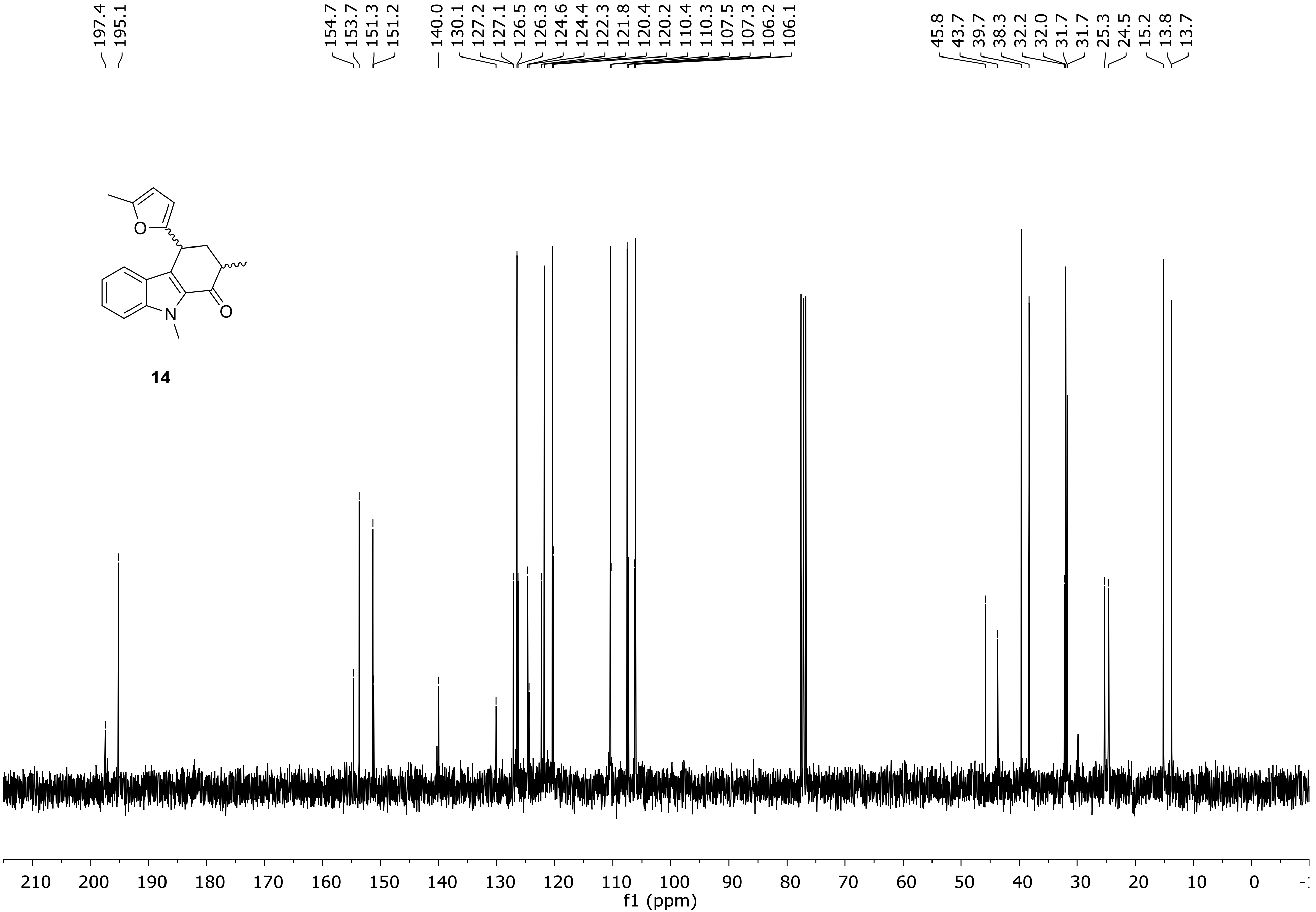

^{1}H-NMR (300 MHz, acetone-d_6)

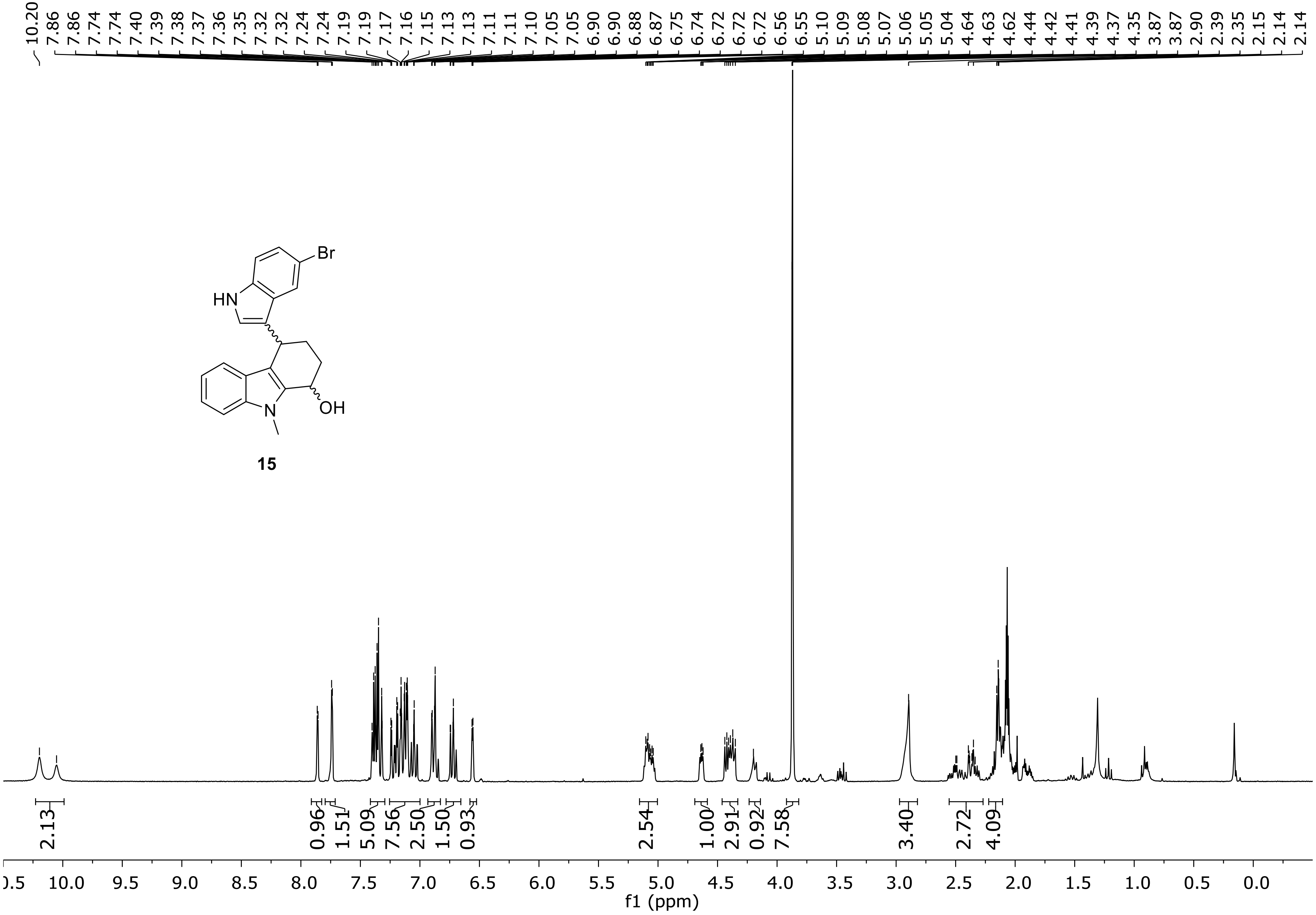

$^{13}C-\{^{1}H\}$NMR (74.5 MHz, acetone-d$_6$)

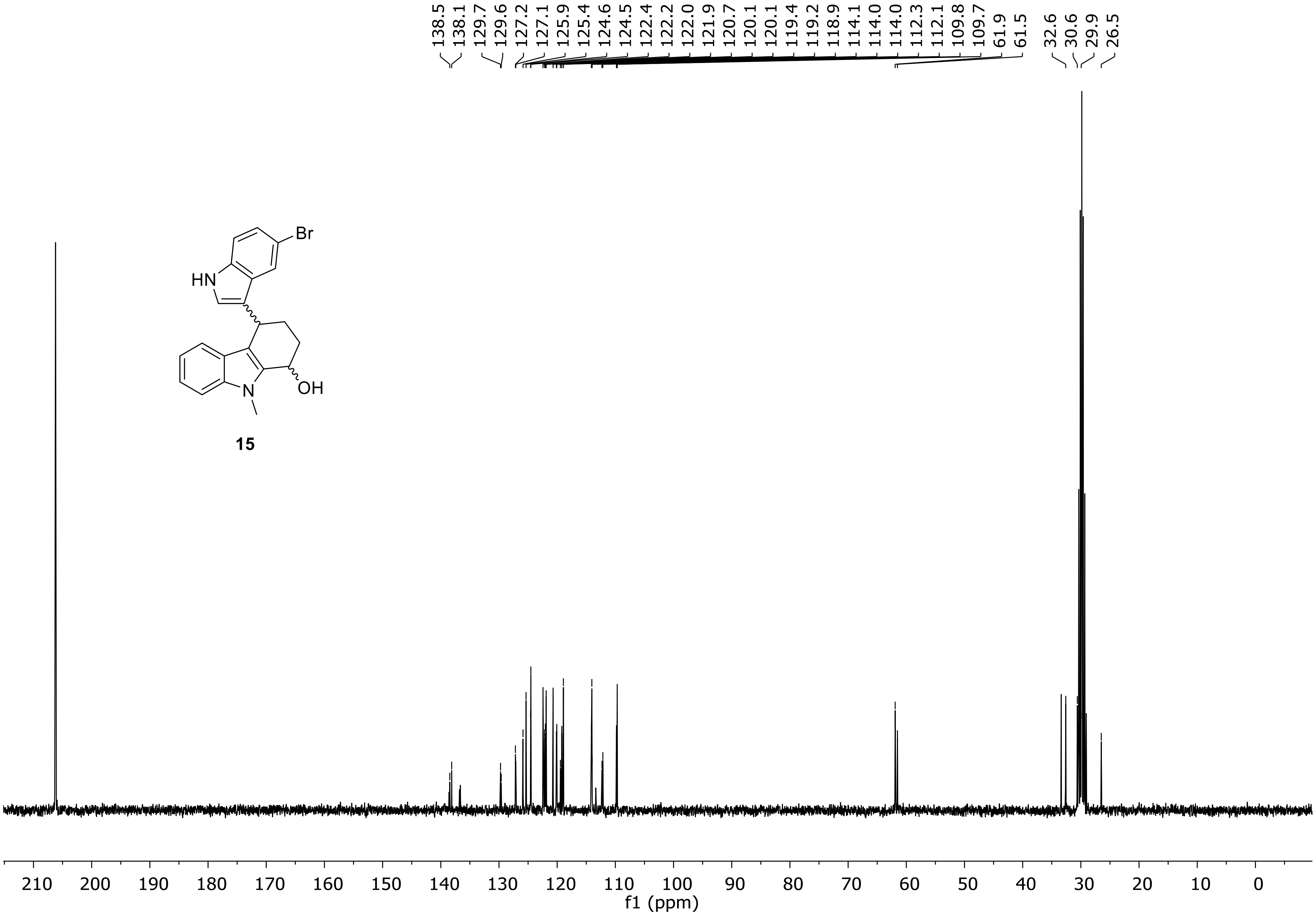

Single Crystal X-ray Diffraction analysis of 8a

CCDC 2307505 contains the supplementary crystallographic data for 4-(1,2-dimethyl-1*H*-indol-3-yl)-9-methyl-2,3,4,9-tetrahydro-1*H*-carbazol-1-one (**8a**). These data are provided free of charge by The Cambridge Crystallographic Data Centre.

Figure S1

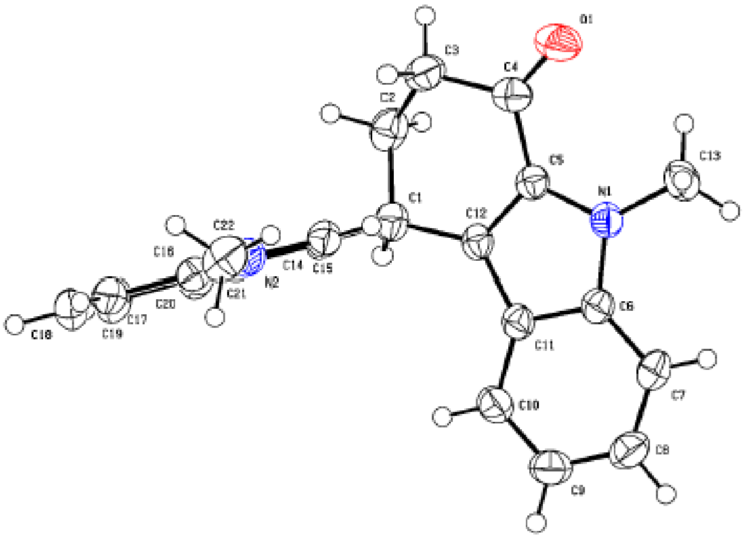

Figure S1: ORTEP Molecular structure of **8a** in the crystal. Displacement ellipsoids correspond to 50% probability.

Crystal structure determination of compound 8a.

Experimental

Single crystals of $C_{22}H_{20}N_2O$ **8a** were grown from a dichloromethane/diethylether solution at 20 °C. A suitable crystal was selected, and data was collected on a **'Bruker APEX-II CCD'** diffractometer. The crystal was kept at 220 (2) K during data collection. Using Olex2,[1] the structure was solved with the ShelXS,[2] structure solution program using Direct Methods and refined with the ShelXL[3] refinement package using Least Squares minimization.

Crystal data and structure refinement for 8a.

Empirical formula	$C_{22}H_{20}N_2O$
Formula weight	328.40
Temperature/K	220(2)
Crystal system	monoclinic
Space group	P1 2_1/c 1
a/Å	9.6792(9)
b/Å	12.8510(12)
c/Å	13.3520(12)
α/°	90
β/°	93.581(4)
γ/°	90
Volume/Å^3	1657.6(3)
Z	4
ρ_{calc}g/cm^3	1.316
μ/mm^{-1}	0.637
F(000)	696.0
T_{min};T_{max}	0.6137;0.7536
Crystal size/mm^3	0.05 × 0.1 × 0.2
Radiation	CuKα (λ = 1.54178)
2Θ range for data collection/°	9.16 to 144.41
Index ranges	$-11 \le h \le 11$, $-15 \le k \le 15$, $-16 \le l \le 16$
Reflections collected	48385
Independent reflections	3252 [R_{int} = 0.0617, R_{sigma} = 0.0229]
Obs. Refl. $F_o \geq 4\sigma(F_o)$	2737
Data/restraints/parameters	3252/0/228
Goodness-of-fit on F^2	1.061
Final R indexes [I>=2σ (I)]	R_1 = 0.0415, wR_2 = 0.0956
Final R indexes [all data]	R_1 = 0.0524, wR_2 = 0.1088
Largest diff. peak/hole / e Å^{-3}	0.191/−0.196

References

1. Dolomanov, O. V.; Bourhis, L. J.; Gildea, R. J.; Howard, J. A. K.; Puschmann H. *J. Appl. Cryst.* **2009**, *42*, 339–341.
2. Sheldrick, G. M. *Acta Cryst.* **2008**, *A64*, 112–122.
3. Sheldrick, G.M. *Acta Cryst.* **2015**, *C71*, 3–8.
