# Supplementary figures and images for: Brønsted Acid-Catalyzed Synthesis of 4-Functionalized Tetrahydrocarbazol-1-ones from 1,4-Dicarbonylindole Derivatives
Source: J Org Chem. 2023 Dec 12;89(1):505–20. doi: 10.1021/acs.joc.3c02248 (PMC10777410; doi:10.1021/acs.joc.3c02248)

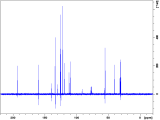

Supplement: Supplementary file 2 — jo3c02248_si_002.zip [file jo3c02248_si_002.zip › 10a/13C NMR/pdata/1/thumb.png]

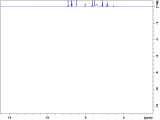

Supplement: Supplementary file 2 — jo3c02248_si_002.zip [file jo3c02248_si_002.zip › 10a/1H NMR/pdata/1/thumb.png]

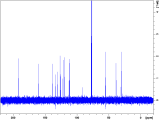

Supplement: Supplementary file 2 — jo3c02248_si_002.zip [file jo3c02248_si_002.zip › 10b/13C NMR/pdata/1/thumb.png]

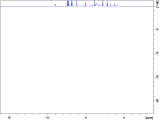

Supplement: Supplementary file 2 — jo3c02248_si_002.zip [file jo3c02248_si_002.zip › 10b/1H NMR/pdata/1/thumb.png]

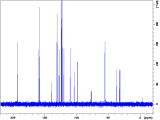

Supplement: Supplementary file 2 — jo3c02248_si_002.zip [file jo3c02248_si_002.zip › 10c/13C NMR/pdata/1/thumb.png]

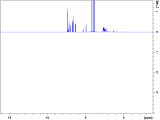

Supplement: Supplementary file 2 — jo3c02248_si_002.zip [file jo3c02248_si_002.zip › 10c/1H NMR/pdata/1/thumb.png]

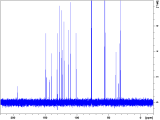

Supplement: Supplementary file 2 — jo3c02248_si_002.zip [file jo3c02248_si_002.zip › 10d/13C NMR/pdata/1/thumb.png]

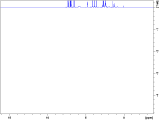

Supplement: Supplementary file 2 — jo3c02248_si_002.zip [file jo3c02248_si_002.zip › 10d/1H NMR/pdata/1/thumb.png]

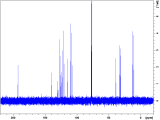

Supplement: Supplementary file 2 — jo3c02248_si_002.zip [file jo3c02248_si_002.zip › 10e/13C NMR/pdata/1/thumb.png]

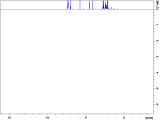

Supplement: Supplementary file 2 — jo3c02248_si_002.zip [file jo3c02248_si_002.zip › 10e/1H NMR/pdata/1/thumb.png]
