# Supplementary material for: Brønsted Acid-Catalyzed Synthesis of 4-Functionalized Tetrahydrocarbazol-1-ones from 1,4-Dicarbonylindole Derivatives
Source: J Org Chem. 2023 Dec 12;89(1):505–20. doi: 10.1021/acs.joc.3c02248 (PMC10777410; doi:10.1021/acs.joc.3c02248)
Supplement: Supplementary file 2 — jo3c02248_si_002.zip [file jo3c02248_si_002.zip › 6d/13C NMR/pdata/1/email_SG-462-F1_20_1.pdf]

UNIVERSIDAD DE BURGOS | Vicerrectorado de Investigación, Transferencia e Innovación

PARQUE CIENTÍFICO TECNOLÓGICO PCT

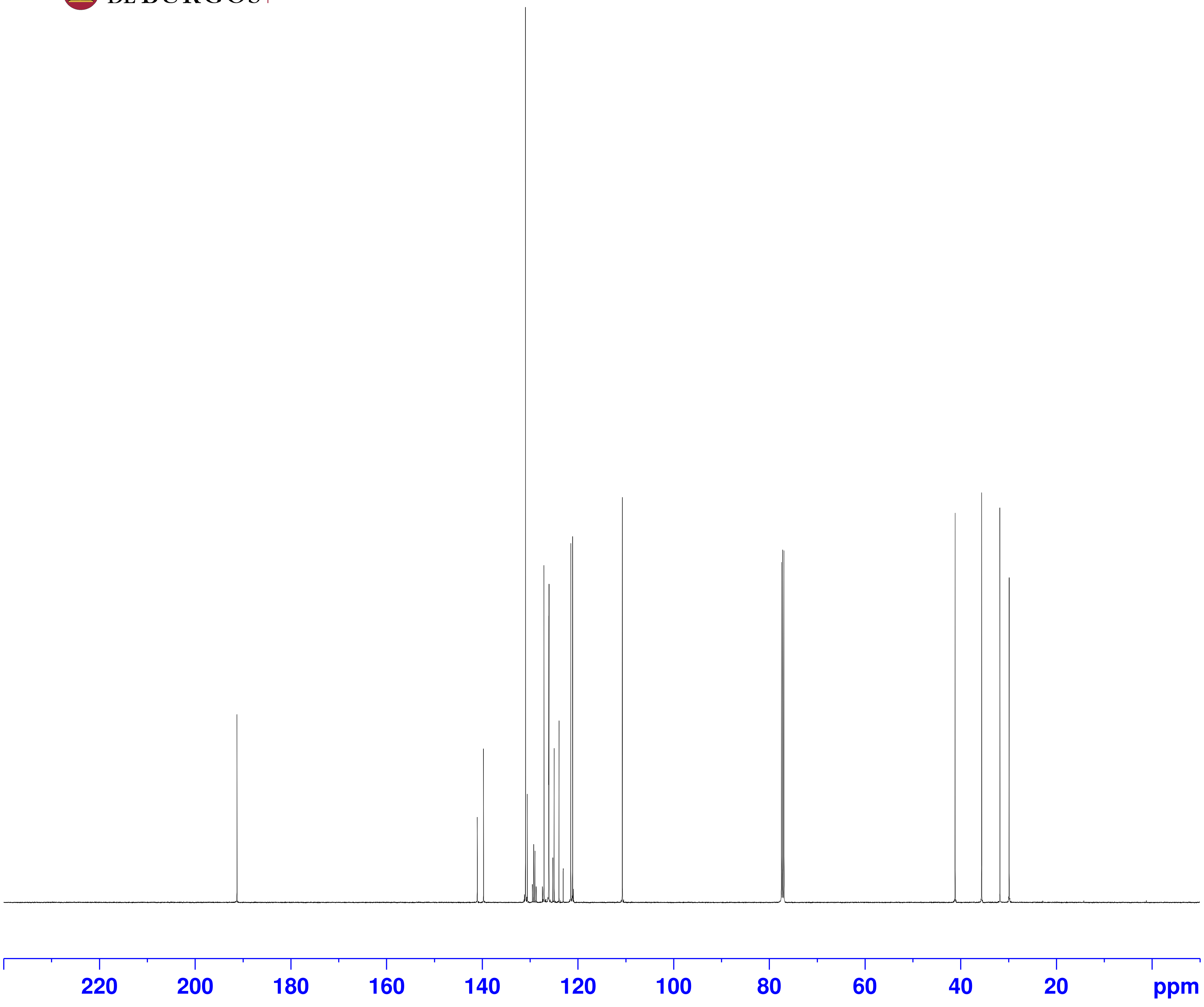

Current Data Parameters		
NAME	SG-462-F1	
EXPNO	20	
PROCNO	1	
F2 - Acquisition Parameters		
Date_	20230613	
Time	9.35	h
INSTRUM	AV NEO	
PROBHD	Z151574_0146 (	
PULPROG	zgpg30	
TD	65536	
SOLVENT	CDCl3	
NS	7500	
DS	4	
SWH	32679.739	Hz
FIDRES	0.997306	Hz
AQ	1.0027008	sec
RG	101	
DW	15.300	usec
DE	6.50	usec
TE	298.0	K
D1	2.00000000	sec
D11	0.03000000	sec
TD0	1	
SFO1	125.7791662	MHz
NUC1	13C	
P0	3.00	usec
P1	9.00	usec
PLW1	103.20999908	W
SFO2	500.1620006	MHz
NUC2	1H	
CPDPRG[2	waltz65	
PCPD2	80.00	usec
PLW2	26.31999969	W
PLW12	0.25084999	W
PLW13	0.12616999	W
F2 - Processing parameters		
SI	32768	
SF	125.7653220	MHz
WDW	EM	
SSB	0	
LB	1.00	Hz
GB	0	
PC	1.40	
