# Supplementary material for: Brønsted Acid-Catalyzed Synthesis of 4-Functionalized Tetrahydrocarbazol-1-ones from 1,4-Dicarbonylindole Derivatives
Source: J Org Chem. 2023 Dec 12;89(1):505–20. doi: 10.1021/acs.joc.3c02248 (PMC10777410; doi:10.1021/acs.joc.3c02248)
Supplement: Supplementary file 2 — jo3c02248_si_002.zip [file jo3c02248_si_002.zip › 6d/1H NMR/pdata/1/email_SG-462-F1_10_1.pdf]

UNIVERSIDAD DE BURGOS | Vicerrectorado de Investigación, Transferencia e Innovación

PARQUE CIENTÍFICO TECNOLÓGICO PCT

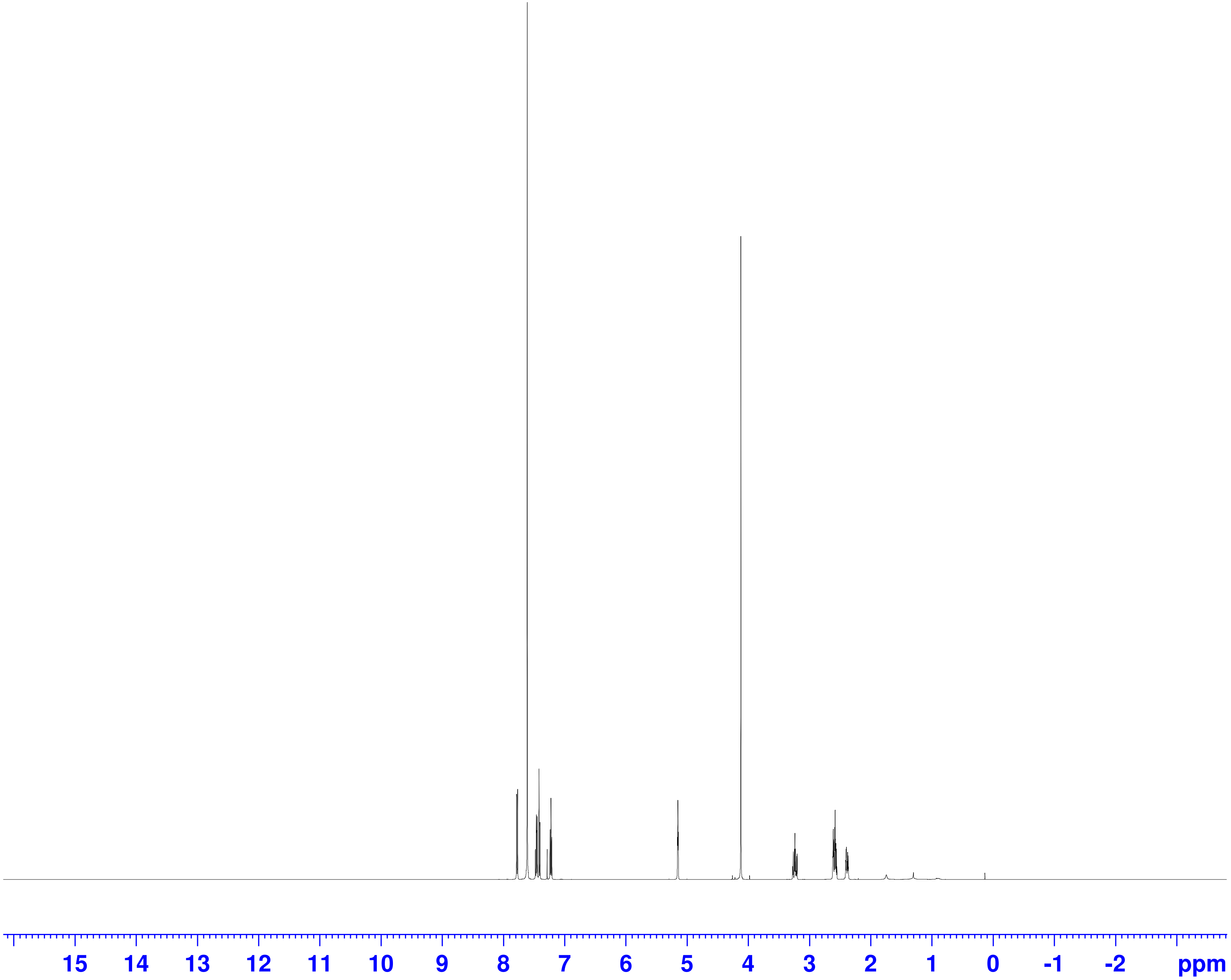


```
Current Data Parameters
NAME             SG-462-F1
EXPNO                   10
PROCNO                   1

F2 - Acquisition Parameters
Date_             20230613
Time                  3.10 h
INSTRUM             AV NEO
PROBHD    Z151574_0146 (
PULPROG               zg30
TD                   65536
SOLVENT              CDCl3
NS                      16
DS                       2
SWH              10000.000 Hz
FIDRES            0.305176 Hz
AQ               3.2767999 sec
RG                      57
DW                  50.000 usec
DE                   12.17 usec
TE                   298.0 K
D1              1.00000000 sec
TD0                      1
SFO1           500.1630885 MHz
NUC1                    1H
P0                    2.60 usec
P1                    7.81 usec
PLW1           26.31999969 W

F2 - Processing parameters
SI                   65536
SF             500.1600000 MHz
WDW                     EM
SSB                      0
LB                    0.30 Hz
GB                       0
PC                    1.00
```
